# Puumala Hantavirus Genotypes in Humans, France, 2012–2016

**DOI:** 10.3201/eid2501.180270

**Published:** 2019-01

**Authors:** Jean-Marc Reynes, Damien Carli, Damien Thomas, Guillaume Castel

**Affiliations:** Institut Pasteur, Lyon, France (J.-M. Reynes, D. Carli, D. Thomas);; CBGP, INRA, CIRAD, IRD, Montpellier SupAgro, Université Montpellier, Montpellier, France (G. Castel)

**Keywords:** France, Puumala virus, orthohantavirus, humans, genotype, viruses, zoonoses, France, hantavirus

## Abstract

The analysis of the nucleoprotein gene of 77 Puumala hantavirus strains detected in human samples in France during 2012–2016 showed that all belonged to the Central European lineage. We observed 2 main clusters, geographically structured; one included strains with the Q64 signature and the other strains with the R64 signature.

Puumala virus (PUUV) is the main hantavirus detected in Europe. Several variants of this enveloped trisegmented RNA virus species have been reported, comprising PUUV stricto sensu, detected in humans, and the Asian Hokkaido and Muju viruses, not yet detected in humans. PUUV variant, hosted in the wild by the bank vole (*Myodes glaerolus*), is responsible for a mild hemorrhagic fever with renal syndrome called nephropathia epidemica (*1**,**2*). Within the variant PUUV, 8 lineages have been described, according to phylogenetic analysis of the small (S) RNA segment coding domain sequence (CDS) of the strains. Each lineage is constituted by well-supported and geographically structured clusters of variants, supporting the hypothesis of a hantavirus/host co-evolution (*3**,**4*). Furthermore, it has been shown that this genetic diversity may affect the molecular detection of PUUV in humans (*5*). Consequently, the genotyping of local PUUV strains is also essential for laboratory diagnostics.

PUUV is also the main hantavirus detected in France. Approximately 100 human hospitalized cases are detected annually, all of them located in the northeastern part of the country (*6*). Sequences of 7 PUUV strains have been studied so far, and all were detected in bank voles (*7**,**8*). We report the analysis of the S segment CDS for the nucleoprotein N from 77 additional strains detected in human cases in France during 2012–2016.

## The Study

As part of our surveillance assignment, using serologic and molecular assays, we detected 470 laboratory-confirmed human hantavirus cases from 2012–2016 in France, including Tula virus (n = 1), Seoul virus (n = 6), and PUUV (n = 228) infections (*6*). We did not obtain strains from all samples for several reasons, including the absence of molecular testing because of lack of samples, samples being taken too long after the date of onset, and inadequate sample storage temperature before reception in our laboratory. We detected 162 PUUV strains using both real-time PCR and nested reverse transcription PCR (RT-PCR) (*9**,**10*), 33 using real-time PCR, and 33 using nested RT-PCR. The discordance of results between the 2 techniques could be explained by a viral load in the sample close to our limit of detection. We identified these 228 PUUV strains in 28 of the 34 hantavirus-endemic departments (administrative divisions) in France (*6*). We used 3 overlapping heminested RT-PCRs to obtain the S segment sequences. After sequencing by Sanger method, we obtained the entire S CDS from 77 strains coming from 17 departments (Appendix Tables 1,2). We deposited PUUV sequences in GenBank (accession nos. MG923598–MG923674). We mapped strains according to the municipality of exposure. We used ClustalW and Muscle implemented in MEGA7 (https://www.megasoftware.net) (*8*) to align the S segment CDS and to deduce aa sequences from these strains and from 115 other PUUV strains published and available in GenBank (as of December 1, 2017). We then ordered the sequences according to aa similarity. We conducted maximum-likelihood phylogenetic analysis with 1,000 bootstrap replicates by using PhyML version 3.0 (http://www.atgc-montpellier.fr/phyml) implemented in Seaview version 4.6.1 (*8*), on the basis of the S segment CDS using a representative sample in which sequences were identical at the aa level, to reduce the size of the tree. We also performed phylogenetic analysis on the generalized time-reversible model with a gamma distribution (GTR+Γ) with 4 rate categories, with the assumption that a certain fraction of sites are evolutionarily invariable according to the best-fit substitution model proposed by SMS version 1.8.2, available online on the ATGC bioinformatics platform (http://www.atgc-montpellier.fr/sms/).

**Figure 1:**
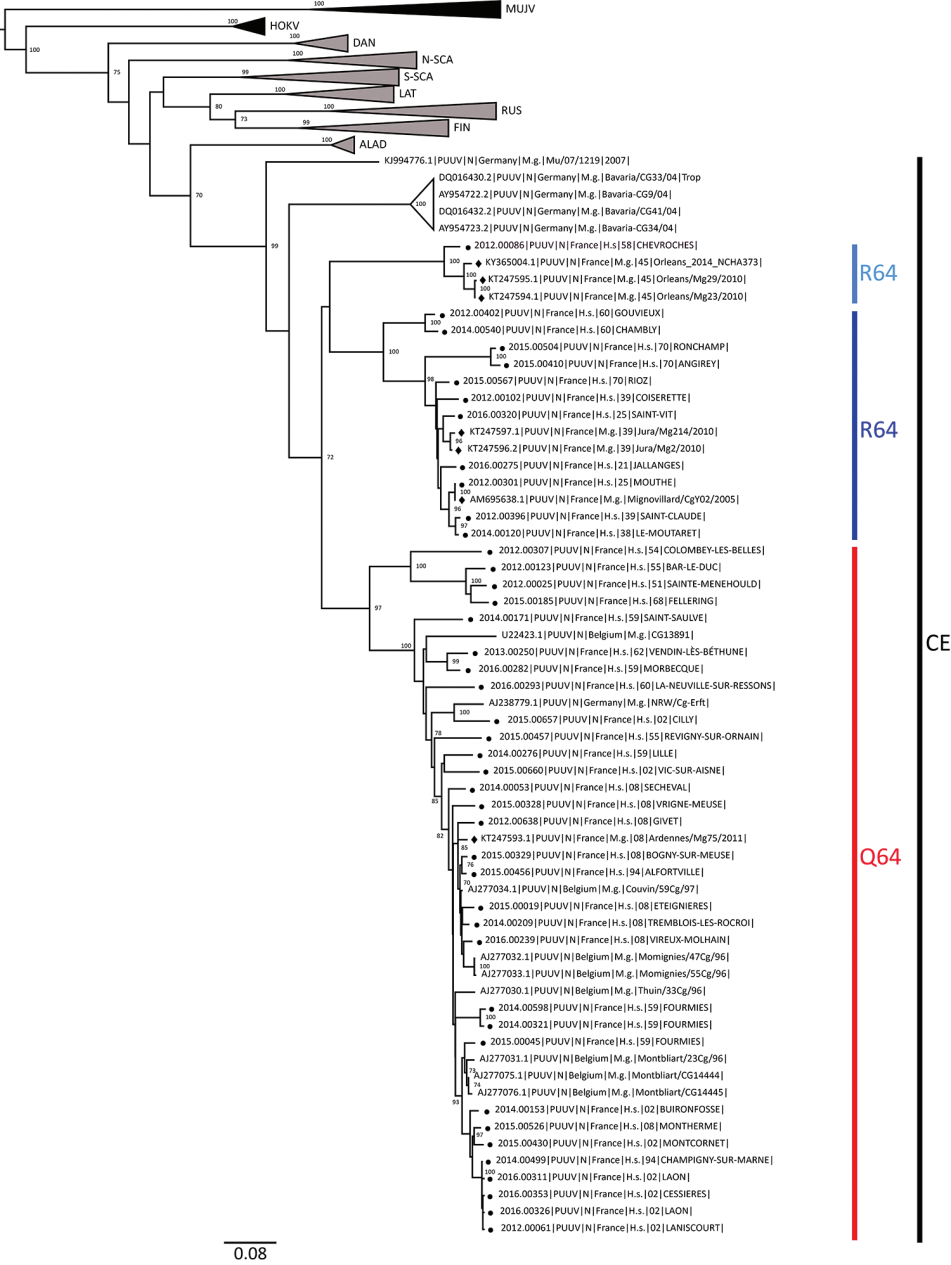
Phylogenetic tree constructed using the maximum-likelihood approach based on the complete small-segment RNA nucleotide coding sequences of representative Puumala virus (PUUV) strains detected in human cases in France, 2012–2016 (circles), and on those published and representative of PUUV strains detected in Europe. Diamonds indicate sequences of strains detected in rodents as reported elsewhere (*7**,**8*). Bootstrap percentages >70% (from 1,000 resamplings) are indicated at each node; GenBank accession numbers are indicated for reference strains. Scale bar indicates nucleotide substitutions per site. ALAD, Alpe-Adrian lineage; CE, Central European lineage; DAN, Danish lineage; FIN, Finnish lineage; LAT, Latvian lineage; N-SCA, north-Scandinavian lineage; S-SCA; south-Scandinavian lineage; RUS, Russian lineage; MUJV, Muju virus; HOKV, Hokkaido virus.

**Figure 2:**
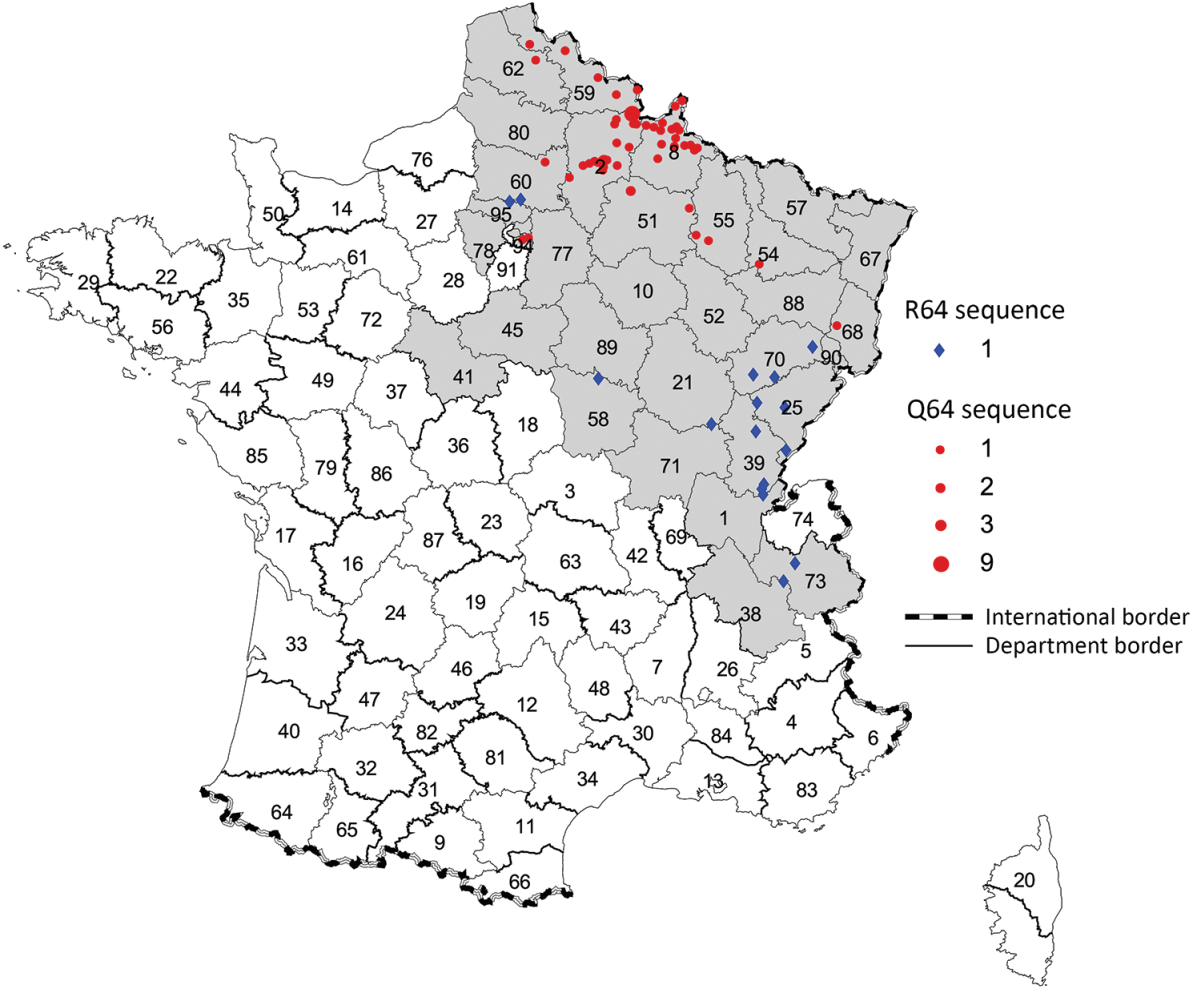
Location of the Puumala virus small segment RNA coding domain sequence sublineages Q64 and R64 detected in human cases, by municipality of exposure, France, 2012–2016. Gray shading indicates the hantavirus-endemic area; red circles indicate Q64 sequences, by size; blue diamonds indicate R64 sequences.

The phylogenetic analysis showed that the PUUV S CDS were grouped into the 8 lineages as previously described (*3**,**4*). All of the PUUV sequences from France detected in humans belonged to the Central European lineage (Figure 1). We distinguished 2 main sublineages, the first harboring the aa signature Q64 and including strains from Belgium and Germany (*3*), and the second with strains harboring an arginine (R) amino acid at position 64. Within this cluster, strains isolated from bank voles in the Loiret department (coded 45) cluster together with branch support of 100; they share the K258 signature (*8*) with the human strain 2012.00086 isolated in the Nièvre department (coded 58), neighbor of the Loiret department (Figure 1). Of note, strains from the Q64 cluster were from the northeast part of the endemic region, whereas the strains from the R64 clusters were detected in the south of the endemic area, except for strains carrying the I276 residue; those came from the northwest part of the endemic region in the Oise department (coded 60), where the Q64 cluster was co-circulating (Figure 2).

Several real-time RT-PCRs targeting the PUUV S segment have been implemented to diagnose PUUV infection in humans (*5**,**9**,**11**–**14*). We detected some mismatches between the sequences of the France strains and those of the primers and probes (for example, at the 3′ end of the forward primer designed by Garin D et al. [*12*]), which could jeopardize the detection of some PUUV strains from France (Appendix Figure). However, we did not perform assays to evaluate this hypothesis.

## Conclusions

Genomic findings from this study revealed that the 77 PUUV strains studied and detected in humans in France during 2012–2016 all belonged to the Central European lineage. Within this lineage, the strains were clustered within 2 sublineages, with marked geographical patterns: the first included strains with the Q64 signature, a hallmark of strains from Belgium, and the second carried an arginine (R) at position 64. These results confirm the clustering observed within the 7 PUUV strains from France so far studied and obtained from rodents (*7*,*8*) and highlight the geographic diversity of strains from France within this lineage.

We were unable to obtain the S segment CDS from all strains, especially from the few strains detected in the departments (e.g., 10, 52, 67, 89) located between the northeastern part and the southeastern part of the hantavirus-endemic area; these 2 parts, reported as main endemic areas in Reynes et al. (*6*), were overrepresented in our dataset. The lack of amplification could be a result of improper storage of the specimens for several days at 4°C by peripheral laboratories for serologic diagnostic testing before sending to the National Reference Center; the low viral load observed in the specimens at reception (crossing point >35); the limit of detection of our S CDS overlapping heminested RT-PCR (reduced 10-fold, compared with detection limit using our molecular diagnostic methods); or mismatch between the sequences of PUUV strains and those of our primers used for S segment CDS amplification. Therefore, other clusters may be present in France, and we cannot clearly determine the geographic overlap of the clusters we described. Furthermore, our study was limited to the S segment CDS, whereas it has been shown that intercluster reassortment of the small, medium, and large segments in PUUV strains can occur, which could be the case in the area in which the 2 sublineages co-circulate (*15*).

Future efforts will be focused on more efficient sequencing of PUUV S segment CDS and also M and L segment CDS, using next-generation sequencing and amplicon approaches for samples with low viral load. We hope to identify more sequences from more geographic areas for use in molecular diagnostic development and in implementing a comprehensive phylogeographic analysis, permitting a better molecular description of PUUV strains in France and further investigation of their evolution.

Appendix Additional information about Puumala virus in humans in France.
