# Supplementary material for: Puumala Hantavirus Genotypes in Humans, France, 2012–2016
Source: Emerg Infect Dis. 2019 Jan;25(1):140–3. doi: 10.3201/eid2501.180270 (PMC6302603; doi:10.3201/eid2501.180270)
Supplement: Appendix — Additional information about Puumala virus in humans in France. [file 18-0270-Techapp-s1.pdf]

Article DOI: https://doi.org/10.3201/eid2501.180270

Puumala Hantavirus Genotypes in Humans, France, 2012–2016

Appendix

Appendix Table 1. Results of the complete S coding domain sequence acquisition among the 228 Puumala virus strains detected in humans, France, 2012–2016

Administrative territories		Results					
Region	Department (code)*	No. complete CDS	No. partial CDS	No. amplification failure	No samples not tested	No. samples unavailable	Total
Hauts-de-France	Ain (02)	18	10	3	30	4	65
	Nord (59)	15	5	4	0	9	33
	Oise (60)	3	3	1	0	1	8
	Pas-de-Calais (62)	1	0	1	0	2	4
	Somme (80)	0	0	0	0	0	0
Grand-Est	Ardennes (08)	17	4	3	21	0	45
	Aube (10)	0	1	0	0	0	1
	Marne (51)	3	2	1	0	3	9
	Haute-Marne (52)	0	0	0	0	1	1
	Meurthe-et-Moselle (54)	1	1	0	0	0	2
	Meuse (55)	2	0	0	0	2	4
	Moselle (57)	0	0	0	0	1	1
	Bas-Rhin (67)	0	0	0	0	1	1
	Haut-Rhin (68)	1	0	0	0	1	2
	Vosges (88)	0	0	0	0	0	0
Ile-de-France	Paris (75)	0	0	0	0	0	0
	Seine-et-Marne (77)	0	1	1	0	0	2
	Yvelines (78)	0	1	1	0	0	2
	Hauts-de-Seine (92)	0	0	0	0	1	1
	Seine-Saint-Denis (93)	0	1	0	0	0	1
	Val-de-Marne (94)	2	0	0	0	0	2
	Val-d'Oise (95)	0	0	0	0	0	0
Centre Val-de-Loire	Loiret (45)	0	0	0	0	1	1
Bourgogne Franche-Comté	Côte d'Or (21)	1	0	1	0	3	5
	Doubs (25)	3	2	5	0	3	13
	Jura (39)	4	0	2	0	6	12
	Nièvre (58)	1	0	1	0	0	2
	Haute-Saône (70)	3	0	1	0	2	6
	Saône-et-Loire (71)	0	0	0	0	0	0
	Yonne (89)	0	0	2	0	0	2
	Territoire de Belfort (90)	0	1	0	0	0	1
Auvergne Rhône-Alpes	Ain (1)	0	0	0	0	0	0
	Isère (38)	1	0	0	0	0	1
	Savoie (73)	1	0	0	0	0	1
Total		77	32	27	51	41	228

* The 34 departments listed are the hantavirus-endemic departments (*1*).

Appendix Table 2. Primers used in the 3 overlapping heminested RT-PCR assays performed to obtain the PUUV S CDS*

Heminested RT-PCR	Step	Product size, bp	Primers	Positions	Sequences (5′-3′)
1	RT-PCR	920	PUUV-S-9 F	9–31	GAC TCC TTG AAR AGY TRC TAC GA
			PUUV-S-928 R	928–909	CTC CTG ARG CTG CRT ART CA
	Nested PCR	700	PUUV-S-9 F	9–31	GAC TCC TTG AAR AGY TRC TAC GA
			PUUV-S-708R	708–689	KAC TCC CAT YAC AGG RCT CA
2	RT-PCR	725	PUUV-S-376 F	376–395	CCR AGT GGY CAA ACA GCR GA
			PUUV-F-1100 R	1100–1079	CCA ACA GTT TTR GAH GCC ATR A
	Nested PCR	629	PUUV-S-472 F	472–491	CGT GGG AGA CAR ACT GTR AA
			PUUV-S-1100 R	376–395	CCR AGT GGY CAA ACA GCR GA
3	RT-PCR	917	PUUV-S-919 F	919–938	GCY TCA GGA GAT CCD ACA TC
			PUUV-S-1835 R	1835–1812	CCT TGA AAA GCA ATC AAG AAA TC
	Nested PCR	599	PUUV-S-919 F	919–938	GCY TCA GGA GAT CCD ACA TC
			PUUV-S-1517 R	1517–1494	YYT AYT TCC AGK TAA ACC CTG ATT

*Positions according to the sequence GenBank accession no U22423.1. RT-PCR, reverse transcription PCR.

Appendix Figure. Alignment of the small ARN coding domain sequences obtained from Puumala virus strains detected in humans, France, 2012–2016. The first 2 lines of the alignment colorized in red are repeated at each page change. Positions and sequences of primers and probes used to detect Puumala virus by real time RT-PCR are indicated (*2*–*7*), as well as those used to detect several hantavirus species, including Puumala virus, by nested RT-PCR (*8*).

Kramski M et al. Clinical Chemistry 2007 PUUV F GARR

Garin D et al Microb. Infect 2001 PUF1 GAAAAAAACTGGGATGAGTGACTTG CRRSAp ATAACCCGCCATGAACAACAGCTTG ACAACGGTCTGTTTTCGAGTT PUR1

```
                                                             *        20         *        40         *        60         *        80         *       100
2014.00598|PUUV|N|France|H.s.|59|FOURMIES|          : ATGAGTGACTTGACAGACATTCAGGAGGATATAACCCGCCATGAACAACAACTTGTTGTTGCCAGACAGAAACTCAAGGATGCAGAAAAGGCGGTGGAGA : 100
2014.00233|PUUV|N|France|H.s.|59|FOURMIES|          : ..............................................................T..................................... : 100
2014.00097|PUUV|N|France|H.s.|02|SAINT-MICHEL|      : .................................................................................................... : 100
2014.00488|PUUV|N|France|H.s.|08|SIGNY-LE-PETIT|    : ..................................................................................................A. : 100
2014.00613|PUUV|N|France|H.s.|59|FOURMIES|          : .................................................................................................... : 100
2015.00402|PUUV|N|France|H.s.|08|CHARLEVILLE-MEZIE  : .................................................................................................... : 100
2015.00422|PUUV|N|France|H.s.|59|ANOR|              : .................................................................................................... : 100
2015.00488|PUUV|N|France|H.s.|08|SEDAN|             : .................................................................................................... : 100
2015.00498|PUUV|N|France|H.s.|08|REMILLY-AILLICOUR  : .................................................................................................... : 100
2016.00295|PUUV|N|France|H.s.|08|CORNY-MACHEROMENI  : .................................................................................................... : 100
2016.00333|PUUV|N|France|H.s.|59|FOURMIES|          : .................................................................................................... : 100
2016.00345|PUUV|N|France|H.s.|59|FOURMIES|          : .................................................................................................... : 100
2016.00357|PUUV|N|France|H.s.|02|HIRSON|            : .................................................................................................... : 100
2016.00427|PUUV|N|France|H.s.|59|FOURMIES|          : .................................................................................................... : 100
2016.00469|PUUV|N|France|H.s.|02|PREMONTRE|         : .......................................................................G..............G............. : 100
AJ277075.1|PUUV|N|Belgium|M.g.|Montbliart/CG14444|  : ......................................T............................................................. : 100
2014.00053|PUUV|N|France|H.s.|08|SECHEVAL|          : .................................................................................................... : 100
KT247593.1|PUUV|N|France|M.g.|08|Ardennes/Mg75/201  : .........................................................................................A..A....... : 100
KT247592.1|PUUV|N|France|M.g.|08|Ardennes/Mg156/20  : ............................................................................................A....... : 100
2015.00019|PUUV|N|France|H.s.|08|ETEIGNIERES|       : .................................................................................................... : 100
2016.00239|PUUV|N|France|H.s.|08|VIREUX-MOLHAIN|    : .................................................................................................... : 100
2015.00329|PUUV|N|France|H.s.|08|BOGNY-SUR-MEUSE|   : .................................................................................................... : 100
2014.00209|PUUV|N|France|H.s.|08|TREMBLOIS-LES-ROC  : .................................................................................................... : 100
2015.00456|PUUV|N|France|H.s.|94|ALFORTVILLE|       : .................................................................................................... : 100
2012.00018|PUUV|N|France|H.s.|08|ROCROI|            : .................................................................................................... : 100
AJ277032.1|PUUV|N|Belgium|M.g.|Momignies/47Cg/96|   : .................................................................................................... : 100
AJ277034.1|PUUV|N|Belgium|M.g.|Couvin/59Cg/97|      : .................................................................................................... : 100
AJ277033.1|PUUV|N|Belgium|M.g.|Momignies/55Cg/96|   : .................................................................................................... : 100
2012.00638|PUUV|N|France|H.s.|08|GIVET|             : .................................................................................................... : 100
2015.00328|PUUV|N|France|H.s.|08|VRIGNE-MEUSE|      : ....................................................................A............................... : 100
2015.00419|PUUV|N|France|H.s.|08|DOUZY|             : .................................................................................................... : 100
2016.00310|PUUV|N|France|H.s.|08|THIN-LE-MOUTIER|   : .................................................................................................... : 100
AJ277030.1|PUUV|N|Belgium|M.g.|Thuin/33Cg/96|       : .................................................................................................... : 100
AJ238779.1|PUUV|N|Germany|M.g.|NRW/Cg-Erft|         : ....................................................................A..G....................A....... : 100
2014.00276|PUUV|N|France|H.s.|59|LILLE|             : .....................................................C.................G..............G............. : 100
U22423.1|PUUV|N|Belgium|M.g.|CG13891|               : ..................................................G.................A..G............................ : 100
2014.00321|PUUV|N|France|H.s.|59|FOURMIES|          : .................................................................................................... : 100
2015.00457|PUUV|N|France|H.s.|55|REVIGNY-SUR-ORNAI  : .......................................................................G............................ : 100
2013.00250|PUUV|N|France|H.s.|62|VENDIN-LS-BTHUNE|  : .......................A..A........T................................A............................... : 100
2016.00282|PUUV|N|France|H.s.|59|MORBECQUE|         : ..........................A........T................................A.....T........................G : 100
AJ277076.1|PUUV|N|Belgium|M.g.|Montbliart/CG14445|  : ......................................T............................................................. : 100
2014.00153|PUUV|N|France|H.s.|02|BUIRONFOSSE|       : ....................................................................A............................... : 100
2014.00171|PUUV|N|France|H.s.|59|SAINT-SAULVE|      : ...........A..............A.........................................A..G.....A.....G........A....... : 100
2016.00293|PUUV|N|France|H.s.|60|LA-NEUVILLE-SUR-R  : .......................A..A.........................................A..G.................A........A. : 100
2014.00499|PUUV|N|France|H.s.|94|CHAMPIGNY-SUR-MAR  : ....................................................................A............................... : 100
2012.00057|PUUV|N|France|H.s.|59|COUSOLRE|          : ......................................T............................................................. : 100
2012.00349|PUUV|N|France|H.s.|02|ENGLANCOURT|       : ....................................................................A............................... : 100
2014.00135|PUUV|N|France|H.s.|02|ATHIES-SOUS-LAON|  : ....................................................................A............................... : 100
2014.00184|PUUV|N|France|H.s.|59|FOURMIES|          : ......................................T.......................T..................................... : 100
2016.00182|PUUV|N|France|H.s.|08|BOULZICOURT|       : ......................................T...........G................................................. : 100
2016.00286|PUUV|N|France|H.s.|02|TRUCY|             : ....................................................................A............................... : 100
2016.00325|PUUV|N|France|H.s.|59|AULNOYE-AYMERIES|  : ......................................T.......................T..............A...................... : 100
2016.00452|PUUV|N|France|H.s.|02|LAON|              : ....................................................................A............................... : 100
2016.00467|PUUV|N|France|H.s.|02|AISNE|             : ....................................................................A.......................A....... : 100
```

```
                                                                                                          Kramski M et al. Clinical Chemistry 2007  PUUV F  GARR
Garin D et al Microb. Infect 2001  PUF1 GAAAAAAACTGGGATGAGTGACTTG         CRRSAp   ATAACCCGCCATGAACAACAGCTTG ACAACGGTCTGTTTTCGAGTT  PUR1

                                                                  *        20         *        40         *        60         *        80         *       100
2014.00598|PUUV|N|France|H.s.|59|FOURMIES|         : ATGAGTGACTTGACAGACATTCAGGAGGATATAACCCGCCATGAACAACAACTTGTTGTTGCCAGACAGAAACTCAAGGATGCAGAAAAGGCGGTGGAGA :  100
2015.00657|PUUV|N|France|H.s.|02|CILLY|            : .................T.................T................................A..G..............G.....A....... :  100
2015.00526|PUUV|N|France|H.s.|08|MONTHERME|        : .................................................................................................... :  100
2015.00045|PUUV|N|France|H.s.|59|FOURMIES|         : ......................................T............................................................. :  100
2015.00430|PUUV|N|France|H.s.|02|MONTCORNET|       : ....................................................................A............................... :  100
2016.00311|PUUV|N|France|H.s.|02|LAON|             : ....................................................................A............................... :  100
AJ277031.1|PUUV|N|Belgium|M.g.|Montbliart/23Cg/96| : ......................................T............................................................. :  100
2016.00326|PUUV|N|France|H.s.|02|LAON|             : ............G.......................................................A............................... :  100
2016.00353|PUUV|N|France|H.s.|02|CESSIERES|        : ....................................................................A.......................A....... :  100
2012.00061|PUUV|N|France|H.s.|02|LANISCOURT|       : ....................................................................A.....T......................... :  100
2014.00174|PUUV|N|France|H.s.|02|ST-ERME-OUTRE-ET- : ....................................................................A............................... :  100
2016.00268|PUUV|N|France|H.s.|02|PRESLES-ET-THIERN : ....................................................................A............................... :  100
2015.00660|PUUV|N|France|H.s.|02|VIC-SUR-AISNE|    : ..........................A............................................G............................ :  100
2012.00025|PUUV|N|France|H.s.|51|SAINTE-MENEHOULD| : .................T.....A...................................G........A..G..T.................A.....A. :  100
2015.00665|PUUV|N|France|H.s.|51|REIMS|            : .................T................................G........G.....G..A..G..............G.....A.....A. :  100
2012.00307|PUUV|N|France|H.s.|54|COLOMBEY-LES-BELL : ............G....T..................................................A..G....................A.....A. :  100
2012.00123|PUUV|N|France|H.s.|55|BAR-LE-DUC|       : .................T.................T.......................G........A..G..T...........G...........A. :  100
2012.00278|PUUV|N|France|H.s.|51|REIMS|            : .................T................................G..............G..A..G..T...........G.....A.....A. :  100
2015.00185|PUUV|N|France|H.s.|68|FELLERING|        : .................T.........................................G..T.....A..G..T.................A.....A. :  100
KJ994776.1|PUUV|N|Germany|M.g.|Mu/07/1219|2007|    : ...........A...........A................................G........G..A..G..T...........G.....A....... :  100
2012.00086|PUUV|N|France|H.s|58|CHEVROCHES|        : ...........A........C.........................................A.....A..G..T.................A....... :  100
KT247595.1|PUUV|N|France|M.g.|45|Orleans/Mg29/2010 : ...........A.....T..C.......T.................................A.....A..G..............G...........A. :  100
KT247594.1|PUUV|N|France|M.g.|45|Orleans/Mg23/2010 : ...........A.....T..C.......T.................................A.....A..G..............G...........A. :  100
KY365004.1|PUUV|N|France|M.g.|45|Orleans/NCHA373/2 : ...........A.....T..C..A.......................................A.....A..G...........................A. :  100
2012.00301|PUUV|N|France|H.s.|25|MOUTHE|           : ...g................A..A....................................................................A....... :  100
AM695638.1|PUUV|N|France|M.g.|Mignovillard/CgY02/2 : ...G................A..A....................................................................A....... :  100
KT247597.1|PUUV|N|France|M.g.|39|Jura/Mg214/2010|  : .......................A............................................................................ :  100
KT247596.2|PUUV|N|France|M.g.|39|Jura/Mg2/2010|    : .......................A............................................................................ :  100
2012.00102|PUUV|N|France|H.s.|39|COISERETTE|       : .......................A............................................................................ :  100
2012.00536|PUUV|N|France|H.s.|39|LA-PESSE|         : .......................A............................................................................ :  100
2014.00622|PUUV|N|France|H.s.|39|ARBOIS|           : ....................A..A............................................................................ :  100
2015.00567|PUUV|N|France|H.s.|70|RIOZ|             : .......................A............................................................................ :  100
2014.00637|PUUV|N|France|H.s.|25|SAULES|           : .......................A............................................................................ :  100
2012.00396|PUUV|N|France|H.s.|39|SAINT-CLAUDE|     : ....................A..A............................................................................ :  100
2014.00120|PUUV|N|France|H.s.|38|LE-MOUTARET|      : ....................A..A..................................................T......................... :  100
2015.00153|PUUV|N|France|H.s.|73|GREZY-SUR-ISERE|  : ....................A..A..................................................T......................... :  100
2015.00504|PUUV|N|France|H.s.|70|RONCHAMP|         : ..............G........A............................................................................ :  100
2016.00275|PUUV|N|France|H.s.|21|JALLANGES|        : .................T.....A............................................................................ :  100
2016.00320|PUUV|N|France|H.s.|25|SAINT-VIT|        : .......................A............................................................................ :  100
2015.00410|PUUV|N|France|H.s.|70|ANGIREY|          : ..............G........A............................................................................ :  100
DQ016430.2|PUUV|N|Germany|M.g.|Bavaria/CG33/04|    : ....................................................................A..G...........................A. :  100
DQ016432.2|PUUV|N|Germany|M.g.|Bavaria/CG41/04|    : ....................................................................A..G...........................A. :  100
AY954723.2|PUUV|N|Germany|M.g.|Bavaria-CG34/04|    : ....................................................................A..G.............G.............A. :  100
AY954722.2|PUUV|N|Germany|M.g.|Bavaria-CG9/04|     : .................T..................................................A..G...........................A. :  100
2012.00402|PUUV|N|France|H.s.|60|GOUVIEUX|         : ........T........T..................................................A............................... :  100
2014.00540|PUUV|N|France|H.s.|60|CHAMBLY|          : .................T..................................................A............................... :  100
AJ314600.1|PUUV|N|Balkan|M.g.|Balkan-1|            : .................T..C..A.......................G......................................G.....A.....A. :  100
AJ314601.1|PUUV|N|Balkan|M.g.|Balkan-2|            : .................T..C..A.......................G......................................G.....A.....A. :  100
FN377821.1|PUUV|N|Hungary|M.g.|Mg9/HungaryTR17/00| : ............G....T..C..A.......................G..........................T...........G.....A.....A. :  100
FN377822.1|PUUV|N|Hungary|M.g.|Mg23/HungaryTR17/00 : .................T..C..A.......................G......................................G.....A.....A. :  100
GQ339473.1|PUUV|N|Sweden|M.g.|Kiviniemi/Mg3/05|    : .................T.....A...........T..............G.................A.....T.................A....... :  100
GQ339474.1|PUUV|N|Sweden|M.g.|Kiviniemi/Mg5/05|    : .................T.....A...........T..............G.................A.....T.................A.....A. :  100
GQ339475.1|PUUV|N|Sweden|M.g.|Kiviniemi/Mg6/05|    : .................T.....A...........T..............G.................A.....T.................A.....A. :  100
GQ339476.1|PUUV|N|Sweden|M.g.|Aijajarvi/Mg7/05|    : .................T.....A...........T..............G.................A.....T.................A....... :  100
```

```
                                                                       Kramski M et al. Clinical Chemistry 2007  PUUV F  GARR
Garin D et al Microb. Infect 2001  PUF1 GAAAAAAACTGGGATGAGTGACTTG        CRRSAp   ATAACCCGCCATGAACAACAGCTTG ACAACGGTCTGTTTTCGAGTT  PUR1

                                                        *        20         *        40         *        60         *        80         *       100
2014.00598|PUUV|N|France|H.s.|59|FOURMIES|        : ATGAGTGACTTGACAGACATTCAGGAGGATATAACCCGCCATGAACAACAACTTGTTGTTGCCAGACAGAAACTCAAGGATGCAGAAAAGGCGGTGGAGA :  100
GQ339477.1|PUUV|N|Sweden|M.g.|Aijajarvi/Mg9/05|   : .................T.....A...........T..............G.................A.....T.................A....... :  100
GQ339478.1|PUUV|N|Sweden|M.g.|Jockfall/Mg12/05|   : .................T.....A...........T..............G.................A.....T.................A....... :  100
GQ339482.1|PUUV|N|Sweden|M.g.|Kalvudden/Mg22/05|  : .................T.....A..A........T..............G.................A.....T.................A....... :  100
GQ339479.1|PUUV|N|Sweden|M.g.|Moskosel/Mg17/05|   : .................T..C..A..........................G.......................T..A..............A....... :  100
GQ339481.1|PUUV|N|Sweden|M.g.|Ljustrask/Mg20/05|  : .................T..C..A..........................G...........T.....A.....T..A..............A....... :  100
GQ339480.1|PUUV|N|Sweden|M.g.|Gyttjea/Mg19/05|    : .................T..C..A..........................G.................A.....T..A..............A....... :  100
AY526219.1|PUUV|N|Sweden|Human|Umea/hu|           : .................T..C..A..........................G.................A.....T...........G.....A.....A. :  100
AJ223380.1|PUUV|N|Sweden|M.g.|Tavelsjo/Cg81/94|   : ................CT..C..A........T.................G..............G..A.....T...........G.....A.....A. :  100
U14137.1|PUUV|N|Bosnia-H.|M.g.|Vranica|           : ........TC.......T..C..A..........................G..............G..A.....T...........G.....A.....A. :  100
AJ223371.1|PUUV|N|Sweden|M.g.|Huggberget/Cg36/94| : .................T.....A........T.................G..............G..A.....T...........G.....A.....A. :  100
Z48586.1|PUUV|N|Sweden|M.g.|Vindeln/L20Cg/83|     : .................T..C..A..........................G.................A.....T...........G.....A.....A. :  100
AJ223374.1|PUUV|N|Sweden|M.g.|Mellansel/Cg47/94|  : .................T..C..A..........................G.......................T...........G.....A.....A. :  100
AJ223375.1|PUUV|N|Sweden|M.g.|Mellansel/Cg49/94|  : .................T..C..A..........................G.......................T...........G.....A.....A. :  100
AJ238791.1|PUUV|N|Denmark|M.g.|Fyn/19|            : ...........A........C................................G..............A.....T..............A..A.....A. :  100
AJ278092.1|PUUV|N|Denmark|M.g.|Fyn47|             : ....................C..A.............................G..............A.....T...........G..A..A.....A. :  100
AJ278093.1|PUUV|N|Denmark|M.g.|Fyn131|            : ....................C..A.............................G..............A.....T...........G..A..A.....A. :  100
AJ223368.1|PUUV|N|Norway|M.g.|Eidsvoll/1124v|     : .................T..C..A..C..G.................G..........C..................A........G.G...A.....A. :  100
AJ223369.1|PUUV|N|Norway|M.g.|Eidsvoll/Cg1138/87| : .................T..C..A..C..G.................G..........C..................A........G.G...A.....A. :  100
JN657228.1|PUUV|N|Latvia|M.g.|Jelgava/Mg149/2008| : ...........A...........A..........................G...........T.....A.....T..A...........A..A.....A. :  100
KX757839.1|PUUV|N|Lithuania|M.g.|LT15/164|2015|   : ...........A.....T..C..A...........T..............G.................A.....T..A...........A..A....... :  100
KX757840.1|PUUV|N|Lithuania|M.g.|LT15/174|2015|   : ...........A.....T..C..A...........T..............G.................A.....T..A...........A..A....... :  100
KX815394.1|PUUV|N|Poland|M.g.|KS13/855|2009|      : .................T..C..A..........................G.................A.....T..A...........A..A.....A. :  100
KX757841.1|PUUV|N|Lithuania|M.g.|LT15/201|2015|   : ...........A.....T..C..A...........T..............G.................A.....T..A...........A..A....... :  100
AJ314598.1|PUUV|N|Russia|M.g.|Baltic/49Cg/00|     : ............G....T..C..A....................G.........................................G.GA..A......G :  100
AJ314599.1|PUUV|N|Russia|M.g.|Baltic/205Cg/00|    : ............G....T..A..A....................G..G......................................G.GA..A......G :  100
JN657229.1|PUUV|N|Latvia|M.g.|Madona/Mg99/2008|   : ............G....T..C..A....................G..G..............A........G..T...........G.GA..A.....AG :  100
JN657232.1|PUUV|N|Latvia|M.g.|Madona/Mg233/2008|  : ............G....T..C..A....................G..G..............A........G..T...........G.GA..A.....AG :  100
JN657230.1|PUUV|N|Latvia|M.g.|Jelgava/Mg136/2008| : ............G....T..C..A....................G..G...................................G..G.GA..A.....AG :  100
JN657231.1|PUUV|N|Latvia|M.g.|Jelgava/Mg140/2008| : ............G....T..C..A....................G..G......................................G.GA..A.....AG :  100
Z30707.1|PUUV|N|Russia|M.g.|Udmurtia/458Cg/88|    : ....................C..A.....G..............G.......................A.................G.GA..A.....AG :  100
Z30706.1|PUUV|N|Russia|M.g.|Udmurtia/444Cg/88|    : ....................C..A.....G..............G.......................A.................G.GA..A.....AG :  100
Z84204.1|PUUV|N|Russia|M.g.|Kazan|                : ....................C..A.....G..............G.......................A.................G.GA..A.....AG :  100
Z30708.1|PUUV|N|Russia|M.g.|Udmurtia/338Cg/92|    : ....................C..A.....G..............G.......................A.................G.GA..A.....AG :  100
Z21497.1|PUUV|N|Russia|M.g.|Udmurtia/894Cg/91|    : ....................C..A.....G..............G.......................A.................G.GA..A.....AG :  100
AB433843.2|PUUV|N|Russia|M.g.|Samara_49/CG/2005|  : ....................C..A.....G..............G.......................A.................G.GA..A.....AG :  100
AB433845.2|PUUV|N|Russia|M.g.|Samara_94/CG/2005|  : ....................C..A.....G..............G.......................A.................G.GA..A.....AG :  100
L11347.1|PUUV|N|Russia|Human|P360|                : ....................C..A.....G..............G.......................A.................G.GA..A.....AG :  100
AB297665.2|PUUV|N|Russia|M.g.|DTK/Ufa-97|1997|    : ....................C..A.....G..............G.......................A.................G.GA..A.....AG :  100
M32750.1|PUUV|N|Russia|M.g.|CG1820|               : ....................C..A.....G..............G.......................A.................G.GA..A.....AG :  100
AF442613.1|PUUV|N|Russia|M.g.|CG17/Baskiria-2001| : ............G.......C..A.....G..............G.............C.........A.................G.GA..A.....AG :  100
KX815395.1|PUUV|N|Poland|M.g.|KS14/118|2009|      : ...........A.....T..C..A....................G..G....................A.................G.GA..A.....AG :  100
GQ339483.1|PUUV|N|Sweden|M.g.|Bergsjobo/Mg25/05|  : ............G....T..C.....C..G.................G..........C.........A.................G.GA..A......G :  100
AJ223377.1|PUUV|N|Sweden|M.g.|Solleftea/Cg6/95|   : ............G....T..C.....C..G.................G..........C.........A.................G.GA..A......G :  100
GQ339484.1|PUUV|N|Sweden|M.g.|Faboviken/Mg26/05|  : ............G....T..C.....C..G.................G..........C.........A.................G.GA..A....... :  100
GQ339485.1|PUUV|N|Sweden|M.g.|Mangelbo/Mg1/05|    : .................T..C.....C..A.................G.....G....C.........A.................G.G...A.....A. :  100
GQ339486.1|PUUV|N|Sweden|M.g.|Munga/Mg2/05|       : .................T..C.....C..A.................G.....G....C.........A.................G.G...A.....A. :  100
GQ339487.1|PUUV|N|Sweden|M.g.|Munga/Mg16/05|      : .................T..C.....C..A.................G.....G....C.........A.................G.G...A.....A. :  100
AJ223376.1|PUUV|N|Sweden|M.g.|Solleftea/Cg3/95|   : .................T..C.....C..G.................G..........C.........A.................G.GA..A....... :  100
AF367071.1|PUUV|N|Russia|M.r.|CRF366|             : ....C............T.....A..............T...........G.......C.........A.....T..A..........GA..A....... :  100
AF367064.1|PUUV|N|Russia|M.g.|CG144|              : .................T.....A..............T...........G.......C.........A.....T..A..........GA..A....... :  100
AF367065.1|PUUV|N|Russia|M.g.|CG168|              : .................T.....A..............T...........G.......C.........A.....T..A..........GA..A....... :  100
AF367068.1|PUUV|N|Russia|M.g.|CG315|              : .................T.....A..............T...........G.......C.........A.....T..A..........GA..A....... :  100
AF367069.1|PUUV|N|Russia|M.r.|CRF161|             : .................T.....A..............T...........G.......C.........A.....T..A..........GA..A....... :  100
```

```
                                                                                              Kramski M et al. Clinical Chemistry 2007  PUUV F  GARR
Garin D et al Microb. Infect 2001  PUF1 GAAAAAAACTGGGATGAGTGACTTG        CRRSAp   ATAACCCGCCATGAACAACAGCTTG ACAACGGTCTGTTTTCGAGTT  PUR1

                                                              *        20         *        40         *        60         *        80         *       100
2014.00598|PUUV|N|France|H.s.|59|FOURMIES|        : ATGAGTGACTTGACAGACATTCAGGAGGATATAACCCGCCATGAACAACAACTTGTTGTTGCCAGACAGAAACTCAAGGATGCAGAAAAGGCGGTGGAGA :  100
AF367070.1|PUUV|N|Russia|M.r.|CRF308|             : .................T.....A..............T...........G.......C.........A.....T..A..........GA..A....... :  100
AF367066.1|PUUV|N|Russia|M.g.|CG215|              : .................T.....A..............T...........G.......C.........A.....T..A..........GA..A....... :  100
AF367067.1|PUUV|N|Russia|M.g.|CG222|              : .................T.....A..............T...........G.......C.........A.....T..A..........GA..A....... :  100
Z46942.1|PUUV|N|Finland|M.g.|Puumala/1324Cg/79|   : .................T..C..A.......................G..G...........T.....A.....T..A..........GA..A.....AG :  100
Z30702.1|PUUV|N|Finland|M.g.|Evo/12Cg/93|         : .................T..C..A.......................G..G.................A.....T...........G.GA..A.....AG :  100
Z30703.1|PUUV|N|Finland|M.g.|Evo/13Cg/93|         : .................T..C..A.......................G..G.................A.....T...........G.GA..A.....AG :  100
Z30704.1|PUUV|N|Finland|M.g.|Evo/14Cg/93|         : .................T..C..A.......................G..G.................A.....T...........G.GA..A.....AG :  100
Z30705.1|PUUV|N|Finland|M.g.|Evo/15Cg/93|         : .................T..C..A.......................G..G.................AGG...T...........G.GA..A.....AG :  100
Z69985.1|PUUV|N|Finland|M.g.|Virrat/25Cg/95|      : .................T..C..A..........................G.................A.....T...........G.GA..A.....AG :  100
JN831947.1|PUUV|N|Finland|Pieksamaki/human_lung/20 : .................T..C..A.......................G..G.................A.....T..A........G.GA..A.....AG :  100
JN831943.1|PUUV|N|Finland|Pieksamaki/Mg7/2008|    : .................T..C..A.......................G..G.................A.....T..A........G.GA..A.....AG :  100
JN831950.1|PUUV|N|Finland|Pieksamaki/human_kidney/ : .................T..C..A.......................G..G.................A.....T..A........G.GA..A.....AG :  100
JQ319166.1|PUUV|N|Finland|Konnevesi/Mg_014B/2005| : .................T..C..A.......................G..G.................A.....T..A........G.GA..A.....AG :  100
JQ319169.1|PUUV|N|Finland|Konnevesi/Mg_027B/2005| : .................T..C..A.......................G..G.................A.....T..A........G.GA..A.....AG :  100
JQ319167.1|PUUV|N|Finland|Konnevesi/Mg_015B/2005| : .................T..C..A..............T........G..G.................A.....T..A........G.GA..A.....AG :  100
JQ319164.1|PUUV|N|Finland|Konnevesi/Mg_06B/2005|  : .................T..C..A.......................G..G.................A.....T..A........G.GA..A.....AG :  100
JQ319165.1|PUUV|N|Finland|Konnevesi/Mg_09B/2005|  : .................T..C..A.......................G..G.................A.....T..A........G.GA..A.....AG :  100
JQ319170.1|PUUV|N|Finland|Konnevesi/Mg_074B/2005| : .................T..C..A.......................G..G.................A.....T..A........G.GA..A.....AG :  100
JQ319171.1|PUUV|N|Finland|Konnevesi/Mg_M114B/2005| : .................T..C..A..............T........G..G.................A.....T..A........G.GA..A.....AG :  100
AJ238788.1|PUUV|N|Russia|M.g.|Karhumaki|          : .................T..C..A.......................G..G.................A.....T.............GA..A.....AG :  100
AJ238789.1|PUUV|N|Russia|M.g.|Kolodozero|         : .................T..C..A.......................G....................A.....T..A........G.GA..A.....AG :  100
AJ314597.1|PUUV|N|Finland|M.g.|Pallasjarvi/63Cg/98 : .................T..C..A.......................G..G.................A.....T...........G.GA..A.....AG :  100
NC_005224.1|PUUV|N|Finland|M.g.|Sotkamo-revu-NCBI2 : .................T..C..A.......................G......A.............A.....T...........G.GA..A.....AG :  100
AJ238790.1|PUUV|N|Russia|M.g.|Gomselga|           : .................T..C..A.......................G..G.................A.....T.............GA..A.....AG :  100
AB010731.1|Hokkaido-V|N|Japan|M.r.|Tobetsu-60Cr-93 : .............A...T..C..........................................C.G..A..G..T.GA........G..A..A.....A. :  100
AB010730.1|Hokkaido-V|N|Japan|M.r.|Kamiiso-8Cr-95| : .............A...T..C..........................................C.G..A..G..T.GA........G..A..A.....A. :  100
JX046487.1|Muju-V|N|South-Korea|M.r.|11-5|2011    : ...........A.AG..A..A..A..A......G......................G.....A..G..A..G....G...............A.....AG :  100
JX046484.1|Muju-V|N|South-Korea|M.r.|11-4|2011|   : ...........A.AG..A..A..A..A......G......................G.....A..G..A..G....G...............A.....AG :  100
JX028273.1|Muju-V|N|South-Korea|M.r.|11-1|2011|   : ...........A.AG..A..A..A..A......G......................G.....A..G..A..G....G...............A.....AG :  100
DQ138128.1|Muju-V|N|South-Korea|E.r.|00-18|       : ...........A.AG..A..A..A..A..C...G.............G........G..G..A..G..A.......G.........G.....A......G :  100
DQ138140.1|Muju-V|N|South-Korea|E.r.|99-27|       : ...........A.AG..A..A..A..A..C...G.............G........G..G..A..G..A.......G.........G.....A......G :  100
DQ138133.1|Muju-V|N|South-Korea|E.r.|96-1|        : ...........A.AG..A..A..A..A..C...G.............G........G..G..A..G..A.......G.........G.....A......G :  100
DQ138142.1|Muju-V|N|South-Korea|E.r.|99-28|       : ...........A.AG..A..A..A..A..C...G.............G........G..G..A..G..A.......G.........G.....A......G :  100
```

```
  Lagerqvist N et al. J Clin Microbiol 2016  PUUV_F  TGGACCCRGATGACGTTAACPUUV_PACACTGCAAGCAAGYGTTGTYTGTCACAGTCGTCAC  PUUV_R
Kramski M et al. Clin Chemistry 2007   (end PUUV F)  TGGACCCRGATGACGTTAA           PUUVTMGB  CAACARACAGTGTCAGCA                (end of PUUV R)  TACCGTCT

                                                                                                      Evander M et al. J Clin Microbiol  2007  S237 5' GGCAGA
                                                                *        120         *        140         *        160         *        180         *        200
2014.00598|PUUV|N|France|H.s.|59|FOURMIES|        : TGGACCCAGATGACGTTAACAAAAACACATTGCAAGCAAGGCAACAGACAGTGTCAGCACTGGAGGACAAACTTGCAGACTTCAAGCGACAAATGGCAGA :  200
2014.00233|PUUV|N|France|H.s.|59|FOURMIES|        : .................................................................................................... :  200
2014.00097|PUUV|N|France|H.s.|02|SAINT-MICHEL|    : .................................................................................................... :  200
2014.00488|PUUV|N|France|H.s.|08|SIGNY-LE-PETIT|  : .................................................................................................... :  200
2014.00613|PUUV|N|France|H.s.|59|FOURMIES|        : .................................................................................................... :  200
2015.00402|PUUV|N|France|H.s.|08|CHARLEVILLE-MEZIE : ...........................................................................................G........ :  200
2015.00422|PUUV|N|France|H.s.|59|ANOR|            : .................................................................................................... :  200
2015.00488|PUUV|N|France|H.s.|08|SEDAN|           : ...........................................................................................G........ :  200
2015.00498|PUUV|N|France|H.s.|08|REMILLY-AILLICOUR : .................................................................................................... :  200
2016.00295|PUUV|N|France|H.s.|08|CORNY-MACHEROMENI : ...................................................................T................................ :  200
2016.00333|PUUV|N|France|H.s.|59|FOURMIES|        : .................................................................................................... :  200
2016.00345|PUUV|N|France|H.s.|59|FOURMIES|        : .................................................................................................... :  200
2016.00357|PUUV|N|France|H.s.|02|HIRSON|          : .................................................................................................... :  200
2016.00427|PUUV|N|France|H.s.|59|FOURMIES|        : .................................................................................................... :  200
2016.00469|PUUV|N|France|H.s.|02|PREMONTRE|       : .......G.....T...............C...................................................................... :  200
AJ277075.1|PUUV|N|Belgium|M.g.|Montbliart/CG14444| : .................................................................................................... :  200
2014.00053|PUUV|N|France|H.s.|08|SECHEVAL|        : ...........................................................................................G........ :  200
KT247593.1|PUUV|N|France|M.g.|08|Ardennes/Mg75/201 : .............................C.............................................................G........ :  200
KT247592.1|PUUV|N|France|M.g.|08|Ardennes/Mg156/20 : .............................C.............................................................G........ :  200
2015.00019|PUUV|N|France|H.s.|08|ETEIGNIERES|     : ...........................................................................................G........ :  200
2016.00239|PUUV|N|France|H.s.|08|VIREUX-MOLHAIN|  : ...........................................................................................G........ :  200
2015.00329|PUUV|N|France|H.s.|08|BOGNY-SUR-MEUSE| : ...........................................................................................G........ :  200
2014.00209|PUUV|N|France|H.s.|08|TREMBLOIS-LES-ROC : ...........................................................................................G........ :  200
2015.00456|PUUV|N|France|H.s.|94|ALFORTVILLE|     : ...........................................................................................G........ :  200
2012.00018|PUUV|N|France|H.s.|08|ROCROI|          : ......................................................................G....................G........ :  200
AJ277032.1|PUUV|N|Belgium|M.g.|Momignies/47Cg/96| : ...........................................................................................G........ :  200
AJ277034.1|PUUV|N|Belgium|M.g.|Couvin/59Cg/97|    : ...........................................................................................G........ :  200
AJ277033.1|PUUV|N|Belgium|M.g.|Momignies/55Cg/96| : ...........................................................................................G........ :  200
2012.00638|PUUV|N|France|H.s.|08|GIVET|           : ...........................................................................................G........ :  200
2015.00328|PUUV|N|France|H.s.|08|VRIGNE-MEUSE|    : .................................................................................................... :  200
2015.00419|PUUV|N|France|H.s.|08|DOUZY|           : .................................................................................................... :  200
2016.00310|PUUV|N|France|H.s.|08|THIN-LE-MOUTIER| : .................................................................................................... :  200
AJ277030.1|PUUV|N|Belgium|M.g.|Thuin/33Cg/96|     : .................................................................................................... :  200
AJ238779.1|PUUV|N|Germany|M.g.|NRW/Cg-Erft|       : ......................G............................................................................. :  200
2014.00276|PUUV|N|France|H.s.|59|LILLE|           : .................................................................................................... :  200
U22423.1|PUUV|N|Belgium|M.g.|CG13891|             : ......................G.........................................A................................... :  200
2014.00321|PUUV|N|France|H.s.|59|FOURMIES|        : .................................................................................................... :  200
2015.00457|PUUV|N|France|H.s.|55|REVIGNY-SUR-ORNAI : .......G.....T...................................................................................... :  200
2013.00250|PUUV|N|France|H.s.|62|VENDIN-LS-BTHUNE| : .................................................................................................... :  200
2016.00282|PUUV|N|France|H.s.|59|MORBECQUE|       : .....................................G.............................................................. :  200
AJ277076.1|PUUV|N|Belgium|M.g.|Montbliart/CG14445| : .................................................................................................... :  200
2014.00153|PUUV|N|France|H.s.|02|BUIRONFOSSE|     : ........................G........................................................................... :  200
2014.00171|PUUV|N|France|H.s.|59|SAINT-SAULVE|    : .............................C.A..............T..........................C.......................... :  200
2016.00293|PUUV|N|France|H.s.|60|LA-NEUVILLE-SUR-R : ..............................................T....................T................................ :  200
2014.00499|PUUV|N|France|H.s.|94|CHAMPIGNY-SUR-MAR : ...................................................................T................................ :  200
2012.00057|PUUV|N|France|H.s.|59|COUSOLRE|        : .................................................................................................... :  200
2012.00349|PUUV|N|France|H.s.|02|ENGLANCOURT|     : .................................................................................................... :  200
2014.00135|PUUV|N|France|H.s.|02|ATHIES-SOUS-LAON| : .................................................................................................... :  200
2014.00184|PUUV|N|France|H.s.|59|FOURMIES|        : .........................T.......................................................................... :  200
2016.00182|PUUV|N|France|H.s.|08|BOULZICOURT|     : .................................................................................................... :  200
2016.00286|PUUV|N|France|H.s.|02|TRUCY|           : .................................................................................................... :  200
2016.00325|PUUV|N|France|H.s.|59|AULNOYE-AYMERIES| : .................................................................................................... :  200
2016.00452|PUUV|N|France|H.s.|02|LAON|            : .................................................................................................... :  200
2016.00467|PUUV|N|France|H.s.|02|AISNE|           : .................................................................................................... :  200
```

```
  Lagerqvist N et al. J Clin Microbiol 2016  PUUV_F  TGGACCCRGATGACGTTAACPUUV_PACACTGCAAGCAAGYGTTGTYTGTCACAGTCGTCAC  PUUV_R
Kramski M et al. Clin Chemistry 2007   (end PUUV F)  TGGACCCRGATGACGTTAA            PUUVTMGB  CAACARACAGTGTCAGCA                (end of PUUV R)  TACCGTCT

                                                                                                Evander M et al. J Clin Microbiol  2007  S237 5' GGCAGA
                                                              *         120         *         140         *         160         *         180         *         200
2014.00598|PUUV|N|France|H.s.|59|FOURMIES|         : TGGACCCAGATGACGTTAACAAAAACACATTGCAAGCAAGGCAACAGACAGTGTCAGCACTGGAGGACAAACTTGCAGACTTCAAGCGACAAATGGCAGA :  200
2015.00657|PUUV|N|France|H.s.|02|CILLY|            : .................................................................................................... :  200
2015.00526|PUUV|N|France|H.s.|08|MONTHERME|        : .................................................................................................... :  200
2015.00045|PUUV|N|France|H.s.|59|FOURMIES|         : .........................T.......................................................................... :  200
2015.00430|PUUV|N|France|H.s.|02|MONTCORNET|       : .................................................................................................... :  200
2016.00311|PUUV|N|France|H.s.|02|LAON|             : .................................................................................................... :  200
AJ277031.1|PUUV|N|Belgium|M.g.|Montbliart/23Cg/96| : .................................................................................................... :  200
2016.00326|PUUV|N|France|H.s.|02|LAON|             : .................................................................................................... :  200
2016.00353|PUUV|N|France|H.s.|02|CESSIERES|        : .................................................................................................... :  200
2012.00061|PUUV|N|France|H.s.|02|LANISCOURT|       : .................................................................................................... :  200
2014.00174|PUUV|N|France|H.s.|02|ST-ERME-OUTRE-ET- : .................................................................................................... :  200
2016.00268|PUUV|N|France|H.s.|02|PRESLES-ET-THIERN : .................................................................................................... :  200
2015.00660|PUUV|N|France|H.s.|02|VIC-SUR-AISNE|    : ...........................................................T...............................G.....C.. :  200
2012.00025|PUUV|N|France|H.s.|51|SAINTE-MENEHOULD| : .............................C.T..............A............T..........G....................G........ :  200
2015.00665|PUUV|N|France|H.s.|51|REIMS|            : .............................C.T..............A............T..........G....................G........ :  200
2012.00307|PUUV|N|France|H.s.|54|COLOMBEY-LES-BELL : .............................C.T............................................G..............G........ :  200
2012.00123|PUUV|N|France|H.s.|55|BAR-LE-DUC|       : .............................C.T..............A............T..........G....................G........ :  200
2012.00278|PUUV|N|France|H.s.|51|REIMS|            : .............................C.T..............A............T...............................G........ :  200
2015.00185|PUUV|N|France|H.s.|68|FELLERING|        : .............................C.T..............A............T...............................G........ :  200
KJ994776.1|PUUV|N|Germany|M.g.|Mu/07/1219|2007|    : ......................G..T...........C........A............T....A................A.......AG......... :  200
2012.00086|PUUV|N|France|H.s|58|CHEVROCHES|        : .............................C.T...........................T.............................AG......... :  200
KT247595.1|PUUV|N|France|M.g.|45|Orleans/Mg29/2010 : .............................C.T...........................T.............................AG......... :  200
KT247594.1|PUUV|N|France|M.g.|45|Orleans/Mg23/2010 : .............................C.T...........................T.............................AG......... :  200
KY365004.1|PUUV|N|France|M.g.|45|Orleans/NCHA373/2 : .............................C.T...........................T.......T..................A..AG......... :  200
2012.00301|PUUV|N|France|H.s.|25|MOUTHE|           : .............................C.............................T....A.....................A..AG......... :  200
AM695638.1|PUUV|N|France|M.g.|Mignovillard/CgY02/2 : .............................C.............................T....A.....................A..AG......... :  200
KT247597.1|PUUV|N|France|M.g.|39|Jura/Mg214/2010|  : .............................C.C...........................T....A.....................A..AG......... :  200
KT247596.2|PUUV|N|France|M.g.|39|Jura/Mg2/2010|    : .............................C.............................T....A.....................A..AG......... :  200
2012.00102|PUUV|N|France|H.s.|39|COISERETTE|       : .......G.....................C..................................A.....................A..AG......... :  200
2012.00536|PUUV|N|France|H.s.|39|LA-PESSE|         : .......G.....................C..................................A.....................A..AG......... :  200
2014.00622|PUUV|N|France|H.s.|39|ARBOIS|           : .............................C.............................T....A.....................A..AG......... :  200
2015.00567|PUUV|N|France|H.s.|70|RIOZ|             : .............................C.............................T....A.....................A..AG......... :  200
2014.00637|PUUV|N|France|H.s.|25|SAULES|           : .............................C.............................T....A.....................A..AG......... :  200
2012.00396|PUUV|N|France|H.s.|39|SAINT-CLAUDE|     : .............................C.............................T....A.....................A..AG......... :  200
2014.00120|PUUV|N|France|H.s.|38|LE-MOUTARET|      : .............................C.............................T....A.....................A..AG......... :  200
2015.00153|PUUV|N|France|H.s.|73|GREZY-SUR-ISERE|  : .............................C.............................T....A.....................A..AG......... :  200
2015.00504|PUUV|N|France|H.s.|70|RONCHAMP|         : .......G.................T...C.............................T....A....................AA..AG......... :  200
2016.00275|PUUV|N|France|H.s.|21|JALLANGES|        : .............................C.............................T....A.....................A..AG......... :  200
2016.00320|PUUV|N|France|H.s.|25|SAINT-VIT|        : .............................C.............................T....A.....................A..AG......... :  200
2015.00410|PUUV|N|France|H.s.|70|ANGIREY|          : .........................T...C.............................T....A................A...AA..AG......... :  200
DQ016430.2|PUUV|N|Germany|M.g.|Bavaria/CG33/04|    : ......................G..T.....A..............A.................A..T.....C.....T.A.......AG......... :  200
DQ016432.2|PUUV|N|Germany|M.g.|Bavaria/CG41/04|    : ......................G........A..............A.................A.....T..C.....T.A....A..AG......... :  200
AY954723.2|PUUV|N|Germany|M.g.|Bavaria-CG34/04|    : ...................T..G........A..............A.................A........C.....T.A....A..AG......... :  200
AY954722.2|PUUV|N|Germany|M.g.|Bavaria-CG9/04|     : ......................G........A..............A.................A........C.....T.A.......AG......... :  200
2012.00402|PUUV|N|France|H.s.|60|GOUVIEUX|         : .........................T...C.............................T....A.....................A..AG......... :  200
2014.00540|PUUV|N|France|H.s.|60|CHAMBLY|          : .........................T...C.............................T....A.....................A..AG......... :  200
AJ314600.1|PUUV|N|Balkan|M.g.|Balkan-1|            : .........................T...C.............................T.............C........T......AG......... :  200
AJ314601.1|PUUV|N|Balkan|M.g.|Balkan-2|            : .........................T...C................A............T.............C........T......AG......... :  200
FN377821.1|PUUV|N|Hungary|M.g.|Mg9/HungaryTR17/00| : .........................T...C................A............T.............C........T......AG......... :  200
FN377822.1|PUUV|N|Hungary|M.g.|Mg23/HungaryTR17/00 : .........................T...C................A............T.............C........T.....GAG......... :  200
GQ339473.1|PUUV|N|Sweden|M.g.|Kiviniemi/Mg3/05|    : .......G.....................C................A....................T.....C..G..............G........ :  200
GQ339474.1|PUUV|N|Sweden|M.g.|Kiviniemi/Mg5/05|    : .......G.....................C................A....................T.....C.................G........ :  200
GQ339475.1|PUUV|N|Sweden|M.g.|Kiviniemi/Mg6/05|    : .......G.....................C................A....................T.....C.................G........ :  200
GQ339476.1|PUUV|N|Sweden|M.g.|Aijajarvi/Mg7/05|    : .......G.....................C................A....................T.....C.................G........ :  200
```

```
  Lagerqvist N et al. J Clin Microbiol 2016  PUUV_F  TGGACCCRGATGACGTTAACPUUV_PACACTGCAAGCAAGYGTTGTYTGTCACAGTCGTCAC  PUUV_R
Kramski M et al. Clin Chemistry 2007   (end PUUV F)  TGGACCCRGATGACGTTAA            PUUVTMGB  CAACARACAGTGTCAGCA                 (end of PUUV R)  TACCGTCT

                                                                                                      Evander M et al. J Clin Microbiol  2007  S237 5' GGCAGA
                                                              *        120         *        140         *        160         *        180         *        200
2014.00598|PUUV|N|France|H.s.|59|FOURMIES|       : TGGACCCAGATGACGTTAACAAAAACACATTGCAAGCAAGGCAACAGACAGTGTCAGCACTGGAGGACAAACTTGCAGACTTCAAGCGACAAATGGCAGA :  200
GQ339477.1|PUUV|N|Sweden|M.g.|Aijajarvi/Mg9/05|  : .......G.....................C................A....................T.....C..G..............G........ :  200
GQ339478.1|PUUV|N|Sweden|M.g.|Jockfall/Mg12/05|  : .......G.....................C................A....................T.....C.................G........ :  200
GQ339482.1|PUUV|N|Sweden|M.g.|Kalvudden/Mg22/05| : .......G.....................C..................................A..T.......................G........ :  200
GQ339479.1|PUUV|N|Sweden|M.g.|Moskosel/Mg17/05|  : .............................C...........................................C.................G........ :  200
GQ339481.1|PUUV|N|Sweden|M.g.|Ljustrask/Mg20/05| : .......G.....................C...........................................C.................G........ :  200
GQ339480.1|PUUV|N|Sweden|M.g.|Gyttjea/Mg19/05|   : .......G.....................C.....................................T.....C.................G........ :  200
AY526219.1|PUUV|N|Sweden|Human|Umea/hu|          : .......G.....................C.....................................T.....C................G......... :  200
AJ223380.1|PUUV|N|Sweden|M.g.|Tavelsjo/Cg81/94|  : .......G.....................C.....................................T.....................AG......... :  200
U14137.1|PUUV|N|Bosnia-H.|M.g.|Vranica|          : ..T....G.....................C.....................................T.....C...............AG......... :  200
AJ223371.1|PUUV|N|Sweden|M.g.|Huggberget/Cg36/94|: .......G.....................C..................................A..T.....C...............AG......... :  200
Z48586.1|PUUV|N|Sweden|M.g.|Vindeln/L20Cg/83|    : .......G.....................C................A............T.......T.....C.......A........G......... :  200
AJ223374.1|PUUV|N|Sweden|M.g.|Mellansel/Cg47/94| : .......G.....................C.....................................T.....C................G......... :  200
AJ223375.1|PUUV|N|Sweden|M.g.|Mellansel/Cg49/94| : .......G.....................C.....................................T.....C................G......... :  200
AJ238791.1|PUUV|N|Denmark|M.g.|Fyn/19|           : ...................T.........C.....................................T........G.........A..AG......... :  200
AJ278092.1|PUUV|N|Denmark|M.g.|Fyn47|            : ...................T.........C.....................................T.....C.....T......A..AG......... :  200
AJ278093.1|PUUV|N|Denmark|M.g.|Fyn131|           : ...................T.........C..G..................................T.....C.....T......A..AG....A.... :  200
AJ223368.1|PUUV|N|Norway|M.g.|Eidsvoll/1124v|    : .............................C.....................................T.................A..GA.......... :  200
AJ223369.1|PUUV|N|Norway|M.g.|Eidsvoll/Cg1138/87|: .............................C.....................................T.................A..GA.......... :  200
JN657228.1|PUUV|N|Latvia|M.g.|Jelgava/Mg149/2008|: .......G..............G......C.............................T.......T..................A..AG......... :  200
KX757839.1|PUUV|N|Lithuania|M.g.|LT15/164|2015|  : .......G..............G......C.............................T.......T..................A..AG......... :  200
KX757840.1|PUUV|N|Lithuania|M.g.|LT15/174|2015|  : .......G..............G......C.............................T.......T..................A..AG......... :  200
KX815394.1|PUUV|N|Poland|M.g.|KS13/855|2009|     : .......G..............G......C..........A..................T.......T..................A..AG......... :  200
KX757841.1|PUUV|N|Lithuania|M.g.|LT15/201|2015|  : .......G.....T........G......C.A...........................T.......T..................A..AG......... :  200
AJ314598.1|PUUV|N|Russia|M.g.|Baltic/49Cg/00|    : ......................G......C.A..............A............T.....................A....A..AG......... :  200
AJ314599.1|PUUV|N|Russia|M.g.|Baltic/205Cg/00|   : ......................G......C.A..............A.................A..T.............A....A..AG......... :  200
JN657229.1|PUUV|N|Latvia|M.g.|Madona/Mg99/2008|  : ......................G......C.A..............A....................T..G..C.......A....A..AGG........ :  200
JN657232.1|PUUV|N|Latvia|M.g.|Madona/Mg233/2008| : ......................G......C.A..............A....................T..G..C.......A....A..AGG........ :  200
JN657230.1|PUUV|N|Latvia|M.g.|Jelgava/Mg136/2008|: ......................G......C.A..............A....................T..G..C.......A....A..AGG........ :  200
JN657231.1|PUUV|N|Latvia|M.g.|Jelgava/Mg140/2008|: ......................G......C.A..............A....................T..G..C.......A....A..AGG........ :  200
Z30707.1|PUUV|N|Russia|M.g.|Udmurtia/458Cg/88|   : .......G.....................C.A..............A............T.......T.....C..T....A....A..AG......... :  200
Z30706.1|PUUV|N|Russia|M.g.|Udmurtia/444Cg/88|   : .......G...........T.........C.A..............A............T.......T.....C.......A....A..AG......... :  200
Z84204.1|PUUV|N|Russia|M.g.|Kazan|               : .......G.....................C.A..............A............T.......T.....C.......A....A..AG......... :  200
Z30708.1|PUUV|N|Russia|M.g.|Udmurtia/338Cg/92|   : .......G.....................C.A..............A............T.......T.....C.......AT...A..AG......... :  200
Z21497.1|PUUV|N|Russia|M.g.|Udmurtia/894Cg/91|   : .......G.....................C.A..............A............T.......T.....C.......A....A..AG......... :  200
AB433843.2|PUUV|N|Russia|M.g.|Samara_49/CG/2005| : .......G.....................C.A..............A............T.......T.....C.......A....A..AG......... :  200
AB433845.2|PUUV|N|Russia|M.g.|Samara_94/CG/2005| : .......G.....................C.A..............A............T.......T.....C.......A....A..AG......... :  200
L11347.1|PUUV|N|Russia|Human|P360|               : ..T....G..............G........A........A.....A....................T.....C.......A....A..AG......... :  200
AB297665.2|PUUV|N|Russia|M.g.|DTK/Ufa-97|1997|   : ..T....G..............G........A........A.....A....................T.....C.......A....A..AG......... :  200
M32750.1|PUUV|N|Russia|M.g.|CG1820|              : .......G..............G.G......A........A.....A....................T.....C.......A....A..AG......... :  200
AF442613.1|PUUV|N|Russia|M.g.|CG17/Baskiria-2001|: .......G..............G........A..............A....................T.....C.......A....A..AG......... :  200
KX815395.1|PUUV|N|Poland|M.g.|KS14/118|2009|     : .......G.................T...C.A.........................................C.....T......A...G......... :  200
GQ339483.1|PUUV|N|Sweden|M.g.|Bergsjobo/Mg25/05| : ......................G......C.....................................T.....C.....T.....A...AG......... :  200
AJ223377.1|PUUV|N|Sweden|M.g.|Solleftea/Cg6/95|  : ......................G......C.....................................T.....C.....T.....A...AG......... :  200
GQ339484.1|PUUV|N|Sweden|M.g.|Faboviken/Mg26/05| : ......................G..T...C...........................................C.....T..T..A...AG......... :  200
GQ339485.1|PUUV|N|Sweden|M.g.|Mangelbo/Mg1/05|   : ......................G......C..........A..................T.......T.....C.....T.....A...AG......... :  200
GQ339486.1|PUUV|N|Sweden|M.g.|Munga/Mg2/05|      : ......................G......C..........A..........................T.....C.....T.....A...AG......... :  200
GQ339487.1|PUUV|N|Sweden|M.g.|Munga/Mg16/05|     : ......................G......C..........A..........................T.....C.....T.....A...AG......... :  200
AJ223376.1|PUUV|N|Sweden|M.g.|Solleftea/Cg3/95|  : ......................G..T...C...........................................C.....T..T..A...AG......... :  200
AF367071.1|PUUV|N|Russia|M.r.|CRF366|            : ...................T.........C.......C.....................T.......T.....C.......A....A.GAG......... :  200
AF367064.1|PUUV|N|Russia|M.g.|CG144|             : ...................T.........C.......C.....................T.......T.....C.......A....A.GAG......... :  200
AF367065.1|PUUV|N|Russia|M.g.|CG168|             : ...................T.........C.......C.....................T.......T.....C.......A....A.GAG......... :  200
AF367068.1|PUUV|N|Russia|M.g.|CG315|             : ...................T.........C.......C.....................T.......T.....C.......A....A.GAG......... :  200
AF367069.1|PUUV|N|Russia|M.r.|CRF161|            : ...................T.........C.......C.....................T.......T.....C.......A....A.GAG......... :  200
```

```
  Lagerqvist N et al. J Clin Microbiol 2016  PUUV_F  TGGACCCRGATGACGTTAACPUUV_PACACTGCAAGCAAGYGTTGTYTGTCACAGTCGTCAC  PUUV_R
Kramski M et al. Clin Chemistry 2007   (end PUUV F)  TGGACCCRGATGACGTTAA            PUUVTMGB  CAACARACAGTGTCAGCA                (end of PUUV R)  TACCGTCT

                                                                                                    Evander M et al. J Clin Microbiol  2007  S237 5’ GGCAGA
                                                              *        120         *        140         *        160         *        180         *        200
2014.00598|PUUV|N|France|H.s.|59|FOURMIES|         : TGGACCCAGATGACGTTAACAAAAACACATTGCAAGCAAGGCAACAGACAGTGTCAGCACTGGAGGACAAACTTGCAGACTTCAAGCGACAAATGGCAGA :  200
AF367070.1|PUUV|N|Russia|M.r.|CRF308|              : ...................T.........C.......C.....................T.......T.....C.......A....A.GAG......... :  200
AF367066.1|PUUV|N|Russia|M.g.|CG215|               : ...................T.........C.......C.....................T.......T.....C.......A....A.GAG......... :  200
AF367067.1|PUUV|N|Russia|M.g.|CG222|               : ...................T.........C.......C.....................T.......T.....C.......A....A.GAG......... :  200
Z46942.1|PUUV|N|Finland|M.g.|Puumala/1324Cg/79|    : .............................C.......C........A............T.......T.....C.......A....A..AG......... :  200
Z30702.1|PUUV|N|Finland|M.g.|Evo/12Cg/93|          : ...................T.........C.A.....C........A....................T.....C.......A....A..AGG........ :  200
Z30703.1|PUUV|N|Finland|M.g.|Evo/13Cg/93|          : ...................T.........C.A.....C........A....................T.....C.......A....A..AGG........ :  200
Z30704.1|PUUV|N|Finland|M.g.|Evo/14Cg/93|          : ...................T.........C.A.....C........A....................T.....C...A...A....A..AGG........ :  200
Z30705.1|PUUV|N|Finland|M.g.|Evo/15Cg/93|          : ...................T......G..C.A.....C........A....................T.....C.......A....A..AGG........ :  200
Z69985.1|PUUV|N|Finland|M.g.|Virrat/25Cg/95|       : ...................T.........C.......C...........................................A....A..AG......... :  200
JN831947.1|PUUV|N|Finland|Pieksamaki/human_lung/20 : .............................C.......C........A.................A..T.....C.......A....A..AGG........ :  200
JN831943.1|PUUV|N|Finland|Pieksamaki/Mg7/2008|     : .............................C.......C........A.................A..T.....C.......A....A..AGG........ :  200
JN831950.1|PUUV|N|Finland|Pieksamaki/human_kidney/ : .............................C.......C........A.................A..T.....C.......A....A..AGG........ :  200
JQ319166.1|PUUV|N|Finland|Konnevesi/Mg_O14B/2005|  : .........................T...C.A.....C........A....................T.....C.......A....A..AG......... :  200
JQ319169.1|PUUV|N|Finland|Konnevesi/Mg_O27B/2005|  : .........................T...C.......C........A....................T.....C.......A....A..AG......... :  200
JQ319167.1|PUUV|N|Finland|Konnevesi/Mg_O15B/2005|  : .............................C.......C........A....................T.....C.......A....A..AG......... :  200
JQ319164.1|PUUV|N|Finland|Konnevesi/Mg_O6B/2005|   : .........................T...C.......C........A....................T.....C.......A....A..AG......... :  200
JQ319165.1|PUUV|N|Finland|Konnevesi/Mg_O9B/2005|   : .........................T...C.......C........A....................T.....C.......A....A..AG......... :  200
JQ319170.1|PUUV|N|Finland|Konnevesi/Mg_O74B/2005|  : .........................T...C.A.....C........A....................T.....C.......A....A..AG......... :  200
JQ319171.1|PUUV|N|Finland|Konnevesi/Mg_M114B/2005| : .............................C.......C........A....................T.....C.......A....A..AG......... :  200
AJ238788.1|PUUV|N|Russia|M.g.|Karhumaki|           : ...................T.........C.......C...................................C.......A....A..A.G........ :  200
AJ238789.1|PUUV|N|Russia|M.g.|Kolodozero|          : ...................T.........C.......C...................................C..G....A....A..AGG........ :  200
AJ314597.1|PUUV|N|Finland|M.g.|Pallasjarvi/63Cg/98 : ...................T.........C.......C.............................T.....C.......A....A..AGG........ :  200
NC_005224.1|PUUV|N|Finland|M.g.|Sotkamo-revu-NCBI2 : ...................T.........C.......C........A..........................C.......A....A..AGG........ :  200
AJ238790.1|PUUV|N|Russia|M.g.|Gomselga|            : ...................T.........C.......C...................................C.......A....A..A.G........ :  200
AB010731.1|Hokkaido-V|N|Japan|M.r.|Tobetsu-60Cr-93 : ......................G......C........C.......A............T....A..T.....................AG......... :  200
AB010730.1|Hokkaido-V|N|Japan|M.r.|Kamiiso-8Cr-95| : ......................G......C........C.......A............T....A..T........C............AG......... :  200
JX046487.1|Muju-V|N|South-Korea|M.r.|11-5|2011     : .......C..............G......C....G...C....................T.......T..................A............. :  200
JX046484.1|Muju-V|N|South-Korea|M.r.|11-4|2011|    : .......C..............G......C....G...C....................T.......T..................A............. :  200
JX028273.1|Muju-V|N|South-Korea|M.r.|11-1|2011|    : .......C..............G......C....G...C....................T.......T..................A............. :  200
DQ138128.1|Muju-V|N|South-Korea|E.r.|00-18|        : ......................G......C....G...C.......A............T.......T..................A............. :  200
DQ138140.1|Muju-V|N|South-Korea|E.r.|99-27|        : ......................G......C....G...C.......A............T.......T..................A............. :  200
DQ138133.1|Muju-V|N|South-Korea|E.r.|96-1|         : ......................G......C....G...C.......A............T.......T..................A............. :  200
DQ138142.1|Muju-V|N|South-Korea|E.r.|99-28|        : ......................G......C....G...C.......A............T.......T..................A............. :  200
```

```
                                                                                                                    Bowen MD et al. J Med Virol 1997  PPT334C  TATGGIAAT
                                                     ACYACACAGGKCC  PUUV R  Kramski M et al. Clinical Chemistry 2007
                                                                                         GTTGACCCTAACTCGGTCTAC  S304 3’
                                                     TGCTGTGTCCAGG       TGGATACAAAGCCTACTGAT  S263-282              (Evander M et al. J Clin Microbiol  2007)
                                                              *       220         *       240         *       260         *       280         *       300
2014.00598|PUUV|N|France|H.s.|59|FOURMIES|          : TGCTGTGTCCAGGAAAAAAATGGATACTAAACCTACTGACCCGACTGGGATTGAGCCTGATGACCATCTCAAAGAGCGATCAAGCCTCAGATATGGGAAT : 300
2014.00233|PUUV|N|France|H.s.|59|FOURMIES|          : .................................................................................................... : 300
2014.00097|PUUV|N|France|H.s.|02|SAINT-MICHEL|      : .................................................................................................... : 300
2014.00488|PUUV|N|France|H.s.|08|SIGNY-LE-PETIT|    : .................................................................................................... : 300
2014.00613|PUUV|N|France|H.s.|59|FOURMIES|          : .................................................................................................... : 300
2015.00402|PUUV|N|France|H.s.|08|CHARLEVILLE-MEZIE  : ........................................................................G...A....................... : 300
2015.00422|PUUV|N|France|H.s.|59|ANOR|              : .................................................................................................... : 300
2015.00488|PUUV|N|France|H.s.|08|SEDAN|             : ........................................................................G........................... : 300
2015.00498|PUUV|N|France|H.s.|08|REMILLY-AILLICOUR  : .................................................................................................... : 300
2016.00295|PUUV|N|France|H.s.|08|CORNY-MACHEROMENI  : .................................................................................................... : 300
2016.00333|PUUV|N|France|H.s.|59|FOURMIES|          : .................................................................................................... : 300
2016.00345|PUUV|N|France|H.s.|59|FOURMIES|          : .................................................................................................... : 300
2016.00357|PUUV|N|France|H.s.|02|HIRSON|            : .................................................................................................... : 300
2016.00427|PUUV|N|France|H.s.|59|FOURMIES|          : .................................................................................................... : 300
2016.00469|PUUV|N|France|H.s.|02|PREMONTRE|         : ..................G.....................................................G........................... : 300
AJ277075.1|PUUV|N|Belgium|M.g.|Montbliart/CG14444|  : ........................................................................G.......................A... : 300
2014.00053|PUUV|N|France|H.s.|08|SECHEVAL|          : ........................................................................G........................... : 300
KT247593.1|PUUV|N|France|M.g.|08|Ardennes/Mg75/201  : ........................................................................G........................... : 300
KT247592.1|PUUV|N|France|M.g.|08|Ardennes/Mg156/20  : ........................................................................G........................... : 300
2015.00019|PUUV|N|France|H.s.|08|ETEIGNIERES|       : ........................................................................G........................... : 300
2016.00239|PUUV|N|France|H.s.|08|VIREUX-MOLHAIN|    : ........................................................................G........................... : 300
2015.00329|PUUV|N|France|H.s.|08|BOGNY-SUR-MEUSE|   : ........................................................................G........................... : 300
2014.00209|PUUV|N|France|H.s.|08|TREMBLOIS-LES-ROC  : ........................................................................G........................... : 300
2015.00456|PUUV|N|France|H.s.|94|ALFORTVILLE|       : ........................................................................G........................... : 300
2012.00018|PUUV|N|France|H.s.|08|ROCROI|            : ........................................................................G........................... : 300
AJ277032.1|PUUV|N|Belgium|M.g.|Momignies/47Cg/96|   : ........................................................................G........................... : 300
AJ277034.1|PUUV|N|Belgium|M.g.|Couvin/59Cg/97|      : ........................................................................G........................... : 300
AJ277033.1|PUUV|N|Belgium|M.g.|Momignies/55Cg/96|   : ........................................................................G........................... : 300
2012.00638|PUUV|N|France|H.s.|08|GIVET|             : .................................................................................................... : 300
2015.00328|PUUV|N|France|H.s.|08|VRIGNE-MEUSE|      : ............................................................C...........G...A................C...... : 300
2015.00419|PUUV|N|France|H.s.|08|DOUZY|             : ........................................................................G........................... : 300
2016.00310|PUUV|N|France|H.s.|08|THIN-LE-MOUTIER|   : .................................................................................................... : 300
AJ277030.1|PUUV|N|Belgium|M.g.|Thuin/33Cg/96|       : .......................................T................................G..A........................ : 300
AJ238779.1|PUUV|N|Germany|M.g.|NRW/Cg-Erft|         : ..................G.....................................................G........................... : 300
2014.00276|PUUV|N|France|H.s.|59|LILLE|             : ........................................................................G..A........................ : 300
U22423.1|PUUV|N|Belgium|M.g.|CG13891|               : ......C..A........G.....................................................G..A........................ : 300
2014.00321|PUUV|N|France|H.s.|59|FOURMIES|          : .................................................................................................... : 300
2015.00457|PUUV|N|France|H.s.|55|REVIGNY-SUR-ORNAI  : ..................G.....................................................G........................... : 300
2013.00250|PUUV|N|France|H.s.|62|VENDIN-LS-BTHUNE|  : ..................G............................................T........G...........T............... : 300
2016.00282|PUUV|N|France|H.s.|59|MORBECQUE|         : ..................G............................................T..C.....G........................... : 300
AJ277076.1|PUUV|N|Belgium|M.g.|Montbliart/CG14445|  : ........................................................................G........G..............A... : 300
2014.00153|PUUV|N|France|H.s.|02|BUIRONFOSSE|       : ........................................................................G........................... : 300
2014.00171|PUUV|N|France|H.s.|59|SAINT-SAULVE|      : ..................G.....................................................G..............T..G......... : 300
2016.00293|PUUV|N|France|H.s.|60|LA-NEUVILLE-SUR-R  : ..................G.....................................................G...A....................... : 300
2014.00499|PUUV|N|France|H.s.|94|CHAMPIGNY-SUR-MAR  : ........................................................................G........................... : 300
2012.00057|PUUV|N|France|H.s.|59|COUSOLRE|          : ........................................................................G........................... : 300
2012.00349|PUUV|N|France|H.s.|02|ENGLANCOURT|       : ........................................................................G........................... : 300
2014.00135|PUUV|N|France|H.s.|02|ATHIES-SOUS-LAON|  : ...............................................................T.....T..G...A....................... : 300
2014.00184|PUUV|N|France|H.s.|59|FOURMIES|          : .................................................................................................... : 300
2016.00182|PUUV|N|France|H.s.|08|BOULZICOURT|       : ........................................................................G.......................A... : 300
2016.00286|PUUV|N|France|H.s.|02|TRUCY|             : ...........................C............................................G........................... : 300
2016.00325|PUUV|N|France|H.s.|59|AULNOYE-AYMERIES|  : ........................................................................G........................... : 300
2016.00452|PUUV|N|France|H.s.|02|LAON|              : ...............................................................T.....T..G...A....................... : 300
2016.00467|PUUV|N|France|H.s.|02|AISNE|             : ........................................................................G.......................A... : 300
```

```
                                                                                  Bowen MD et al. J Med Virol 1997  PPT334C  TATGGIAAT
                                              ACYACACAGGKCC  PUUV R  Kramski M et al. Clinical Chemistry 2007
                                                                                GTTGACCCTAACTCGGTCTAC  S304 3'
                                              TGCTGTGTCCAGG       TGGATACAAAGCCTACTGAT  S263-282              (Evander M et al. J Clin Microbiol  2007)
                                                       *       220         *       240         *       260         *       280         *       300
2014.00598|PUUV|N|France|H.s.|59|FOURMIES|    : TGCTGTGTCCAGGAAAAAAATGGATACTAAACCTACTGACCCGACTGGGATTGAGCCTGATGACCATCTCAAAGAGCGATCAAGCCTCAGATATGGGAAT :  300
2015.00657|PUUV|N|France|H.s.|02|CILLY|       : ..................G.....................................................G........................... :  300
2015.00526|PUUV|N|France|H.s.|08|MONTHERME|   : ........................................................................G........................... :  300
2015.00045|PUUV|N|France|H.s.|59|FOURMIES|    : ........................................................................G........................... :  300
2015.00430|PUUV|N|France|H.s.|02|MONTCORNET|  : ................................................A.......................G........................... :  300
2016.00311|PUUV|N|France|H.s.|02|LAON|        : ........................................................................G........................... :  300
AJ277031.1|PUUV|N|Belgium|M.g.|Montbliart/23Cg/96| : ........................................................................G.......................A... :  300
2016.00326|PUUV|N|France|H.s.|02|LAON|        : .AT.....................................................................G........................... :  300
2016.00353|PUUV|N|France|H.s.|02|CESSIERES|   : ..T......................G..............................................G........................... :  300
2012.00061|PUUV|N|France|H.s.|02|LANISCOURT|  : ..T.....................................................................G........................... :  300
2014.00174|PUUV|N|France|H.s.|02|ST-ERME-OUTRE-ET- : ..T.....................................................................G........................... :  300
2016.00268|PUUV|N|France|H.s.|02|PRESLES-ET-THIERN : ..T.....................................................................G........................... :  300
2015.00660|PUUV|N|France|H.s.|02|VIC-SUR-AISNE| : .....................................................................T..G........................... :  300
2012.00025|PUUV|N|France|H.s.|51|SAINTE-MENEHOULD| : ..................G............................................T..C.....G...........T..T..G.....A... :  300
2015.00665|PUUV|N|France|H.s.|51|REIMS|       : ..................G............................................T..C.....G........G..T..T..G.....A... :  300
2012.00307|PUUV|N|France|H.s.|54|COLOMBEY-LES-BELL : .........T............................................A........T..C.....G...........T..T..G.....A... :  300
2012.00123|PUUV|N|France|H.s.|55|BAR-LE-DUC|  : ...............................................................T..C.....G...........T..T..G.....A... :  300
2012.00278|PUUV|N|France|H.s.|51|REIMS|       : ..................G............................................T..C.....G...........T..T........A... :  300
2015.00185|PUUV|N|France|H.s.|68|FELLERING|   : ..................G............................................T..C.....G...........T.....G.....A... :  300
KJ994776.1|PUUV|N|Germany|M.g.|Mu/07/1219|2007| : ..............................G........T..............A.....C..T..C.....G..A..G.....T.....G.....A... :  300
2012.00086|PUUV|N|France|H.s|58|CHEVROCHES|   : ..................G...........G.......................A.................G...........T..T..G.....A... :  300
KT247595.1|PUUV|N|France|M.g.|45|Orleans/Mg29/2010 : ..................G...........G...G...................A.................G..............T..G.....A... :  300
KT247594.1|PUUV|N|France|M.g.|45|Orleans/Mg23/2010 : ..................G...........G...G...................A.................G..............T..G.....A... :  300
KY365004.1|PUUV|N|France|M.g.|45|Orleans/NCHA373/2 : ..................G...........G...G...................A.................G..............T..G.....A... :  300
2012.00301|PUUV|N|France|H.s.|25|MOUTHE|      : ..................G...........G.......................A...........C.....G...........T..T........A... :  300
AM695638.1|PUUV|N|France|M.g.|Mignovillard/CgY02/2 : ..................G...........G.......................A...........C.....G...........T..T..G.....A... :  300
KT247597.1|PUUV|N|France|M.g.|39|Jura/Mg214/2010| : ..................G....................................A...........C.....G...........T..T..G.....A... :  300
KT247596.2|PUUV|N|France|M.g.|39|Jura/Mg2/2010| : ..................G....................................A...........C.....G...........T..T..G.....A... :  300
2012.00102|PUUV|N|France|H.s.|39|COISERETTE|  : ..................G....................................A...........C.....G...........T..T..G.....A... :  300
2012.00536|PUUV|N|France|H.s.|39|LA-PESSE|    : ..................G....................................A...........C.....G...........T..T..G.....A... :  300
2014.00622|PUUV|N|France|H.s.|39|ARBOIS|      : ..................G...........G.......................A...........C.....G..............T..G......... :  300
2015.00567|PUUV|N|France|H.s.|70|RIOZ|        : ..................G....................................A...........C.....G...........T..T..G.....A... :  300
2014.00637|PUUV|N|France|H.s.|25|SAULES|      : ..................G....................................A...........C.....G...........T..T..G.....A... :  300
2012.00396|PUUV|N|France|H.s.|39|SAINT-CLAUDE| : ..................G...........G.......................A...........C.....G...........T..T..G......... :  300
2014.00120|PUUV|N|France|H.s.|38|LE-MOUTARET| : ..................G...........G.......................A...........C.....G...........T..T..G......... :  300
2015.00153|PUUV|N|France|H.s.|73|GREZY-SUR-ISERE| : ..................G...........G.......................A...........C.....G...........T..T..G......... :  300
2015.00504|PUUV|N|France|H.s.|70|RONCHAMP|    : ..................G....................................A.................G...........T..T..G......... :  300
2016.00275|PUUV|N|France|H.s.|21|JALLANGES|   : ..................G...........G.......................A...........C.....G...........T..T..G.....A... :  300
2016.00320|PUUV|N|France|H.s.|25|SAINT-VIT|   : ..................G....................................A...........C.....G...........T..T..G.....A... :  300
2015.00410|PUUV|N|France|H.s.|70|ANGIREY|     : ..................G....................................A.................G...........T..T..G......... :  300
DQ016430.2|PUUV|N|Germany|M.g.|Bavaria/CG33/04| : ..........C.......G...........G........T..............A..A.................A...........T..G.....A... :  300
DQ016432.2|PUUV|N|Germany|M.g.|Bavaria/CG41/04| : ..........C.......G...........G........T..............A..............T.....A...........T..G.....A... :  300
AY954723.2|PUUV|N|Germany|M.g.|Bavaria-CG34/04| : ..........C.......G...........G........T..............A..A...........T.....A...........T..G.....A... :  300
AY954722.2|PUUV|N|Germany|M.g.|Bavaria-CG9/04| : ..........C.......G...........G........T..............A..A...........T.....A...........T..G.....A... :  300
2012.00402|PUUV|N|France|H.s.|60|GOUVIEUX|    : ..................G....................................................................T..G.....A... :  300
2014.00540|PUUV|N|France|H.s.|60|CHAMBLY|     : ..................G......................................C..............G..............T..G.....A... :  300
AJ314600.1|PUUV|N|Balkan|M.g.|Balkan-1|       : ..................G...........G.......................A...........C..T..G..............T..G.....A... :  300
AJ314601.1|PUUV|N|Balkan|M.g.|Balkan-2|       : ..................G...........G.......................A...........C.....G..............T..G.....A... :  300
FN377821.1|PUUV|N|Hungary|M.g.|Mg9/HungaryTR17/00| : ..................G...........G.......................A...........C.....G..............T..G.....A... :  300
FN377822.1|PUUV|N|Hungary|M.g.|Mg23/HungaryTR17/00 : ..............................G.......................A...........C.....G..............T..G.....A... :  300
GQ339473.1|PUUV|N|Sweden|M.g.|Kiviniemi/Mg3/05| : ...............G..............G...........A..............A........C.....G..AA..........GC.......A... :  300
GQ339474.1|PUUV|N|Sweden|M.g.|Kiviniemi/Mg5/05| : ...............G..............G...........A..............A.....T..C.....G..AA..........GC.......A... :  300
GQ339475.1|PUUV|N|Sweden|M.g.|Kiviniemi/Mg6/05| : ...............G..............G...........A..............A.....T..C.....G..AA..........GC.......A... :  300
GQ339476.1|PUUV|N|Sweden|M.g.|Aijajarvi/Mg7/05| : ...............G..............G...........A..............A........C.....G..AA..........GC.......A... :  300
```

```
                                                                                                      Bowen MD et al. J Med Virol 1997  PPT334C  TATGGIAAT
                                                      ACYACACAGGKCC  PUUV R  Kramski M et al. Clinical Chemistry 2007
                                                                                               GTTGACCCTAACTCGGTCTAC  S304 3’
                                                      TGCTGTGTCCAGG       TGGATACAAAGCCTACTGAT  S263-282                (Evander M et al. J Clin Microbiol  2007)
                                                               *       220         *       240         *       260         *       280         *       300
2014.00598|PUUV|N|France|H.s.|59|FOURMIES|          : TGCTGTGTCCAGGAAAAAAATGGATACTAAACCTACTGACCCGACTGGGATTGAGCCTGATGACCATCTCAAAGAGCGATCAAGCCTCAGATATGGGAAT :  300
GQ339477.1|PUUV|N|Sweden|M.g.|Aijajarvi/Mg9/05|     : ...............G..............G...........A..............A........C.....G..AA..........GC.......A... :  300
GQ339478.1|PUUV|N|Sweden|M.g.|Jockfall/Mg12/05|     : ...............G..G...........G...........A..............A........C.....G..AA..........GC.......A... :  300
GQ339482.1|PUUV|N|Sweden|M.g.|Kalvudden/Mg22/05|    : ..................G...........G...........A..............A........C.....G..AA..........GC.G.....A... :  300
GQ339479.1|PUUV|N|Sweden|M.g.|Moskosel/Mg17/05|     : C..............G..............G........T..A..............A........C.....G..AA........T.GC.......A... :  300
GQ339481.1|PUUV|N|Sweden|M.g.|Ljustrask/Mg20/05|    : ...............G..G...........G........T..A..............A........C.....G...A........T.GC.......A... :  300
GQ339480.1|PUUV|N|Sweden|M.g.|Gyttjea/Mg19/05|      : ...............G..G...........G........T..A..............A........C.....G..AA.......TT.GC.......A... :  300
AY526219.1|PUUV|N|Sweden|Human|Umea/hu|             : ...............G...........A..G........T..A..............A........C.....G..A...........GC.......A... :  300
AJ223380.1|PUUV|N|Sweden|M.g.|Tavelsjo/Cg81/94|     : ..................G........A..G........T..A..............A........C.....G...A..........GC.......A... :  300
U14137.1|PUUV|N|Bosnia-H.|M.g.|Vranica|             : ..................G........A..G........T..A..............A........C.....G...A..........GC.......A... :  300
AJ223371.1|PUUV|N|Sweden|M.g.|Huggberget/Cg36/94|   : ..................G........A..G........T..A..............A........C.....G..............GC.......A... :  300
Z48586.1|PUUV|N|Sweden|M.g.|Vindeln/L20Cg/83|       : ...............G..G........A..G........T..A..............A........C.....G..............GC.T.....A... :  300
AJ223374.1|PUUV|N|Sweden|M.g.|Mellansel/Cg47/94|    : ...........................A..G........T..A..............A......T.C.....G..A...........GC.......A... :  300
AJ223375.1|PUUV|N|Sweden|M.g.|Mellansel/Cg49/94|    : ...........................A..G........T..A..............A........C.....G..A...........GC.......A... :  300
AJ238791.1|PUUV|N|Denmark|M.g.|Fyn/19|              : ..........C.......G.......................A...........A...........C........AA.......T........C..A... :  300
AJ278092.1|PUUV|N|Denmark|M.g.|Fyn47|               : ..........C.......G.......................A...........A...........C........AA.......T...........A... :  300
AJ278093.1|PUUV|N|Denmark|M.g.|Fyn131|              : ..........C.......G.......................A...........A...........C........AA.......T...........A... :  300
AJ223368.1|PUUV|N|Norway|M.g.|Eidsvoll/1124v|       : ..........C....G..............G........T..............A.....................A.......T...........A... :  300
AJ223369.1|PUUV|N|Norway|M.g.|Eidsvoll/Cg1138/87|   : ..........C....G..............G........T..............A.....................A.......T...........A... :  300
JN657228.1|PUUV|N|Latvia|M.g.|Jelgava/Mg149/2008|   : ..........C.......G...................................A...........C..T..G..A..............G.....A... :  300
KX757839.1|PUUV|N|Lithuania|M.g.|LT15/164|2015|     : ......................................................A........T........G..A..............G.....A... :  300
KX757840.1|PUUV|N|Lithuania|M.g.|LT15/174|2015|     : ......................................................A........T........G..A..............G.....A... :  300
KX815394.1|PUUV|N|Poland|M.g.|KS13/855|2009|        : ..................G...................................A...........C..T..G..A..............G.....A... :  300
KX757841.1|PUUV|N|Lithuania|M.g.|LT15/201|2015|     : ...................................T..................A........T........G..A..............G.....A... :  300
AJ314598.1|PUUV|N|Russia|M.g.|Baltic/49Cg/00|       : ..........C....G..G...................................A...........C.....G...A...........C.......A... :  300
AJ314599.1|PUUV|N|Russia|M.g.|Baltic/205Cg/00|      : ..........C............................T..............A...........C.....G..AA..........TC.......A... :  300
JN657229.1|PUUV|N|Latvia|M.g.|Madona/Mg99/2008|     : ..........C....G..............G.......................A.................G..AA..........T........A... :  300
JN657232.1|PUUV|N|Latvia|M.g.|Madona/Mg233/2008|    : ..........C....G..............G.......................A.................G..AA..........T........A... :  300
JN657230.1|PUUV|N|Latvia|M.g.|Jelgava/Mg136/2008|   : ..........C....G..................G...................A.................G..AA..........T........A... :  300
JN657231.1|PUUV|N|Latvia|M.g.|Jelgava/Mg140/2008|   : ..........C....G..................G...................A.................G..AA..........T........A... :  300
Z30707.1|PUUV|N|Russia|M.g.|Udmurtia/458Cg/88|      : ......................................................A........T........G...A..........T........A... :  300
Z30706.1|PUUV|N|Russia|M.g.|Udmurtia/444Cg/88|      : ..........C...........................................A........T........G...A..........T........A... :  300
Z84204.1|PUUV|N|Russia|M.g.|Kazan|                  : ..........C...........................................A........T........G...A..........T........A... :  300
Z30708.1|PUUV|N|Russia|M.g.|Udmurtia/338Cg/92|      : .T........C............................T..............A........T........G...A.........CT........A... :  300
Z21497.1|PUUV|N|Russia|M.g.|Udmurtia/894Cg/91|      : ..........C...................G....................C..A........T........G...A..........T........A... :  300
AB433843.2|PUUV|N|Russia|M.g.|Samara_49/CG/2005|    : ..........C....G......................................A.................G...A....C.....T........A... :  300
AB433845.2|PUUV|N|Russia|M.g.|Samara_94/CG/2005|    : ..........C....G......................................A.................G...A..........T........A... :  300
L11347.1|PUUV|N|Russia|Human|P360|                  : ..........C....G......................................A........T........G...A..........T........A... :  300
AB297665.2|PUUV|N|Russia|M.g.|DTK/Ufa-97|1997|      : ..........C....G......................................A........T........G...A..........T........A... :  300
M32750.1|PUUV|N|Russia|M.g.|CG1820|                 : ..........C....G......................................A........T........G...A..........T........A... :  300
AF442613.1|PUUV|N|Russia|M.g.|CG17/Baskiria-2001|   : ..........C....G......................................A........T........G...A..........T........A... :  300
KX815395.1|PUUV|N|Poland|M.g.|KS14/118|2009|        : ..........C....G..............G.......................A...........C..T..G...A...................A... :  300
GQ339483.1|PUUV|N|Sweden|M.g.|Bergsjobo/Mg25/05|    : ..........C....................................................T..C.....G..AA...................A... :  300
AJ223377.1|PUUV|N|Sweden|M.g.|Solleftea/Cg6/95|     : ..........C....................................................T..C.....G..AA...................A... :  300
GQ339484.1|PUUV|N|Sweden|M.g.|Faboviken/Mg26/05|    : ..........C............................T..............A...........C..T..G..AAT..................A... :  300
GQ339485.1|PUUV|N|Sweden|M.g.|Mangelbo/Mg1/05|      : ..........C....G.......................T..............A..A.....T...........AA.......T............G.. :  300
GQ339486.1|PUUV|N|Sweden|M.g.|Munga/Mg2/05|         : ..........C............................T..............A..A.....T........G..AA.......T...........A... :  300
GQ339487.1|PUUV|N|Sweden|M.g.|Munga/Mg16/05|        : ..........C............................T..............A..A.....T........G..AA.......T...........A... :  300
AJ223376.1|PUUV|N|Sweden|M.g.|Solleftea/Cg3/95|     : ..........C............................T..............A...........C.........A...................A... :  300
AF367071.1|PUUV|N|Russia|M.r.|CRF366|               : ..........C.......G...........G...G...................A...........C.....G...A.......T..T........A... :  300
AF367064.1|PUUV|N|Russia|M.g.|CG144|                : ..........C.......G...........G...G...................A...........C.....G...A.......T..T........A... :  300
AF367065.1|PUUV|N|Russia|M.g.|CG168|                : ..........C.......G...........G...G...................A...........C.....G...A.......T..T........A... :  300
AF367068.1|PUUV|N|Russia|M.g.|CG315|                : ..........C.......G...........G...G...................A...........C.....G...A.......T..T........A... :  300
AF367069.1|PUUV|N|Russia|M.r.|CRF161|               : ..........C.......G...........G...G...................A...........C.....G...A.......T..T........A... :  300
```

```
                                                                                                            Bowen MD et al. J Med Virol 1997  PPT334C  TATGGIAAT
                                                   ACYACACAGGKCC  PUUV R  Kramski M et al. Clinical Chemistry 2007
                                                                                          GTTGACCCTAACTCGGTCTAC  S304 3'
                                                   TGCTGTGTCCAGG       TGGATACAAAGCCTACTGAT  S263-282              (Evander M et al. J Clin Microbiol  2007)
                                                            *        220         *        240         *        260         *        280         *        300
2014.00598|PUUV|N|France|H.s.|59|FOURMIES|         : TGCTGTGTCCAGGAAAAAAATGGATACTAAACCTACTGACCCGACTGGGATTGAGCCTGATGACCATCTCAAAGAGCGATCAAGCCTCAGATATGGGAAT :  300
AF367070.1|PUUV|N|Russia|M.r.|CRF308|              : ..........C.......G...........G...G.....................A...........C.....G...A.......T..T........A... :  300
AF367066.1|PUUV|N|Russia|M.g.|CG215|               : ..........C.......G...........G...G.....................A...........C.....G...A.......T..T........A... :  300
AF367067.1|PUUV|N|Russia|M.g.|CG222|               : ..........C.......G...........G...G.....................A...........C.....G...A.......T..T........A... :  300
Z46942.1|PUUV|N|Finland|M.g.|Puumala/1324Cg/79|    : ..................G...............G.....................A........T..C.....G...A.......T..T..G.....A... :  300
Z30702.1|PUUV|N|Finland|M.g.|Evo/12Cg/93|          : ..........C................A...........T..............A........T..C..T..G...A..........T..G.....A... :  300
Z30703.1|PUUV|N|Finland|M.g.|Evo/13Cg/93|          : ..........C................A...........T..............A........T..C..T..G...A..........T..G.....A... :  300
Z30704.1|PUUV|N|Finland|M.g.|Evo/14Cg/93|          : ..........C................G..........................A........T..C.....G...A..........T..G.....A... :  300
Z30705.1|PUUV|N|Finland|M.g.|Evo/15Cg/93|          : ..........C................A..........................A........T..C.....G...A..........T..G.....A... :  300
Z69985.1|PUUV|N|Finland|M.g.|Virrat/25Cg/95|       : ..........C.......G...................................A...........C.....G...A..........T..G.....A... :  300
JN831947.1|PUUV|N|Finland|Pieksamaki/human_lung/20 : ......................................................A..G.....T..C.....G...A..........T..G.....A... :  300
JN831943.1|PUUV|N|Finland|Pieksamaki/Mg7/2008|     : ......................................................A..G.....T..C.....G...A..........T..G.....A... :  300
JN831950.1|PUUV|N|Finland|Pieksamaki/human_kidney/ : ......................................................A..G.....T..C.....G...A..........T..G.....A... :  300
JQ319166.1|PUUV|N|Finland|Konnevesi/Mg_O14B/2005|  : ......................................................A........T..C.....G...A..........T..G.....A... :  300
JQ319169.1|PUUV|N|Finland|Konnevesi/Mg_O27B/2005|  : ......................................................A........T..C.....G...A..........T..G.....A... :  300
JQ319167.1|PUUV|N|Finland|Konnevesi/Mg_O15B/2005|  : ..................G...................................A........T..C.....G...A..........T..G.....A... :  300
JQ319164.1|PUUV|N|Finland|Konnevesi/Mg_O6B/2005|   : ......................................................A........T..C.....G...A..........T..G.....A... :  300
JQ319165.1|PUUV|N|Finland|Konnevesi/Mg_O9B/2005|   : ......................................................A........T..C.....G...A..........T..G.....A... :  300
JQ319170.1|PUUV|N|Finland|Konnevesi/Mg_O74B/2005|  : ......................................................A........T..C.....G...A..........T..G.....A... :  300
JQ319171.1|PUUV|N|Finland|Konnevesi/Mg_M114B/2005| : ..................G...................................A........T..C.....G...A..........T..G.....A... :  300
AJ238788.1|PUUV|N|Russia|M.g.|Karhumaki|           : ..........C........................................GA.A.......TT..C.........A...........GAG.....A... :  300
AJ238789.1|PUUV|N|Russia|M.g.|Kolodozero|          : ..........C............................T..............A...........C.....G...A.............G.....A... :  300
AJ314597.1|PUUV|N|Finland|M.g.|Pallasjarvi/63Cg/98 : ..........C...........................................A...........C.....G...A..........T..G.....A... :  300
NC_005224.1|PUUV|N|Finland|M.g.|Sotkamo-revu-NCBI2 : ......................................................A...........C.....G...A..........T..G.....A... :  300
AJ238790.1|PUUV|N|Russia|M.g.|Gomselga|            : ..........C...........................................A........T..C.........A.............G.....A... :  300
AB010731.1|Hokkaido-V|N|Japan|M.r.|Tobetsu-60Cr-93 : ..T...............G...........G...G.....................A...........C.....G..TA....T.................. :  300
AB010730.1|Hokkaido-V|N|Japan|M.r.|Kamiiso-8Cr-95| : ..T...............G...........G...G.....................A...........C.....G...A....T.................. :  300
JX046487.1|Muju-V|N|South-Korea|M.r.|11-5|2011     : CTT.......C.......G...........G........T....................C..T..C.....G..AA.............G......... :  300
JX046484.1|Muju-V|N|South-Korea|M.r.|11-4|2011|    : CTT.......C.......G...........G........T....................C..T..C.....G..AA.............G......... :  300
JX028273.1|Muju-V|N|South-Korea|M.r.|11-1|2011|    : CTT.......C.......G...........G........T....................C..T..C.....G..AA.............G......... :  300
DQ138128.1|Muju-V|N|South-Korea|E.r.|00-18|        : CTT...............G...........G...................................C.........A.......T............... :  300
DQ138140.1|Muju-V|N|South-Korea|E.r.|99-27|        : CTT...............G...........G...................................C.........A.......T............... :  300
DQ138133.1|Muju-V|N|South-Korea|E.r.|96-1|         : CTT...............G...........G.......................A...........C.........A.......T............... :  300
DQ138142.1|Muju-V|N|South-Korea|E.r.|99-28|        : CTT...............G...........G...................................C.........A.......T............... :  300
```

```
                                                    GTCCTTGATGT  (end of PPT334C)    CCIAGTGGICAIACAGC  PPT376C  Bowen MD et al. J Med Virol 1997

                                          Aitichou M at al J Virol Meth 2005 HANTAV1U     GWGGVCARACAGCWGAYT                     PUU1P  TTCACAATTCCTATCA
                                                             *         320         *         340         *         360         *         380         *         400
2014.00598|PUUV|N|France|H.s.|59|FOURMIES|         : GTCCTTGATGTGAATGCCATTGATATAGAAGAACCAAGTGGTCAAACAGCAGACTGGTATACAATTGGAGTGTATGTTATAGGCTTCACACTCCCCATCA :  400
2014.00233|PUUV|N|France|H.s.|59|FOURMIES|         : ...........A................................................................................T..T..T. :  400
2014.00097|PUUV|N|France|H.s.|02|SAINT-MICHEL|     : .................................................................................................... :  400
2014.00488|PUUV|N|France|H.s.|08|SIGNY-LE-PETIT|   : ............................................................................................T....... :  400
2014.00613|PUUV|N|France|H.s.|59|FOURMIES|         : .................................................................................................... :  400
2015.00402|PUUV|N|France|H.s.|08|CHARLEVILLE-MEZIE : ............................................................................................T..T..T. :  400
2015.00422|PUUV|N|France|H.s.|59|ANOR|             : .................................................................................................... :  400
2015.00488|PUUV|N|France|H.s.|08|SEDAN|            : ............................................................................................T..T..T. :  400
2015.00498|PUUV|N|France|H.s.|08|REMILLY-AILLICOUR : .................................................................................................... :  400
2016.00295|PUUV|N|France|H.s.|08|CORNY-MACHEROMENI : ...........A.................G.................................................................T.... :  400
2016.00333|PUUV|N|France|H.s.|59|FOURMIES|         : .................................................................................................... :  400
2016.00345|PUUV|N|France|H.s.|59|FOURMIES|         : .................................................................................................... :  400
2016.00357|PUUV|N|France|H.s.|02|HIRSON|           : .................................................................................................... :  400
2016.00427|PUUV|N|France|H.s.|59|FOURMIES|         : .............................................................................C...................... :  400
2016.00469|PUUV|N|France|H.s.|02|PREMONTRE|        : ...........A...........................................................A....................T..T..T. :  400
AJ277075.1|PUUV|N|Belgium|M.g.|Montbliart/CG14444| : ...........A................................................................................T..T..T. :  400
2014.00053|PUUV|N|France|H.s.|08|SECHEVAL|         : ............................................................................................T..T..TG :  400
KT247593.1|PUUV|N|France|M.g.|08|Ardennes/Mg75/201 : ...........A.....T..........................................................................T..T..T. :  400
KT247592.1|PUUV|N|France|M.g.|08|Ardennes/Mg156/20 : ...........A.....T..........................................................................T..T..T. :  400
2015.00019|PUUV|N|France|H.s.|08|ETEIGNIERES|      : ...........A.....T..........................................................................T..T..T. :  400
2016.00239|PUUV|N|France|H.s.|08|VIREUX-MOLHAIN|   : ...........A................................................................................T..T..T. :  400
2015.00329|PUUV|N|France|H.s.|08|BOGNY-SUR-MEUSE|  : ...........A................................................................................T..T..T. :  400
2014.00209|PUUV|N|France|H.s.|08|TREMBLOIS-LES-ROC : ...........A..........................................................................T.....T..T..T. :  400
2015.00456|PUUV|N|France|H.s.|94|ALFORTVILLE|      : ...........A................................................................................T..T..T. :  400
2012.00018|PUUV|N|France|H.s.|08|ROCROI|           : ...........A.................................................................G........T.....T..T..T. :  400
AJ277032.1|PUUV|N|Belgium|M.g.|Momignies/47Cg/96|  : ...........A..............................................................C..C..............T..T..T. :  400
AJ277034.1|PUUV|N|Belgium|M.g.|Couvin/59Cg/97|     : ...........A................................................................................T..T..T. :  400
AJ277033.1|PUUV|N|Belgium|M.g.|Momignies/55Cg/96|  : ...........A..............................................................C..C..............T..T..T. :  400
2012.00638|PUUV|N|France|H.s.|08|GIVET|            : ...........A...............................................C.....................A..........T..T..T. :  400
2015.00328|PUUV|N|France|H.s.|08|VRIGNE-MEUSE|     : ......................................................................................T.....T..T..T. :  400
2015.00419|PUUV|N|France|H.s.|08|DOUZY|            : ......................................................................................T.....T..T..T. :  400
2016.00310|PUUV|N|France|H.s.|08|THIN-LE-MOUTIER|  : ...........A...................................................................................T.... :  400
AJ277030.1|PUUV|N|Belgium|M.g.|Thuin/33Cg/96|      : ...........A.........................................T................................T.....T..T..T. :  400
AJ238779.1|PUUV|N|Germany|M.g.|NRW/Cg-Erft|        : ............................................................................................T..T..T. :  400
2014.00276|PUUV|N|France|H.s.|59|LILLE|            : ...........A..........................................................................T.....T..T..T. :  400
U22423.1|PUUV|N|Belgium|M.g.|CG13891|              : ...........A.................G..................................C...........C........T.....T..T..T. :  400
2014.00321|PUUV|N|France|H.s.|59|FOURMIES|         : .................................................................................................... :  400
2015.00457|PUUV|N|France|H.s.|55|REVIGNY-SUR-ORNAI : ...........A.......................G...................................A....................T..T..T. :  400
2013.00250|PUUV|N|France|H.s.|62|VENDIN-LS-BTHUNE| : ...........A...........................................................A.....C..............T.....T. :  400
2016.00282|PUUV|N|France|H.s.|59|MORBECQUE|        : ...........A...........................................................A.....C..............T.....T. :  400
AJ277076.1|PUUV|N|Belgium|M.g.|Montbliart/CG14445| : ...........A................................................................................T..T..T. :  400
2014.00153|PUUV|N|France|H.s.|02|BUIRONFOSSE|      : ...........A................................................................................T..T.... :  400
2014.00171|PUUV|N|France|H.s.|59|SAINT-SAULVE|     : ...........A.................................................................C........T.....T..T..T. :  400
2016.00293|PUUV|N|France|H.s.|60|LA-NEUVILLE-SUR-R : ...........A................................G..............C.................C........T.....T..T..T. :  400
2014.00499|PUUV|N|France|H.s.|94|CHAMPIGNY-SUR-MAR : ............................................................................................T..T.... :  400
2012.00057|PUUV|N|France|H.s.|59|COUSOLRE|         : ...........A................................................................................T..T..T. :  400
2012.00349|PUUV|N|France|H.s.|02|ENGLANCOURT|      : ...........A................................................................................T..T.... :  400
2014.00135|PUUV|N|France|H.s.|02|ATHIES-SOUS-LAON| : ............................................................................................T..T.... :  400
2014.00184|PUUV|N|France|H.s.|59|FOURMIES|         : ...........A................................................................................T..T..T. :  400
2016.00182|PUUV|N|France|H.s.|08|BOULZICOURT|      : ...........A.................G..............G...............................................T..T..T. :  400
2016.00286|PUUV|N|France|H.s.|02|TRUCY|            : ............................................................................................T..T.... :  400
2016.00325|PUUV|N|France|H.s.|59|AULNOYE-AYMERIES| : ...........A................................................................................T..T..T. :  400
2016.00452|PUUV|N|France|H.s.|02|LAON|             : ............................................................................................T..T.... :  400
2016.00467|PUUV|N|France|H.s.|02|AISNE|            : ...........A.........................................T......................................T..T.... :  400
```

```
                                                                   GTCCTTGATGT   (end of PPT334C)    CCIAGTGGICAIACAGC  PPT376C  Bowen MD et al. J Med Virol 1997

                                                       Aitichou M at al J Virol Meth 2005 HANTAV1U     GWGGVCARACAGCWGAYT                  PUU1P  TTCACAATTCCTATCA
                                                                                *         320         *         340         *         360         *         380         *         400
2014.00598|PUUV|N|France|H.s.|59|FOURMIES|         : GTCCTTGATGTGAATGCCATTGATATAGAAGAACCAAGTGGTCAAACAGCAGACTGGTATACAATTGGAGTGTATGTTATAGGCTTCACACTCCCCATCA :  400
2015.00657|PUUV|N|France|H.s.|02|CILLY|            : ......................................................................................T.....T..T..T. :  400
2015.00526|PUUV|N|France|H.s.|08|MONTHERME|        : .................T..........................................................................T..T.... :  400
2015.00045|PUUV|N|France|H.s.|59|FOURMIES|         : ...........A................................................................................T..T..T. :  400
2015.00430|PUUV|N|France|H.s.|02|MONTCORNET|       : ............................................................................................T..T..T. :  400
2016.00311|PUUV|N|France|H.s.|02|LAON|             : ............................................................................................T..T.... :  400
AJ277031.1|PUUV|N|Belgium|M.g.|Montbliart/23Cg/96| : ...........A......................................G.................G.......................T..T..T. :  400
2016.00326|PUUV|N|France|H.s.|02|LAON|             : ............................................................................................T..T.... :  400
2016.00353|PUUV|N|France|H.s.|02|CESSIERES|        : ............................................................................................T..T.... :  400
2012.00061|PUUV|N|France|H.s.|02|LANISCOURT|       : ............................................................................................T..T.... :  400
2014.00174|PUUV|N|France|H.s.|02|ST-ERME-OUTRE-ET- : ............................................................................................T..T.... :  400
2016.00268|PUUV|N|France|H.s.|02|PRESLES-ET-THIERN : ............................................................................................T..T.... :  400
2015.00660|PUUV|N|France|H.s.|02|VIC-SUR-AISNE|    : .......................................................................A..............T.....T..T..T. :  400
2012.00025|PUUV|N|France|H.s.|51|SAINTE-MENEHOULD| : .......................C.....G..G......................................A.....C.....T.....G.....T.... :  400
2015.00665|PUUV|N|France|H.s.|51|REIMS|            : ...........A...........C........G......................................A.....C.....T...........T..T. :  400
2012.00307|PUUV|N|France|H.s.|54|COLOMBEY-LES-BELL : ...........A...........C.....G..G....................T........G........A.....C.....T..T.....T..T.... :  400
2012.00123|PUUV|N|France|H.s.|55|BAR-LE-DUC|       : ...........A...........C.....G..G..C...................................A.....C.....T.....G.....T.... :  400
2012.00278|PUUV|N|France|H.s.|51|REIMS|            : ...........A...........C.....G..G....................T.................A.....C.....T..T..T.....T..T. :  400
2015.00185|PUUV|N|France|H.s.|68|FELLERING|        : .......................C.....G..G....................T.................A.....C.....T.....G.....T.... :  400
KJ994776.1|PUUV|N|Germany|M.g.|Mu/07/1219|2007|    : ................................G..T.................T.................C.....C..T.....T.....T.....T. :  400
2012.00086|PUUV|N|France|H.s|58|CHEVROCHES|        : .................T........T..G.......................T.................C..............T........T..T. :  400
KT247595.1|PUUV|N|France|M.g.|45|Orleans/Mg29/2010 : ..............C...........T...........C..C...........T.................C..............T........T..T. :  400
KT247594.1|PUUV|N|France|M.g.|45|Orleans/Mg23/2010 : ..............C...........T...........C..C...........T.................C..............T........T..T. :  400
KY365004.1|PUUV|N|France|M.g.|45|Orleans/NCHA373/2 : ..........................T...........C..C...........T.................C..............T........T..T. :  400
2012.00301|PUUV|N|France|H.s.|25|MOUTHE|           : .................T.....C.................C...........T..............G..A.....C........T........T..T. :  400
AM695638.1|PUUV|N|France|M.g.|Mignovillard/CgY02/2 : .................T.....C.................C...........T..............G..A.....C........T........T..T. :  400
KT247597.1|PUUV|N|France|M.g.|39|Jura/Mg214/2010|  : .................T.....C.................C...........T..............G..A..............T........T.... :  400
KT247596.2|PUUV|N|France|M.g.|39|Jura/Mg2/2010|    : .................T..C..C.................C...........T..............G..A..............T........T.... :  400
2012.00102|PUUV|N|France|H.s.|39|COISERETTE|       : .................T.....C..............C..C...........T..............G..A.....C........T........T..T. :  400
2012.00536|PUUV|N|France|H.s.|39|LA-PESSE|         : .................T.....C..............C..C...........T..............G..A.....C........T........T..T. :  400
2014.00622|PUUV|N|France|H.s.|39|ARBOIS|           : .................T.....C.................C...........T..............G..A.....C........T........T..T. :  400
2015.00567|PUUV|N|France|H.s.|70|RIOZ|             : .................T.....C.................C...........T.................A.....C........T.....T..T..T. :  400
2014.00637|PUUV|N|France|H.s.|25|SAULES|           : .................T.....C.................C...........T..............G..A.....C..............T..T..T. :  400
2012.00396|PUUV|N|France|H.s.|39|SAINT-CLAUDE|     : .................T.....C.................C...........T..............G..A.....C.................T..T. :  400
2014.00120|PUUV|N|France|H.s.|38|LE-MOUTARET|      : .................T.....C.................C...........T..............G..A.....C........T........T..T. :  400
2015.00153|PUUV|N|France|H.s.|73|GREZY-SUR-ISERE|  : .................T.....C.................C...........T..............G..A.....C........T........T..T. :  400
2015.00504|PUUV|N|France|H.s.|70|RONCHAMP|         : .................T.....C.......................T....................G..A.....C........T........T..T. :  400
2016.00275|PUUV|N|France|H.s.|21|JALLANGES|        : .................T.....C............................................G..A.....C........T.....T..T..T. :  400
2016.00320|PUUV|N|France|H.s.|25|SAINT-VIT|        : .................T.....C.................C...........T..............G..A..............T........T..T. :  400
2015.00410|PUUV|N|France|H.s.|70|ANGIREY|          : .................T.....C.......................T....................G..A.....C........T........T..T. :  400
DQ016430.2|PUUV|N|Germany|M.g.|Bavaria/CG33/04|    : ..............C..T.....C.............................T..............G..T.....C.....T...........T.... :  400
DQ016432.2|PUUV|N|Germany|M.g.|Bavaria/CG41/04|    : .................T.....C...........G.................T..............G..T.....C.....T...........T.... :  400
AY954723.2|PUUV|N|Germany|M.g.|Bavaria-CG34/04|    : .................T.....C...........G.................T..............G..T.....C.....T..T........T.... :  400
AY954722.2|PUUV|N|Germany|M.g.|Bavaria-CG9/04|     : .................T.....C...........G.................T..............G..T.....C.....T...........T.... :  400
2012.00402|PUUV|N|France|H.s.|60|GOUVIEUX|         : .......................C.................C...........T.....C........G..A..............T........T..T. :  400
2014.00540|PUUV|N|France|H.s.|60|CHAMBLY|          : .......................C.................C...........T.....C........G..A..............T........T..T. :  400
AJ314600.1|PUUV|N|Balkan|M.g.|Balkan-1|            : ................................G......................................T.....C..T.....T........G..T. :  400
AJ314601.1|PUUV|N|Balkan|M.g.|Balkan-2|            : ................................G....................T.................T.....C..T.....T........G..T. :  400
FN377821.1|PUUV|N|Hungary|M.g.|Mg9/HungaryTR17/00| : ...........A....................G......................................T.....C..T.....T........G..T. :  400
FN377822.1|PUUV|N|Hungary|M.g.|Mg23/HungaryTR17/00 : ................................G..G...................................T.....C..T.....T........G..T. :  400
GQ339473.1|PUUV|N|Sweden|M.g.|Kiviniemi/Mg3/05|    : .................T.....C........G........C..G........T........T........A.....A.....G........A..T..T. :  400
GQ339474.1|PUUV|N|Sweden|M.g.|Kiviniemi/Mg5/05|    : .....C...........T.....C........G........C..G........T........T........A.....A.....G........A..T..T. :  400
GQ339475.1|PUUV|N|Sweden|M.g.|Kiviniemi/Mg6/05|    : .....C...........T.....C........G........C..G........T........T........A.....A.....G........A..T..T. :  400
GQ339476.1|PUUV|N|Sweden|M.g.|Aijajarvi/Mg7/05|    : .................T.....C........G........C..G........T........T........A.....A.....G........A..T..T. :  400
```

```
                                                     GTCCTTGATGT  (end of PPT334C)    CCIAGTGGICAIACAGC  PPT376C  Bowen MD et al. J Med Virol 1997

                                         Aitichou M at al J Virol Meth 2005 HANTAV1U     GWGGVCARACAGCWGAYT                     PUU1P  TTCACAATTCCTATCA
                                                               *       320         *       340         *       360         *       380         *       400
2014.00598|PUUV|N|France|H.s.|59|FOURMIES|        : GTCCTTGATGTGAATGCCATTGATATAGAAGAACCAAGTGGTCAAACAGCAGACTGGTATACAATTGGAGTGTATGTTATAGGCTTCACACTCCCCATCA :  400
GQ339477.1|PUUV|N|Sweden|M.g.|Aijajarvi/Mg9/05|   : .................T.....C........G........C..G........T........T..C.....A.....A.....G........A..T..T. :  400
GQ339478.1|PUUV|N|Sweden|M.g.|Jockfall/Mg12/05|   : .................T.....C........G........A...........T........C........A.....A.....G........A..T..T. :  400
GQ339482.1|PUUV|N|Sweden|M.g.|Kalvudden/Mg22/05|  : .......................C.................C..G.................T..A.....A.....A.....G........A..T..T. :  400
GQ339479.1|PUUV|N|Sweden|M.g.|Moskosel/Mg17/05|   : .................T..C..C........G...........G........T........T..............A.....G..T.....G..T..T. :  400
GQ339481.1|PUUV|N|Sweden|M.g.|Ljustrask/Mg20/05|  : .................T..C..C........G...........G.................T..............A.....G..T.....G..T..T. :  400
GQ339480.1|PUUV|N|Sweden|M.g.|Gyttjea/Mg19/05|    : .................T.....C........G...........G........T........T..............A.....G..T.....G..T..T. :  400
AY526219.1|PUUV|N|Sweden|Human|Umea/hu|           : ....................C....................C...........T.......................A.....G..T.....A..T..T. :  400
AJ223380.1|PUUV|N|Sweden|M.g.|Tavelsjo/Cg81/94|   : ................................G..G.....C...........T.......................A.....G........G..T..T. :  400
U14137.1|PUUV|N|Bosnia-H.|M.g.|Vranica|           : ................................G..G.....C...........T.......................A.....G........G..T..T. :  400
AJ223371.1|PUUV|N|Sweden|M.g.|Huggberget/Cg36/94| : .....................................................T.......................A.....G..T.....A..T..T. :  400
Z48586.1|PUUV|N|Sweden|M.g.|Vindeln/L20Cg/83|     : ................................G........C........C..........................G.....G........A..T..T. :  400
AJ223374.1|PUUV|N|Sweden|M.g.|Mellansel/Cg47/94|  : .....................................................T.......................G.....G..T.....A..T..T. :  400
AJ223375.1|PUUV|N|Sweden|M.g.|Mellansel/Cg49/94|  : .....................................................T.......................G.....G..T.....A..T..T. :  400
AJ238791.1|PUUV|N|Denmark|M.g.|Fyn/19|            : ...........A........A..C..T.....G........G..G........T........T..A.....A.....G..T..T..T.....T.....T. :  400
AJ278092.1|PUUV|N|Denmark|M.g.|Fyn47|             : ...........A.....T..A..C..T.....G........G..G........T........C..A.....A.....G..T..T..T...........T. :  400
AJ278093.1|PUUV|N|Denmark|M.g.|Fyn131|            : ...........A.....T..A..C..T.....G........G..G........T........C..A.....A.....G..T..T..T...........T. :  400
AJ223368.1|PUUV|N|Norway|M.g.|Eidsvoll/1124v|     : ....................A.....T..G.....G..........................C........C.....G..T.....T........G..T. :  400
AJ223369.1|PUUV|N|Norway|M.g.|Eidsvoll/Cg1138/87| : ..........................T..G.....G..........................C........C.....G..T.....T........G..T. :  400
JN657228.1|PUUV|N|Latvia|M.g.|Jelgava/Mg149/2008| : .................T..C.....T.....G..........................C.....A.....A.....C..T..G..T........T..T. :  400
KX757839.1|PUUV|N|Lithuania|M.g.|LT15/164|2015|   : ...........A.....T........T.....G........C..G....................A..G..A.....G..T..T...........T..T. :  400
KX757840.1|PUUV|N|Lithuania|M.g.|LT15/174|2015|   : ...........A.....T........T.....G........C..G....................A.....A.....G..T..T..T........T..T. :  400
KX815394.1|PUUV|N|Poland|M.g.|KS13/855|2009|      : ...........A.....T.....C..T..G..G........C..G........T...........A..G........G..T..G..T........T..T. :  400
KX757841.1|PUUV|N|Lithuania|M.g.|LT15/201|2015|   : ...........A.....T........T.....G........C..G....................A..G..A.....G..T..T...........T..T. :  400
AJ314598.1|PUUV|N|Russia|M.g.|Baltic/49Cg/00|     : ...........A.....T.....C..T.....G....................T........T..A...........G.....G..T.....T..T.... :  400
AJ314599.1|PUUV|N|Russia|M.g.|Baltic/205Cg/00|    : ...........A.....T.....C..T.....G....................T.....C..T..A.....C.....G.....A..T.....T..T.... :  400
JN657229.1|PUUV|N|Latvia|M.g.|Madona/Mg99/2008|   : ...........A..............T........G..........................T..A..G..T.....G...A.T..T...T.A....... :  400
JN657232.1|PUUV|N|Latvia|M.g.|Madona/Mg233/2008|  : ...........A..............T........G..........................T..A..G..T.....G...A.T..T...T.A....... :  400
JN657230.1|PUUV|N|Latvia|M.g.|Jelgava/Mg136/2008| : ..T........A..............T..G..G.............................C..A..G..C.....G.....T..T.....A....... :  400
JN657231.1|PUUV|N|Latvia|M.g.|Jelgava/Mg140/2008| : ..T........A..............T..G..G.............................C..A..G..C.....G.....T..T.....A....... :  400
Z30707.1|PUUV|N|Russia|M.g.|Udmurtia/458Cg/88|    : ...........A...........C..C.................G........T........T........C.....A.....T..T...A.T....... :  400
Z30706.1|PUUV|N|Russia|M.g.|Udmurtia/444Cg/88|    : .......................C..C.................G........T........T........C.....G.....T..T...A.T....... :  400
Z84204.1|PUUV|N|Russia|M.g.|Kazan|                : ...........A...........C..C.................G........T........T........C.....A.....T..T...A.T....... :  400
Z30708.1|PUUV|N|Russia|M.g.|Udmurtia/338Cg/92|    : ...........A...........C..C.................G........T........T........C.....A.....T..T...A.T....... :  400
Z21497.1|PUUV|N|Russia|M.g.|Udmurtia/894Cg/91|    : ...........A...........C..C.................G........T........T........C.....A.....T..T...A.T..T.... :  400
AB433843.2|PUUV|N|Russia|M.g.|Samara_49/CG/2005|  : ...........A...........C..T..G..............G........T........T........C.....G.....G......A.T....... :  400
AB433845.2|PUUV|N|Russia|M.g.|Samara_94/CG/2005|  : ...........A...........C..T..G..............G........T........T........C.....G.....G..T...A.T....... :  400
L11347.1|PUUV|N|Russia|Human|P360|                : .................T.....C..T..............C..G........T........T..C.....C.....A.....G......A.T..T.... :  400
AB297665.2|PUUV|N|Russia|M.g.|DTK/Ufa-97|1997|    : .................T.....C..T..............C..G........T........T..C.....C.....A.....G......A.T..T.... :  400
M32750.1|PUUV|N|Russia|M.g.|CG1820|               : .................T.....C..T..............C..G........T........T..C.....C.....A.....G......A.T..T.... :  400
AF442613.1|PUUV|N|Russia|M.g.|CG17/Baskiria-2001| : .................T.....C..T..............C..G........T........T..C.....C.....A.....G......A.T..T.... :  400
KX815395.1|PUUV|N|Poland|M.g.|KS14/118|2009|      : ...........A.....A........T........G.....C.................C..C..A..G..C.....A.....G......A.T.....T. :  400
GQ339483.1|PUUV|N|Sweden|M.g.|Bergsjobo/Mg25/05|  : ..........................T..............C.....T.....T........T........T.....G........T........T..T. :  400
AJ223377.1|PUUV|N|Sweden|M.g.|Solleftea/Cg6/95|   : ..........................T..............C.....T.....T........T........T.....G........T........T..T. :  400
GQ339484.1|PUUV|N|Sweden|M.g.|Faboviken/Mg26/05|  : ..........................T.....G..G.....C.....T.....T........T........C.....A........T........T..T. :  400
GQ339485.1|PUUV|N|Sweden|M.g.|Mangelbo/Mg1/05|    : .................T........T.....G..............T.....T........T........T.....G........T........T..T. :  400
GQ339486.1|PUUV|N|Sweden|M.g.|Munga/Mg2/05|       : .........A.......T........T.....G..............T.....T........T........T.....G........T........T..T. :  400
GQ339487.1|PUUV|N|Sweden|M.g.|Munga/Mg16/05|      : .................T........T.....G..............T.....T........T........T.....G........T........T..T. :  400
AJ223376.1|PUUV|N|Sweden|M.g.|Solleftea/Cg3/95|   : ..........................T..............C.....T.....T........T........C.....G........T........T..T. :  400
AF367071.1|PUUV|N|Russia|M.r.|CRF366|             : .................A........T..G........C..C..G........T........C..A...A.T.....G........T.....T....... :  400
AF367064.1|PUUV|N|Russia|M.g.|CG144|              : .................A........T..G........C..C..G........T........C..A...A.T.....G........T.....T....... :  400
AF367065.1|PUUV|N|Russia|M.g.|CG168|              : .................A........T..G........C..C..G.................C..A.....T.....A........T.....T....... :  400
AF367068.1|PUUV|N|Russia|M.g.|CG315|              : .................A........T..G........C..C..G........T........C..A.....T.....G........T.....T....... :  400
AF367069.1|PUUV|N|Russia|M.r.|CRF161|             : .................A........T..G........C..C..G.................C..A.....T.....A........T.....T....... :  400
```

```
                                                             GTCCTTGATGT   (end of PPT334C)    CCIAGTGGICAIACAGC  PPT376C  Bowen MD et al. J Med Virol 1997

                                              Aitichou M at al J Virol Meth 2005 HANTAV1U     GWGGVCARACAGCWGAYT                      PUU1P  TTCACAATTCCTATCA
                                                                      *        320         *        340         *        360         *        380         *        400
2014.00598|PUUV|N|France|H.s.|59|FOURMIES|         : GTCCTTGATGTGAATGCCATTGATATAGAAGAACCAAGTGGTCAAACAGCAGACTGGTATACAATTGGAGTGTATGTTATAGGCTTCACACTCCCCATCA :  400
AF367070.1|PUUV|N|Russia|M.r.|CRF308|              : .................A........T..G........C..C..G........T........C..A.....T.....G........T.....T....... :  400
AF367066.1|PUUV|N|Russia|M.g.|CG215|               : .................A........T..G........C..C..G........T........C..A.....T.....G........T.....T....... :  400
AF367067.1|PUUV|N|Russia|M.g.|CG222|               : .................A........T..G........C..C..G........T........C..A.....T.....G........T.....T....... :  400
Z46942.1|PUUV|N|Finland|M.g.|Puumala/1324Cg/79|    : .................A..C.....T..............C...........T.................T.....G.....GC.T.....T..T.... :  400
Z30702.1|PUUV|N|Finland|M.g.|Evo/12Cg/93|          : .................A........T..G.......................T.................T.....A.....A......A....T.... :  400
Z30703.1|PUUV|N|Finland|M.g.|Evo/13Cg/93|          : .................A........T..G.......................T.................T.....A.....A......A....T.... :  400
Z30704.1|PUUV|N|Finland|M.g.|Evo/14Cg/93|          : .................A........T..G.......................T.................T.....A.....A......A....T.... :  400
Z30705.1|PUUV|N|Finland|M.g.|Evo/15Cg/93|          : ...T.C...........A........T..G.......................T.................T.....A.....A......A....T.... :  400
Z69985.1|PUUV|N|Finland|M.g.|Virrat/25Cg/95|       : .................A........T..G...........C...........T.................T.....G..G..A........T..T.... :  400
JN831947.1|PUUV|N|Finland|Pieksamaki/human_lung/20 : ..............C..A........T..G...........C.............................T.....G.....A........T..T.... :  400
JN831943.1|PUUV|N|Finland|Pieksamaki/Mg7/2008|     : ..............C..A........T..G...........C.............................T.....G.....A........T..T.... :  400
JN831950.1|PUUV|N|Finland|Pieksamaki/human_kidney/ : ..............C..A........T..G...........C.............................T.....G.....A........T..T.... :  400
JQ319166.1|PUUV|N|Finland|Konnevesi/Mg_O14B/2005|  : .................A........T..G...........C..G........T.................T.....G.....A...........T.... :  400
JQ319169.1|PUUV|N|Finland|Konnevesi/Mg_O27B/2005|  : .................A........T..G...........C..G........T.................T.....G.....A...........T.... :  400
JQ319167.1|PUUV|N|Finland|Konnevesi/Mg_O15B/2005|  : .................A........T..............C..G........T.................T.....G.....A...........T.... :  400
JQ319164.1|PUUV|N|Finland|Konnevesi/Mg_O6B/2005|   : .................A........T..G...........C..G........T.................T.....G.....A...........T.... :  400
JQ319165.1|PUUV|N|Finland|Konnevesi/Mg_O9B/2005|   : .................A........T..G...........C..G........T.................T.....G.....A...........T.... :  400
JQ319170.1|PUUV|N|Finland|Konnevesi/Mg_O74B/2005|  : .................A........T..G...........C..G........T.................T.....G.....A...........T.... :  400
JQ319171.1|PUUV|N|Finland|Konnevesi/Mg_M114B/2005| : .................A........T..............C..G........T.................T.....G.....A...........T.... :  400
AJ238788.1|PUUV|N|Russia|M.g.|Karhumaki|           : .....C...........A........T..G...........C...........T...........C.....T.....A.....A........T..T..T. :  400
AJ238789.1|PUUV|N|Russia|M.g.|Kolodozero|          : .................A........T..G.....G.....C...........T.................C.....A.....A..T.....T.....T. :  400
AJ314597.1|PUUV|N|Finland|M.g.|Pallasjarvi/63Cg/98 : .................A........T..G...........C....................G........T.....A.....A........T.....T. :  400
NC_005224.1|PUUV|N|Finland|M.g.|Sotkamo-revu-NCBI2 : ...........A.....A.....C..T..............C...........T.......................G.....G........T..T.... :  400
AJ238790.1|PUUV|N|Russia|M.g.|Gomselga|            : .....C...........A........T..G..C........C...........T...........C.....T.....A.....G........T..T.... :  400
AB010731.1|Hokkaido-V|N|Japan|M.r.|Tobetsu-60Cr-93 : .................A.....C........G..T.................T........C..A.....A...A.A.....G..T.....A..T..T. :  400
AB010730.1|Hokkaido-V|N|Japan|M.r.|Kamiiso-8Cr-95| : .................A.....C........G..T..........................C..A.....A...A.A.....G..T.....A..T..T. :  400
JX046487.1|Muju-V|N|South-Korea|M.r.|11-5|2011     : .................A..A..C.................C..........................G..A..CA.C..T..G..T...T.A..G..TG :  400
JX046484.1|Muju-V|N|South-Korea|M.r.|11-4|2011|    : .................A..A..C.................C..........................G..A..CA.C..T..G......T.A..G..TG :  400
JX028273.1|Muju-V|N|South-Korea|M.r.|11-1|2011|    : .................A..A..C.................C..........................G..A..CA.C..T..G..T...T.A..G..TG :  400
DQ138128.1|Muju-V|N|South-Korea|E.r.|00-18|        : .................A..A................................T..............T........C..T..G..T.....G..A..TG :  400
DQ138140.1|Muju-V|N|South-Korea|E.r.|99-27|        : .................A..A.......................G........T..............T........C..T..G..T.....G..A..TG :  400
DQ138133.1|Muju-V|N|South-Korea|E.r.|96-1|         : .................A..A................................T..............T........C..C..G..T.....G..A..TG :  400
DQ138142.1|Muju-V|N|South-Korea|E.r.|99-28|        : .................A..A.......................G........T..............T........C..T..G..T.....G..A..TG :  400
```

```
                                                            Korva M et al. Microbes Infect 2009  PUUL ACATCATTTGAGGA

                                          TTTTGAAGGC  (PUU1P)  Aitichou M at al J Virol Meth 2005
                                                   *       420         *       440         *       460         *       480         *       500
2014.00598|PUUV|N|France|H.s.|59|FOURMIES|         : TCCTGAAGGCTTTATATATGTTGTCAACACGTGGGAGACAAACTGTGAAGGAAAACAAAGGgACTCGTATCCGTTTTAAAGATGACACTTCCTTTGAGGA :  500
2014.00233|PUUV|N|France|H.s.|59|FOURMIES|         : .......................................................T............................................ :  500
2014.00097|PUUV|N|France|H.s.|02|SAINT-MICHEL|     : ...........................................A........................................................ :  500
2014.00488|PUUV|N|France|H.s.|08|SIGNY-LE-PETIT|   : .................................................................................................... :  500
2014.00613|PUUV|N|France|H.s.|59|FOURMIES|         : .................................................................................................... :  500
2015.00402|PUUV|N|France|H.s.|08|CHARLEVILLE-MEZIE : ..........C..G...................................A.................................................. :  500
2015.00422|PUUV|N|France|H.s.|59|ANOR|             : .................................................................................................... :  500
2015.00488|PUUV|N|France|H.s.|08|SEDAN|            : ..........C......................................A.........................................T........ :  500
2015.00498|PUUV|N|France|H.s.|08|REMILLY-AILLICOUR : .................................................................................................... :  500
2016.00295|PUUV|N|France|H.s.|08|CORNY-MACHEROMENI : .................................................................................................... :  500
2016.00333|PUUV|N|France|H.s.|59|FOURMIES|         : .................................................................................................... :  500
2016.00345|PUUV|N|France|H.s.|59|FOURMIES|         : .................................................................................................... :  500
2016.00357|PUUV|N|France|H.s.|02|HIRSON|           : .................................................................................................... :  500
2016.00427|PUUV|N|France|H.s.|59|FOURMIES|         : .................................................................................................... :  500
2016.00469|PUUV|N|France|H.s.|02|PREMONTRE|        : ....A.........................................A..A..G......................................T........ :  500
AJ277075.1|PUUV|N|Belgium|M.g.|Montbliart/CG14444| : ...........C.....................................A............................................C..... :  500
2014.00053|PUUV|N|France|H.s.|08|SECHEVAL|         : ..........C......................................A.........................................T........ :  500
KT247593.1|PUUV|N|France|M.g.|08|Ardennes/Mg75/201 : .................................................A..........................C.................C..... :  500
KT247592.1|PUUV|N|France|M.g.|08|Ardennes/Mg156/20 : .................................................A..........................C.................C..... :  500
2015.00019|PUUV|N|France|H.s.|08|ETEIGNIERES|      : ........................................G........A...........A..............C....................... :  500
2016.00239|PUUV|N|France|H.s.|08|VIREUX-MOLHAIN|   : ........................................G........A...........A................................C..... :  500
2015.00329|PUUV|N|France|H.s.|08|BOGNY-SUR-MEUSE|  : ........................................G........A...........A..............C.................C..... :  500
2014.00209|PUUV|N|France|H.s.|08|TREMBLOIS-LES-ROC : ........................................G........A...........A..............C.................C..... :  500
2015.00456|PUUV|N|France|H.s.|94|ALFORTVILLE|      : ........................................G........A...........A..............C.................C..A.. :  500
2012.00018|PUUV|N|France|H.s.|08|ROCROI|           : ..T.....................................G........A...........A..............C.................C..... :  500
AJ277032.1|PUUV|N|Belgium|M.g.|Momignies/47Cg/96|  : ........................................G........A..........................C.................C..... :  500
AJ277034.1|PUUV|N|Belgium|M.g.|Couvin/59Cg/97|     : ........................................G........A...........A..............C.................C..... :  500
AJ277033.1|PUUV|N|Belgium|M.g.|Momignies/55Cg/96|  : ........................................G........A..........................C.................C..... :  500
2012.00638|PUUV|N|France|H.s.|08|GIVET|            : .................................................A..........................C.................C..... :  500
2015.00328|PUUV|N|France|H.s.|08|VRIGNE-MEUSE|     : .................................................A..........................C.................C..... :  500
2015.00419|PUUV|N|France|H.s.|08|DOUZY|            : .................................................A..........................C....................... :  500
2016.00310|PUUV|N|France|H.s.|08|THIN-LE-MOUTIER|  : ....................................................G............................................... :  500
AJ277030.1|PUUV|N|Belgium|M.g.|Thuin/33Cg/96|      : .................................................A..........................C.................C..... :  500
AJ238779.1|PUUV|N|Germany|M.g.|NRW/Cg-Erft|        : ...........C.G...........G.......................A..G....................C..C........T..A........A.. :  500
2014.00276|PUUV|N|France|H.s.|59|LILLE|            : .................................................A..........................C..G.....T.....T........ :  500
U22423.1|PUUV|N|Belgium|M.g.|CG13891|              : ..........C..G...................................A...........A.....C..T.....C..............T........ :  500
2014.00321|PUUV|N|France|H.s.|59|FOURMIES|         : .T.................................................................................................. :  500
2015.00457|PUUV|N|France|H.s.|55|REVIGNY-SUR-ORNAI : ....A...............C.A.................G.....A..A.........................................T........ :  500
2013.00250|PUUV|N|France|H.s.|62|VENDIN-LS-BTHUNE| : ..T.................C............................A....................T....................T........ :  500
2016.00282|PUUV|N|France|H.s.|59|MORBECQUE|        : ..T..............................................A....................T.....C..............T........ :  500
AJ277076.1|PUUV|N|Belgium|M.g.|Montbliart/CG14445| : ...........C.....................................A............................................C..... :  500
2014.00153|PUUV|N|France|H.s.|02|BUIRONFOSSE|      : ..........CC.....................................A............................................C..... :  500
2014.00171|PUUV|N|France|H.s.|59|SAINT-SAULVE|     : ..T......................G....................T..A....................T.....C........T.....T........ :  500
2016.00293|PUUV|N|France|H.s.|60|LA-NEUVILLE-SUR-R : .T............................................A..A..........................C........T.....T........ :  500
2014.00499|PUUV|N|France|H.s.|94|CHAMPIGNY-SUR-MAR : ...........C.....................................A.................C..........................C..... :  500
2012.00057|PUUV|N|France|H.s.|59|COUSOLRE|         : ...........C.....................................A............................................C..... :  500
2012.00349|PUUV|N|France|H.s.|02|ENGLANCOURT|      : ...........C.....................................A............................................C..... :  500
2014.00135|PUUV|N|France|H.s.|02|ATHIES-SOUS-LAON| : ...........C.....................................A.................C.......................T..C..... :  500
2014.00184|PUUV|N|France|H.s.|59|FOURMIES|         : ...........C.....................................A............................................C..... :  500
2016.00182|PUUV|N|France|H.s.|08|BOULZICOURT|      : ...........C.....................................A............................................C..... :  500
2016.00286|PUUV|N|France|H.s.|02|TRUCY|            : ...........C.....................................A.................C..........................C..... :  500
2016.00325|PUUV|N|France|H.s.|59|AULNOYE-AYMERIES| : ...........C.....................................A............................................C..... :  500
2016.00452|PUUV|N|France|H.s.|02|LAON|             : ...........C.....................................A.................C.......................T..C..... :  500
2016.00467|PUUV|N|France|H.s.|02|AISNE|            : ...........C.....................................A............................................C..... :  500
```

```
                                                                                                                         Korva M et al. Microbes Infect 2009  PUUL ACATCATTTGAGGA

                                                               TTTTGAAGGC  (PUU1P)  Aitichou M at al J Virol Meth 2005
                                                                        *       420         *       440         *       460         *       480         *       500
2014.00598|PUUV|N|France|H.s.|59|FOURMIES|        : TCCTGAAGGCTTTATATATGTTGTCAACACGTGGGAGACAAACTGTGAAGGAAAACAAAGGgACTCGTATCCGTTTTAAAGATGACACTTCCTTTGAGGA :  500
2015.00657|PUUV|N|France|H.s.|02|CILLY|           : ......................A..G.......................A..G.................T.....C........T.............. :  500
2015.00526|PUUV|N|France|H.s.|08|MONTHERME|       : ...........C......................A..............A.................................................. :  500
2015.00045|PUUV|N|France|H.s.|59|FOURMIES|        : ...........C.....................................A.............................................C..... :  500
2015.00430|PUUV|N|France|H.s.|02|MONTCORNET|      : ...........C..........A..........................A..............A................................... :  500
2016.00311|PUUV|N|France|H.s.|02|LAON|            : ...........C.C...................................A.................C...........................C..... :  500
AJ277031.1|PUUV|N|Belgium|M.g.|Montbliart/23Cg/96| : ...........C.....................................A....................................T.............. :  500
2016.00326|PUUV|N|France|H.s.|02|LAON|            : ...........C.....................................A.................C...........................C..... :  500
2016.00353|PUUV|N|France|H.s.|02|CESSIERES|       : ...........C.....................................A.................C...........................C..... :  500
2012.00061|PUUV|N|France|H.s.|02|LANISCOURT|      : ...........C.....................................A.................C...........................C..... :  500
2014.00174|PUUV|N|France|H.s.|02|ST-ERME-OUTRE-ET- : ...........C.....................................A.................C...........................C..... :  500
2016.00268|PUUV|N|France|H.s.|02|PRESLES-ET-THIERN : ...........C.....................................A.................C...........................C..... :  500
2015.00660|PUUV|N|France|H.s.|02|VIC-SUR-AISNE|   : .............G....................A...........A..A...........A..............C........T.....T........ :  500
2012.00025|PUUV|N|France|H.s.|51|SAINTE-MENEHOULD| : ..T.A.....CC....C.................A..............A.....T.....A..A.....T..A...........T..A..T........ :  500
2015.00665|PUUV|N|France|H.s.|51|REIMS|           : .TT.A.....CC....C.................A..G...........A.....T.....A..A.....T..A...........T..A..T........ :  500
2012.00307|PUUV|N|France|H.s.|54|COLOMBEY-LES-BELL : .T........C.........C.A...........A..G..G........A.....T..............T..A...........T.............. :  500
2012.00123|PUUV|N|France|H.s.|55|BAR-LE-DUC|      : ..T.A.....CC....C.................A..............A..G..T.....A..A.....T..A...........T..A..T........ :  500
2012.00278|PUUV|N|France|H.s.|51|REIMS|           : .TT.A.....CC.G..C.................A..............A..G..T........A.....T..A...........T..A..T..C..... :  500
2015.00185|PUUV|N|France|H.s.|68|FELLERING|       : ..T.A.....CC....C.................A..............A.....T.....A..A.....T..A.....G.....T..A........... :  500
KJ994776.1|PUUV|N|Germany|M.g.|Mu/07/1219|2007|   : .......A...C..........A.................G.....A........T........A.....T..G.....G.....T.....A........ :  500
2012.00086|PUUV|N|France|H.s|58|CHEVROCHES|       : ....A.....C..G......C.T........A.....G........A..A........g..a.....c..T..A...........T..A..T.....A.. :  500
KT247595.1|PUUV|N|France|M.g.|45|Orleans/Mg29/2010 : ....A.....CC.G..C...C.T........A.....G...........A........G..A........T..A...........T..A..T.....A.. :  500
KT247594.1|PUUV|N|France|M.g.|45|Orleans/Mg23/2010 : ....A.....CC.G..C...C.T........A.....G...........A........G..A........T..A...........T..A..T.....A.. :  500
KY365004.1|PUUV|N|France|M.g.|45|Orleans/NCHA373/2 : ....A..A..CC.G......C.T........A.....G...........A........G..A........T..A...........T..A..T.....A.. :  500
2012.00301|PUUV|N|France|H.s.|25|MOUTHE|          : ..T.A.....C.....C.....A.................G.....A........T..G..A..C..C..T..A.....G...........A........ :  500
AM695638.1|PUUV|N|France|M.g.|Mignovillard/CgY02/2 : ..T.A.....C.....C.....A.................G.....A........T..G..A..C..C..T..A.....G...........A........ :  500
KT247597.1|PUUV|N|France|M.g.|39|Jura/Mg214/2010| : ..T.......C.....C.....A.................G.....A........T..G..A..C..C..T..A.....G...........A........ :  500
KT247596.2|PUUV|N|France|M.g.|39|Jura/Mg2/2010|   : ..T.......C.....C.....A.................G.....A........T..G..A..C..C..T..A.....G...........A........ :  500
2012.00102|PUUV|N|France|H.s.|39|COISERETTE|      : ..T.A.....C..G..C.....A.................G..C..A........T..G..a..c..C..T..A.....G...........A........ :  500
2012.00536|PUUV|N|France|H.s.|39|LA-PESSE|        : ..T.A.....C..G..C.....A.................G..C..A........T..G..A..C..C..T..A.....G...........A........ :  500
2014.00622|PUUV|N|France|H.s.|39|ARBOIS|          : ..T.A.....C.....C.....A.................G..............T..G..A..C..C..T..A.....G...........A........ :  500
2015.00567|PUUV|N|France|H.s.|70|RIOZ|            : ..T.A.....C.....C.....A.......................A........T..G..A..C..C..T..A.....G...........A........ :  500
2014.00637|PUUV|N|France|H.s.|25|SAULES|          : ..T.A.....C.....C.....A.......................A........T..G..A..c..C..T..A.....g...........A........ :  500
2012.00396|PUUV|N|France|H.s.|39|SAINT-CLAUDE|    : ..T.A.....C..G..C.....A.................G.....A........T..G..a..C.....T..A.....G...........A........ :  500
2014.00120|PUUV|N|France|H.s.|38|LE-MOUTARET|     : ..T.A.....C.....C.....A.................G.....A........T..G..A..C.....T..A.....G...........A........ :  500
2015.00153|PUUV|N|France|H.s.|73|GREZY-SUR-ISERE| : ..T.A.....C.....C.....A.................G.....A........T..G..A..C.....T..A.....G...........A........ :  500
2015.00504|PUUV|N|France|H.s.|70|RONCHAMP|        : .T........A.....C.....A...........A.....G.....A..A..G..T..G.....C..A..T..A.....G...........A........ :  500
2016.00275|PUUV|N|France|H.s.|21|JALLANGES|       : ..T.A.....C.....C.....A.................G.....A........T..G..A..C..C..T..A.................A........ :  500
2016.00320|PUUV|N|France|H.s.|25|SAINT-VIT|       : ..T.......C.....C.....A.................G.....A........T..G..A..C..C..T..A.....g...........A........ :  500
2015.00410|PUUV|N|France|H.s.|70|ANGIREY|         : .T..A.....A.....C.....A...........A.....G.....A..A..G..T..G.....C..A..T..A.....G...........A........ :  500
DQ016430.2|PUUV|N|Germany|M.g.|Bavaria/CG33/04|   : ..T.......C..G........A.....C...........G...........G..............A..T..G...........T..C..A........ :  500
DQ016432.2|PUUV|N|Germany|M.g.|Bavaria/CG41/04|   : ..T.A.....C..G........A.....C...........G...........G..............C.....G...........T.....A........ :  500
AY954723.2|PUUV|N|Germany|M.g.|Bavaria-CG34/04|   : ..T.......C..G........A.....C...........G...........G..T...........C.....G...........T.....A........ :  500
AY954722.2|PUUV|N|Germany|M.g.|Bavaria-CG9/04|    : ..T.......C..G........A.....C...........G...........G..............C.....G...........T.....A........ :  500
2012.00402|PUUV|N|France|H.s.|60|GOUVIEUX|        : ....A.....C.....C.....A.................G..............T...........C..TA.G...........t.....a........ :  500
2014.00540|PUUV|N|France|H.s.|60|CHAMBLY|         : ....A.....C.....C...C.A.................G..............T...........C..TA.G...........T.....A........ :  500
AJ314600.1|PUUV|N|Balkan|M.g.|Balkan-1|           : .A........AC.C........A........A..A.....G........A.....T.....A..A..C..A........G.....T..A..A........ :  500
AJ314601.1|PUUV|N|Balkan|M.g.|Balkan-2|           : .T........AC.C........A...........A.....G.....A..A.....T.....A..A..C..A........G.....T..A..A........ :  500
FN377821.1|PUUV|N|Hungary|M.g.|Mg9/HungaryTR17/00| : .A........AC.C........A........A..A.....G..............T.....A..A..C..A.....C..G.....T..A..A........ :  500
FN377822.1|PUUV|N|Hungary|M.g.|Mg23/HungaryTR17/00 : .A........AC.T........A........A..A..G..G.....A..A.....T.....A..A..C..A........G.....T..A..A........ :  500
GQ339473.1|PUUV|N|Sweden|M.g.|Kiviniemi/Mg3/05|   : .TT.A.....CC.T..C...C.A...........A..G........T.....G........A..A..C.....C..C........T.....A........ :  500
GQ339474.1|PUUV|N|Sweden|M.g.|Kiviniemi/Mg5/05|   : .TT.A.....CC.T..C...C.A...........A..G........T.....G........A..A..C.....C..C..............A........ :  500
GQ339475.1|PUUV|N|Sweden|M.g.|Kiviniemi/Mg6/05|   : .TT.A.....CC.T..C...C.A...........A..G........T.....G........A..A..C.....C..C..............A........ :  500
GQ339476.1|PUUV|N|Sweden|M.g.|Aijajarvi/Mg7/05|   : .TT.A.....CC.T..C...C.A...........A..G........T.....G........A..A..C.....C..C........T.....A........ :  500
```

```
                                                                                                    Korva M et al. Microbes Infect 2009  PUUL ACATCATTTGAGGA

                                                           TTTTGAAGGC  (PUU1P)  Aitichou M at al J Virol Meth 2005
                                                                     *        420         *        440         *        460         *        480         *        500
2014.00598|PUUV|N|France|H.s.|59|FOURMIES|          : TCCTGAAGGCTTTATATATGTTGTCAACACGTGGGAGACAAACTGTGAAGGAAAACAAAGGgACTCGTATCCGTTTTAAAGATGACACTTCCTTTGAGGA :  500
GQ339477.1|PUUV|N|Sweden|M.g.|Aijajarvi/Mg9/05|     : .TT.A.....CC.T..C...C.A...........A..G........T.....G........A..A..C.....C..C........T.....A........ :  500
GQ339478.1|PUUV|N|Sweden|M.g.|Jockfall/Mg12/05|     : .TT.......CC.T..C...C.A..............G........T.....G........A..A........C.................A........ :  500
GQ339482.1|PUUV|N|Sweden|M.g.|Kalvudden/Mg22/05|    : ..T.......CC.T..C...C.A..............G........T..A..G...........A........C.................A........ :  500
GQ339479.1|PUUV|N|Sweden|M.g.|Moskosel/Mg17/05|     : .TT.......CC.T..C...C.T..............G..G.....T..A..G...........A.....T..C.................A........ :  500
GQ339481.1|PUUV|N|Sweden|M.g.|Ljustrask/Mg20/05|    : .TT.......CC.T..C...C.T..............G........T.....G...........A.....T..C..C.....C.....C..A..C..... :  500
GQ339480.1|PUUV|N|Sweden|M.g.|Gyttjea/Mg19/05|      : ..T.......CC.T..C...C.T..............G........T.....G...........A.....T..C..C...............A........ :  500
AY526219.1|PUUV|N|Sweden|Human|Umea/hu|             : .TT.......C.....C...C.T..C.....................T.....G..T........A........C.....G...........A.....A.. :  500
AJ223380.1|PUUV|N|Sweden|M.g.|Tavelsjo/Cg81/94|     : .TT....A..CC....C...C.T..C.....C....................G..T........A......A.A.....G.....T.....A........ :  500
U14137.1|PUUV|N|Bosnia-H.|M.g.|Vranica|             : .TT....A..CC....C...C.T..C.....C....................G..T........A..GTAT..A.....G.....T.....A........ :  500
AJ223371.1|PUUV|N|Sweden|M.g.|Huggberget/Cg36/94|   : .AT.......CC....C...C.T..T..........................G..T........A......A.A.....G.....T.....A........ :  500
Z48586.1|PUUV|N|Sweden|M.g.|Vindeln/L20Cg/83|       : .TT....A..CC..........C.T..C........................G..T........A......A.G.....G.....T..C..A........ :  500
AJ223374.1|PUUV|N|Sweden|M.g.|Mellansel/Cg47/94|    : .T........C.....C...C.T..C........A...........T.....G..T........A........C.....G.....T.....A..C..A.. :  500
AJ223375.1|PUUV|N|Sweden|M.g.|Mellansel/Cg49/94|    : .T........C.....C...C.T..C........A...........T.....G..T........A........C.....G....G......A..C..A.. :  500
AJ238791.1|PUUV|N|Denmark|M.g.|Fyn/19|              : .AT.A......C.G........A......A.G..A.....G........A..G..T.....A..A.....A..A...............A........A.. :  500
AJ278092.1|PUUV|N|Denmark|M.g.|Fyn47|               : .AT.A......C.G..C.....A..T..CA.G..A.....G..G........G..T........A.....A..G..C........T..A..T.....A.. :  500
AJ278093.1|PUUV|N|Denmark|M.g.|Fyn131|              : .AT.A......C.G..C.....A......A.G..A.....G..G........G..T........A.....A..G..C........T..A..T.....A.. :  500
AJ223368.1|PUUV|N|Norway|M.g.|Eidsvoll/1124v|       : ..T.A.....AC.T..C...C.A...........A.....G..............T........AA.A..T..G.....G.....T..A..A........ :  500
AJ223369.1|PUUV|N|Norway|M.g.|Eidsvoll/Cg1138/87|   : ..T.A.....AC.T..C...C.A...........A.....G..............T........AA.A..T..G...........T..A..A........ :  500
JN657228.1|PUUV|N|Latvia|M.g.|Jelgava/Mg149/2008|   : .TT.A.....A.....C...C.T..............G.....A........G..T........A..G..AA.A..C...........A..A........ :  500
KX757839.1|PUUV|N|Lithuania|M.g.|LT15/164|2015|     : .T..A.....CC.G..C...C.T....................A...........T........A..G..AA.G..C..G........A..A........ :  500
KX757840.1|PUUV|N|Lithuania|M.g.|LT15/174|2015|     : .T..A.....C..G..C...C.T....................A.....A.....T.....A..A..G..TA.G.....G........A..A........ :  500
KX815394.1|PUUV|N|Poland|M.g.|KS13/855|2009|        : ....A.....C.....C...C.T.................G..A..A........T.....A..A..A..A..A.....G.....T..A..A........ :  500
KX757841.1|PUUV|N|Lithuania|M.g.|LT15/201|2015|     : .T..A.....CC.G..C...C.T....................A...........T........A..G..AA.G..C..G........A..A........ :  500
AJ314598.1|PUUV|N|Russia|M.g.|Baltic/49Cg/00|       : ....A.....A..............G...........G..G.................G.....AA.A..AA.A.....G........A..A........ :  500
AJ314599.1|PUUV|N|Russia|M.g.|Baltic/205Cg/00|      : ....T.....A.......................A..G..G.....A..............A..AA.G..AA.A.....G.....T..A..A........ :  500
JN657229.1|PUUV|N|Latvia|M.g.|Madona/Mg99/2008|     : ....A.....A..G..C....................G........A..................AA.G..AA.G.....G.....T..A..T..C..... :  500
JN657232.1|PUUV|N|Latvia|M.g.|Madona/Mg233/2008|    : ....A.....A..G..C....................G........A..................AA.G..AA.G.....G.....T..A..T..C..... :  500
JN657230.1|PUUV|N|Latvia|M.g.|Jelgava/Mg136/2008|   : ....A.....A..G..C....................G........A..A.....T........AA.G..AA.A...........T..A.....C..... :  500
JN657231.1|PUUV|N|Latvia|M.g.|Jelgava/Mg140/2008|   : ....A.....A..G..C....................G........A..A.....T........AA.G..AA.A...........T..A.....C..... :  500
Z30707.1|PUUV|N|Russia|M.g.|Udmurtia/458Cg/88|      : .TT.A......C....C.................A.....G..C..A..A.....T..G..A..A..G...A.G..C...........A..G........ :  500
Z30706.1|PUUV|N|Russia|M.g.|Udmurtia/444Cg/88|      : .TT.A...........C.................A.....G..C..A..A.....T.....A..A..G...A.G..C...........A..G........ :  500
Z84204.1|PUUV|N|Russia|M.g.|Kazan|                  : .TT.A......C....C.................A.....G..C..A..A.....T.....A..A..G...A.G..C...........A..G........ :  500
Z30708.1|PUUV|N|Russia|M.g.|Udmurtia/338Cg/92|      : .TT.A......C....C.................A.....G..CA.A..A.....T..G..A..A..G...A.G..C...........A..G........ :  500
Z21497.1|PUUV|N|Russia|M.g.|Udmurtia/894Cg/91|      : .TT........C......................A.....G..C..A..A.....T.....A..A..G...A.G..C...........A..A........ :  500
AB433843.2|PUUV|N|Russia|M.g.|Samara_49/CG/2005|    : .TT.A...........C.................A........C..A..A.....T.....A..A..G...A.G..C..G........A..A........ :  500
AB433845.2|PUUV|N|Russia|M.g.|Samara_94/CG/2005|    : .TT.A...........C...C.............A.....G..C..A..A.....T.....A..A..A..TA.A.....G........A..A........ :  500
L11347.1|PUUV|N|Russia|Human|P360|                  : .TT........C......................A.....G.....A..............A..A..G...A.G..C..G........A..A........ :  500
AB297665.2|PUUV|N|Russia|M.g.|DTK/Ufa-97|1997|      : .TT........C......................A.....G.....A..............A..A..G...A.G..C..G........A..A........ :  500
M32750.1|PUUV|N|Russia|M.g.|CG1820|                 : .TT........C......................A.....G.....A..............A..A..G...A.G..C..G........A..A........ :  500
AF442613.1|PUUV|N|Russia|M.g.|CG17/Baskiria-2001|   : .TT........C......................A.....G.....A..............A..A..G...A.G..C..G........A..A........ :  500
KX815395.1|PUUV|N|Poland|M.g.|KS14/118|2009|        : ....A..A..A..G......C...................G.....A..A.....T..G..A..A..G..AA.A.....G.....T..C..A........ :  500
GQ339483.1|PUUV|N|Sweden|M.g.|Bergsjobo/Mg25/05|    : ..T.A.....AC.T..........................G..A.....A........G..A..AA.G..TA.A.....G........C..A.....A.. :  500
AJ223377.1|PUUV|N|Sweden|M.g.|Solleftea/Cg6/95|     : ..T.A.....AC.T..........................G..A.....A........G..A..AA.G..TA.A.....G........C..A.....A.. :  500
GQ339484.1|PUUV|N|Sweden|M.g.|Faboviken/Mg26/05|    : ..T.A.....AC.T..C....................G..G........A.....T..G..A..AA.G...A.A.....G........C..A.....A.. :  500
GQ339485.1|PUUV|N|Sweden|M.g.|Mangelbo/Mg1/05|      : .TT.A.....AC.C..C.................A...........A..A..............AA.G..TA.A.....G........C..A.....A.. :  500
GQ339486.1|PUUV|N|Sweden|M.g.|Munga/Mg2/05|         : .TT.A.....AC.C..C.................A...........A..................AA.G...A.A.....G........C..A.....A.. :  500
GQ339487.1|PUUV|N|Sweden|M.g.|Munga/Mg16/05|        : .TT.A.....AC.C..C.................A...........A..................AA.G...A.A.....G........C..A.....A.. :  500
AJ223376.1|PUUV|N|Sweden|M.g.|Solleftea/Cg3/95|     : ..T.A.....AC.T..C.......................G........A.....T........AA.G...A.A.....G.....T..C..A.....A.. :  500
AF367071.1|PUUV|N|Russia|M.r.|CRF366|               : ....T..A...C...........................................T........A.....AA.G..............A..A........ :  500
AF367064.1|PUUV|N|Russia|M.g.|CG144|                : ....T..A...C...........................................T........A.....AA.G..............A..A........ :  500
AF367065.1|PUUV|N|Russia|M.g.|CG168|                : ....T..A...C...........................................T..G.....A.....AA.G..............A..A........ :  500
AF367068.1|PUUV|N|Russia|M.g.|CG315|                : ....T..A...C...........................................T........A.....AA.G..............A..A........ :  500
AF367069.1|PUUV|N|Russia|M.r.|CRF161|               : ....T..A...C...........................................T........A.....AA.G..............A..A........ :  500
```

```
                                                                                        Korva M et al. Microbes Infect 2009  PUUL ACATCATTTGAGGA

                                                       TTTTGAAGGC  (PUU1P)  Aitichou M at al J Virol Meth 2005
                                                                *       420         *       440         *       460         *       480         *       500
2014.00598|PUUV|N|France|H.s.|59|FOURMIES|          : TCCTGAAGGCTTTATATATGTTGTCAACACGTGGGAGACAAACTGTGAAGGAAAACAAAGGgACTCGTATCCGTTTTAAAGATGACACTTCCTTTGAGGA :  500
AF367070.1|PUUV|N|Russia|M.r.|CRF308|               : ....T..A...C.............................................T........A.....AA.G..............A..A........ :  500
AF367066.1|PUUV|N|Russia|M.g.|CG215|                : ....T..A...C.............................................T.....A..A.....AA.G..............A..A........ :  500
AF367067.1|PUUV|N|Russia|M.g.|CG222|                : ....T..A...C.............................................T.....A..A.....AA.G..............A..A........ :  500
Z46942.1|PUUV|N|Finland|M.g.|Puumala/1324Cg/79|     : ....T..A..C.....C...C.T.....G...........................T........A.....AA.G...........T..A..A.....A.. :  500
Z30702.1|PUUV|N|Finland|M.g.|Evo/12Cg/93|           : .T..T..A.....G..C...C.T.....G...........G........A.....T..G.....A.....AA.G.....G.....T..A..A.....A.. :  500
Z30703.1|PUUV|N|Finland|M.g.|Evo/13Cg/93|           : .T..T..A.....G..C...C.T.....G...........G........A.....T..G.....A.....AA.G.....G.....T..A..A.....A.. :  500
Z30704.1|PUUV|N|Finland|M.g.|Evo/14Cg/93|           : .T..T..A..C..G..C...C.T.....G...........G........A.....T..G..T..A.....AA.G.....G.....T..A..A.....A.. :  500
Z30705.1|PUUV|N|Finland|M.g.|Evo/15Cg/93|           : .T..T..A.....G..C...C.T.....G...........G........A.....T..G..T..A.....AA.G.....G.....T..A..A.....A.. :  500
Z69985.1|PUUV|N|Finland|M.g.|Virrat/25Cg/95|        : ....T..A..C.....C...C.T.....G...........G..C.....A..G..T.......A.....AA.A..C..G.....T......A.....A.. :  500
JN831947.1|PUUV|N|Finland|Pieksamaki/human_lung/20  : ....T..A..C.....C...C.T.....G...........................T........A.....AA.G.....G.....T..A..A.....A.. :  500
JN831943.1|PUUV|N|Finland|Pieksamaki/Mg7/2008|      : ....T..A..C.....C...C.T.....G...........................T........A.....AA.G.....G.....T..A..A.....A.. :  500
JN831950.1|PUUV|N|Finland|Pieksamaki/human_kidney/  : ....T..A..C.....C...C.T.....G...........................T........A.....AA.G.....G.....T..A..A.....A.. :  500
JQ319166.1|PUUV|N|Finland|Konnevesi/Mg_014B/2005|   : ....T.....C.....C...C.T.....G.................A.....G..T........A.....AA.G...........T..A..A.....A.. :  500
JQ319169.1|PUUV|N|Finland|Konnevesi/Mg_027B/2005|   : ....T.....C.....C...C.T.....G.................A.....G..T........A.....AA.G...........T..A..A.....A.. :  500
JQ319167.1|PUUV|N|Finland|Konnevesi/Mg_015B/2005|   : ....T.....C.....C...C.T.....G...........G...........G..T.....A..A.....AA.G...........T..A..A.....A.. :  500
JQ319164.1|PUUV|N|Finland|Konnevesi/Mg_06B/2005|    : ....T.....C.....C...C.T.....G.................A.....G..T........A.....AA.G...........T..A..A.....A.. :  500
JQ319165.1|PUUV|N|Finland|Konnevesi/Mg_09B/2005|    : ....T.....C.....C...C.T.....G.................A.....G..T........A.....AA.G...........T..A..A.....A.. :  500
JQ319170.1|PUUV|N|Finland|Konnevesi/Mg_074B/2005|   : ....T.....C.....C...C.T.....G.................A.....G..T........A.....AA.G...........T..A..A.....A.. :  500
JQ319171.1|PUUV|N|Finland|Konnevesi/Mg_M114B/2005|  : ....T.....C.....C...C.T.....G...........G...........G..T.....A..A.....AA.G...........T..A..A.....A.. :  500
AJ238788.1|PUUV|N|Russia|M.g.|Karhumaki|            : ....T..A..CC....C...C.T........A........G.....A........T..G.....G..C..AA.G..C..G.....T..A..A.....A.. :  500
AJ238789.1|PUUV|N|Russia|M.g.|Kolodozero|           : .T..T...........C...C.T.....G...........G.................G.....A..C..AA.G..C..G.....T..A..A.....A.. :  500
AJ314597.1|PUUV|N|Finland|M.g.|Pallasjarvi/63Cg/98  : ....T..A..C.....C...C.T...........A.....G.....A.....G..T..G.....A..C..AA.G.....G.....T..A..A.....A.. :  500
NC_005224.1|PUUV|N|Finland|M.g.|Sotkamo-revu-NCBI2  : ....T..A........C...C.C.....G...........G.....A........T..G.....A.....AA.G.....G.....T..A..A.....A.. :  500
AJ238790.1|PUUV|N|Russia|M.g.|Gomselga|             : ....T..A........C...C.C.....G...........G.....A........T..G.....A.....AA.G.....G.....T..A..A........ :  500
AB010731.1|Hokkaido-V|N|Japan|M.r.|Tobetsu-60Cr-93  : .T..A.....AC.T..............C..A..AC.T...........A..G...........AA.G.....A.....G.....T..A..A.....A.. :  500
AB010730.1|Hokkaido-V|N|Japan|M.r.|Kamiiso-8Cr-95|  : .T..A.....AC.T..C...........C..A..AC.T...........A.....T........GA.G.....A.....G.....T..A..A.....A.. :  500
JX046487.1|Muju-V|N|South-Korea|M.r.|11-5|2011      : .......A..AC.G..C...C.A..T...A.G..T..G.....A..T..A..............AA.G..TA.A.....G.................A.. :  500
JX046484.1|Muju-V|N|South-Korea|M.r.|11-4|2011|     : .......A..AC.G..C...C.A..T...A.G..T..G.....A..T..A..............AA.G..TA.A.....G.................A.. :  500
JX028273.1|Muju-V|N|South-Korea|M.r.|11-1|2011|     : .......A..AC....C...C.A..T...A.G..T..G.....A..T..A..............AA.G..TA.A.....G.................A.. :  500
DQ138128.1|Muju-V|N|South-Korea|E.r.|00-18|         : .......A..A..G...........C...A.G..A..G.....A..C..A..G..T..G..A..A..A..TA.G.....G........C..A.....A.. :  500
DQ138140.1|Muju-V|N|South-Korea|E.r.|99-27|         : .......A..A..G...........C...A.G..A..G.....A..C..A..G..T..G..A..A..A..TA.G.....G........C..A.....A.. :  500
DQ138133.1|Muju-V|N|South-Korea|E.r.|96-1|          : .......A..A..G...........C...A.G..A..G.....A..C..A..G..T..G..A..A..A..TA.G.....G........C..A.....A.. :  500
DQ138142.1|Muju-V|N|South-Korea|E.r.|99-28|         : .......A..A..G...........C...A.G..A..G.....A..C..A..G..T..G..A..A..A..TA.G.....G........C..A.....A.. :  500
```

```
                Korva M et al. Microbes Infect 2009  CAT (end of PUUL)             PUU S  TCCATGCCAACAGCCCAGTCAAC       CGTCTTCTCGAATGAGG  PUU D
                                                                                    Aitichou M at al J Virol Meth 2005  CGWCTHCTYDAATGTGGWCCT  HANTAV1L
                                                                   *        520         *        540         *        560         *        580         *        600
2014.00598|PUUV|N|France|H.s.|59|FOURMIES|        : CATCAATGGTATCAGGAGACCGAAGCACCTATATGTTTCCATGCCTACTGCTCAATCAACTATGAAGGCTGAAGAATTGACCCCCGGGCGATTTAGAACC :  600
2014.00233|PUUV|N|France|H.s.|59|FOURMIES|        : .....................A.....T................................C.................A........A............ :  600
2014.00097|PUUV|N|France|H.s.|02|SAINT-MICHEL|    : .....................A.............................................................................. :  600
2014.00488|PUUV|N|France|H.s.|08|SIGNY-LE-PETIT|  : ..........................................................................................G......... :  600
2014.00613|PUUV|N|France|H.s.|59|FOURMIES|        : ..........................................................................................G......... :  600
2015.00402|PUUV|N|France|H.s.|08|CHARLEVILLE-MEZIE : .....................A........................................................A.....T............... :  600
2015.00422|PUUV|N|France|H.s.|59|ANOR|            : .................................................................................................... :  600
2015.00488|PUUV|N|France|H.s.|08|SEDAN|           : .....................A.............................C..........................A.....T............... :  600
2015.00498|PUUV|N|France|H.s.|08|REMILLY-AILLICOUR : .................................................................................................... :  600
2016.00295|PUUV|N|France|H.s.|08|CORNY-MACHEROMENI : .....................A......................................C.................A.....T............... :  600
2016.00333|PUUV|N|France|H.s.|59|FOURMIES|        : ............T....................................................................................... :  600
2016.00345|PUUV|N|France|H.s.|59|FOURMIES|        : ............T....................................................................................... :  600
2016.00357|PUUV|N|France|H.s.|02|HIRSON|          : ............T....................................................................................... :  600
2016.00427|PUUV|N|France|H.s.|59|FOURMIES|        : .................................................................................................... :  600
2016.00469|PUUV|N|France|H.s.|02|PREMONTRE|       : .....................A.............................G..........................A.....T........C...... :  600
AJ277075.1|PUUV|N|Belgium|M.g.|Montbliart/CG14444| : ...T........................................................................C.......T............... :  600
2014.00053|PUUV|N|France|H.s.|08|SECHEVAL|        : .....................A.............................C..........................A.....T............... :  600
KT247593.1|PUUV|N|France|M.g.|08|Ardennes/Mg75/201 : .....................A.....T..................................................A.....T............... :  600
KT247592.1|PUUV|N|France|M.g.|08|Ardennes/Mg156/20 : .....................A.....T..................................................A.....T............... :  600
2015.00019|PUUV|N|France|H.s.|08|ETEIGNIERES|     : ............T........A........................................................A.....T............... :  600
2016.00239|PUUV|N|France|H.s.|08|VIREUX-MOLHAIN|  : ...T.................A........................................................A.....T............... :  600
2015.00329|PUUV|N|France|H.s.|08|BOGNY-SUR-MEUSE| : .....................A..A.....................................................A.....T............... :  600
2014.00209|PUUV|N|France|H.s.|08|TREMBLOIS-LES-ROC : .....................A........................................................A.....T............... :  600
2015.00456|PUUV|N|France|H.s.|94|ALFORTVILLE|     : .....................A........................................................A.....T............... :  600
2012.00018|PUUV|N|France|H.s.|08|ROCROI|          : .....................A..............................................................T............... :  600
AJ277032.1|PUUV|N|Belgium|M.g.|Momignies/47Cg/96| : .....................A..A.....................................................A.....T............... :  600
AJ277034.1|PUUV|N|Belgium|M.g.|Couvin/59Cg/97|    : .....................A........................................................A..T..T............... :  600
AJ277033.1|PUUV|N|Belgium|M.g.|Momignies/55Cg/96| : ...................T.A..A.....................................................A.....T............... :  600
2012.00638|PUUV|N|France|H.s.|08|GIVET|           : .....................A......T.................................................A.....T............... :  600
2015.00328|PUUV|N|France|H.s.|08|VRIGNE-MEUSE|    : .....................A..............................................................T............... :  600
2015.00419|PUUV|N|France|H.s.|08|DOUZY|           : T...................................................................................T............... :  600
2016.00310|PUUV|N|France|H.s.|08|THIN-LE-MOUTIER| : .....................A......................................C.................A.....T............... :  600
AJ277030.1|PUUV|N|Belgium|M.g.|Thuin/33Cg/96|     : .......................................T...........G................................T............... :  600
AJ238779.1|PUUV|N|Germany|M.g.|NRW/Cg-Erft|       : ......C..............A..............C..T............................................T..A............ :  600
2014.00276|PUUV|N|France|H.s.|59|LILLE|           : .....................T.....T..G..............A......................................T............... :  600
U22423.1|PUUV|N|Belgium|M.g.|CG13891|             : ...T..C..............A......................................C...............C.......T............... :  600
2014.00321|PUUV|N|France|H.s.|59|FOURMIES|        : T................................................................................................... :  600
2015.00457|PUUV|N|France|H.s.|55|REVIGNY-SUR-ORNAI : .....................A.............................G..........................A.....T........C...... :  600
2013.00250|PUUV|N|France|H.s.|62|VENDIN-LS-BTHUNE| : .....................A......................................C.......................T............... :  600
2016.00282|PUUV|N|France|H.s.|59|MORBECQUE|       : .....................A......................................C.......................T............... :  600
AJ277076.1|PUUV|N|Belgium|M.g.|Montbliart/CG14445| : ...T.................A......................................................C.......T............... :  600
2014.00153|PUUV|N|France|H.s.|02|BUIRONFOSSE|     : T...........................................................................C.......T............... :  600
2014.00171|PUUV|N|France|H.s.|59|SAINT-SAULVE|    : ...T.................A..A...T...................A...........................C.A.....T.....G......... :  600
2016.00293|PUUV|N|France|H.s.|60|LA-NEUVILLE-SUR-R : .........C.....A........................................................G...........T............... :  600
2014.00499|PUUV|N|France|H.s.|94|CHAMPIGNY-SUR-MAR : ............................................................................C....................... :  600
2012.00057|PUUV|N|France|H.s.|59|COUSOLRE|        : ...T........................................................................C.......T............... :  600
2012.00349|PUUV|N|France|H.s.|02|ENGLANCOURT|     : T...........................................................................C.......T............... :  600
2014.00135|PUUV|N|France|H.s.|02|ATHIES-SOUS-LAON| : ............................................................................C....................... :  600
2014.00184|PUUV|N|France|H.s.|59|FOURMIES|        : ...T........................................................................C.......T............... :  600
2016.00182|PUUV|N|France|H.s.|08|BOULZICOURT|     : ...T........................................................................C....T..T............... :  600
2016.00286|PUUV|N|France|H.s.|02|TRUCY|           : .....................T......................................................C....................... :  600
2016.00325|PUUV|N|France|H.s.|59|AULNOYE-AYMERIES| : ...T........T...............................................................C.......T..............T :  600
2016.00452|PUUV|N|France|H.s.|02|LAON|            : ............................................................................C....................... :  600
2016.00467|PUUV|N|France|H.s.|02|AISNE|           : ............................................................................C.A.....T............... :  600
```

```
               Korva M et al. Microbes Infect 2009  CAT (end of PUUL)            PUU S  TCCATGCCAACAGCCCAGTCAAC       CGTCTTCTCGAATGAGG  PUU D
                                                                                  Aitichou M at al J Virol Meth 2005  CGWCTHCTYDAATGTGGWCCT  HANTAV1L
                                                                    *       520         *       540         *       560         *       580         *       600
2014.00598|PUUV|N|France|H.s.|59|FOURMIES|         : CATCAATGGTATCAGGAGACCGAAGCACCTATATGTTTCCATGCCTACTGCTCAATCAACTATGAAGGCTGAAGAATTGACCCCCGGGCGATTTAGAACC :  600
2015.00657|PUUV|N|France|H.s.|02|CILLY|            : ...T..C.............................C...............................................T..A............ :  600
2015.00526|PUUV|N|France|H.s.|08|MONTHERME|        : .....................A.............................C........................C.......T............... :  600
2015.00045|PUUV|N|France|H.s.|59|FOURMIES|         : ...T.................A......................................................C.......T............... :  600
2015.00430|PUUV|N|France|H.s.|02|MONTCORNET|       : .......................................T...........C........................C.......T............... :  600
2016.00311|PUUV|N|France|H.s.|02|LAON|             : ............................................................................C....................... :  600
AJ277031.1|PUUV|N|Belgium|M.g.|Montbliart/23Cg/96| : ...T........................................................................C.......T..A............ :  600
2016.00326|PUUV|N|France|H.s.|02|LAON|             : ............................................................................C....................... :  600
2016.00353|PUUV|N|France|H.s.|02|CESSIERES|        : ............................................................................C....................... :  600
2012.00061|PUUV|N|France|H.s.|02|LANISCOURT|       : ............................................................................C....................... :  600
2014.00174|PUUV|N|France|H.s.|02|ST-ERME-OUTRE-ET- : ............................................................................C....................... :  600
2016.00268|PUUV|N|France|H.s.|02|PRESLES-ET-THIERN : .....................A......................................................C....................... :  600
2015.00660|PUUV|N|France|H.s.|02|VIC-SUR-AISNE|    : ............T..A...........T..G.....................................................T...........G... :  600
2012.00025|PUUV|N|France|H.s.|51|SAINTE-MENEHOULD| : ...A...........AC...A..A..............A.....C..............C.................A..T......A.G..C..G... :  600
2015.00665|PUUV|N|France|H.s.|51|REIMS|            : ...A........T..AC...A..A..T...........A.....C..............C...........G.....A.....T...A.G..C..G... :  600
2012.00307|PUUV|N|France|H.s.|54|COLOMBEY-LES-BELL : ...A...........A.....A......T.G........A....................C....................T..T..AA.G.....G..T :  600
2012.00123|PUUV|N|France|H.s.|55|BAR-LE-DUC|       : ...A.....C.....AC...T..A.........................................................T..T...A.G..C..G... :  600
2012.00278|PUUV|N|France|H.s.|51|REIMS|            : ...A...........AC...A..A..T...........A.....C..............C.................A..T..T...A.G..C..G... :  600
2015.00185|PUUV|N|France|H.s.|68|FELLERING|        : ...A...........AC...A..A..............A.....C...........G..C.................A..T......A.G..C..G... :  600
KJ994776.1|PUUV|N|Germany|M.g.|Mu/07/1219|2007|    : T..T........T.....G..A..A..T..G.....G........A.............................GC.......T..T...........T :  600
2012.00086|PUUV|N|France|H.s|58|CHEVROCHES|        : T...........TC.C..G..A..............G..............C........................GC....T..T.....G.....G... :  600
KT247595.1|PUUV|N|France|M.g.|45|Orleans/Mg29/2010 : T...........TC.C..G..A..............G..............C........................GC....T..T.....G.....G... :  600
KT247594.1|PUUV|N|France|M.g.|45|Orleans/Mg23/2010 : T...........TC.C..G..A..............G..............C........................GC....T..T.....G.....G... :  600
KY365004.1|PUUV|N|France|M.g.|45|Orleans/NCHA373/2 : T...........TC.C..G..A.....T........G..............C........................GC....T..T.....G.....G... :  600
2012.00301|PUUV|N|France|H.s.|25|MOUTHE|           : ...A.....C..T..A.....A......T.G.....A...........A..A.....C..C.....A..A.....G..A..T..T..A........G... :  600
AM695638.1|PUUV|N|France|M.g.|Mignovillard/CgY02/2 : ...A.....C..T..A.....A......T.G.....A...........A..A.....C..C.....A..A.....G..A..T..T..A........G... :  600
KT247597.1|PUUV|N|France|M.g.|39|Jura/Mg214/2010|  : ...A..C..C..T..A.....A......T.G.....A...........A..A.....T..C.....A..A..G..G..A..T..T..A........G... :  600
KT247596.2|PUUV|N|France|M.g.|39|Jura/Mg2/2010|    : ...A..C..C..T..A.....A......T.G.....A...........A..A.....T..C.....A..A..G..G..A..T..T..A........G... :  600
2012.00102|PUUV|N|France|H.s.|39|COISERETTE|       : ...A.....C..T..A.....A......T.G.....A...........A..A.....C........A..A.....G..A..T..T..............T :  600
2012.00536|PUUV|N|France|H.s.|39|LA-PESSE|         : ...A.....C..T..A.....A......T.G.....A...........A..A.....C........A..A.....G..A..T..T..............T :  600
2014.00622|PUUV|N|France|H.s.|39|ARBOIS|           : ...A.....C..T..A.....A......T.G.....A...........A..A.....C..C.....A..A.....G..A..T..T..A........G... :  600
2015.00567|PUUV|N|France|H.s.|70|RIOZ|             : ...A.....C..T..A.....A......T.G.....A...........A..A.....C..C.....A..A.....G..A..T..T..A..G.....G... :  600
2014.00637|PUUV|N|France|H.s.|25|SAULES|           : ...A.....C..T..A.....A......T.G.....A..T........A..A.....C..C.....A..A.....G..A..T..T..A..G.....G... :  600
2012.00396|PUUV|N|France|H.s.|39|SAINT-CLAUDE|     : ...A.....C..T..A.....A......T.G.....A...........A..A.....C..C.....A..A.....G..A..T..T..A........G... :  600
2014.00120|PUUV|N|France|H.s.|38|LE-MOUTARET|      : ...A.....C..T..A.....A......T.G.....A...........A..A.....C..C.....A..A.....G..A..T..T..A........G... :  600
2015.00153|PUUV|N|France|H.s.|73|GREZY-SUR-ISERE|  : ...A.....C..T..A.....A......T.G.....A...........A..A.....C..C.....A..A.....G..A..T..T..A........G... :  600
2015.00504|PUUV|N|France|H.s.|70|RONCHAMP|         : ...A.....C..TC.......A......T.G.....A...........A..A..G..T..C........A.....G..A.....T..A........G... :  600
2016.00275|PUUV|N|France|H.s.|21|JALLANGES|        : ...A.....C..T..A.....A......T.G.....A...........A..A.....C..C.....A..A.....G..A..T..T..A........G... :  600
2016.00320|PUUV|N|France|H.s.|25|SAINT-VIT|        : ...A........T..A.....A......T.G.....A...........A..A.....C..C.....A..A.....G..A..T..T..A........G... :  600
2015.00410|PUUV|N|France|H.s.|70|ANGIREY|          : ...A.....C..TC.......A......T.G.....A...........A..A..G..T..C........A.....G..A.....T..A............ :  600
DQ016430.2|PUUV|N|Germany|M.g.|Bavaria/CG33/04|    : ...T.....C..T........A.....TT.G.....G........C........G...........A...........A.....T..TA.G..C..G... :  600
DQ016432.2|PUUV|N|Germany|M.g.|Bavaria/CG41/04|    : ...T........T........A.....TT.G.....G........C........G...........A.....G.....A.....T..TA.G..C..G..T :  600
AY954723.2|PUUV|N|Germany|M.g.|Bavaria-CG34/04|    : ...T........T........A.....TT.G.....G........C........G...........A.....G.....A.....T..TA.G..C..G..T :  600
AY954722.2|PUUV|N|Germany|M.g.|Bavaria-CG9/04|     : ...T........T........A.....TT.G.....G........C........G...........A...........A.....T..TA.G..C..G..T :  600
2012.00402|PUUV|N|France|H.s.|60|GOUVIEUX|         : ...A.....C..TC.A.....A......T.G.....G...........A..A.....G........A..A.....GC.A..T..T..............T :  600
2014.00540|PUUV|N|France|H.s.|60|CHAMBLY|          : ...A........TC.A.....A......T.G.....G...........A..A.....G........A..A.....GC.A..T..T............... :  600
AJ314600.1|PUUV|N|Balkan|M.g.|Balkan-1|            : ...T.....C..T..A.....A.....TT.......A..T.....A..A..C..G.....A........A.....GC.T..T..T..CA.G.CC..G..A :  600
AJ314601.1|PUUV|N|Balkan|M.g.|Balkan-2|            : .........C..T..A.....A.....TT.......A..T.....A..A..C..G.....A........A.....GC.T..T..T..CA.G.CC..G..A :  600
FN377821.1|PUUV|N|Hungary|M.g.|Mg9/HungaryTR17/00| : ...T.....C..T........A.....TT.......A........A..A..C..G..C..A........A.....GC.T..T..T..CA.G.CC..G..T :  600
FN377822.1|PUUV|N|Hungary|M.g.|Mg23/HungaryTR17/00 : .........C..T..A.....A.....TT.......A........A..A..C..G.....A........A.....GC.T..T..T..CA.G.CC..G..A :  600
GQ339473.1|PUUV|N|Sweden|M.g.|Kiviniemi/Mg3/05|    : ...T...........AC...A..A.....T.....G.................G..C........A..A......C.T..A..G..T..G.CC..T..A :  600
GQ339474.1|PUUV|N|Sweden|M.g.|Kiviniemi/Mg5/05|    : ...T...........AC...A..A.....T.....A.................G..C........A..A......C.T..A..G..T..G.CC..T..A :  600
GQ339475.1|PUUV|N|Sweden|M.g.|Kiviniemi/Mg6/05|    : ...T...........AC...A..A.....T.....A.................G..C........A..A......C.T..A..G..T..G.CC..T..A :  600
GQ339476.1|PUUV|N|Sweden|M.g.|Aijajarvi/Mg7/05|    : ...T...........AC...A..A.....T.....G.................G..C........A..A......C.T..A..G..T..G.CC..T..A :  600
```

```
          Korva M et al. Microbes Infect 2009  CAT (end of PUUL)             PUU S  TCCATGCCAACAGCCCAGTCAAC      CGTCTTCTCGAATGAGG  PUU D
                                                                              Aitichou M at al J Virol Meth 2005  CGWCTHCTYDAATGTGGWCCT  HANTAV1L
                                                         *       520         *       540         *       560         *       580         *       600
2014.00598|PUUV|N|France|H.s.|59|FOURMIES|       : CATCAATGGTATCAGGAGACCGAAGCACCTATATGTTTCCATGCCTACTGCTCAATCAACTATGAAGGCTGAAGAATTGACCCCCGGGCGATTTAGAACC :  600
GQ339477.1|PUUV|N|Sweden|M.g.|Aijajarvi/Mg9/05|  : ...T...........AC....A..A.....T.....G.................G..C........A..A......C.T..A..G..T..G..CC.T..A :  600
GQ339478.1|PUUV|N|Sweden|M.g.|Jockfall/Mg12/05|  : T..T.....C.....AC....A..A.....T.....A..............C..G..C..C.....A..A......C....A..A..T..G..CC.T..A :  600
GQ339482.1|PUUV|N|Sweden|M.g.|Kalvudden/Mg22/05| : ...T...........AC.......A.....T.....G..T..............G..C........A..A......C.T..A..A..C..G...C.T..A :  600
GQ339479.1|PUUV|N|Sweden|M.g.|Moskosel/Mg17/05|  : ...T........T..AC.......A.....T.....A........C...........C..C.....A..G......C.T..A..A.....G..CC.T..A :  600
GQ339481.1|PUUV|N|Sweden|M.g.|Ljustrask/Mg20/05| : ...T...........AC.......A..T..T.....A........C.....C.....C..C.....A..A......C.C..A..A..A..G..CC.T..A :  600
GQ339480.1|PUUV|N|Sweden|M.g.|Gyttjea/Mg19/05|   : ...T...........AC.......A.....T.....A........C...........C..C.....A..A......C.C..A..A.....G..CC.T..A :  600
AY526219.1|PUUV|N|Sweden|Human|Umea/hu|          : T........C..T...C....A........C.....A..T........A..C..G..T........A..A..G...C.T..A..A..T..G...C.C..A :  600
AJ223380.1|PUUV|N|Sweden|M.g.|Tavelsjo/Cg81/94|  : .........C.....AC....A........C.....A...........A..C.....T..C........A......C.T..A..A..T.....CC.C..A :  600
U14137.1|PUUV|N|Bosnia-H.|M.g.|Vranica|          : .........C.....AC....A........C.....A...........A..C.....T..C........A......C.T..A..A..T.....CC.C..A :  600
AJ223371.1|PUUV|N|Sweden|M.g.|Huggberget/Cg36/94|: T........C.....AC....A........C.....A...........A..C.....T..C.....A..A.....GC.T..A..A..T..G..CC.C..A :  600
Z48586.1|PUUV|N|Sweden|M.g.|Vindeln/L20Cg/83|    : T........C.....AC....A........C.....A...........A..C..G..T..C.....A..A......C.T..A..A..T..G..CC.C..A :  600
AJ223374.1|PUUV|N|Sweden|M.g.|Mellansel/Cg47/94| : T........C..T...C....A.....T..C.....A..T.....C..A..C..G..T........A..A..G...C.T..A..A..T..G...C.C..A :  600
AJ223375.1|PUUV|N|Sweden|M.g.|Mellansel/Cg49/94| : T........C..T...C....A.....T..C.....A..T.....C..A..C..G..T........A..A..G...C.T..A..A..T..G...C.C..A :  600
AJ238791.1|PUUV|N|Denmark|M.g.|Fyn/19|           : ...A...........AC.......A...........G..A.....G.....C.....G...........A..G..G........T..T.....CC.C..  :  600
AJ278092.1|PUUV|N|Denmark|M.g.|Fyn47|            : ...A...........AC.......A..T........G..T.....C.....C.C...............A..G.....A........T.....CC.C..T :  600
AJ278093.1|PUUV|N|Denmark|M.g.|Fyn131|           : ...A...........AC.......A..T........G..A.....A.....C.C...............G..G.....A.....T..T.....CC.C..T :  600
AJ223368.1|PUUV|N|Norway|M.g.|Eidsvoll/1124v|    : T........C..T..A.....A..A...........G..T...........A..G.....C.....A..A.....G..A..A..A..C..G...C.G..G :  600
AJ223369.1|PUUV|N|Norway|M.g.|Eidsvoll/Cg1138/87|: T........C..T..A.....A..A...........G..T...........A..G.....C.....A..A.....G..A..A..A..C..G...C.G..G :  600
JN657228.1|PUUV|N|Latvia|M.g.|Jelgava/Mg149/2008|: ...T..C..C..T..A........A..T........G..............C..G.....A........A..G...C.C..A..A..T......C.T..A :  600
KX757839.1|PUUV|N|Lithuania|M.g.|LT15/164|2015|  : T..T.....C.....A.....A..A...T.G....................C........C.....A..A......C.A..A..A..T......C.T..A :  600
KX757840.1|PUUV|N|Lithuania|M.g.|LT15/174|2015|  : T..T.....C.....A.....A..A...T.G....................C........C.....A..A......C.A..A..A..T......C.T..A :  600
KX815394.1|PUUV|N|Poland|M.g.|KS13/855|2009|     : ...T.....C...........A.....TT.G..............C.....C........C.....A..A......C.T..A..A..T......C.T..A :  600
KX757841.1|PUUV|N|Lithuania|M.g.|LT15/201|2015|  : T..T.....C.....A.....A..A...T.G....................C........C.....A..A......C.A..A..A..T......C.T..A :  600
AJ314598.1|PUUV|N|Russia|M.g.|Baltic/49Cg/00|    : T........C..T.....G..A..A..T..T.....A........A...........T..A........A..G...C.T..A..T.........C.G..A :  600
AJ314599.1|PUUV|N|Russia|M.g.|Baltic/205Cg/00|   : ...T.....A..A........A..A..T..G.....C........A...........C..A........A..G...C.C..A..T.....G..CC.T..A :  600
JN657229.1|PUUV|N|Latvia|M.g.|Madona/Mg99/2008|  : ...T........T........C..A..T..G........T........A........C..A........A......C.C..A..T.....G..CC.T..A :  600
JN657232.1|PUUV|N|Latvia|M.g.|Madona/Mg233/2008| : ...T........T........C..A..T..G........T........A........C..A........A......C.C..A..T.....G..CC.T..A :  600
JN657230.1|PUUV|N|Latvia|M.g.|Jelgava/Mg136/2008|: T..T..C..C..T........A..A.....G.....C..T.....C..A..C.....C..A........A......C.C..A..T.....G..CC.T..A :  600
JN657231.1|PUUV|N|Latvia|M.g.|Jelgava/Mg140/2008|: T..T..C..C..T........A..A.....G.....C..T.....C..A..C.....C..A........A......C.C..A..T.....G..CC.T..A :  600
Z30707.1|PUUV|N|Russia|M.g.|Udmurtia/458Cg/88|   : T..T.....C..T........A..A..T........A..............C..G..C..C.....A.........C.T..A.....A..G..CC.T..A :  600
Z30706.1|PUUV|N|Russia|M.g.|Udmurtia/444Cg/88|   : T..T.....C...........A..A..T........A..............C..G..C..C.....A.........C.T..A.....A..G..CC.T..A :  600
Z84204.1|PUUV|N|Russia|M.g.|Kazan|               : T..T.....C...........A..A..T........A..............C..G..C..C.....A.........C.T..A.....A..G..CC.T..A :  600
Z30708.1|PUUV|N|Russia|M.g.|Udmurtia/338Cg/92|   : T..T.....C..T........A..A..T........A..............C..G..C..C.....A.........C.T..A.....A..G..CC.T..A :  600
Z21497.1|PUUV|N|Russia|M.g.|Udmurtia/894Cg/91|   : T..T.....C...........A..A..T........A..............C..G..C..C.....A.........C.T..A..T..A......C.T..A :  600
AB433843.2|PUUV|N|Russia|M.g.|Samara_49/CG/2005| : T..T.....C........G..A..A..T........A...........A.....G..C..C...............C.C..A.....AA.G...C.T..A :  600
AB433845.2|PUUV|N|Russia|M.g.|Samara_94/CG/2005| : T..T.....C.....A.....A..A..T........A.................G..C..C...............C.T..A.....A..G..CC.T..A :  600
L11347.1|PUUV|N|Russia|Human|P360|               : T........C...........A..A...........A..............C..G..C..C.....A.........C.T..A..T..A..G..CC.T..A :  600
AB297665.2|PUUV|N|Russia|M.g.|DTK/Ufa-97|1997|   : T........C...........A..A...........A..............C..G..C..C.....A.........C.T..A..T..A..G..CC.T..A :  600
M32750.1|PUUV|N|Russia|M.g.|CG1820|              : T........C...........A..A...........A..............C..G..C..C.....A.........C.T..A..T..A..G..CC.T..A :  600
AF442613.1|PUUV|N|Russia|M.g.|CG17/Baskiria-2001|: T........C...........A..A...........A..............C..G..C..C.....A.........C.T..A..T..A..G..CC.T..A :  600
KX815395.1|PUUV|N|Poland|M.g.|KS14/118|2009|     : T..T........T.....G..T.................T...........C.....C.................GC....A..T..AA.G...C....A :  600
GQ339483.1|PUUV|N|Sweden|M.g.|Bergsjobo/Mg25/05| : T........C.....A.....T..A..TT.......G...........A..A..G..C..A.....A..A.....G..A..A..A..C..G...C....A :  600
AJ223377.1|PUUV|N|Sweden|M.g.|Solleftea/Cg6/95|  : T........C.....A.....T..A..TT.......G...........A..A..G..C..A.....A........G..A..A..A..C..G...C....A :  600
GQ339484.1|PUUV|N|Sweden|M.g.|Faboviken/Mg26/05| : T........C..T........T..A...T.......G..T........A..A.....C..A.....A..A.....G.....A..A..C......C....A :  600
GQ339485.1|PUUV|N|Sweden|M.g.|Mangelbo/Mg1/05|   : .........C...........A......T.......G..T.....C..A..A..G.....A.....A..A.....G..A..A..A..T......C.G..A :  600
GQ339486.1|PUUV|N|Sweden|M.g.|Munga/Mg2/05|      : .........C...........A......T.......G..T.....C..A..A..G.....A.....A..A.....G..A..A..A..T.....CC.G..A :  600
GQ339487.1|PUUV|N|Sweden|M.g.|Munga/Mg16/05|     : .........C...........A......T.......G..T.....C..A..A..G.....A.....A..A.....G..A..A..A..T.....CC.G..A :  600
AJ223376.1|PUUV|N|Sweden|M.g.|Solleftea/Cg3/95|  : T........C..T........T..A..TT.......A..T........A..A..G..C..A.....A........G.....A..A..C......C....A :  600
AF367071.1|PUUV|N|Russia|M.r.|CRF366|            : ...A.....C..A........A..A...T..........A...........C........A.....A..A..G...C.C..A..T..TA.....C.C..A :  600
AF367064.1|PUUV|N|Russia|M.g.|CG144|             : ...A.....C..A........A..A...T..........A...........C........A.....A..A..G...C.C..A..T..TA.....C.C..A :  600
AF367065.1|PUUV|N|Russia|M.g.|CG168|             : ...A.....C..A........A..A...T.......C..A...........C........A.....A..A..G...C.C..A..T..TA.....C.C..A :  600
AF367068.1|PUUV|N|Russia|M.g.|CG315|             : ...A.....C..A........A..A...T.......C..A...........C........A.....A..A..G...C.C..A..T..TA.....C.C..A :  600
AF367069.1|PUUV|N|Russia|M.r.|CRF161|            : ...A.....C..A........A..A...T.......C..A...........C........A.....A..A..G...C.C..A..T..TA.....C.C..A :  600
```

```
          Korva M et al. Microbes Infect 2009  CAT (end of PUUL)             PUU S  TCCATGCCAACAGCCCAGTCAAC       CGTCTTCTCGAATGAGG  PUU D
                                                                              Aitichou M at al J Virol Meth 2005  CGWCTHCTYDAATGTGGWCCT  HANTAV1L
                                                          *        520         *        540         *        560         *        580         *        600
2014.00598|PUUV|N|France|H.s.|59|FOURMIES|         : CATCAATGGTATCAGGAGACCGAAGCACCTATATGTTTCCATGCCTACTGCTCAATCAACTATGAAGGCTGAAGAATTGACCCCCGGGCGATTTAGAACC :  600
AF367070.1|PUUV|N|Russia|M.r.|CRF308|              : ...A.....C..A........A..A...T......C..A..........C........A.....A..A..G...C.C..A..T..TA.....C.C..A :  600
AF367066.1|PUUV|N|Russia|M.g.|CG215|               : ...A.....C..A........A..A...T......C..A..........C........A.....A..A..G...C.C..A..T..TA.....C.C..A :  600
AF367067.1|PUUV|N|Russia|M.g.|CG222|               : ...A.....C..A........A..A...T......C..A..........C........A.....A..A..G...C.C..A..T..TA.....C.C..A :  600
Z46942.1|PUUV|N|Finland|M.g.|Puumala/1324Cg/79|    : .........C..A.....G..A.....TT..........T.....C..A..C..G.....C........A......C.C..A..A..TA.G...C.C..A :  600
Z30702.1|PUUV|N|Finland|M.g.|Evo/12Cg/93|          : .........C..A..A.....A.....TT...............C.......................A......C.C..A..A..TA....CC.T..A :  600
Z30703.1|PUUV|N|Finland|M.g.|Evo/13Cg/93|          : .........C..A..A.....A.....TT...............C.......................A......C.C..A..A..TA....CC.T..A :  600
Z30704.1|PUUV|N|Finland|M.g.|Evo/14Cg/93|          : .........C..A..A.....A.....TT...............C.......................A......C.C..A..A..TA....CC.T..A :  600
Z30705.1|PUUV|N|Finland|M.g.|Evo/15Cg/93|          : .........C..A..A.....A.....TT...............C.......................A......C.C..A..A..TA....CC.T..A :  600
Z69985.1|PUUV|N|Finland|M.g.|Virrat/25Cg/95|       : ...T..C..C..A........A.....TT.....................C..G.....................C.T..A..A..CA.....C.C..A :  600
JN831947.1|PUUV|N|Finland|Pieksamaki/human_lung/20 : ...T.....C..A.....G..A.....TT....C.....T.....C.....C..G.....C........A......C.C..A..A..TA.G...C.C..A :  600
JN831943.1|PUUV|N|Finland|Pieksamaki/Mg7/2008|     : ...T.....C..A.....G..A.....TT....C.....T.....C.....C..G.....C........A......C.C..A..A..TA.G...C.C..A :  600
JN831950.1|PUUV|N|Finland|Pieksamaki/human_kidney/ : ...T.....C..A.....G..A.....TT....C.....T.....C.....C..G.....C........A......C.C..A..A..TA.G...C.C..A :  600
JQ319166.1|PUUV|N|Finland|Konnevesi/Mg_O14B/2005|  : ......C..C..A.....G..A.....TT.....................C..G.....C........A......C.C..A..G..TA.G...C.C..A :  600
JQ319169.1|PUUV|N|Finland|Konnevesi/Mg_O27B/2005|  : ......C..C..A.....G..A.....TT.....................C..G.....C........A......C.C..A..G..TA.G...C.C..A :  600
JQ319167.1|PUUV|N|Finland|Konnevesi/Mg_O15B/2005|  : ......C..C..A.....G..A.....TT.....................C..G.....C........A......C.C..A..A..TA.G...C.C..A :  600
JQ319164.1|PUUV|N|Finland|Konnevesi/Mg_O6B/2005|   : ......C..C..A.....G..A.....TT.....................C..G.....C........A......C.C..A..G..TA.G...C.CG.A :  600
JQ319165.1|PUUV|N|Finland|Konnevesi/Mg_O9B/2005|   : ......C..C..A.....G..A.....TT.....................C..G.....C........A......C.C..A..G..TA.G...C.CG.A :  600
JQ319170.1|PUUV|N|Finland|Konnevesi/Mg_O74B/2005|  : ......C..C..A.....G..A.....TT.....................C..G.....C........A......C.C..A..G..TA.G...C.C..A :  600
JQ319171.1|PUUV|N|Finland|Konnevesi/Mg_M114B/2005| : ......C..C..A.....G..A.....TT.....................C..G.....C........A......C.C..A..A..TA.G...C.C..A :  600
AJ238788.1|PUUV|N|Russia|M.g.|Karhumaki|           : .........C..A........A.....T..........T..............................A......C.C..A..A..TA.....C.T..A :  600
AJ238789.1|PUUV|N|Russia|M.g.|Kolodozero|          : .........C..A........A.....TT.........T.....C........G.....C........A......C.C..A..A..CA.....C.T..A :  600
AJ314597.1|PUUV|N|Finland|M.g.|Pallasjarvi/63Cg/98 : .........C..A........A.....TT.....................C..G.....C........A......C.C..A..A..TA.....C.T..A :  600
NC_005224.1|PUUV|N|Finland|M.g.|Sotkamo-revu-NCBI2 : .........C..A........A.....TT.........T...........C..G...........A..A......C.C..A..T..CA.....C.C..A :  600
AJ238790.1|PUUV|N|Russia|M.g.|Gomselga|            : G........C..A........A.....T.......C.................G.....C........A..G...C.C..A..A..CA.....C.T..A :  600
AB010731.1|Hokkaido-V|N|Japan|M.r.|Tobetsu-60Cr-93 : .........G..T.....G..A..A..T..G.....G..T.....C..A..........A.............GA.C..A..T...A.G..CC.C...  :  600
AB010730.1|Hokkaido-V|N|Japan|M.r.|Kamiiso-8Cr-95| : ...T..C..G..T..A..G..A..A..T..G.....G.........C..A..........A..........G..GA.T..A..T...A.G...C.C...  :  600
JX046487.1|Muju-V|N|South-Korea|M.r.|11-5|2011     : ...T.....A..TC.C..G..C..A..T........G..A.....A........G..C..........A..G..G..A..A..G..TA.G..C..G..A :  600
JX046484.1|Muju-V|N|South-Korea|M.r.|11-4|2011|    : ...T.....A..TC.C..G..C..A..T........G..A.....A...........C..........A..G..G..A..A..G..TA.G..C..G..A :  600
JX028273.1|Muju-V|N|South-Korea|M.r.|11-1|2011|    : ...T.....G..TC.C..G..C..A..T.....C..G..A.....A...........C..........A.....G..A..A..G..CA.G..C..G..A :  600
DQ138128.1|Muju-V|N|South-Korea|E.r.|00-18|        : ...........TC.A.....T..A..............A.....A...........C..............G...C....A..A..TA.G..CC.G..A :  600
DQ138140.1|Muju-V|N|South-Korea|E.r.|99-27|        : ...........TC.A.....T..A..............A.....A...........C..............G...C....A..A..TA.G..CC.G..A :  600
DQ138133.1|Muju-V|N|South-Korea|E.r.|96-1|         : ...........TC.......T..A..............A.....A...........C..............G...C.A..A..A..TA.G..CC.G..A :  600
DQ138142.1|Muju-V|N|South-Korea|E.r.|99-28|        : ...........TC.A..C..T..A..............A.....A...........C..............G...C....A..A..TA.G..CC.T..A :  600
```

```
                                                                                Bowen MD et al. J Med Virol 1997  TACCCICAITAICCIAA  PPT716R
                                                              *       620         *       640         *       660         *       680         *       700
2014.00598|PUUV|N|France|H.s.|59|FOURMIES|          : ATAGTATGCGGTCTATTTCCAACTCAAATTCAAGTACGCAACATAATGAGCCCTGTCATGGGAGTCATTGGGTTTTCTTTCTTTGTCAAGGATTGGGCAG :  700
2014.00233|PUUV|N|France|H.s.|59|FOURMIES|          : ......................................T...........T....................T....................C....... :  700
2014.00097|PUUV|N|France|H.s.|02|SAINT-MICHEL|      : ...................................G...................................T........T...........C....... :  700
2014.00488|PUUV|N|France|H.s.|08|SIGNY-LE-PETIT|    : .......................................................................T........T...........C....... :  700
2014.00613|PUUV|N|France|H.s.|59|FOURMIES|          : .......................................................................T........T...........C....... :  700
2015.00402|PUUV|N|France|H.s.|08|CHARLEVILLE-MEZIE  : ........T.................G.............................A..............T........T.....T.....C....... :  700
2015.00422|PUUV|N|France|H.s.|59|ANOR|              : .................................................................................................... :  700
2015.00488|PUUV|N|France|H.s.|08|SEDAN|             : ........T.................G.............................A..............T........T.....T.....C....... :  700
2015.00498|PUUV|N|France|H.s.|08|REMILLY-AILLICOUR  : .......................................................................T............................ :  700
2016.00295|PUUV|N|France|H.s.|08|CORNY-MACHEROMENI  : ............T....C.....................................................T....................C....... :  700
2016.00333|PUUV|N|France|H.s.|59|FOURMIES|          : .......................................................................T............................ :  700
2016.00345|PUUV|N|France|H.s.|59|FOURMIES|          : .......................................................................T............................ :  700
2016.00357|PUUV|N|France|H.s.|02|HIRSON|            : .......................................................................T............................ :  700
2016.00427|PUUV|N|France|H.s.|59|FOURMIES|          : .................................................................................................... :  700
2016.00469|PUUV|N|France|H.s.|02|PREMONTRE|         : ........T...T...................G..G.................C..A..............T........T...........C....... :  700
AJ277075.1|PUUV|N|Belgium|M.g.|Montbliart/CG14444|  : ....................G...................................A..............T.....C..T...........C....... :  700
2014.00053|PUUV|N|France|H.s.|08|SECHEVAL|          : ........T.................G.............................A..............T........T.....T.....C....... :  700
KT247593.1|PUUV|N|France|M.g.|08|Ardennes/Mg75/201  : ........T...............................................A..............T........T...........C....... :  700
KT247592.1|PUUV|N|France|M.g.|08|Ardennes/Mg156/20  : ........T...............................................A..............T........T...........C....... :  700
2015.00019|PUUV|N|France|H.s.|08|ETEIGNIERES|       : .....G..T...............................................A..............T........T...........C....... :  700
2016.00239|PUUV|N|France|H.s.|08|VIREUX-MOLHAIN|    : ........T.........................................T.....A..............T........T...........C....... :  700
2015.00329|PUUV|N|France|H.s.|08|BOGNY-SUR-MEUSE|   : ........T...............................................A..............T........T...........C....... :  700
2014.00209|PUUV|N|France|H.s.|08|TREMBLOIS-LES-ROC  : ........T...............................................A..............T.....C..T...........C....T.. :  700
2015.00456|PUUV|N|France|H.s.|94|ALFORTVILLE|       : ........T...............................................G.......................T........A..C....... :  700
2012.00018|PUUV|N|France|H.s.|08|ROCROI|            : ........T...............................................A........T.....T.....C..T...........C....... :  700
AJ277032.1|PUUV|N|Belgium|M.g.|Momignies/47Cg/96|   : ........T.........................................T.....G..............T........T...........C....... :  700
AJ277034.1|PUUV|N|Belgium|M.g.|Couvin/59Cg/97|      : ........T...............................................A..............T........T...........C....... :  700
AJ277033.1|PUUV|N|Belgium|M.g.|Momignies/55Cg/96|   : ........T.........................................T.....G..............T........T...........C....... :  700
2012.00638|PUUV|N|France|H.s.|08|GIVET|             : ........T...................................C...........G..............T........T.....T.....C...T... :  700
2015.00328|PUUV|N|France|H.s.|08|VRIGNE-MEUSE|      : ........T................................T..............A........T.....T........T.....T.....C....... :  700
2015.00419|PUUV|N|France|H.s.|08|DOUZY|             : ...........C.................C..........................A..............T........T...........C....... :  700
2016.00310|PUUV|N|France|H.s.|08|THIN-LE-MOUTIER|   : .................C................................T....................T....................C....... :  700
AJ277030.1|PUUV|N|Belgium|M.g.|Thuin/33Cg/96|       : ........................................................A..............T........T...........C....... :  700
AJ238779.1|PUUV|N|Germany|M.g.|NRW/Cg-Erft|         : ........................................................G..............A........T.....T.....C.....T. :  700
2014.00276|PUUV|N|France|H.s.|59|LILLE|             : ..........................G.............................A..............T........T...........C....... :  700
U22423.1|PUUV|N|Belgium|M.g.|CG13891|               : ........T........C.................G..T...........T.....A........A.....C..C.....T..C..T..A..C....... :  700
2014.00321|PUUV|N|France|H.s.|59|FOURMIES|          : .......................................................................T............................ :  700
2015.00457|PUUV|N|France|H.s.|55|REVIGNY-SUR-ORNAI  : ........T...T.......G...........G..G....................A........T.....T........T...........C....... :  700
2013.00250|PUUV|N|France|H.s.|62|VENDIN-LS-BTHUNE|  : ............T......................G..............T.....G..............C..C.....T.....T.....C....... :  700
2016.00282|PUUV|N|France|H.s.|59|MORBECQUE|         : ........T...T......................G..............T.....G..............C..C.....T.....T.....C....... :  700
AJ277076.1|PUUV|N|Belgium|M.g.|Montbliart/CG14445|  : ....................G...................................A..............T.....C..T...........C....... :  700
2014.00153|PUUV|N|France|H.s.|02|BUIRONFOSSE|       : ....................G.................T.................A..............T.....C..T...........C....... :  700
2014.00171|PUUV|N|France|H.s.|59|SAINT-SAULVE|      : .....G..T........C.................G..............T.....G..............T..C..C........T............. :  700
2016.00293|PUUV|N|France|H.s.|60|LA-NEUVILLE-SUR-R  : ...................................G..A.....C...........G..............T........T.....T.....C....... :  700
2014.00499|PUUV|N|France|H.s.|94|CHAMPIGNY-SUR-MAR  : ........T...........G........A........T.................A..............T.....C..T...........C....... :  700
2012.00057|PUUV|N|France|H.s.|59|COUSOLRE|          : ....................G...................................A..............T.....C..T...........C....... :  700
2012.00349|PUUV|N|France|H.s.|02|ENGLANCOURT|       : ....................G........C........T.................A..............T.....C..T...........C....... :  700
2014.00135|PUUV|N|France|H.s.|02|ATHIES-SOUS-LAON|  : ........T...........G........A........T.................A..............T.....C..T...........C....... :  700
2014.00184|PUUV|N|France|H.s.|59|FOURMIES|          : ....................G...................................A..............T.....C..T...........C....... :  700
2016.00182|PUUV|N|France|H.s.|08|BOULZICOURT|       : ....................G...................................A..............T.....C..T...........C....... :  700
2016.00286|PUUV|N|France|H.s.|02|TRUCY|             : ........T...........G........A........T.................A..............T.....C..T...........C....... :  700
2016.00325|PUUV|N|France|H.s.|59|AULNOYE-AYMERIES|  : ....................G...................................A..............T.....C..T...........C....... :  700
2016.00452|PUUV|N|France|H.s.|02|LAON|              : ....................G........A........T.................A..............T.....C..T...........C....... :  700
2016.00467|PUUV|N|France|H.s.|02|AISNE|             : ....................G..C..............T.................A..............T.....C..T...........C....... :  700
```

```
                                                                 Bowen MD et al. J Med Virol 1997  TACCCICAITAICCIAA  PPT716R
                                                              *       620         *       640         *       660         *       680         *       700
2014.00598|PUUV|N|France|H.s.|59|FOURMIES|          : ATAGTATGCGGTCTATTTCCAACTCAAATTCAAGTACGCAACATAATGAGCCCTGTCATGGGAGTCATTGGGTTTTCTTTCTTTGTCAAGGATTGGGCAG : 700
2015.00657|PUUV|N|France|H.s.|02|CILLY|             : ...................................G.....T..............A.......................T.....G.....C.....T. : 700
2015.00526|PUUV|N|France|H.s.|08|MONTHERME|         : ....................G.................T.................A..............T.....C..T...........C....... : 700
2015.00045|PUUV|N|France|H.s.|59|FOURMIES|          : ....................G...................................A.......................T...........C....... : 700
2015.00430|PUUV|N|France|H.s.|02|MONTCORNET|        : ....................G.................T.................A..............T.....C..T...........C....... : 700
2016.00311|PUUV|N|France|H.s.|02|LAON|              : ........T...........G........A........T.................A..............T.....C..T...........C....... : 700
AJ277031.1|PUUV|N|Belgium|M.g.|Montbliard/23Cg/96|  : ....................G...................................A..............T.....C..T...........C....... : 700
2016.00326|PUUV|N|France|H.s.|02|LAON|              : ........T...........G........A........T.................A..............T.....C..T...........C....... : 700
2016.00353|PUUV|N|France|H.s.|02|CESSIERES|         : ........T...........G........A........T.................A..............T.....C..T...........C....... : 700
2012.00061|PUUV|N|France|H.s.|02|LANISCOURT|        : ........T...........G........A........T.................A..............T.....C..T...........C....... : 700
2014.00174|PUUV|N|France|H.s.|02|ST-ERME-OUTRE-ET-  : ........T...........G........A........T.................A..............T.....C..T...........C....... : 700
2016.00268|PUUV|N|France|H.s.|02|PRESLES-ET-THIERN  : ........T...........G........A........T.................A..............T.....C..T...........C....... : 700
2015.00660|PUUV|N|France|H.s.|02|VIC-SUR-AISNE|     : ........T...............................................G..............T.....C..T.....T.....C....... : 700
2012.00025|PUUV|N|France|H.s.|51|SAINTE-MENEHOULD|  : ........T..C...........C..............T..T........T.....T........A.....C.....C..T.....T.........A... : 700
2015.00665|PUUV|N|France|H.s.|51|REIMS|             : ........T..C...........C...........G..T..T........T.....T........A.....C.....C..T.....T.........A... : 700
2012.00307|PUUV|N|France|H.s.|54|COLOMBEY-LES-BELL  : ........T..CT.G........C.....C.....G..T...........T.....T........T.....C.....A........T............. : 700
2012.00123|PUUV|N|France|H.s.|55|BAR-LE-DUC|        : ........T..............C...........G..T..T........T.....T........A.....C........T.....T.........AT.. : 700
2012.00278|PUUV|N|France|H.s.|51|REIMS|             : ........T..C.......................G..T..T........T.....T........A.....C.....C..T.....T.....C...AT.. : 700
2015.00185|PUUV|N|France|H.s.|68|FELLERING|         : .....T..T..C...........C...........G..T..T........T.....T........A.....C........T.....T.........A... : 700
KJ994776.1|PUUV|N|Germany|M.g.|Mu/07/1219|2007|     : .....G..T..CT.G..C........G........G..A...........T.....G.....G..T..A..C.....A...................... : 700
2012.00086|PUUV|N|France|H.s|58|CHEVROCHES|         : ........T.....T........C..G..C.....G.....T........T...........G.....A..C........T.....T............. : 700
KT247595.1|PUUV|N|France|M.g.|45|Orleans/Mg29/2010  : .....G..T.....T........C..G..C.....G..T..T........T...........G.....A..C.....C..T.....T..A.......... : 700
KT247594.1|PUUV|N|France|M.g.|45|Orleans/Mg23/2010  : .....G..T.....T........C..G..C.....G..T..T........T...........G.....A..C.....C..T.....T..A.......... : 700
KY365004.1|PUUV|N|France|M.g.|45|Orleans/NCHA373/2  : ........T.....T........C..G..C.....G.....T........T...........G.....A..C.....C..T.....T..A.......... : 700
2012.00301|PUUV|N|France|H.s.|25|MOUTHE|            : ........T..C.......................G..A...........T.....G...........C........A..T.....T.........A... : 700
AM695638.1|PUUV|N|France|M.g.|Mignovillard/CgY02/2  : ........T..C.......................G..A...........T.....G...........C........A..T.....T.........A... : 700
KT247597.1|PUUV|N|France|M.g.|39|Jura/Mg214/2010|   : ........T.....T....................G..A..T........T.....G...........C........A..T.....T............. : 700
KT247596.2|PUUV|N|France|M.g.|39|Jura/Mg2/2010|     : ........T.....T....................G..A..T........T.....G...........C........A..T.....T............. : 700
2012.00102|PUUV|N|France|H.s.|39|COISERETTE|        : ........T.....T....................G..A..T........T.....G....................A..T..C..T.........A... : 700
2012.00536|PUUV|N|France|H.s.|39|LA-PESSE|          : ........T.....T....................G..A..T........T.....G....................A..T..C..T.........A... : 700
2014.00622|PUUV|N|France|H.s.|39|ARBOIS|            : ........T..C..........................A...........T.....G.....G..............A........T.........A... : 700
2015.00567|PUUV|N|France|H.s.|70|RIOZ|              : ........T.....T....................G..A...........T.....G...........C..A.....A..T.....T..A......A... : 700
2014.00637|PUUV|N|France|H.s.|25|SAULES|            : ........T.....T....................G..A...........T.....G...........C..A.....A..T.....T..A......A... : 700
2012.00396|PUUV|N|France|H.s.|39|SAINT-CLAUDE|      : ........T..C.......................G..A...........T.....G....................A........T.........A... : 700
2014.00120|PUUV|N|France|H.s.|38|LE-MOUTARET|       : ........T..C.......................G..A...........T.....G....................A........T.........A... : 700
2015.00153|PUUV|N|France|H.s.|73|GREZY-SUR-ISERE|   : ........T..C.......................G..A...........T.....G....................A........T.........A... : 700
2015.00504|PUUV|N|France|H.s.|70|RONCHAMP|          : ........T.....C...........G..c........A..T........T.....G.....G..............A..T...............A... : 700
2016.00275|PUUV|N|France|H.s.|21|JALLANGES|         : ........T.....C....................G..A...........T.....G...........C........A..T.....T..A......A... : 700
2016.00320|PUUV|N|France|H.s.|25|SAINT-VIT|         : ........T.....C....................G..A..T........T.....G...........C........A..T.....T............. : 700
2015.00410|PUUV|N|France|H.s.|70|ANGIREY|           : ..............C...........G..c........A..T........T.....G.....G..............A..T...............A..A : 700
DQ016430.2|PUUV|N|Germany|M.g.|Bavaria/CG33/04|     : .....C......T.............G...........T..T........T.....G...........C..T........T.....A.....C....... : 700
DQ016432.2|PUUV|N|Germany|M.g.|Bavaria/CG41/04|     : .....C......T.........................T..T........T.....G...........C..T........T.....A............. : 700
AY954723.2|PUUV|N|Germany|M.g.|Bavaria-CG34/04|     : .....C......T.........................T..T........T.....G...........C..T........T.....A............. : 700
AY954722.2|PUUV|N|Germany|M.g.|Bavaria-CG9/04|      : .....C......T.............G...........T..T........T.....G...........C..T..............A.....C....... : 700
2012.00402|PUUV|N|France|H.s.|60|GOUVIEUX|          : ........T.....T..C.....C...........G..A..T........T.....G...........C..T.....A..T.....T.........A... : 700
2014.00540|PUUV|N|France|H.s.|60|CHAMBLY|           : ........T.....T..C.................G..A..T........T.....G...........C..T.....A..T.....T.........A... : 700
AJ314600.1|PUUV|N|Balkan|M.g.|Balkan-1|             : ..T..G..T.....C..C.....C........G..G..T.....T.....T.....A.....G..T..A..A..C...........A.........T... : 700
AJ314601.1|PUUV|N|Balkan|M.g.|Balkan-2|             : ..T..G.TT.....C..C..............G..G.....T..T.....T.....G.....G..T..A..A..C.....T..C..A.........T... : 700
FN377821.1|PUUV|N|Hungary|M.g.|Mg9/HungaryTR17/00|  : ..T..G..T.....C..C..............G..G..T..T..T.....T.....G.....G..T..A..A..C.....T.....A.........T... : 700
FN377822.1|PUUV|N|Hungary|M.g.|Mg23/HungaryTR17/00  : ..T..G..T.....C..C.................G.....T..T.....T.....G.....G..T..A..A..C...........A.........T... : 700
GQ339473.1|PUUV|N|Sweden|M.g.|Kiviniemi/Mg3/05|     : .....T..T..GT..........A..G..C.....G..A..T........T..A..A.....G..G.....C..............T..A..G....... : 700
GQ339474.1|PUUV|N|Sweden|M.g.|Kiviniemi/Mg5/05|     : .....T..T..GT..........A..G..C.....G..A..T........T..A..A.....G..G.....C..............T..A..G....... : 700
GQ339475.1|PUUV|N|Sweden|M.g.|Kiviniemi/Mg6/05|     : .....T..T..GT..........A..G..C.....G..A..T........T..A..A.....G..G.....C..............T..A..G....... : 700
GQ339476.1|PUUV|N|Sweden|M.g.|Aijajarvi/Mg7/05|     : .....T..T..GT..........A..G..C.....G..A..T........T..A..A.....G..G.....C..............T..A..G....... : 700
```

```
                                                            Bowen MD et al. J Med Virol 1997  TACCCICAITAICCIAA  PPT716R
                                                    *         620         *         640         *         660         *         680         *         700
2014.00598|PUUV|N|France|H.s.|59|FOURMIES|         : ATAGTATGCGGTCTATTTCCAACTCAAATTCAAGTACGCAACATAATGAGCCCTGTCATGGGAGTCATTGGGTTTTCTTTCTTTGTCAAGGATTGGGCAG :  700
GQ339477.1|PUUV|N|Sweden|M.g.|Aijajarvi/Mg9/05|    : .....T..T..GT..........A..G..C.....G..A..T........T..A..A.....G..G.....C..............T..A.G........ :  700
GQ339478.1|PUUV|N|Sweden|M.g.|Jockfall/Mg12/05|    : .....T..T..GT.G.....G..A..G..C..G..G..A..T........T..A..A........G.....C..............T..A.G........ :  700
GQ339482.1|PUUV|N|Sweden|M.g.|Kalvudden/Mg22/05|   : .....T..T..GT.G........A..G..C.....G..A..T........T..A..A.....G..G.....C..............T..A.G........ :  700
GQ339479.1|PUUV|N|Sweden|M.g.|Moskosel/Mg17/05|    : .....T.....GT.......T..A..G..C.....G..A..T........T..A..A........G.....A...G..........T..A.G........ :  700
GQ339481.1|PUUV|N|Sweden|M.g.|Ljustrask/Mg20/05|   : .....T.....GT.G.....T..A..G..C........A...........T..A..A........G.....A...G..........T..A.G........ :  700
GQ339480.1|PUUV|N|Sweden|M.g.|Gyttjea/Mg19/05|     : .....T.....GT.G.....T..C..G..C.....G..A..T........T..A..A........G.....A...G....T.....T..A.G........ :  700
AY526219.1|PUUV|N|Sweden|Human|Umea/hu|            : .....T..T..CT.......T..A...........T..A..T........T..A..A.....G..G.....C.....C........T.....C...C... :  700
AJ223380.1|PUUV|N|Sweden|M.g.|Tavelsjo/Cg81/94|    : .....C..T..CT....C..T..A...........T..A..T........T..A..A........A.....C..C...........T.....C....... :  700
U14137.1|PUUV|N|Bosnia-H.|M.g.|Vranica|            : .....C..T..CT....C..T..A...........T..A..T........T..A..A........A.....C..C...........T.....C....... :  700
AJ223371.1|PUUV|N|Sweden|M.g.|Huggberget/Cg36/94|  : .....C..T..CT....C..T..A...........C..G..T........T..A..A........G.....C..C...........T............. :  700
Z48586.1|PUUV|N|Sweden|M.g.|Vindeln/L20Cg/83|      : .....C..T..CT....C..T..A...........C..A...........T..A..A........G.....C..............T............. :  700
AJ223374.1|PUUV|N|Sweden|M.g.|Mellansel/Cg47/94|   : ........T..CT....C..T..A...........T..A..T........T..A..A.....G..G.....C.....C........T.....C....... :  700
AJ223375.1|PUUV|N|Sweden|M.g.|Mellansel/Cg49/94|   : ........T..CT....C..T..A...........T..A..T........T..A..A.....G..G.....C.....C........T.....C....... :  700
AJ238791.1|PUUV|N|Denmark|M.g.|Fyn/19|             : ............T....C.....A...........C.....T........T...........G..G.....C..C..C........T...........T. :  700
AJ278092.1|PUUV|N|Denmark|M.g.|Fyn47|              : ............T....C.....A...........C.....A........T.....A.....G..T..A..C.....C..T..C..T..........TT. :  700
AJ278093.1|PUUV|N|Denmark|M.g.|Fyn131|             : ............T....C.....A...........C.....T........T.....G.....G..T..A..C.C...C..T..C..T...........T. :  700
AJ223368.1|PUUV|N|Norway|M.g.|Eidsvoll/1124v|      : ........T..GT.G.....C.....G..C.....G.....T..............T.....G..T.....C..C......................... :  700
AJ223369.1|PUUV|N|Norway|M.g.|Eidsvoll/Cg1138/87|  : ........T..GT.G.....C.....G..C.....G.....T..............T.....G..T.....C..C......................... :  700
JN657228.1|PUUV|N|Latvia|M.g.|Jelgava/Mg149/2008|  : ..T..T..T..CT.............G..A.....G........C.....T..A..A.....T........C..C.....T.....A.....C....... :  700
KX757839.1|PUUV|N|Lithuania|M.g.|LT15/164|2015|    : ..T..T..T...T.G..C........G..A..G..G..T.....C.....T..A..A.....T........A..C..C..T.....G..A..C....... :  700
KX757840.1|PUUV|N|Lithuania|M.g.|LT15/174|2015|    : ..T..T..T...T.G..C........G..A..G..G..T.....C.....T..A..A.....T........A.....C..T.....G..A..C.....G. :  700
KX815394.1|PUUV|N|Poland|M.g.|KS13/855|2009|       : ..T..T..T..C..G..C........G..A.....G.....T..C.....T..A..A.....T..T.....A..C.....T..C..G............. :  700
KX757841.1|PUUV|N|Lithuania|M.g.|LT15/201|2015|    : ..T..T..T...T.G..C........G..A..G..G..T.....C.....T..A..A.....T........A..C..C..T.....G..A..C....... :  700
AJ314598.1|PUUV|N|Russia|M.g.|Baltic/49Cg/00|      : ........T........C..T........A..............C.....T..A..G.....G..A.....T.....C..T.....T..A..C...C... :  700
AJ314599.1|PUUV|N|Russia|M.g.|Baltic/205Cg/00|     : ........T.....G..C..T........A........T..T..C.....T..A..G.....G..A.....T.....C..T........A..C...C... :  700
JN657229.1|PUUV|N|Latvia|M.g.|Madona/Mg99/2008|    : ........T..C.....C..T..C.....A..............C.....T..A..A.....G........C.....G........T..A..C...C... :  700
JN657232.1|PUUV|N|Latvia|M.g.|Madona/Mg233/2008|   : ........T..C.....C..T..C.....A..............C.....T..A..A.....G........C.....G........T..A..C...C... :  700
JN657230.1|PUUV|N|Latvia|M.g.|Jelgava/Mg136/2008|  : G.......T..C.....C..T..C.....A..............C.....T..G..G.....G........C.....G...........A..C...C... :  700
JN657231.1|PUUV|N|Latvia|M.g.|Jelgava/Mg140/2008|  : G.......T..C.....C.AT..C.....A..............C.....T..G..G.....G........C.....G...........A..C...C... :  700
Z30707.1|PUUV|N|Russia|M.g.|Udmurtia/458Cg/88|     : ........T..CT.......T..A..G..A.....G..T.....C.....T..A..A........A.....T.................A..C...C... :  700
Z30706.1|PUUV|N|Russia|M.g.|Udmurtia/444Cg/88|     : ........T..CT.......T..A..G..A.....G..T.....C.....T..A..A........G.....T.................A..C...C... :  700
Z84204.1|PUUV|N|Russia|M.g.|Kazan|                 : ........T..CT.......T..A..G..A.....G..T.....C.....T..A..A........G.....T.................A..C...C... :  700
Z30708.1|PUUV|N|Russia|M.g.|Udmurtia/338Cg/92|     : ........T..CT.......T..A..G..A.....G..T.....C.....T..A..A........G.....T.................A..C...C... :  700
Z21497.1|PUUV|N|Russia|M.g.|Udmurtia/894Cg/91|     : ........T..CT.......T..A..G..A.....G..T.....C.....T..A..A........G.....T.................A..C...C... :  700
AB433843.2|PUUV|N|Russia|M.g.|Samara_49/CG/2005|   : ........T..CT....C..T..A..G..A.....G..T.....C.....T..A..G........G.....T..............T..A..C...C... :  700
AB433845.2|PUUV|N|Russia|M.g.|Samara_94/CG/2005|   : ........T..CT.G..C..T..A..G..A.....G..T.....C.....T..A..G........G.....T..............T..A..C...C.G. :  700
L11347.1|PUUV|N|Russia|Human|P360|                 : ........T..CT....C..T..A..G..A.....T..T.....C.....T..A..A........G.....T...........C..T..A..C...C... :  700
AB297665.2|PUUV|N|Russia|M.g.|DTK/Ufa-97|1997|     : ........T..CT....C..T..A..G..A.....T..T.....C.....T..A..A........G.....T...........C..T..A..C...C... :  700
M32750.1|PUUV|N|Russia|M.g.|CG1820|                : ........T..CT....C..T..A..G..A.....T..T.....C.....T..A..A........G.....T...........C..T..A..C...C... :  700
AF442613.1|PUUV|N|Russia|M.g.|CG17/Baskiria-2001|  : ........T..CT....C..T..A..G..A.....T..T.....C.....T..A..A........G.....T..............T..A..C...C... :  700
KX815395.1|PUUV|N|Poland|M.g.|KS14/118|2009|       : .....G..T..CT....C..T........A..G..G..T..T..C........G..A.....G..A.....C.....A........T...A.C...C... :  700
GQ339483.1|PUUV|N|Sweden|M.g.|Bergsjobo/Mg25/05|   : ........T...........T.....G..............T........T..A........G..T.....C............................ :  700
AJ223377.1|PUUV|N|Sweden|M.g.|Solleftea/Cg6/95|    : ........T...........T.....G..............T........T..A........G..T.....C..........................T. :  700
GQ339484.1|PUUV|N|Sweden|M.g.|Faboviken/Mg26/05|   : ........T...T.......T.....G........G..T..T........T..A..T.....G..T.....C..............T..A.......... :  700
GQ339485.1|PUUV|N|Sweden|M.g.|Mangelbo/Mg1/05|     : ........T..C........C..C..G........G..T..T........T..A..T.....G........C............................ :  700
GQ339486.1|PUUV|N|Sweden|M.g.|Munga/Mg2/05|        : ........T..C.....C..T..C..G...........T..T........T..A..T.....G........C............................ :  700
GQ339487.1|PUUV|N|Sweden|M.g.|Munga/Mg16/05|       : ........T..C.....C..T..C..G...........T..T........T..A..T.....G........C............................ :  700
AJ223376.1|PUUV|N|Sweden|M.g.|Solleftea/Cg3/95|    : ........T...T.......T.....G........G..T..T........T..A..T.....G..T..C..C..............T..A.......... :  700
AF367071.1|PUUV|N|Russia|M.r.|CRF366|              : ........T..AT.G........C..G..C..G..T........T........A..T.....G.....A..C..C..A........T.....C...C... :  700
AF367064.1|PUUV|N|Russia|M.g.|CG144|               : ........T..AT.G........C..G..C..G..T........T........A..T.....G.....A..C..C..A........T.....C...C... :  700
AF367065.1|PUUV|N|Russia|M.g.|CG168|               : ........T..AT.G........C..G..C..G..T........T........A..T.....G.....A..C..C..A........T.....C...C... :  700
AF367068.1|PUUV|N|Russia|M.g.|CG315|               : ........T..AT.G........C..G..C..G..T........T........A..T.....G.....A..C..C..A........T.....C...C... :  700
AF367069.1|PUUV|N|Russia|M.r.|CRF161|              : ........T..AT.G........C..G..C..G..T........T........A..T.....G.....A..C..C..A........T.....C...C... :  700
```

```
                                                                             Bowen MD et al. J Med Virol 1997  TACCCICAITAICCIAA  PPT716R
                                                               *       620         *       640         *       660         *       680         *       700
2014.00598|PUUV|N|France|H.s.|59|FOURMIES|          : ATAGTATGCGGTCTATTTCCAACTCAAATTCAAGTACGCAACATAATGAGCCCTGTCATGGGAGTCATTGGGTTTTCTTTCTTTGTCAAGGATTGGGCAG :  700
AF367070.1|PUUV|N|Russia|M.r.|CRF308|               : ........T..AT.G........C..G..C..G..T........T........A..T.....G.....A..C..C..A........T.....C...C... :  700
AF367066.1|PUUV|N|Russia|M.g.|CG215|                : ........T..AT.G........C..G..C..G..T........T........A..T.....G.....A..C..C..A........T.....C...C... :  700
AF367067.1|PUUV|N|Russia|M.g.|CG222|                : ........T..AT.G........C..G..C..G..T........T........A..T.....G.....A..C..C..A........T.....C...C... :  700
Z46942.1|PUUV|N|Finland|M.g.|Puumala/1324Cg/79|     : ........T.................G..C.....T..T.....C.....T..A..T.....G.....C..T.....A..T.....G..A......C... :  700
Z30702.1|PUUV|N|Finland|M.g.|Evo/12Cg/93|           : ........T.....T...........G..C.....T..T.....C.....T..A..T.....G..T........C..A..T.....A.........C.T. :  700
Z30703.1|PUUV|N|Finland|M.g.|Evo/13Cg/93|           : ........T.....T...........G..C.....T..T.....C.....T..A..T.....G..T........C..A..T.....A.........C.T. :  700
Z30704.1|PUUV|N|Finland|M.g.|Evo/14Cg/93|           : ........T.....T...........G..C.....T..T.....C.....T..A..T.....G..T.....T..C..A..T.....A..A......C.T. :  700
Z30705.1|PUUV|N|Finland|M.g.|Evo/15Cg/93|           : ........T.....T...........G..C.....T..T.....C.....T..A..T.....G..T........C..A..T.....A..A......C.T. :  700
Z69985.1|PUUV|N|Finland|M.g.|Virrat/25Cg/95|        : .....C..T.....C..C........G........T..T.....C.....T..A..T.....G..T.....T.....A..T.....A..A......T.T. :  700
JN831947.1|PUUV|N|Finland|Pieksamaki/human_lung/20 : ........T.................G..C.....T..T.....C.....T..A..T.....G..T..C..T.....A..T.....G..A......C... :  700
JN831943.1|PUUV|N|Finland|Pieksamaki/Mg7/2008|      : ........T.................G..C.....T..T.....C.....T..A..T.....G..T..C..T.....A..T.....G..A......C... :  700
JN831950.1|PUUV|N|Finland|Pieksamaki/human_kidney/ : ........T.................G..C.....T..T.....C.....T..A..T.....G..T..C..T.....A..T.....G..A......C... :  700
JQ319166.1|PUUV|N|Finland|Konnevesi/Mg_O14B/2005|   : ........T.................G..C.....T..T.....C.....T..A........G..T.....T.....A..T.....A..A......C... :  700
JQ319169.1|PUUV|N|Finland|Konnevesi/Mg_O27B/2005|   : ........T.................G..C.....T..T.....C.....T..A........G..T.....T.....A..T.....A..A......C... :  700
JQ319167.1|PUUV|N|Finland|Konnevesi/Mg_O15B/2005|   : ........T.................G..C.....T..T.....C.....T..A........G..T.....T.....A..T.....G..A......C... :  700
JQ319164.1|PUUV|N|Finland|Konnevesi/Mg_O6B/2005|    : ........T.................G..C.....T..T.....C.....T..A........G..T.....T.....A..T.....A..A......C... :  700
JQ319165.1|PUUV|N|Finland|Konnevesi/Mg_O9B/2005|    : ........T.................G..C.....T..T.....C.....T..A........G..T.....T.....A..T.....A..A......C... :  700
JQ319170.1|PUUV|N|Finland|Konnevesi/Mg_O74B/2005|   : ........T.................G..C.....T..T.....C.....T..A........G..T.....T.....A..T.....A..A......C... :  700
JQ319171.1|PUUV|N|Finland|Konnevesi/Mg_M114B/2005|  : ........T.................G..C.....T..T.....C.....T..A........G..T.....T.....A..T.....G..A......C... :  700
AJ238788.1|PUUV|N|Russia|M.g.|Karhumaki|            : ........T.................G..C.....T..T.....C.....T..A..T.....G..T.....T..C..A..T........A......T... :  700
AJ238789.1|PUUV|N|Russia|M.g.|Kolodozero|           : ........T......A....G.....G........T..T.....C.....T..A..T.....G..T...........A.....C..A.........T... :  700
AJ314597.1|PUUV|N|Finland|M.g.|Pallasjarvi/63Cg/98 : ........T.................G........C..T.....C.....T..A..A.....G..T...........A..T.....A.........T... :  700
NC_005224.1|PUUV|N|Finland|M.g.|Sotkamo-revu-NCBI2 : ........T.....T.....C.....G..C..G..T..T.....C.....T..A..T.....G........T.....A........G.........T.T. :  700
AJ238790.1|PUUV|N|Russia|M.g.|Gomselga|             : ........T...........C.....G..C.....T..T..T..CG.C..T.....T.....G........T.....A........G..A..C...T..C :  700
AB010731.1|Hokkaido-V|N|Japan|M.r.|Tobetsu-60Cr-93  : .....C..T..C..T..C..G..A...........T..G..T........T...........G..T.....A..C..A........A..A..C...C.T. :  700
AB010730.1|Hokkaido-V|N|Japan|M.r.|Kamiiso-8Cr-95|  : .....C..T..C..T..C..G..A...........T..G..T........T...........G..T.....A..C..A........T..A..C...C.T. :  700
JX046487.1|Muju-V|N|South-Korea|M.r.|11-5|2011      : ..T.....T..A........T..A...........TA.A..T........T..G..G........G.....C..............A..A........T. :  700
JX046484.1|Muju-V|N|South-Korea|M.r.|11-4|2011|     : ..T.....T..A........T..A...........TA.A..T........T..G..G........G.....C..............A..A........T. :  700
JX028273.1|Muju-V|N|South-Korea|M.r.|11-1|2011|     : ..T.....T..A........T..A...........TA.A..T........T..A..G........G.....C........T.....A..A........T. :  700
DQ138128.1|Muju-V|N|South-Korea|E.r.|00-18|         : ..T..G..T...T.G..C.....A..G..C..G..GA.A.....C.....T.....A........A..A........C........T..A.......TT. :  700
DQ138140.1|Muju-V|N|South-Korea|E.r.|99-27|         : ..T..G..T...T.G..C.....A..G..C..G..GA.A.....C.....T.....A........G..A........C........T..A.......TT. :  700
DQ138133.1|Muju-V|N|South-Korea|E.r.|96-1|          : ..T..G..T...T.G..C.....A..G..C..G..GA.A.....C.....T.....A........A..AA.......C.....G..T..A......T.T. :  700
DQ138142.1|Muju-V|N|South-Korea|E.r.|99-28|         : ..T..G..T...T.G..C.....A..G..C..G..GA.A.....C.....T.....A........G..A........C........T..A.......TT. :  700
```

```
                                                                 *       720         *       740         *       760         *       780         *       800
2014.00598|PUUV|N|France|H.s.|59|FOURMIES|          : ATCGAATTAGGGAATTCATGGAAAAAGAGTGCCCTTTTATTAAACCAGAGGTGAAGCCAGGAACACCAGCCCAGGAAGCTGAATTCCTAAAGAGAAACAA :  800
2014.00233|PUUV|N|France|H.s.|59|FOURMIES|          : .C........A..................................C..C.................................................. :  800
2014.00097|PUUV|N|France|H.s.|02|SAINT-MICHEL|      : ........................................C......................................A.................... :  800
2014.00488|PUUV|N|France|H.s.|08|SIGNY-LE-PETIT|    : ........................................C........................................................... :  800
2014.00613|PUUV|N|France|H.s.|59|FOURMIES|          : ........................................C........................................................... :  800
2015.00402|PUUV|N|France|H.s.|08|CHARLEVILLE-MEZIE  : ....G...................................C.....G..A............................................G..... :  800
2015.00422|PUUV|N|France|H.s.|59|ANOR|              : .................................................................................................... :  800
2015.00488|PUUV|N|France|H.s.|08|SEDAN|             : .C..G...................................C.....G..A............................................G..... :  800
2015.00498|PUUV|N|France|H.s.|08|REMILLY-AILLICOUR  : ........................................C........................................................... :  800
2016.00295|PUUV|N|France|H.s.|08|CORNY-MACHEROMENI  : ................T....................C..C..............A.............................TT............. :  800
2016.00333|PUUV|N|France|H.s.|59|FOURMIES|          : ........................................C........................................................... :  800
2016.00345|PUUV|N|France|H.s.|59|FOURMIES|          : ........................................C........................................................... :  800
2016.00357|PUUV|N|France|H.s.|02|HIRSON|            : ........................................C........................................................... :  800
2016.00427|PUUV|N|France|H.s.|59|FOURMIES|          : ........................................C........................................................... :  800
2016.00469|PUUV|N|France|H.s.|02|PREMONTRE|         : ....G...........T........G..............C...........A.............................G...........G..T.. :  800
AJ277075.1|PUUV|N|Belgium|M.g.|Montbliart/CG14444|  : ....G...................................C......................................................G..... :  800
2014.00053|PUUV|N|France|H.s.|08|SECHEVAL|          : ....G...................................C.....G..A............................................G..... :  800
KT247593.1|PUUV|N|France|M.g.|08|Ardennes/Mg75/201  : ....G...................................C.....................................T...............G..... :  800
KT247592.1|PUUV|N|France|M.g.|08|Ardennes/Mg156/20  : ....G...................................C.....................................T...............G..... :  800
2015.00019|PUUV|N|France|H.s.|08|ETEIGNIERES|       : ....G...................................C......................................................G..... :  800
2016.00239|PUUV|N|France|H.s.|08|VIREUX-MOLHAIN|    : ....G....A..............................C.................T..................................AG..... :  800
2015.00329|PUUV|N|France|H.s.|08|BOGNY-SUR-MEUSE|   : ....G...................................C...........................A....T........................T.. :  800
2014.00209|PUUV|N|France|H.s.|08|TREMBLOIS-LES-ROC  : ....G...................................C......................................................G..... :  800
2015.00456|PUUV|N|France|H.s.|94|ALFORTVILLE|       : ....G...................................C......................................................G..... :  800
2012.00018|PUUV|N|France|H.s.|08|ROCROI|            : ....G...........T.......................C................................A.....................G..... :  800
AJ277032.1|PUUV|N|Belgium|M.g.|Momignies/47Cg/96|   : ....G.............................C.....C......................................................G..... :  800
AJ277034.1|PUUV|N|Belgium|M.g.|Couvin/59Cg/97|      : ....G........G..........................C......................................................G..... :  800
AJ277033.1|PUUV|N|Belgium|M.g.|Momignies/55Cg/96|   : ....G.............................C.....C......................................................G..... :  800
2012.00638|PUUV|N|France|H.s.|08|GIVET|             : ....G...................................C.............................................T.......G..... :  800
2015.00328|PUUV|N|France|H.s.|08|VRIGNE-MEUSE|      : ....G...................................C..........................G.T.........................G..... :  800
2015.00419|PUUV|N|France|H.s.|08|DOUZY|             : ....G...................................C..........................G.T......................A..G..... :  800
2016.00310|PUUV|N|France|H.s.|08|THIN-LE-MOUTIER|   : ................T....................C..C...........A................T...............TT............. :  800
AJ277030.1|PUUV|N|Belgium|M.g.|Thuin/33Cg/96|       : ....G...........T...........A...........C...........A................T.............................. :  800
AJ238779.1|PUUV|N|Germany|M.g.|NRW/Cg-Erft|         : .C..G....AAA................A.......................A........G......T.T..............T.......TG..T.G :  800
2014.00276|PUUV|N|France|H.s.|59|LILLE|             : ....G....................G..A...........C.....................................A........T............. :  800
U22423.1|PUUV|N|Belgium|M.g.|CG13891|               : ....G.....A....CT.................C...C.A........T..A........G...............A.C.....TT.......G..T.. :  800
2014.00321|PUUV|N|France|H.s.|59|FOURMIES|          : ........................................C..G...................T.................................... :  800
2015.00457|PUUV|N|France|H.s.|55|REVIGNY-SUR-ORNAI  : ....G...........T........G..............A.........T.A.............................G...........G..T.. :  800
2013.00250|PUUV|N|France|H.s.|62|VENDIN-LS-BTHUNE|  : ....GG.......G........G.................C..G........T..............G........G.................G..... :  800
2016.00282|PUUV|N|France|H.s.|59|MORBECQUE|         : ....TG.....................................G.......................G........G.................G..... :  800
AJ277076.1|PUUV|N|Belgium|M.g.|Montbliart/CG14445|  : ....G....................G..............C......................................................G....T :  800
2014.00153|PUUV|N|France|H.s.|02|BUIRONFOSSE|       : ....G...................................C...................................G.................G..... :  800
2014.00171|PUUV|N|France|H.s.|59|SAINT-SAULVE|      : ....T..C..A.....T...................................A..A..............................T............. :  800
2016.00293|PUUV|N|France|H.s.|60|LA-NEUVILLE-SUR-R  : .......C........T...........A..T........C..............................T...............T.G.....G..... :  800
2014.00499|PUUV|N|France|H.s.|94|CHAMPIGNY-SUR-MAR  : ....G...................................C...................................G.................G..... :  800
2012.00057|PUUV|N|France|H.s.|59|COUSOLRE|          : ....G...................................C.............................................T.......G..... :  800
2012.00349|PUUV|N|France|H.s.|02|ENGLANCOURT|       : ....G...................................C...........A.......................G.................G..... :  800
2014.00135|PUUV|N|France|H.s.|02|ATHIES-SOUS-LAON|  : ....G...................................C...................................G.................G..... :  800
2014.00184|PUUV|N|France|H.s.|59|FOURMIES|          : ....G...................................C......................................................G..... :  800
2016.00182|PUUV|N|France|H.s.|08|BOULZICOURT|       : ....G.....A.............................C......................................................G..... :  800
2016.00286|PUUV|N|France|H.s.|02|TRUCY|             : ....G...................................C...................................G.................G..... :  800
2016.00325|PUUV|N|France|H.s.|59|AULNOYE-AYMERIES|  : ....G........G....................C.....C......................................................G..... :  800
2016.00452|PUUV|N|France|H.s.|02|LAON|              : ....G...................................C...................................G.................G..... :  800
2016.00467|PUUV|N|France|H.s.|02|AISNE|             : ....G.......................................................................G..C..............G..... :  800
```

```
                                                              *       720         *       740         *       760         *       780         *       800
2014.00598|PUUV|N|France|H.s.|59|FOURMIES|         : ATCGAATTAGGGAATTCATGGAAAAAGAGTGCCCTTTTATTAAACCAGAGGTGAAGCCAGGAACACCAGCCCAGGAAGCTGAATTCCTAAAGAGAAACAA :  800
2015.00657|PUUV|N|France|H.s.|02|CILLY|            : ....G..C..A.........  ..G.................C...........A........G...........................TT.G.....G..T.. :  800
2015.00526|PUUV|N|France|H.s.|08|MONTHERME|        : ....G...................................C...................................G.T...............G..... :  800
2015.00045|PUUV|N|France|H.s.|59|FOURMIES|         : ....G...................................C.............................T.......................G..... :  800
2015.00430|PUUV|N|France|H.s.|02|MONTCORNET|       : ....G...................................C................................T..G.................G..T.. :  800
2016.00311|PUUV|N|France|H.s.|02|LAON|             : ....G...................................C.................G.................G.................G..... :  800
AJ277031.1|PUUV|N|Belgium|M.g.|Montbliart/23Cg/96| : ....G...................................C.............................................T...G...G..... :  800
2016.00326|PUUV|N|France|H.s.|02|LAON|             : ....G...................................C...................................G.................G..... :  800
2016.00353|PUUV|N|France|H.s.|02|CESSIERES|        : ....G...................................C...................................G.................G..... :  800
2012.00061|PUUV|N|France|H.s.|02|LANISCOURT|       : ....G................................C..C...................................G.................G..... :  800
2014.00174|PUUV|N|France|H.s.|02|ST-ERME-OUTRE-ET- : ....G...................................C...................................G.................G..... :  800
2016.00268|PUUV|N|France|H.s.|02|PRESLES-ET-THIERN : ....G...................................C...................................G.................G..... :  800
2015.00660|PUUV|N|France|H.s.|02|VIC-SUR-AISNE|    : ....G.................C..G...........C..C...........C................................T..G.....G..... :  800
2012.00025|PUUV|N|France|H.s.|51|SAINTE-MENEHOULD| : ....G..C..A.....T.....T..G..A.......................C..A.....G...........A....T...G...T.G..A.AG..T.. :  800
2015.00665|PUUV|N|France|H.s.|51|REIMS|            : ....G.....A...........T..G..A........C.....G........C..A.....G..................T...G.........A.AG..T.. :  800
2012.00307|PUUV|N|France|H.s.|54|COLOMBEY-LES-BELL : ....GG..........T.....T..G..A...........C..G........T..A.....G.....G..T.......T...G...T....A.AG..... :  800
2012.00123|PUUV|N|France|H.s.|55|BAR-LE-DUC|       : ....G..C..A.....T.....T..G..A.......................C..A.....G..................T...G...T.G..A.AG..... :  800
2012.00278|PUUV|N|France|H.s.|51|REIMS|            : .C..G.....A.....T.....T..G..A..............G........C..A.....G...........A....T...G.........A.AG..T.. :  800
2015.00185|PUUV|N|France|H.s.|68|FELLERING|        : ....G..C..A.....T.....T..G..A........C..............T..A.....G........T.......T...G...T.G..A.AG..T.. :  800
KJ994776.1|PUUV|N|Germany|M.g.|Mu/07/1219|2007|    : .A..G.....A..G..T...........A.....C.................C.................A.....G........TT..........T.. :  800
2012.00086|PUUV|N|France|H.s|58|CHEVROCHES|        : ....G........G.................T.....C...........A..C..A.....G.....G.T.A.A........G...T...GA.AG..T.G :  800
KT247595.1|PUUV|N|France|M.g.|45|Orleans/Mg29/2010 : ....G....A...G...........G..A..T.....C...........A..C..A.....G.......T.A.A........G...T....A.AG....G :  800
KT247594.1|PUUV|N|France|M.g.|45|Orleans/Mg23/2010 : ....G....A...G...........G..A..T.....C...........A..C..A.....G.......T.A.A........G...T....A.AG....G :  800
KY365004.1|PUUV|N|France|M.g.|45|Orleans/NCHA373/2 : ....G....A...G..............A..T.....C...........A..C..A.....G.......T.A.A........G...T....A.AG....G :  800
2012.00301|PUUV|N|France|H.s.|25|MOUTHE|           : ....G...........T........G..A.....C.....A.........A....A..G..............A............T.......G..T.. :  800
AM695638.1|PUUV|N|France|M.g.|Mignovillard/CgY02/2 : ....G...........T........G..A.....C.....A.........A....A..G..............A............T.......G..T.. :  800
KT247597.1|PUUV|N|France|M.g.|39|Jura/Mg214/2010|  : .C..G...........T.....G..G........C...............A.A..A..G...........T...............T.......G..T.. :  800
KT247596.2|PUUV|N|France|M.g.|39|Jura/Mg2/2010|    : .C..G...........T.....G..G........C...............A.A..A..G...........T...............T.......G..T.. :  800
2012.00102|PUUV|N|France|H.s.|39|COISERETTE|       : .C..G...........T........G..A.....C...............A....A..G...........................T.......G..T.. :  800
2012.00536|PUUV|N|France|H.s.|39|LA-PESSE|         : .C..G...........T........G..A.....C...............A....A..G...........................T.......G..T.. :  800
2014.00622|PUUV|N|France|H.s.|39|ARBOIS|           : .C..G...........T........G..A.....C...............A....A..G..............A............T.......G..T.. :  800
2015.00567|PUUV|N|France|H.s.|70|RIOZ|             : .C..G...........T.....G..G..A.....C...............A....A..G........................C..T.......G..T.. :  800
2014.00637|PUUV|N|France|H.s.|25|SAULES|           : .C..G...........T.....G..G..A.....C...............A....A..G........................C..T.......G..T.. :  800
2012.00396|PUUV|N|France|H.s.|39|SAINT-CLAUDE|     : .C..G...........T........G..A.....A...............A....A.................A............T.......G..T.. :  800
2014.00120|PUUV|N|France|H.s.|38|LE-MOUTARET|      : .C..GG..........T........G..A.....................A....A.................A............T.......G..T.. :  800
2015.00153|PUUV|N|France|H.s.|73|GREZY-SUR-ISERE|  : .C..GG..........T........G..A.....................A....A.................A............T.......G..T.. :  800
2015.00504|PUUV|N|France|H.s.|70|RONCHAMP|         : .C..GG.C........T.....G..G..A.....................A.A.................T..............TT..........T.. :  800
2016.00275|PUUV|N|France|H.s.|21|JALLANGES|        : .C..G...........T.....G..G..A.....C..............TA.A..A..G...........................T.......G..T.. :  800
2016.00320|PUUV|N|France|H.s.|25|SAINT-VIT|        : .C..G...........T.....G..G........C...............A.A..A..............................T.......G..T.. :  800
2015.00410|PUUV|N|France|H.s.|70|ANGIREY|          : .C..GG.C..............G..G..A.....................A.A.....G...........T..............TT..........T.. :  800
DQ016430.2|PUUV|N|Germany|M.g.|Bavaria/CG33/04|    : .A..T...........T.....G.....A.....C..C..C........AA.A..A.....G........A.....G.........T.G........T.. :  800
DQ016432.2|PUUV|N|Germany|M.g.|Bavaria/CG41/04|    : .A..T.....A.....T.....G.....A.....C..C..C........AA....A.....G........A.....G.....G...T..........T.. :  800
AY954723.2|PUUV|N|Germany|M.g.|Bavaria-CG34/04|    : .A..T.....A.....T.....G.....A.....C..C..C........AA....A.....G........A.....G.....G...T..........T.. :  800
AY954722.2|PUUV|N|Germany|M.g.|Bavaria-CG9/04|     : .A..T...........T.....G.....A.....C..C..C........AA....A.......T......A.....G.....G...T..........T.. :  800
2012.00402|PUUV|N|France|H.s.|60|GOUVIEUX|         : .C..GG..........T........G..A.....C..C..C..G...........A.....G.....C.TT...............T.G........T.. :  800
2014.00540|PUUV|N|France|H.s.|60|CHAMBLY|          : ....G...........T...........A.....C..C..C..G........A..A.....G.....T.TT...............T..........T.. :  800
AJ314600.1|PUUV|N|Balkan|M.g.|Balkan-1|            : .GA.GG.G..A.....T.....G.....A.....C.....A.........A.T........G.....CC.G.....G.....G...T....A.....T.. :  800
AJ314601.1|PUUV|N|Balkan|M.g.|Balkan-2|            : .GA.GG.G..A.....T.....G.....A...........A.........A.T........GG....CC.G.....G.....G...T....A.....T.. :  800
FN377821.1|PUUV|N|Hungary|M.g.|Mg9/HungaryTR17/00| : .GA.GG.G..A.....T.....G.....A.....C.....A.........A.T........G.....CC.G.....G.....G...T....A.....T.. :  800
FN377822.1|PUUV|N|Hungary|M.g.|Mg23/HungaryTR17/00 : .GA.GG.G..A.....T.....G.....A...........A.........A.T..A.....G.....CC.G.....G.....G...T....A.....T.. :  800
GQ339473.1|PUUV|N|Sweden|M.g.|Kiviniemi/Mg3/05|    : .CA.G..C..A..G...........G........G.....A..G........C...............A.A.....G.T.......T.G....A...T.. :  800
GQ339474.1|PUUV|N|Sweden|M.g.|Kiviniemi/Mg5/05|    : .CA.G..C..A..G...........G..A.....G.....A..G........C...............A.A.....G.T.......T......A...T.. :  800
GQ339475.1|PUUV|N|Sweden|M.g.|Kiviniemi/Mg6/05|    : .CA.G..C..A..G...........G..A.....G.....A..G........C...............A.A.....G.T.......T......A...T.. :  800
GQ339476.1|PUUV|N|Sweden|M.g.|Aijajarvi/Mg7/05|    : .CA.G..C..A..G...........G........G.....A..G........C...............A.A.....G.T.......T.G....A...T.. :  800
```

```
                                                         *        720         *        740         *        760         *        780         *        800
2014.00598|PUUV|N|France|H.s.|59|FOURMIES|          : ATCGAATTAGGGAATTCATGGAAAAAGAGTGCCCTTTTATTAAACCAGAGGTGAAGCCAGGAACACCAGCCCAGGAAGCTGAATTCCTAAAGAGAAACAA :  800
GQ339477.1|PUUV|N|Sweden|M.g.|Aijajarvi/Mg9/05|     : .CA.G..C..A..G...........G........G.....A..G........C...............A.A.....G.T.......T.G....A...T.. :  800
GQ339478.1|PUUV|N|Sweden|M.g.|Jockfall/Mg12/05|     : .CA.G..C..A..............G........A.....A..G........T...............A.A.....G.T.......T.G..A.AG..T.. :  800
GQ339482.1|PUUV|N|Sweden|M.g.|Kalvudden/Mg22/05|    : ..A.G..C..A..G..T..............T..G.....A...........T...............A.A.....G.T.......T.G....AG..T.. :  800
GQ339479.1|PUUV|N|Sweden|M.g.|Moskosel/Mg17/05|     : .CA.G..C..A..G..T........G........G..C..A...........T.....G.........A.A......AT.......T.G....AG..T.. :  800
GQ339481.1|PUUV|N|Sweden|M.g.|Ljustrask/Mg20/05|    : .CA.G..C..A..G...........G........A..C..A...........T..............GA.A......AT.......T.G....AG..T.. :  800
GQ339480.1|PUUV|N|Sweden|M.g.|Gyttjea/Mg19/05|      : .CA.G..C..A..G..T........G........A..C..A...........T..A...........GA.A......AT......T..G....AG..T.. :  800
AY526219.1|PUUV|N|Sweden|Human|Umea/hu|             : .CA.G..A..A..G..T........G..A.....A.....A..G.....A..T..A.....G......A.A......AT...G...T......A...T.. :  800
AJ223380.1|PUUV|N|Sweden|M.g.|Tavelsjo/Cg81/94|     : .CA.G..AC.A..G..T........G..A..T..A.....A..G........T..A.....G......A.A..A..GAT.......T......A...T.. :  800
U14137.1|PUUV|N|Bosnia-H.|M.g.|Vranica|             : .CA.G..AC.A..G...........G..A..T..A.....A..G........T..A.....G......A.A..A..GAT.......T......A...T.. :  800
AJ223371.1|PUUV|N|Sweden|M.g.|Huggberget/Cg36/94|   : .CA.G..AC.A..G...........G..A.....A.....A..G........T..A.....G......A.A..A..GAT......TT.G....A...T.. :  800
Z48586.1|PUUV|N|Sweden|M.g.|Vindeln/L20Cg/83|       : .CA.G..AC.A..G..T........G..A.....A.....A..G.....A..T..A.....G......A.A.....GAT...G...T......A...T.. :  800
AJ223374.1|PUUV|N|Sweden|M.g.|Mellansel/Cg47/94|    : ..A.G..A..A.....T.....G..G..A.....A.....A..G.....A..T..A.....G......A.A......AT...G...T.G....A...T.. :  800
AJ223375.1|PUUV|N|Sweden|M.g.|Mellansel/Cg49/94|    : ..A.G..A..A.....T.....G..G..A.....A.....A..G.....A..T..A.....G......A.A......AT...G...T.G....A...T.. :  800
AJ238791.1|PUUV|N|Denmark|M.g.|Fyn/19|              : .GA.G..A.....G..T........G..A.....C.....A..G......C.T..A.....G...AAT..A..A........G...T....A.AG..T.G :  800
AJ278092.1|PUUV|N|Denmark|M.g.|Fyn47|               : .GA.G..A..A.....T........G..A.....C..CG.A..G......C.T..A..T..G...AAT..A..A........G...T....A.AG..T.G :  800
AJ278093.1|PUUV|N|Denmark|M.g.|Fyn131|              : ..A.G..A..A.....T........G..A.....C..CG.A..G......C.T..A..T..G...AAT..A..A........G...T....A.AG..T.G :  800
AJ223368.1|PUUV|N|Norway|M.g.|Eidsvoll/1124v|       : ..A.G..C..A..G........G...........C.....G........A..C.GA...........T..A.....G.T...G..TT..........T.. :  800
AJ223369.1|PUUV|N|Norway|M.g.|Eidsvoll/Cg1138/87|   : ..A.G..C..A..G........G...........C.....G........A..C.GA...........T..A.....G.T...G..TT..........T.. :  800
JN657228.1|PUUV|N|Latvia|M.g.|Jelgava/Mg149/2008|   : .A..G.....A..G........G.....A.....C.....C........A..T..A.....G........A..A.....T..G..TT....A.AG..T.. :  800
KX757839.1|PUUV|N|Lithuania|M.g.|LT15/164|2015|     : .AA....C..A.....T.....G.....A..T.....C..C....................G........A..A..G.TA..G...T....A.AG..T.. :  800
KX757840.1|PUUV|N|Lithuania|M.g.|LT15/174|2015|     : .AA....C..A.....T...........A..T.....C..C...........T........G........A..A..G.TA..G...T....A.AG..T.. :  800
KX815394.1|PUUV|N|Poland|M.g.|KS13/855|2009|        : .AA....C..A.....T.....G.....A..T.....C..C........A..C........G........A..A..G.T...G..TT.G..A.AG..T.. :  800
KX757841.1|PUUV|N|Lithuania|M.g.|LT15/201|2015|     : .AA....C..A.....T.....G.....A..T.....C..C....................G........A..A..G.TA..G...T....A.AG..T.. :  800
AJ314598.1|PUUV|N|Russia|M.g.|Baltic/49Cg/00|       : .AA.G..C.AA..G..T.....G..G..A...........A........A..T..A..............A.....G.TG.....T..G..A..G..T.. :  800
AJ314599.1|PUUV|N|Russia|M.g.|Baltic/205Cg/00|      : .AA....C.AA..G..T.....G..G..A........C..A........A..C..A............A.A.....G.TA.....T..G..A..G..T.. :  800
JN657229.1|PUUV|N|Latvia|M.g.|Madona/Mg99/2008|     : ..A....C.AA.....T.....G..G..A........C..A........T..C..A..............A.....GATA.....T........G..T.. :  800
JN657232.1|PUUV|N|Latvia|M.g.|Madona/Mg233/2008|    : ..A....C.AA.....T.....G..G..A........C..A........T..C..A..............A.....GATA.....T........G..T.. :  800
JN657230.1|PUUV|N|Latvia|M.g.|Jelgava/Mg136/2008|   : ..A....C.AA..G........G..G..AA..........A........T..T..A..............A.....G.TA.....T........G..T.. :  800
JN657231.1|PUUV|N|Latvia|M.g.|Jelgava/Mg140/2008|   : ..A....C.AA..G........G..G..A...........A........T..T..A..............A.....G.TA.....T........G..T.. :  800
Z30707.1|PUUV|N|Russia|M.g.|Udmurtia/458Cg/88|      : .GA.G........T..T........G..A.....C..C..A..G.....A..T..A..C..G........A.....GATA.....TT.G..A.......G :  800
Z30706.1|PUUV|N|Russia|M.g.|Udmurtia/444Cg/88|      : .GA.G........T..T........G..A.....C..C..A..G.....A..T..A..C..G........A.....GATA.....TT....A.......G :  800
Z84204.1|PUUV|N|Russia|M.g.|Kazan|                  : .GA.G........T..T........G..A.C...C..C..A..G.....A..T..A..C..G........A.....GATA.....TT.G..A.......G :  800
Z30708.1|PUUV|N|Russia|M.g.|Udmurtia/338Cg/92|      : .GA.G........T..T........G..A.....C..C..A..G.....A..T..A..C..G........A.....GATA.....TT.G..A.....T.G :  800
Z21497.1|PUUV|N|Russia|M.g.|Udmurtia/894Cg/91|      : .GA.G........T..T........G..A.....C..C..A..G.....A..T..A..C..G........A.....GATA.....TT.G..A.......G :  800
AB433843.2|PUUV|N|Russia|M.g.|Samara_49/CG/2005|    : .AA..........G..T.......GG..A........C..A..G.....A..T.....T..G........A.....G.TA.....TT.G..A.....T.G :  800
AB433845.2|PUUV|N|Russia|M.g.|Samara_94/CG/2005|    : .AA..........G..T.......GG..A.....C..C..A..G.....A..T.....T..G........A.....G.TA.....TT.G..A.....T.G :  800
L11347.1|PUUV|N|Russia|Human|P360|                  : .AAA.........G..T.....G.....A........C..A..G.....A..T..A..T..G........A.....G.TA.....TT.G..A.....T.G :  800
AB297665.2|PUUV|N|Russia|M.g.|DTK/Ufa-97|1997|      : .AAA.........G..T.....G.....A........C..A..G.....A..T..A..T..G........A.....G.TA.....TT.G..A.....T.G :  800
M32750.1|PUUV|N|Russia|M.g.|CG1820|                 : .AAA.........G..T.....G.....A........C..A..G.....A..T..A..T..G........A.....G.TA.....TT.G..A.....T.G :  800
AF442613.1|PUUV|N|Russia|M.g.|CG17/Baskiria-2001|   : .AAA.........G..T.....G.....A........C..A..G.....A..T..A..T..G........A.....G.TA.....TT.G..A.....T.G :  800
KX815395.1|PUUV|N|Poland|M.g.|KS14/118|2009|        : .AA......AA..G........G..G........C.....A........A..C..A..............A.....G.TA......T....A..G..T.. :  800
GQ339483.1|PUUV|N|Sweden|M.g.|Bergsjobo/Mg25/05|    : .AA.G.....A.....T...........A..T........A.....G..A.....A..T...........A.....G.T..............A...T.. :  800
AJ223377.1|PUUV|N|Sweden|M.g.|Solleftea/Cg6/95|     : .AA.G.....A.....T...........A..T........A.....G..A.....A..T...........A.....G.T..............A...T.. :  800
GQ339484.1|PUUV|N|Sweden|M.g.|Faboviken/Mg26/05|    : .AA.G.....A.....T.......................A........A..A.....T...........A..A..G.T.......T......A...... :  800
GQ339485.1|PUUV|N|Sweden|M.g.|Mangelbo/Mg1/05|      : .AA.G...........T.....T..G..A........C..A........A..A..A..T...........G......AT.......T.G....A...... :  800
GQ339486.1|PUUV|N|Sweden|M.g.|Munga/Mg2/05|         : .AA.G...........T.....G..G..A........C..A........A..A..A..T...........G......AT.......T......A...T.. :  800
GQ339487.1|PUUV|N|Sweden|M.g.|Munga/Mg16/05|        : .AA.G...........T.....G..G..A........C..A........A..A..A..T...........G......AT.......T......A...T.. :  800
AJ223376.1|PUUV|N|Sweden|M.g.|Solleftea/Cg3/95|     : .AA.G.....A.....T.......................A........A........T..........TA.....GAT.......T......A...T.. :  800
AF367071.1|PUUV|N|Russia|M.r.|CRF366|               : .AA.G.....A..G..T........G..A.....C.....A........A..T..A.....C........A.....GAT...GA.G.....A.AG..T.. :  800
AF367064.1|PUUV|N|Russia|M.g.|CG144|                : .AA.G.....A..G..T........G..A.....C.....A........A..T..A.....C........A.....GAT...GA.G.....A.AG..T.. :  800
AF367065.1|PUUV|N|Russia|M.g.|CG168|                : .AA.G.....A..G..T........G..A.....C.....A........A..T..A.....C.T......A.....GAT...GA.G.....A.AG..T.. :  800
AF367068.1|PUUV|N|Russia|M.g.|CG315|                : .AA.G.....A..G..T........G..A.....C.....G........A..T..A.....C........A.....GAT...GA.G.....A.AG..T.. :  800
AF367069.1|PUUV|N|Russia|M.r.|CRF161|               : .AA.G.....A..G..T........G..A.....C.....A........A..T..A.....C........A.....GAT...GA.G.....A.AG..T.. :  800
```

```
                                                                    *       720         *       740         *       760         *       780         *       800
2014.00598|PUUV|N|France|H.s.|59|FOURMIES|         : ATCGAATTAGGGAATTCATGGAAAAAGAGTGCCCTTTTATTAAACCAGAGGTGAAGCCAGGAACACCAGCCCAGGAAGCTGAATTCCTAAAGAGAAACAA :  800
AF367070.1|PUUV|N|Russia|M.r.|CRF308|              : .AA.G.....A..G..T........G..A.....C.....A........A..T..A.....C........A.....GAT...GA.G.....A.AG..T.. :  800
AF367066.1|PUUV|N|Russia|M.g.|CG215|               : .GA.G.....A..G..T........G..A.....C.....A........A..T..A.....C........A.....GAT...GA.G.....A.AG..T.. :  800
AF367067.1|PUUV|N|Russia|M.g.|CG222|               : .GA.G.....A..G..T........G..A.....C.....A........A..T..A.....C........A.....GAT...GA.G.....A.AG..T.. :  800
Z46942.1|PUUV|N|Finland|M.g.|Puumala/1324Cg/79|    : .GA.......A..G........G..G..A.....A..C..C..G.....A..C..A.....C........A.GA..GAT...GA.GT.G..A.....T.. :  800
Z30702.1|PUUV|N|Finland|M.g.|Evo/12Cg/93|          : .AA.G..CC.A..G..............A.....A..C..A..G.....AA.T........T........A.....GATG..GA.GT.G..A..G..T.. :  800
Z30703.1|PUUV|N|Finland|M.g.|Evo/13Cg/93|          : .AA.G..CC.A..G..............A.....A..C..A..G.....AA.T........T........A.....GATG..GA.GT.G..A..G..T.. :  800
Z30704.1|PUUV|N|Finland|M.g.|Evo/14Cg/93|          : .AA.G..CC.A..G..............A.....A..C..A..G.....AA.T........T........A.....GATG..GA.GT.G..A..G..T.. :  800
Z30705.1|PUUV|N|Finland|M.g.|Evo/15Cg/93|          : .AA.G..CC.A..G..............A.....A..C..A..G.....AA.T........T........A.....GATG..GA.GT.G..A..G..T.. :  800
Z69985.1|PUUV|N|Finland|M.g.|Virrat/25Cg/95|       : .GA....C..A..G....................A.....A..G.....AA.T..A.....T........A..A..GAT...GA.GT....A.....T.. :  800
JN831947.1|PUUV|N|Finland|Pieksamaki/human_lung/20 : .AA.......A..G...........G..A.....G..C..C..G..T..A..T..A.....C........A..A..GAT...GA.GT.G..A.....T.. :  800
JN831943.1|PUUV|N|Finland|Pieksamaki/Mg7/2008|     : .AA.......A..G...........G..A.....G..C..C..G..T..A..T..A.....C........A..A..GAT...GA.GT.G..A.....T.. :  800
JN831950.1|PUUV|N|Finland|Pieksamaki/human_kidney/ : .AA.......A..G...........G..A.....G..C..C..G..T..A..T..A.....C........A..A..GAT...GA.GT.G..A.....T.. :  800
JQ319166.1|PUUV|N|Finland|Konnevesi/Mg_O14B/2005|  : .GA....C..A..G...........G..A.....G..C.....G.....A..T..A.....C.....G..A..A..GAT...GA.GT.G..A.....T.. :  800
JQ319169.1|PUUV|N|Finland|Konnevesi/Mg_O27B/2005|  : .GA....C..A..G...........G..A.....G..C.....G.....A..T..A.....C.....G..A..A..GAT...GA.GT.G..A.....T.. :  800
JQ319167.1|PUUV|N|Finland|Konnevesi/Mg_O15B/2005|  : .GA....C..A..G...........G..A..T..G..C.....G.....A..T..A.....C.....G..A..A..GAT...GA.GT.G..A.....T.. :  800
JQ319164.1|PUUV|N|Finland|Konnevesi/Mg_O6B/2005|   : .GA....C..A..G.......G...G..A.....G..C.....G.....A..T..A.....C.....G..A..A..GAT...GA.GT.G..A.....T.. :  800
JQ319165.1|PUUV|N|Finland|Konnevesi/Mg_O9B/2005|   : .GA....C..A..G.......G...G..A.....G..C.....G.....A..T..A.....C.....G..A..A..GAT...GA.GT.G..A.....T.. :  800
JQ319170.1|PUUV|N|Finland|Konnevesi/Mg_O74B/2005|  : .GA....C..A..G...........G..A.....G..C.....G.....A..T..A.....C.....G..A..A..GAT...GA.GT.G..A.....T.. :  800
JQ319171.1|PUUV|N|Finland|Konnevesi/Mg_M114B/2005| : .GA....C..A..G...........G..A..T..G..C.....G.....A..T..A.....C.....G..A..A..GAT...GA.GT.G..A.....T.. :  800
AJ238788.1|PUUV|N|Russia|M.g.|Karhumaki|           : .GA....CC.A..G........G..G........A.....A........A..T........T........A.....GAT...GA.GT...GA.....T.. :  800
AJ238789.1|PUUV|N|Russia|M.g.|Kolodozero|          : .GA.G..C..A..G...........G........A.....A..G.....A..T........T...T....A..A..GAT...GA.GT.G..A..G..T.. :  800
AJ314597.1|PUUV|N|Finland|M.g.|Pallasjarvi/63Cg/98 : .GA....C..A..G....................A..C..A..G..G.....T........T..C.....A..A..G.T...GA.GT.G........T.. :  800
NC_005224.1|PUUV|N|Finland|M.g.|Sotkamo-revu-NCBI2 : .GA....C..A..G....................A..C..A..G..T..A..A..A.....C........A.....GAT...GA.GT....A.....T.. :  800
AJ238790.1|PUUV|N|Russia|M.g.|Gomselga|            : .GA....C.CA..G...........G........A..C..A........A..T........T.....G..A..A..GAT...GA.GT.T..A.....T.. :  800
AB010731.1|Hokkaido-V|N|Japan|M.r.|Tobetsu-60Cr-93 : .G.......AA...........G..G........A..C..A....................G..C.....AA.A..G.TG..GA.TT....A.....T.. :  800
AB010730.1|Hokkaido-V|N|Japan|M.r.|Kamiiso-8Cr-95| : .G.......AA.....T.....G..G........A.....A..G..G.................C.....AA.A..G.TG..GA.TT....A........ :  800
JX046487.1|Muju-V|N|South-Korea|M.r.|11-5|2011     : .CA......AA..G........G.....A..T..G..C..A..G......CC...A..G..T.....T..TAGT....T...G..TT.....C....T.. :  800
JX046484.1|Muju-V|N|South-Korea|M.r.|11-4|2011|    : .CA......AA..G........G.....A..T..G..C..A..G......CC...A..G..T.....T..TAGT....T...G..TT.....C....T.. :  800
JX028273.1|Muju-V|N|South-Korea|M.r.|11-1|2011|    : .CA......AA..G........G.....A..T..G..C..A..G......CCA..A..G..T.....T..TAGT...AT...G..TT.....C....T.. :  800
DQ138128.1|Muju-V|N|South-Korea|E.r.|00-18|        : .AA....C.AA..G........G.....A.....G........G.....ACCT..A.....C........TAGT....T...G...T.G..AC....... :  800
DQ138140.1|Muju-V|N|South-Korea|E.r.|99-27|        : .AA....C.AA..G........G.....A.....A........G.....ACCT..A.....C........TAGT....T...G...T.G..AC....... :  800
DQ138133.1|Muju-V|N|South-Korea|E.r.|96-1|         : .AAA...C.AA..G........G.....A.....A........G.....ACCT..A.....C........TAGT...AT...G...T.G..A........ :  800
DQ138142.1|Muju-V|N|South-Korea|E.r.|99-28|        : .AA....C.AA..G........G.....A.....A........G.....ACCT..A.....C........TAGT....T...G...T.G..AC....... :  800
```

```
                                                             *       820         *       840         *       860         *       880         *       900
2014.00598|PUUV|N|France|H.s.|59|FOURMIES|          : AATATACTTTATGCAGAGGCAGGAAGTGCTGGATAAAAACCATGTTGCTGATATTGATAAGTTGATTGACTATGCAGCCTCAGGAGATCCAACATCACCA :  900
2014.00233|PUUV|N|France|H.s.|59|FOURMIES|          : ..............................................................................T..................... :  900
2014.00097|PUUV|N|France|H.s.|02|SAINT-MICHEL|      : .................................................................................................... :  900
2014.00488|PUUV|N|France|H.s.|08|SIGNY-LE-PETIT|    : .................................................................................................... :  900
2014.00613|PUUV|N|France|H.s.|59|FOURMIES|          : ...............................................................A.................................... :  900
2015.00402|PUUV|N|France|H.s.|08|CHARLEVILLE-MEZIE  : ....................................G............................................................... :  900
2015.00422|PUUV|N|France|H.s.|59|ANOR|              : .................................................................................................... :  900
2015.00488|PUUV|N|France|H.s.|08|SEDAN|             : ....................................G................................T.............................. :  900
2015.00498|PUUV|N|France|H.s.|08|REMILLY-AILLICOUR  : .................................................................................................... :  900
2016.00295|PUUV|N|France|H.s.|08|CORNY-MACHEROMENI  : .................................................................................................... :  900
2016.00333|PUUV|N|France|H.s.|59|FOURMIES|          : .................................................................................................... :  900
2016.00345|PUUV|N|France|H.s.|59|FOURMIES|          : .................................................................................................... :  900
2016.00357|PUUV|N|France|H.s.|02|HIRSON|            : .................................................................................................... :  900
2016.00427|PUUV|N|France|H.s.|59|FOURMIES|          : .................................................................................................... :  900
2016.00469|PUUV|N|France|H.s.|02|PREMONTRE|         : .....................A.....A...........T.............................T.............................. :  900
AJ277075.1|PUUV|N|Belgium|M.g.|Montbliart/CG14444|  : ...........................A.............................C.....A.................................... :  900
2014.00053|PUUV|N|France|H.s.|08|SECHEVAL|          : ....................................G................................T.............................. :  900
KT247593.1|PUUV|N|France|M.g.|08|Ardennes/Mg75/201  : ........................C..A........................................................................ :  900
KT247592.1|PUUV|N|France|M.g.|08|Ardennes/Mg156/20  : ........................C..A........................................................................ :  900
2015.00019|PUUV|N|France|H.s.|08|ETEIGNIERES|       : ...............A........T......................T........................C........................... :  900
2016.00239|PUUV|N|France|H.s.|08|VIREUX-MOLHAIN|    : ........................T..A...........T................................C........................... :  900
2015.00329|PUUV|N|France|H.s.|08|BOGNY-SUR-MEUSE|   : ........................C..A........................................................................ :  900
2014.00209|PUUV|N|France|H.s.|08|TREMBLOIS-LES-ROC  : ........................C..A..........................C.................C........................... :  900
2015.00456|PUUV|N|France|H.s.|94|ALFORTVILLE|       : ........................C..A........................................................................ :  900
2012.00018|PUUV|N|France|H.s.|08|ROCROI|            : ........................C..A..........................C............................................. :  900
AJ277032.1|PUUV|N|Belgium|M.g.|Momignies/47Cg/96|   : ..................A.....C..A...........T................................C........................... :  900
AJ277034.1|PUUV|N|Belgium|M.g.|Couvin/59Cg/97|      : ........................C..A........................................................................ :  900
AJ277033.1|PUUV|N|Belgium|M.g.|Momignies/55Cg/96|   : ..................A.....C..A...........T................................C........................... :  900
2012.00638|PUUV|N|France|H.s.|08|GIVET|             : ........................T..A.................................C.A.................................... :  900
2015.00328|PUUV|N|France|H.s.|08|VRIGNE-MEUSE|      : ...........................AT..................................................T..................... :  900
2015.00419|PUUV|N|France|H.s.|08|DOUZY|             : G.................A........A........................................................................ :  900
2016.00310|PUUV|N|France|H.s.|08|THIN-LE-MOUTIER|   : .................................................................................................... :  900
AJ277030.1|PUUV|N|Belgium|M.g.|Thuin/33Cg/96|       : .........C.................A........................................................................ :  900
AJ238779.1|PUUV|N|Germany|M.g.|NRW/Cg-Erft|         : .....................A.....AT............................C...C.......T.............................. :  900
2014.00276|PUUV|N|France|H.s.|59|LILLE|             : ...........................A........................................................................ :  900
U22423.1|PUUV|N|Belgium|M.g.|CG13891|               : ......T........A..A..A.....A........G..T...........A.................T........T..................... :  900
2014.00321|PUUV|N|France|H.s.|59|FOURMIES|          : .................................................................................................... :  900
2015.00457|PUUV|N|France|H.s.|55|REVIGNY-SUR-ORNAI  : .....................A.....A...........T.....G.......................T.............................. :  900
2013.00250|PUUV|N|France|H.s.|62|VENDIN-LS-BTHUNE|  : .....................A.....AT.........................................T........T..................... :  900
2016.00282|PUUV|N|France|H.s.|59|MORBECQUE|         : .....................A..G..............................................T........T........C............ :  900
AJ277076.1|PUUV|N|Belgium|M.g.|Montbliart/CG14445|  : ...........................A.............................C.....A.................................... :  900
2014.00153|PUUV|N|France|H.s.|02|BUIRONFOSSE|       : ...........................A........................................................................ :  900
2014.00171|PUUV|N|France|H.s.|59|SAINT-SAULVE|      : .....................A.....A..A.......................................T.............................. :  900
2016.00293|PUUV|N|France|H.s.|60|LA-NEUVILLE-SUR-R  : G....................A.....AT.................................C.......T.................C............ :  900
2014.00499|PUUV|N|France|H.s.|94|CHAMPIGNY-SUR-MAR  : .....................A.....A..............................C........C................................. :  900
2012.00057|PUUV|N|France|H.s.|59|COUSOLRE|          : ......T....................A........................................................................ :  900
2012.00349|PUUV|N|France|H.s.|02|ENGLANCOURT|       : ...........................A.......................................C................................ :  900
2014.00135|PUUV|N|France|H.s.|02|ATHIES-SOUS-LAON|  : .....................A.....A..............................C........C................................. :  900
2014.00184|PUUV|N|France|H.s.|59|FOURMIES|          : ...........................A..............................C........C................................. :  900
2016.00182|PUUV|N|France|H.s.|08|BOULZICOURT|       : ...........................A.............................C.....A.................................... :  900
2016.00286|PUUV|N|France|H.s.|02|TRUCY|             : .....................A.....A..............................C........C................................. :  900
2016.00325|PUUV|N|France|H.s.|59|AULNOYE-AYMERIES|  : ...........................A........................................................................ :  900
2016.00452|PUUV|N|France|H.s.|02|LAON|              : .....................A.....A..............................C........C................................. :  900
2016.00467|PUUV|N|France|H.s.|02|AISNE|             : ...........................A.......................................C..............G.................. :  900
```

```
                                                              *       820         *       840         *       860         *       880         *       900
2014.00598|PUUV|N|France|H.s.|59|FOURMIES|           : AATATACTTTATGCAGAGGCAGGAAGTGCTGGATAAAAACCATGTTGCTGATATTGATAAGTTGATTGACTATGCAGCCTCAGGAGATCCAACATCACCA : 900
2015.00657|PUUV|N|France|H.s.|02|CILLY|              : .....................A.....A.........G.............................C..T..............G..C...........G : 900
2015.00526|PUUV|N|France|H.s.|08|MONTHERME|          : ...........................A........................................................................ : 900
2015.00045|PUUV|N|France|H.s.|59|FOURMIES|           : ...............A...........A...................T.................................................... : 900
2015.00430|PUUV|N|France|H.s.|02|MONTCORNET|         : ...........................A......................................C................................. : 900
2016.00311|PUUV|N|France|H.s.|02|LAON|               : ..........C..........A.....A.............................C........C................................. : 900
AJ277031.1|PUUV|N|Belgium|M.g.|Montbliart/23Cg/96|   : ...........................A.............................C.......................................... : 900
2016.00326|PUUV|N|France|H.s.|02|LAON|               : .....................A.....A.............................C........C................................. : 900
2016.00353|PUUV|N|France|H.s.|02|CESSIERES|          : .....................A.....A.............................C........C................................. : 900
2012.00061|PUUV|N|France|H.s.|02|LANISCOURT|         : .....................A.....A.............................C........C................................. : 900
2014.00174|PUUV|N|France|H.s.|02|ST-ERME-OUTRE-ET-   : .....................A.....A.............................C........C................................. : 900
2016.00268|PUUV|N|France|H.s.|02|PRESLES-ET-THIERN   : .....................A.....A.............................C........C................................. : 900
2015.00660|PUUV|N|France|H.s.|02|VIC-SUR-AISNE|      : ...........................AT........................................T.............................. : 900
2012.00025|PUUV|N|France|H.s.|51|SAINTE-MENEHOULD|   : .G....T...........A..A..................T.....G.A.....................T..........................G... : 900
2015.00665|PUUV|N|France|H.s.|51|REIMS|              : .G.G..T...........A..A.....AT...........T.....G.A.....................T..........................G... : 900
2012.00307|PUUV|N|France|H.s.|54|COLOMBEY-LES-BELL   : .G...................A..G...........G..T.....G.................A.....T..............G..C...........G : 900
2012.00123|PUUV|N|France|H.s.|55|BAR-LE-DUC|         : .G....T..............A.....A............T.....G.A.............C.......T..........................G... : 900
2012.00278|PUUV|N|France|H.s.|51|REIMS|              : .G....T...........A..A.....A............T.....G.A..C..................T..........................G... : 900
2015.00185|PUUV|N|France|H.s.|68|FELLERING|          : .G....T...........A..A.....A............T.....A.TA....................T..........................G... : 900
KJ994776.1|PUUV|N|Germany|M.g.|Mu/07/1219|2007|      : .G.C..T.........C.A.....T..A..C..............G.....C........AC.......T........T........C..T......... : 900
2012.00086|PUUV|N|France|H.s|58|CHEVROCHES|          : GG.G..T..C.....AC.C..A..T.....T........T...........C...........A.....T.................C..C..C.....T : 900
KT247595.1|PUUV|N|France|M.g.|45|Orleans/Mg29/2010   : GG.G.....C.....AC.C..A..T..A..T........T.....A.....C...........A..C..T.........G..........C..T.....T : 900
KT247594.1|PUUV|N|France|M.g.|45|Orleans/Mg23/2010   : GG.G.....C.....AC.C..A..T..A..T........T.....A.....C...........A..C..T.........G..........C..T.....T : 900
KY365004.1|PUUV|N|France|M.g.|45|Orleans/NCHA373/2   : GG.G.....C.....AC.C..A..T.....T........T.....A.....C...........A..C..T.........G.......C..C..T.....T : 900
2012.00301|PUUV|N|France|H.s.|25|MOUTHE|             : G...............C.T..A..T..AT.......G..T.....A.....C.........C.......T....................T........C : 900
AM695638.1|PUUV|N|France|M.g.|Mignovillard/CgY02/2   : G...............C.T..A..T..AT.......G..T.....A.....C.........C.......T....................T........C : 900
KT247597.1|PUUV|N|France|M.g.|39|Jura/Mg214/2010|    : G...............C.T..A..T.....A........T.....A.....C.........C.......T....................T........T : 900
KT247596.2|PUUV|N|France|M.g.|39|Jura/Mg2/2010|      : G...............C.T..A..T..AT..........T.....A.....C.........C.......T....................T........C : 900
2012.00102|PUUV|N|France|H.s.|39|COISERETTE|         : G........C......C.T..A..T..AT..........T.....A.....C.........C.......T....................T........C : 900
2012.00536|PUUV|N|France|H.s.|39|LA-PESSE|           : G........C......C.T..A..T..AT..........T.....A.....C.........C.......T....................T........C : 900
2014.00622|PUUV|N|France|H.s.|39|ARBOIS|             : G...............C.T..A..T...T.......G..T.....A.....C.........C.......T....................T........T : 900
2015.00567|PUUV|N|France|H.s.|70|RIOZ|               : G...............C.T..A..T..AT..........T.....A.....C.........C.......T....................T........T : 900
2014.00637|PUUV|N|France|H.s.|25|SAULES|             : G...............C.T.....T..AT..........T.....A.....C.........C.......T....................T........T : 900
2012.00396|PUUV|N|France|H.s.|39|SAINT-CLAUDE|       : G...............C.T..A..T..AT.......G..T.....A.....C.........C.......T....................T........C : 900
2014.00120|PUUV|N|France|H.s.|38|LE-MOUTARET|        : G...............C.T..A..T..AT.......G..T.....A.....C.........C.......T....................T........T : 900
2015.00153|PUUV|N|France|H.s.|73|GREZY-SUR-ISERE|    : G...............C.T..A..T..AT.......G..T.....A.....C.........C.......T....................T........T : 900
2015.00504|PUUV|N|France|H.s.|70|RONCHAMP|           : G.....T.........C.C..A..T...T.......G..T.....A.....C.........C.A.....T....................T........G : 900
2016.00275|PUUV|N|France|H.s.|21|JALLANGES|          : G...............C.T.....T...T..........T.....A...............C.......T....................T........C : 900
2016.00320|PUUV|N|France|H.s.|25|SAINT-VIT|          : G...............C.T..A..T..AT..........T.....A.....C.........C.......T.............................T : 900
2015.00410|PUUV|N|France|H.s.|70|ANGIREY|            : G.....T.........C.C..A..T...T.......G..T.....A.....C.....C...C.A.....T....................T........G : 900
DQ016430.2|PUUV|N|Germany|M.g.|Bavaria/CG33/04|      : .G.T..T........AC.C..A..T..AT....C.....T.....G.................A.....T....................C........T : 900
DQ016432.2|PUUV|N|Germany|M.g.|Bavaria/CG41/04|      : .G.T...........AC.C..A..T..AT..........T.....G.................A.....T....................T........T : 900
AY954723.2|PUUV|N|Germany|M.g.|Bavaria-CG34/04|      : .G.T...........AC.C..A..T..AT....C.....T.....G.................A.....T....................T........T : 900
AY954722.2|PUUV|N|Germany|M.g.|Bavaria-CG9/04|       : .G.T...........AC.C..A..T...T....C.....T.....G.................A.....T....................C........T : 900
2012.00402|PUUV|N|France|H.s.|60|GOUVIEUX|           : ................C.C..A..CA.A........G........A.....C..................T........T........C..C......... : 900
2014.00540|PUUV|N|France|H.s.|60|CHAMBLY|            : .G....T.........C.C..A..CA.A........G........G.....C..................T........T........C..C......... : 900
AJ314600.1|PUUV|N|Balkan|M.g.|Balkan-1|              : GG.G.....C......C.T..A..T.....A........T.....G.A................C....T..............G..............AT : 900
AJ314601.1|PUUV|N|Balkan|M.g.|Balkan-2|              : .G.G.....C......C.T..A..T.....A........T.....G.A.....................T..C...........G..C............T : 900
FN377821.1|PUUV|N|Hungary|M.g.|Mg9/HungaryTR17/00|   : GG.G.....C......C.T..A..T.....A........T.....G.A.....................T..C...........G..C.........C..T : 900
FN377822.1|PUUV|N|Hungary|M.g.|Mg23/HungaryTR17/00   : GG.......C......C.T..A..T.....A........T.....G.G.....................T..C...........G..C............T : 900
GQ339473.1|PUUV|N|Sweden|M.g.|Kiviniemi/Mg3/05|      : .G.T.....CC.AG.CC.C..AA.T..T...........T.....A.....C..............A........T........................ : 900
GQ339474.1|PUUV|N|Sweden|M.g.|Kiviniemi/Mg5/05|      : .G.T.....CC.AG.CC.C..AA.T..T...........T.....A....................A........T........................ : 900
GQ339475.1|PUUV|N|Sweden|M.g.|Kiviniemi/Mg6/05|      : .G.T.....CC.AG.CC.C..AA.T..T...........T.....A....................A........T........................ : 900
GQ339476.1|PUUV|N|Sweden|M.g.|Aijajarvi/Mg7/05|      : .G.T.....CC.AG.CC.C..AA.T..T...........T.....A.....C..............A........T........................ : 900
```

```
                                                   *       820         *       840         *       860         *       880         *       900
2014.00598|PUUV|N|France|H.s.|59|FOURMIES|      : AATATACTTTATGCAGAGGCAGGAAGTGCTGGATAAAAACCATGTTGCTGATATTGATAAGTTGATTGACTATGCAGCCTCAGGAGATCCAACATCACCA :  900
GQ339477.1|PUUV|N|Sweden|M.g.|Aijajarvi/Mg9/05|   : .G.T.....CC.AG.CC.C..AA.T..T...........T.....A.....C..............A........T........................ :  900
GQ339478.1|PUUV|N|Sweden|M.g.|Jockfall/Mg12/05|   : .G.C..T..CC.AG.CC.C..AA.T..T..A..C..G..T.....G.....C..............A........T..........C............ :  900
GQ339482.1|PUUV|N|Sweden|M.g.|Kalvudden/Mg22/05|  : .G.C..T..CC.AG.CC.C..AA.T..T..A........T.....A.....C..............A........T........................ :  900
GQ339479.1|PUUV|N|Sweden|M.g.|Moskosel/Mg17/05|   : .G.C..T..CT..G.CC.T..AA.T..T..A..C.....T.....G..C..C...........A...........T........G..............T :  900
GQ339481.1|PUUV|N|Sweden|M.g.|Ljustrask/Mg20/05|  : .G.C..T..CT..G.CC.T..AA.T..T..A..C.....T.....G..C..............A...........T........G..............T :  900
GQ339480.1|PUUV|N|Sweden|M.g.|Gyttjea/Mg19/05|    : .G.C..T..CT..G.CC.T..AACT..T..T..C.....T.....G..C..C...........A...........T........G..............C :  900
AY526219.1|PUUV|N|Sweden|Human|Umea/hu|           : .G.T.........G.CC.C..AA.T..C..A.....G..T.....G.....C........A..A...........T........G..C............ :  900
AJ223380.1|PUUV|N|Sweden|M.g.|Tavelsjo/Cg81/94|   : .G.T.........G.CC.C..AATT..T..A........T.....G..............A..A...........T........G............... :  900
U14137.1|PUUV|N|Bosnia-H.|M.g.|Vranica|           : .G.T.........G.CC.C..AA.T..T..A........T.....G..............A..A.....T.....T........G............... :  900
AJ223371.1|PUUV|N|Sweden|M.g.|Huggberget/Cg36/94| : .G.C.........G.CC.C...A.T..T..A........T.....G..A..C........A..A...........T........G............... :  900
Z48586.1|PUUV|N|Sweden|M.g.|Vindeln/L20Cg/83|     : .G.T.........G.CC.C..AA.T..T..A........T.....G.....C........A..A..C........T........G............... :  900
AJ223374.1|PUUV|N|Sweden|M.g.|Mellansel/Cg47/94|  : .G.T.........G.CC.C..AA.T..CT.A.....G..T.....G.....C...........A...........T..........C............ :  900
AJ223375.1|PUUV|N|Sweden|M.g.|Mellansel/Cg49/94|  : .G.T.........G.CC.C..AA.T..CT.A.....G..T.....G.....C...........A...........T..........C............ :  900
AJ238791.1|PUUV|N|Denmark|M.g.|Fyn/19|            : GG.T......C..A.TC.C.....T..A..T.....G..T...........C.........C.A..A...........A.....T..C..T.....C..T :  900
AJ278092.1|PUUV|N|Denmark|M.g.|Fyn47|             : GG.T.........A.TC.C.....T.....T.....G..T.....A.....C........AC....A...........A.....T.....T.....C..T :  900
AJ278093.1|PUUV|N|Denmark|M.g.|Fyn131|            : GG.T.........A.TC.C.....T.....T.....G..T.....A.....C........AC....A...........A.....T.....T....GC..T :  900
AJ223368.1|PUUV|N|Norway|M.g.|Eidsvoll/1124v|     : GG....T..C.....AC.C.....T..C..T..C.....T.....G..............AC.T.....T.................C............ :  900
AJ223369.1|PUUV|N|Norway|M.g.|Eidsvoll/Cg1138/87| : GG....T..C.....AC.C.....T..C..T..C.....T.....G..............AC.T.....T.................C............ :  900
JN657228.1|PUUV|N|Latvia|M.g.|Jelgava/Mg149/2008| : GG.T.....C.....AC.C.....T.....T..C..G..T.....G.....C..C..............T........T.....................T :  900
KX757839.1|PUUV|N|Lithuania|M.g.|LT15/164|2015|   : .G.T..T........AC.C.....T.....T.....G........G..............A.......................G..C............ :  900
KX757840.1|PUUV|N|Lithuania|M.g.|LT15/174|2015|   : .G.T..T........AC.T.....T..A..T.....G........G..............A..A.....................C............ :  900
KX815394.1|PUUV|N|Poland|M.g.|KS13/855|2009|      : GG.T..T..C......C.C.....T.....T..C..G..T.....G...............C.A.....................C............ :  900
KX757841.1|PUUV|N|Lithuania|M.g.|LT15/201|2015|   : .G.T..T........AC.C.....T.....T.....G........G..............A.......................G..C............ :  900
AJ314598.1|PUUV|N|Russia|M.g.|Baltic/49Cg/00|     : .G.G.........A.CC.C.....T..C..T..C.....T...........C.........C.A...........T.....G........T........T :  900
AJ314599.1|PUUV|N|Russia|M.g.|Baltic/205Cg/00|    : .G.G.........A.CC.T.....T..C..T..C.....T...........C.........C.A...........T...............T........T :  900
JN657229.1|PUUV|N|Latvia|M.g.|Madona/Mg99/2008|   : .G...........A.CC.T.....T..C..T.....G........G.....C..C.....AC.A...........T.....T.....C...........T :  900
JN657232.1|PUUV|N|Latvia|M.g.|Madona/Mg233/2008|  : .G...........A.CC.T.....T..C..T.....G........G.....C..C.....AC.A...........T.....T.....C...........T :  900
JN657230.1|PUUV|N|Latvia|M.g.|Jelgava/Mg136/2008| : .G...........A.CC.T.....T..C..C.....G........G.....C..C.....AC.A.....T.....T.....T........T........T :  900
JN657231.1|PUUV|N|Latvia|M.g.|Jelgava/Mg140/2008| : .G...........A.CC.T.....T..C..C.....G........G.....C..C.....AC.A.....T.....T.....T........T........T :  900
Z30707.1|PUUV|N|Russia|M.g.|Udmurtia/458Cg/88|    : GG.T..T..C...ACTC.C..A..T..T..T........T.....G.....C...........A...........T.....T..T..C..T........T :  900
Z30706.1|PUUV|N|Russia|M.g.|Udmurtia/444Cg/88|    : GG.T..T..C...ACTC.C..A..T..T..T........T.....G.....C...........A.................T..T..C..T........T :  900
Z84204.1|PUUV|N|Russia|M.g.|Kazan|                : GG.T..T..C...ACTC.C..A..T..T..T........T.....G.....C...........A...........T.....T..T..C..T........T :  900
Z30708.1|PUUV|N|Russia|M.g.|Udmurtia/338Cg/92|    : GG.T..T..C...ACTC.C..A..T..T..T........T.....G.....C...........A.................T.....C..T........T :  900
Z21497.1|PUUV|N|Russia|M.g.|Udmurtia/894Cg/91|    : GG.T..T..C...ACTC.C..A..T..T..T........T.....G.....C...........A...........T.....T..T..C..T........T :  900
AB433843.2|PUUV|N|Russia|M.g.|Samara_49/CG/2005|  : .G.T..T..C...ACCC.C..A..T..T..T........T.....G.....C.....C.....A...........T.....T..T..C..T........T :  900
AB433845.2|PUUV|N|Russia|M.g.|Samara_94/CG/2005|  : .G.T..T..C...ACCC.C..A..T..T..T........T.....G.....C...........A...........T.....T..T..C..T........T :  900
L11347.1|PUUV|N|Russia|Human|P360|                : .G.T..T..C...ACCC.C.....T..T..T..C.....T.....G.....C..C....................T.....T..T..C..T.....G..T :  900
AB297665.2|PUUV|N|Russia|M.g.|DTK/Ufa-97|1997|    : .G.T..T..C...ACCC.C.....T..T..T..C.....T.....G.....C..C....................T.....T..T..C..T.....G..T :  900
M32750.1|PUUV|N|Russia|M.g.|CG1820|               : .G.T..T..C...ACCC.C.....T..T..T..C.....T.....G.....C..C....................T.....T..T..C..T.....G..T :  900
AF442613.1|PUUV|N|Russia|M.g.|CG17/Baskiria-2001| : .G.T..T..C...ACCC.C.....T..T..T........T.....G.....C..C....................T.....T..T..C..T........T :  900
KX815395.1|PUUV|N|Poland|M.g.|KS14/118|2009|      : .G.T..T..C...A.TC.C..A..T..C..T..C..G........G.....C........A..A...........C.....T.....C..T........T :  900
GQ339483.1|PUUV|N|Sweden|M.g.|Bergsjobo/Mg25/05|  : G..C..T.........C.C..A..T.....T..C.....T.....G.....C..C..............T.....T...........C..T......... :  900
AJ223377.1|PUUV|N|Sweden|M.g.|Solleftea/Cg6/95|   : G..C..T.........C.C..A..T.....T..C.....T.....G.....C..C..............T.................C..T......... :  900
GQ339484.1|PUUV|N|Sweden|M.g.|Faboviken/Mg26/05|  : G..C............C.C.....T.....T........T.....G.....C..C.....A........T.....T........G..C..T......... :  900
GQ339485.1|PUUV|N|Sweden|M.g.|Mangelbo/Mg1/05|    : G..C...........AC.T.....T..A..T..C.....T.....G.....C........A........T.....T...........C...........T :  900
GQ339486.1|PUUV|N|Sweden|M.g.|Munga/Mg2/05|       : G..C...........AC.T.....T.....T..C.....T.....G.....C........A........T.....T...........C...........T :  900
GQ339487.1|PUUV|N|Sweden|M.g.|Munga/Mg16/05|      : G..C...........AC.T.....T.....T..C.....T.....G.....C........A........T.....T...........C...........T :  900
AJ223376.1|PUUV|N|Sweden|M.g.|Solleftea/Cg3/95|   : G..C.....C......C.C.....T.....T........T.....G..A..C........A........T.....T...........C..T......... :  900
AF367071.1|PUUV|N|Russia|M.r.|CRF366|             : ...C..T........TC.A.....T.....T........T.....G..A..C.............................T........T..G.....T :  900
AF367064.1|PUUV|N|Russia|M.g.|CG144|              : ...C..T........TC.A.....T.....T........T.....G..A..C.............................T........T..G.....T :  900
AF367065.1|PUUV|N|Russia|M.g.|CG168|              : ...C..T........TC.A.....T.....T........T.....G..A..C..C..........................T........T..G.....T :  900
AF367068.1|PUUV|N|Russia|M.g.|CG315|              : ...C..T........TC.A.....T.....T........T.....G..A..C..C..........................T........T..G.....T :  900
AF367069.1|PUUV|N|Russia|M.r.|CRF161|             : ...C..T........TC.A.....T.....T........T.....G..A..C..C..........................T........T..G.....T :  900
```

```
                                                               *       820         *       840         *       860         *       880         *       900
2014.00598|PUUV|N|France|H.s.|59|FOURMIES|        : AATATACTTTATGCAGAGGCAGGAAGTGCTGGATAAAAACCATGTTGCTGATATTGATAAGTTGATTGACTATGCAGCCTCAGGAGATCCAACATCACCA :  900
AF367070.1|PUUV|N|Russia|M.r.|CRF308|             : ...C..T........TC.A.....T.....T........T.....G..A..C..G..........................T........T..G.....T :  900
AF367066.1|PUUV|N|Russia|M.g.|CG215|              : ...C..T........TC.A.....T.....T........T.....G..A..C..C..........................T........T..G.....T :  900
AF367067.1|PUUV|N|Russia|M.g.|CG222|              : ...C..T........TC.A.....T.....T........T.....G..A..C..C..........................T........T..G.....T :  900
Z46942.1|PUUV|N|Finland|M.g.|Puumala/1324Cg/79|   : G..C............C.T.....T.....T..C.....T.....G..A..C...........A.................T..T..C..T........T :  900
Z30702.1|PUUV|N|Finland|M.g.|Evo/12Cg/93|         : G..C.....C......C.C.....T.....T..C...........G..A..C..C......C.A.................T.....C..C........T :  900
Z30703.1|PUUV|N|Finland|M.g.|Evo/13Cg/93|         : G..C.....C......C.C.....T.....T..C...........G..A..C..C......C.A.................T.....C..C........T :  900
Z30704.1|PUUV|N|Finland|M.g.|Evo/14Cg/93|         : G..C.....C......C.C.....T.....T..C...........G..A..C..C......C.A.................T....GC..C........T :  900
Z30705.1|PUUV|N|Finland|M.g.|Evo/15Cg/93|         : G..C.....C......C.C.....T.....T..C...........G..A..C..C......C.A.................T.....C..C........T :  900
Z69985.1|PUUV|N|Finland|M.g.|Virrat/25Cg/95|      : ...C..T..C......C.C..A..T..A..T..C..G..T.....G..A..C........AC.A.................T........C........T :  900
JN831947.1|PUUV|N|Finland|Pieksamaki/human_lung/20 : G..T............C.C.....T..A..T........T.....G..A..............A.................T..T..C..C..G.....T :  900
JN831943.1|PUUV|N|Finland|Pieksamaki/Mg7/2008|    : G..T............C.C.....T..A..T........T.....G..A..............A.................T..T..C..C..G.....T :  900
JN831950.1|PUUV|N|Finland|Pieksamaki/human_kidney/ : G..T............C.C.....T..A..T........T.....G..A..............A.................T..T..C..C..G.....T :  900
JQ319166.1|PUUV|N|Finland|Konnevesi/Mg_O14B/2005| : ...T............C.C.....T..A..T..C.....T.....G..A..C.............................C..T..C..T........T :  900
JQ319169.1|PUUV|N|Finland|Konnevesi/Mg_O27B/2005| : ...T............C.C.....T..A..T..C.....T.....G..A..C.............................C..T..C..T........T :  900
JQ319167.1|PUUV|N|Finland|Konnevesi/Mg_O15B/2005| : ...T............C.C.....T..A..T..C.....T.....G..A..C.............................C..T..C..T........T :  900
JQ319164.1|PUUV|N|Finland|Konnevesi/Mg_O6B/2005|  : ...T............C.C.....T..A..T..C.....T.....G..A..C.............................C..T..C..T........T :  900
JQ319165.1|PUUV|N|Finland|Konnevesi/Mg_O9B/2005|  : ...T............C.C.....T..A..T..C.....T.....G..A..C.............................C..T..C..T........T :  900
JQ319170.1|PUUV|N|Finland|Konnevesi/Mg_O74B/2005| : ...T............C.C.....T..A..T..C.....T.....G..A..C.............................G..T..C..T........T :  900
JQ319171.1|PUUV|N|Finland|Konnevesi/Mg_M114B/2005| : ...T............C.C.....T..A..T..C.....T.....G..A..C.............................C..T..C..T........T :  900
AJ238788.1|PUUV|N|Russia|M.g.|Karhumaki|          : G..C..T..C......C.C..A..T..A..T..C.....T.....G..A..C...........A.....TCTC.....T..T.....C..T........T :  900
AJ238789.1|PUUV|N|Russia|M.g.|Kolodozero|         : G..C..T..C......C.C..A..T.....T..C...........G..C..C.....C...C.A.................T.....C..T..G.....T :  900
AJ314597.1|PUUV|N|Finland|M.g.|Pallasjarvi/63Cg/98 : G..C..T..C......C.C.....T.....T..C.....T.....G..A..C...........A.................T.....C..C.....G... :  900
NC_005224.1|PUUV|N|Finland|M.g.|Sotkamo-revu-NCBI2 : G..C............C.C.....T.....T..C.....T.....G..A..C.....C.....A.................T.....C..T........T :  900
AJ238790.1|PUUV|N|Russia|M.g.|Gomselga|           : G..C..T..C......C.C.....T.....T..C.....T.....G..A..C..........CA.................T.A...C..T........T :  900
AB010731.1|Hokkaido-V|N|Japan|M.r.|Tobetsu-60Cr-93 : .G.T.....C.....AC....A..T.....T.....G..T.....CC.C..C..C.....AC.A.................T........T..G.....T :  900
AB010730.1|Hokkaido-V|N|Japan|M.r.|Kamiiso-8Cr-95| : .G.T.....C.....AC.......T.....T.....G..T.....CC.C..C..C.....AC.A.................T........C........T :  900
JX046487.1|Muju-V|N|South-Korea|M.r.|11-5|2011    : G..C..T..C...AGC..A..A..T..CT..........T......C....C.........C.............T...........C...........T :  900
JX046484.1|Muju-V|N|South-Korea|M.r.|11-4|2011|   : G..C..T..C...AGC..A..A..T..CT..........T......C....C.........C.............T...........C...........T :  900
JX028273.1|Muju-V|N|South-Korea|M.r.|11-1|2011|   : G..C..T..C...AGC..A..A..T..C...........T......C....C.........C.............T...........C...........T :  900
DQ138128.1|Muju-V|N|South-Korea|E.r.|00-18|       : G..C..T..C...A.T.....A..T..CT.A.....G..T......C....C........A........T.............................T :  900
DQ138140.1|Muju-V|N|South-Korea|E.r.|99-27|       : G..C..T..C...A.T.....A..T..CT.A.....G..T......C....C........AC.......T.............................T :  900
DQ138133.1|Muju-V|N|South-Korea|E.r.|96-1|        : G..C..T..C...A.T.....A..T..CT.A.....G..T.....AC.............AC.......T.............................T :  900
DQ138142.1|Muju-V|N|South-Korea|E.r.|99-28|       : G..C..T..C...A.T.....A..T..CT.A.....G..T......C....C........AC.......T.............................T :  900
```

```
                                                   ACCCAIAAACGIACACG  PPT986R  Bowen MD et al. J Med Virol 1997
                                                              *        920         *        940         *        960         *        980         *       1000
2014.00598|PUUV|N|France|H.s.|59|FOURMIES|         : GATAATATTGAAGCCCCGAATGCACCCTGGGTGTTTGCTTGTGCACCCGACCGGTGCCCACCGACATGTATTTATGTTGCAGGGATGGCTGAACTAGGTG : 1000
2014.00233|PUUV|N|France|H.s.|59|FOURMIES|         : .................A.................................................................................. : 1000
2014.00097|PUUV|N|France|H.s.|02|SAINT-MICHEL|     : .................................................................................................... : 1000
2014.00488|PUUV|N|France|H.s.|08|SIGNY-LE-PETIT|   : ..........................T................................G..A..................................... : 1000
2014.00613|PUUV|N|France|H.s.|59|FOURMIES|         : ...........................................................G..A..................................... : 1000
2015.00402|PUUV|N|France|H.s.|08|CHARLEVILLE-MEZIE : .................A..............A.....C.......................A..................................... : 1000
2015.00422|PUUV|N|France|H.s.|59|ANOR|             : .................................................................................................... : 1000
2015.00488|PUUV|N|France|H.s.|08|SEDAN|            : .................A....................C.......................A..............................T...... : 1000
2015.00498|PUUV|N|France|H.s.|08|REMILLY-AILLICOUR : .................................................................................................... : 1000
2016.00295|PUUV|N|France|H.s.|08|CORNY-MACHEROMENI : .................A.................................................................................. : 1000
2016.00333|PUUV|N|France|H.s.|59|FOURMIES|         : .................................................................................................... : 1000
2016.00345|PUUV|N|France|H.s.|59|FOURMIES|         : .................................................................................................... : 1000
2016.00357|PUUV|N|France|H.s.|02|HIRSON|           : .................................................................................................... : 1000
2016.00427|PUUV|N|France|H.s.|59|FOURMIES|         : ..............T.......................C............................................................. : 1000
2016.00469|PUUV|N|France|H.s.|02|PREMONTRE|        : ........A..G.....A..............C.................T...........A..............................T...... : 1000
AJ277075.1|PUUV|N|Belgium|M.g.|Montbliart/CG14444| : .................A....................C........T.............................................T...... : 1000
2014.00053|PUUV|N|France|H.s.|08|SECHEVAL|         : .................A....................C.......................A..............................T...... : 1000
KT247593.1|PUUV|N|France|M.g.|08|Ardennes/Mg75/201 : .................A..............A.....C........T.............................................T...... : 1000
KT247592.1|PUUV|N|France|M.g.|08|Ardennes/Mg156/20 : .................A..............A.....C........T.............................................T...... : 1000
2015.00019|PUUV|N|France|H.s.|08|ETEIGNIERES|      : .................A..............A.....C.....G..T.............................................T...... : 1000
2016.00239|PUUV|N|France|H.s.|08|VIREUX-MOLHAIN|   : .................A..............A.....C........T.....A.......................................T...... : 1000
2015.00329|PUUV|N|France|H.s.|08|BOGNY-SUR-MEUSE|  : .................A..............A.....C........T.....A.......................................T...... : 1000
2014.00209|PUUV|N|France|H.s.|08|TREMBLOIS-LES-ROC : .................A..............A.....C.....G..T..............A..............................T...... : 1000
2015.00456|PUUV|N|France|H.s.|94|ALFORTVILLE|      : .................A..............A.....C........T.....A........A..............................T...... : 1000
2012.00018|PUUV|N|France|H.s.|08|ROCROI|           : .................A..............A.....C.....G..T..............A..............................T...... : 1000
AJ277032.1|PUUV|N|Belgium|M.g.|Momignies/47Cg/96|  : ..C..............A..............A.....C.....G..T.............................................T...... : 1000
AJ277034.1|PUUV|N|Belgium|M.g.|Couvin/59Cg/97|     : .................A..............A.....C.....G..T.............................................T...... : 1000
AJ277033.1|PUUV|N|Belgium|M.g.|Momignies/55Cg/96|  : ..C..............A..............A.....C.....G..T.............................................T...... : 1000
2012.00638|PUUV|N|France|H.s.|08|GIVET|            : .....C...........A..............A.....C........T.............................................T...... : 1000
2015.00328|PUUV|N|France|H.s.|08|VRIGNE-MEUSE|     : .................A.................C........G................................................T...... : 1000
2015.00419|PUUV|N|France|H.s.|08|DOUZY|            : .................A....................C.....G.....T...........A..............................T...... : 1000
2016.00310|PUUV|N|France|H.s.|08|THIN-LE-MOUTIER|  : .................A...........................................................................T...... : 1000
AJ277030.1|PUUV|N|Belgium|M.g.|Thuin/33Cg/96|      : .....C...........A....................C.....G.................T..............................T...... : 1000
AJ238779.1|PUUV|N|Germany|M.g.|NRW/Cg-Erft|        : ...........G.....A..............A.....C...........T..A........A.....C.....C..................T...... : 1000
2014.00276|PUUV|N|France|H.s.|59|LILLE|            : ..C..............A....................C........T..T...........A.....C....................C...T...... : 1000
U22423.1|PUUV|N|Belgium|M.g.|CG13891|              : .....C.....GT...................A.....C........T..T................................................. : 1000
2014.00321|PUUV|N|France|H.s.|59|FOURMIES|         : ...............................................T.................................................... : 1000
2015.00457|PUUV|N|France|H.s.|55|REVIGNY-SUR-ORNAI : ........A..G.....A........A.....C..............T..T...........A..........................C...T...... : 1000
2013.00250|PUUV|N|France|H.s.|62|VENDIN-LS-BTHUNE| : ...........GT....A..............A.....C...........T..A........A..G.......................C...T...... : 1000
2016.00282|PUUV|N|France|H.s.|59|MORBECQUE|        : ...........GT....A..............A.....C........T..T..A...............G...................C.......... : 1000
AJ277076.1|PUUV|N|Belgium|M.g.|Montbliart/CG14445| : .................A....................C........T.............................................T...... : 1000
2014.00153|PUUV|N|France|H.s.|02|BUIRONFOSSE|      : .................A..............A.....C.....G..T.....A....................CA.................T...... : 1000
2014.00171|PUUV|N|France|H.s.|59|SAINT-SAULVE|     : ...G.C.....GT....A........T.....A.....C........T..T..A........A..........................C...T...... : 1000
2016.00293|PUUV|N|France|H.s.|60|LA-NEUVILLE-SUR-R : ...........GT....A........T.....A.....C........T..T..A........A........C.................A...T...... : 1000
2014.00499|PUUV|N|France|H.s.|94|CHAMPIGNY-SUR-MAR : .................A..............A.....C.....G..T...........................A.................T...... : 1000
2012.00057|PUUV|N|France|H.s.|59|COUSOLRE|         : .................A....................C.....G..T...........................A.................T...... : 1000
2012.00349|PUUV|N|France|H.s.|02|ENGLANCOURT|      : .................A..............A.....C.....G..T.....A....................CA.................T...... : 1000
2014.00135|PUUV|N|France|H.s.|02|ATHIES-SOUS-LAON| : .................A..............A.....C.....G..T...........................A.................T...... : 1000
2014.00184|PUUV|N|France|H.s.|59|FOURMIES|         : .................A....................C........T...........................A.................T...... : 1000
2016.00182|PUUV|N|France|H.s.|08|BOULZICOURT|      : .................a....................C........T...........................A.................T...... : 1000
2016.00286|PUUV|N|France|H.s.|02|TRUCY|            : .................A..............A.....C.....G..T...........................A.................T...... : 1000
2016.00325|PUUV|N|France|H.s.|59|AULNOYE-AYMERIES| : .................A....................C.....G..T..............A............A.................T...... : 1000
2016.00452|PUUV|N|France|H.s.|02|LAON|             : .................A..............A.....C.....G..T...........................A.................T...... : 1000
2016.00467|PUUV|N|France|H.s.|02|AISNE|            : .................A..............A.....C........T....................C......A.................T...... : 1000
```

```
                                                                  ACCCAIAAACGIACACG   PPT986R   Bowen MD et al. J Med Virol 1997
                                                         *         920         *         940         *         960         *         980         *        1000
2014.00598|PUUV|N|France|H.s.|59|FOURMIES|         : GATAATATTGAAGCCCCGAATGCACCCTGGGTGTTTGCTTGTGCACCCGACCGGTGCCCACCGACATGTATTTATGTTGCAGGGATGGCTGAACTAGGTG : 1000
2015.00657|PUUV|N|France|H.s.|02|CILLY|            : ...........G.....a..............A..............T..T..A........A.....C.....CA..T.............GT....C. : 1000
2015.00526|PUUV|N|France|H.s.|08|MONTHERME|        : .................A..............A.....C.....G..T...........................A.................T...... : 1000
2015.00045|PUUV|N|France|H.s.|59|FOURMIES|         : .................a....................C........T...........................A.................T...... : 1000
2015.00430|PUUV|N|France|H.s.|02|MONTCORNET|       : .................A..............A.....C.....G..T...........................A.................T...... : 1000
2016.00311|PUUV|N|France|H.s.|02|LAON|             : .................A..............A.....C.....G..T...........................A.................T...... : 1000
AJ277031.1|PUUV|N|Belgium|M.g.|Montbliart/23Cg/96| : .................A....................C........T...........................A.................T...... : 1000
2016.00326|PUUV|N|France|H.s.|02|LAON|             : .................A..............A.....C.....G..T...........................A.................T...... : 1000
2016.00353|PUUV|N|France|H.s.|02|CESSIERES|        : .................A..............A.....C.....G..T...........................A.................T...... : 1000
2012.00061|PUUV|N|France|H.s.|02|LANISCOURT|       : .................A..............A.....C.....G..T...........................A.................T...... : 1000
2014.00174|PUUV|N|France|H.s.|02|ST-ERME-OUTRE-ET- : .................A..............A.....C.....G..T...........................A.................T...... : 1000
2016.00268|PUUV|N|France|H.s.|02|PRESLES-ET-THIERN : .................A..............A.....C.....G..T...........................A.................T...... : 1000
2015.00660|PUUV|N|France|H.s.|02|VIC-SUR-AISNE|    : .................A....................C........T..T...........T..............................T...... : 1000
2012.00025|PUUV|N|France|H.s.|51|SAINTE-MENEHOULD| : ........C..GT....T........T...........A.....T..T..T..A........A....................A.....A..GT...... : 1000
2015.00665|PUUV|N|France|H.s.|51|REIMS|            : ...........GT....T........T.....A.....A.....C..T..T..A........A..G.................A.....A..GT...... : 1000
2012.00307|PUUV|N|France|H.s.|54|COLOMBEY-LES-BELL : .....C.....GT....T........T.................T..T..T..A........A.....C........A.....A.....A...T...... : 1000
2012.00123|PUUV|N|France|H.s.|55|BAR-LE-DUC|       : ...........GT....T........T...........A.....T..T..T..A........A..........................A...T...... : 1000
2012.00278|PUUV|N|France|H.s.|51|REIMS|            : ...........GT....T........T.....A.....A.....T..T..T..A........A..G..C..............A.....A...T...... : 1000
2015.00185|PUUV|N|France|H.s.|68|FELLERING|        : ...........GT....T........T...........A.....T..T..T..A........A...........C........A.....A...T...... : 1000
KJ994776.1|PUUV|N|Germany|M.g.|Mu/07/1219|2007|    : ..C..C......T.T..T........A.....A.....C.....C..T..T..A........A..........................A..GT...... : 1000
2012.00086|PUUV|N|France|H.s|58|CHEVROCHES|        : ............T.A..T........T.....A.....c.....C..t..T..A.....T..A..........................a..gT...... : 1000
KT247595.1|PUUV|N|France|M.g.|45|Orleans/Mg29/2010 : ...........GT.A..C........T.....A..C..C.....C..T..T..A.....T..A..........................A...T...... : 1000
KT247594.1|PUUV|N|France|M.g.|45|Orleans/Mg23/2010 : ...........GT.A..C........T.....A..C..C.....C..T..T..A.....T..A..........................A...T...... : 1000
KY365004.1|PUUV|N|France|M.g.|45|Orleans/NCHA373/2 : ........C..GT.A..C........T.....A..C..C.....C..T..T..A.....T..A........C.................A..GT...... : 1000
2012.00301|PUUV|N|France|H.s.|25|MOUTHE|           : ..C........GT....T....................C.....C..T..T..A........A.....C..............A.....A.......... : 1000
AM695638.1|PUUV|N|France|M.g.|Mignovillard/CgY02/2 : ..C........GT....T....................C.....C..T..T..A........A.....C..............A.....A.......... : 1000
KT247597.1|PUUV|N|France|M.g.|39|Jura/Mg214/2010|  : ...........GT.T..T....................C.....C..T..T..A........A.....C..............A.....A.......... : 1000
KT247596.2|PUUV|N|France|M.g.|39|Jura/Mg2/2010|    : ...........GT.T..T....................C.....C..T..T..A........A.....C..............A.....A.......... : 1000
2012.00102|PUUV|N|France|H.s.|39|COISERETTE|       : ...........GT.T..T.....G..............C.....C..T..T..A........A.....C..............A.....A...T...... : 1000
2012.00536|PUUV|N|France|H.s.|39|LA-PESSE|         : ...........GT.T..T.....G..............C.....C..T..T..A........A.....C..............A.....A.......... : 1000
2014.00622|PUUV|N|France|H.s.|39|ARBOIS|           : ...........GT....T....................C.....C..T..T..A........A.....C..............A.....A.......... : 1000
2015.00567|PUUV|N|France|H.s.|70|RIOZ|             : ...........GT.T..T........T...........C.....C..T..T..A........A.....C..............A.....A...T...... : 1000
2014.00637|PUUV|N|France|H.s.|25|SAULES|           : ...........GT.T..T........T...........C.....C..T..T..A........A.....C..............A.....A...T...... : 1000
2012.00396|PUUV|N|France|H.s.|39|SAINT-CLAUDE|     : ...........GT....T....................C.....C..T..T..A........A.....C..............A.....A.......... : 1000
2014.00120|PUUV|N|France|H.s.|38|LE-MOUTARET|      : ...........GT....T....................C.....C..T..T..A........A.....C..............A.....A.......... : 1000
2015.00153|PUUV|N|France|H.s.|73|GREZY-SUR-ISERE|  : ...........GT....T....................C.....C..T..T..A........A.....C..............A.....A.......... : 1000
2015.00504|PUUV|N|France|H.s.|70|RONCHAMP|         : .....C.....GT.T..T..............A.....A.....T..T..T..A..............C..............A.....A...T...... : 1000
2016.00275|PUUV|N|France|H.s.|21|JALLANGES|        : ...........GT.T..T....................C.....C..T..T..A........A.....C..............A.....A.......... : 1000
2016.00320|PUUV|N|France|H.s.|25|SAINT-VIT|        : ..C........GT.T..T.G..................C.....C..T..T..A........A.....C..............A.....A.......... : 1000
2015.00410|PUUV|N|France|H.s.|70|ANGIREY|          : .....C.....GT.T..T..............A.....A.....T..T..T..A..............C....................A...T...... : 1000
DQ016430.2|PUUV|N|Germany|M.g.|Bavaria/CG33/04|    : ..C..C.....GT.T..T.....C..A.....A.....A.....C..T...A.A........A..T.................A.....A..GT...... : 1000
DQ016432.2|PUUV|N|Germany|M.g.|Bavaria/CG41/04|    : ..C..C.....GT.T..T.....C..A.....A.....A.....C..T...A.A........A..T..............G..A.....A..GT...... : 1000
AY954723.2|PUUV|N|Germany|M.g.|Bavaria-CG34/04|    : ..C..C.....GT.T..A.....C..A...........A.....C..T...A.A........A..T..............G..A.....A..GT...... : 1000
AY954722.2|PUUV|N|Germany|M.g.|Bavaria-CG9/04|     : ..C..C.....GT.T..T.....C..A.....A.....A.....C..T...A.A........A..T.................A.....A..GT...... : 1000
2012.00402|PUUV|N|France|H.s.|60|GOUVIEUX|         : ..C........gT.T..T........T...........C.....T..t..t..A........A..........................A..GT...... : 1000
2014.00540|PUUV|N|France|H.s.|60|CHAMBLY|          : ...........GT.T..T........T...........C.....T..T..T..A........A..........................A..GT...... : 1000
AJ314600.1|PUUV|N|Balkan|M.g.|Balkan-1|            : ..C..C..A...T....T........A.....A.....A.....T..A..TA....T.....A.....C..........................T.... : 1000
AJ314601.1|PUUV|N|Balkan|M.g.|Balkan-2|            : ..C..C..A...T....T........A.....A..C..A.....T..A..TA....T.....A.....C..C.......................T.... : 1000
FN377821.1|PUUV|N|Hungary|M.g.|Mg9/HungaryTR17/00| : ..C..C..A...T....T........A.....A.....A.....T..A..TA....T.....A.....C..C.......................T.... : 1000
FN377822.1|PUUV|N|Hungary|M.g.|Mg23/HungaryTR17/00 : ..C..C..A...T....T........A.....A.....A.....T..A..TA....T.....A.....C..C.......................T.... : 1000
GQ339473.1|PUUV|N|Sweden|M.g.|Kiviniemi/Mg3/05|    : .....C......T.T..T........A.....T.....A.....T..T..T...........A..C.....C...........A.....A.....C.... : 1000
GQ339474.1|PUUV|N|Sweden|M.g.|Kiviniemi/Mg5/05|    : .....C......T.T..T........A.....T.....A.....T..T..T...........A..C.....C...........A.....A.....C.... : 1000
GQ339475.1|PUUV|N|Sweden|M.g.|Kiviniemi/Mg6/05|    : .....C......T.T..T........A.....T.....A.....T..T..T...........A..C.....C...........A.....A.....C.... : 1000
GQ339476.1|PUUV|N|Sweden|M.g.|Aijajarvi/Mg7/05|    : .....C......T.T..T........A.....T.....A.....T..T..T...........A..C.....C...........A.....A.....C.... : 1000
```

```
                                                                      ACCCAIAAACGIACACG  PPT986R  Bowen MD et al. J Med Virol 1997
                                                         *       920         *       940         *       960         *       980         *      1000
2014.00598|PUUV|N|France|H.s.|59|FOURMIES|           : GATAATATTGAAGCCCCGAATGCACCCTGGGTGTTTGCTTGTGCACCCGACCGGTGCCCACCGACATGTATTTATGTTGCAGGGATGGCTGAACTAGGTG : 1000
GQ339477.1|PUUV|N|Sweden|M.g.|Aijajarvi/Mg9/05|      : .....C......T.T..T........A.....T.....A.....T..T..T...........A..C.....C...........A.....A.....C.... : 1000
GQ339478.1|PUUV|N|Sweden|M.g.|Jockfall/Mg12/05|      : ..C..C......T.T..C........A.....T.....A.....C..T..T...........A..T..C..C...........A.....A.....T.... : 1000
GQ339482.1|PUUV|N|Sweden|M.g.|Kalvudden/Mg22/05|     : ..C.........T....T........A.....T.....A.....C..T..T...........A..T.....C.................A.....T.... : 1000
GQ339479.1|PUUV|N|Sweden|M.g.|Moskosel/Mg17/05|      : ..C..C..C...T.T..T........A.....T.....A.....C..T..T...........A..C.....C...........A.....A.....T.... : 1000
GQ339481.1|PUUV|N|Sweden|M.g.|Ljustrask/Mg20/05|     : ..C..C..C...T....T........A.....T.....A.....C..T..T...........A..C.....C...........A.....A.....T.... : 1000
GQ339480.1|PUUV|N|Sweden|M.g.|Gyttjea/Mg19/05|       : ..C..C..C...T.T..T........A.....T.....A.....C..T..T..............C.....C...........A.....A..G..T.... : 1000
AY526219.1|PUUV|N|Sweden|Human|Umea/hu|              : .....C......T.T..T........G.....T.....A.....T..T........T........G.....C.................A.....T.... : 1000
AJ223380.1|PUUV|N|Sweden|M.g.|Tavelsjo/Cg81/94|      : ............T....T........A.....C.....A........T.....A..T.....A..T...........C.....A.....A.....T.... : 1000
U14137.1|PUUV|N|Bosnia-H.|M.g.|Vranica|              : ............T....T........A.....C.....A........T.....A..T.....A..T...........C.....A.....A.....T.... : 1000
AJ223371.1|PUUV|N|Sweden|M.g.|Huggberget/Cg36/94|    : .....C......T....T........A.....T.....A........T.....A..T.....A..T...........C.....A.....A.....T.... : 1000
Z48586.1|PUUV|N|Sweden|M.g.|Vindeln/L20Cg/83|        : ..C..C......T....A........A.....T.....A........T.....A..T.....A..T.................A.....A.....T.... : 1000
AJ223374.1|PUUV|N|Sweden|M.g.|Mellansel/Cg47/94|     : .....C......T.T..T........A.....T.....A.....T..T.....A..T.....A........C.....A...........A.....TA... : 1000
AJ223375.1|PUUV|N|Sweden|M.g.|Mellansel/Cg49/94|     : .....C......T.T..T........A.....T.....A........T.....A..T.....A........C.....A...........A.....T.... : 1000
AJ238791.1|PUUV|N|Denmark|M.g.|Fyn/19|               : ..............A..T........A.....A...T.A.....C..G..T..A........A.....C....................A.....T.... : 1000
AJ278092.1|PUUV|N|Denmark|M.g.|Fyn47|                : ..............A..T........A.....A.....A.....C..AC.TA..........A.....C....................A..G..T.... : 1000
AJ278093.1|PUUV|N|Denmark|M.g.|Fyn131|               : ..............A..T........A....GA.....A.....C..AC.TA..........A.....C....................A..G..T.... : 1000
AJ223368.1|PUUV|N|Norway|M.g.|Eidsvoll/1124v|        : ..C.G......GT.A..A........A.....T.....C.....T..T...A....T.....T........C........T..A...........T.... : 1000
AJ223369.1|PUUV|N|Norway|M.g.|Eidsvoll/Cg1138/87|    : ..C.G......GT.T..A........A.....T.....C.....T..T...A....T.....T........C...........A...........T.... : 1000
JN657228.1|PUUV|N|Latvia|M.g.|Jelgava/Mg149/2008|    : .....C.....GT....T........A.....A.....A...........T..A..T..G..A.....C..A.................A...T...... : 1000
KX757839.1|PUUV|N|Lithuania|M.g.|LT15/164|2015|      : ..C..C..A..GT.T..T........A.....A.....A.....C..T.....A........A.....C..A...........A.....A..GT...... : 1000
KX757840.1|PUUV|N|Lithuania|M.g.|LT15/174|2015|      : ..C..C..A...T.T..T.....G..A.....A.....A.....C..T.....A........A.....C..A...........A.....A..GT...... : 1000
KX815394.1|PUUV|N|Poland|M.g.|KS13/855|2009|         : ..C..C.....GT.T..T........A.....A.....A........T.....A........A.....C..A.................A.......... : 1000
KX757841.1|PUUV|N|Lithuania|M.g.|LT15/201|2015|      : ..C..C..A..GT.T..T........A.....A.....A.....C..T.....A........A.....C..A...........A.....A..GT...... : 1000
AJ314598.1|PUUV|N|Russia|M.g.|Baltic/49Cg/00|        : ...G.C..A..GT.T..C..C.....A.....A.....C........A.....A..T..T..A.....C....................A..GT.G.... : 1000
AJ314599.1|PUUV|N|Russia|M.g.|Baltic/205Cg/00|       : ...G.C..A..GT.T..T........G.....A..............A..T..A..T..T..A..........................A..G..G..C. : 1000
JN657229.1|PUUV|N|Latvia|M.g.|Madona/Mg99/2008|      : ...G.C..A...T.T..T........A...........C........A..T.....T.....A.....C....................A..GT...... : 1000
JN657232.1|PUUV|N|Latvia|M.g.|Madona/Mg233/2008|     : ...G.C..A...T.T..T........A...........C........A..T.....T.....A.....C....................A..GT...... : 1000
JN657230.1|PUUV|N|Latvia|M.g.|Jelgava/Mg136/2008|    : ...G.C..A...T.T..T........A...........C........A..T........T..A.....C....................A..G..G.... : 1000
JN657231.1|PUUV|N|Latvia|M.g.|Jelgava/Mg140/2008|    : ...G.C..A...T.T..T........A...........C........A..T........T..A.....C....................A..G..G.... : 1000
Z30707.1|PUUV|N|Russia|M.g.|Udmurtia/458Cg/88|       : ...G.C......T....T........G.....A.....C........A..T.....T..C..T.....C...........T............T.G.... : 1000
Z30706.1|PUUV|N|Russia|M.g.|Udmurtia/444Cg/88|       : ...G.C......T....T........G.....A.....C........A..T.....T..C..T.....C...........T............T.G.... : 1000
Z84204.1|PUUV|N|Russia|M.g.|Kazan|                   : ...G.C......T....T........G.....A.....C........A..T.....T..C..T.....C...........T............T.G.... : 1000
Z30708.1|PUUV|N|Russia|M.g.|Udmurtia/338Cg/92|       : ...G.C......T....T........G.....A.....C........A..T........C..T.....C...........T............T.G.... : 1000
Z21497.1|PUUV|N|Russia|M.g.|Udmurtia/894Cg/91|       : ...G.C......T....T........G.....A.....C........A..T........C..T.....C..........TT............T.G.... : 1000
AB433843.2|PUUV|N|Russia|M.g.|Samara_49/CG/2005|     : ...G.C..C...T....T........A.....A.....C........A..T.....T..C..A...........C.....T............T....C. : 1000
AB433845.2|PUUV|N|Russia|M.g.|Samara_94/CG/2005|     : ...G.C..C...T....T........A.....A..............A..T.....T..C..A.................T...........GT...... : 1000
L11347.1|PUUV|N|Russia|Human|P360|                   : ...G.C..C...T.T..T........A.....A..............A..T........C..A.................T............T...... : 1000
AB297665.2|PUUV|N|Russia|M.g.|DTK/Ufa-97|1997|       : ...G.C..C...T.T..T........A.....A..............A..T........C..A.................T............T...... : 1000
M32750.1|PUUV|N|Russia|M.g.|CG1820|                  : ...G.C..C...T.T..T........A.....A..............A..T........C..A.................T............T...... : 1000
AF442613.1|PUUV|N|Russia|M.g.|CG17/Baskiria-2001|    : ...G.C..C...T.T..T........A.....A..............A..T........C..A.................T............T...... : 1000
KX815395.1|PUUV|N|Poland|M.g.|KS14/118|2009|         : ...G.C..A..GT....T........T.....A..............A..T..C.....C..A.....C....................A..GT...... : 1000
GQ339483.1|PUUV|N|Sweden|M.g.|Bergsjobo/Mg25/05|     : ........A..GT.T..A........A.....T.....C.....T..A..T..A..T..G..T.....C..............T.....A..G..T.... : 1000
AJ223377.1|PUUV|N|Sweden|M.g.|Solleftea/Cg6/95|      : ........A..GT.T..A........A.....T..C..C.....T..A..T..A..T..G..T....................T.....A..G..T.... : 1000
GQ339484.1|PUUV|N|Sweden|M.g.|Faboviken/Mg26/05|     : ..C.....A..GT.T..A.....G..A...........C.....T..A..T..A..T.....T.....C..............C.....A..G..T.... : 1000
GQ339485.1|PUUV|N|Sweden|M.g.|Mangelbo/Mg1/05|       : ..C........GT.T..A........A...........C.....T..A..T..A..T.....T.....C..............T.....A..GT...... : 1000
GQ339486.1|PUUV|N|Sweden|M.g.|Munga/Mg2/05|          : ..C........GT.T..A........A...........C.....T..A..T..A..T.....T.....C..............T.....A..GT...... : 1000
GQ339487.1|PUUV|N|Sweden|M.g.|Munga/Mg16/05|         : ..C........GT.T..A........A...........A.....T..A..T..A..T.....T.....C..............T.....A..GT...... : 1000
AJ223376.1|PUUV|N|Sweden|M.g.|Solleftea/Cg3/95|      : .....C..A..GT.T..A.....G..A...........C.....T..A..T..A..T.....T.....C..............C.....A..G..T.... : 1000
AF367071.1|PUUV|N|Russia|M.r.|CRF366|                : ..C........CT.T..T...A.T..A.....A.....C.....T..A..T..A.....C..A.....C..............A........G..T.... : 1000
AF367064.1|PUUV|N|Russia|M.g.|CG144|                 : ..C........CT.T..T.....T..A.....A.....C.....T..A..T..A.....C..A.....C..............A........G..T.... : 1000
AF367065.1|PUUV|N|Russia|M.g.|CG168|                 : ..C........CT.T..T.....T..A.....A.....C.....T..A..T..A.....C..A.....C..............A........G..T.... : 1000
AF367068.1|PUUV|N|Russia|M.g.|CG315|                 : ..C........CT.T..T.....T..A.....A.....C.....T..A..T..A.....C..A.....C..............A........G..T.... : 1000
AF367069.1|PUUV|N|Russia|M.r.|CRF161|                : ..C........CT.T..T.....T..A.....A.....C.....T..A..T..A.....C..A.....C...........G..A........G..T.... : 1000
```

```
                                                                                ACCCAIAAACGIACACG  PPT986R  Bowen MD et al. J Med Virol 1997
                                                            *         920         *         940         *         960         *         980         *        1000
2014.00598|PUUV|N|France|H.s.|59|FOURMIES|         : GATAATATTGAAGCCCCGAATGCACCCTGGGTGTTTGCTTGTGCACCCGACCGGTGCCCACCGACATGTATTTATGTTGCAGGGATGGCTGAACTAGGTG : 1000
AF367070.1|PUUV|N|Russia|M.r.|CRF308|              : ..C........CT.T..T.....T..A.....A.....C.....T..A..T..A.....C..A.....C..............A........G..T.... : 1000
AF367066.1|PUUV|N|Russia|M.g.|CG215|               : ..C........CT.T..T.....T..A.....A.....C.....T..A..T..A.....C..A.....C..............A........G..T.... : 1000
AF367067.1|PUUV|N|Russia|M.g.|CG222|               : ..C........CT.T..T.....T..A.....A.....C.....T..A..T..A.....C..A.....C..............A........G..T.... : 1000
Z46942.1|PUUV|N|Finland|M.g.|Puumala/1324Cg/79|    : ..C..C.....TT....T........A.....A.....A........A..T..A..T.....A.............................G..T..G. : 1000
Z30702.1|PUUV|N|Finland|M.g.|Evo/12Cg/93|          : .....C.....TT.T..T.....G..A.....T.....A........A.....A..T.....A........C.................A..G..T..G. : 1000
Z30703.1|PUUV|N|Finland|M.g.|Evo/13Cg/93|          : .....C.....TT.T..T.....G..A.....T.....A........A.....A..T.....A........C.................A..G..T..G. : 1000
Z30704.1|PUUV|N|Finland|M.g.|Evo/14Cg/93|          : .....C.....TT.T..T.....G..A.....T.....A.....G..A.....A..T.....A........C.................A..G..T..G. : 1000
Z30705.1|PUUV|N|Finland|M.g.|Evo/15Cg/93|          : .....C.....TT.T..T.....G..A.....T.....A.....G..A.....A..T.....A........C.................A..G..T..G. : 1000
Z69985.1|PUUV|N|Finland|M.g.|Virrat/25Cg/95|       : .....C..C..CT.T..T........A.....C.....A........A........T.....A........C.................A..G..T..G. : 1000
JN831947.1|PUUV|N|Finland|Pieksamaki/human_lung/20 : .....C.....TT....T........A.....A.....A........A.....A..T.....A........C....................G..T..G. : 1000
JN831943.1|PUUV|N|Finland|Pieksamaki/Mg7/2008|    : .....C.....TT....T........A.....A.....A........A.....A..T.....A........C....................G..T..G. : 1000
JN831950.1|PUUV|N|Finland|Pieksamaki/human_kidney/ : .....C.....TT....T........A.....A.....A........A.....A..T.....A........C....................G..T..G. : 1000
JQ319166.1|PUUV|N|Finland|Konnevesi/Mg_O14B/2005|  : ..C..C.....TT....T.....G..A.....A.....A........A.....A..T.....A.............................G..T..G. : 1000
JQ319169.1|PUUV|N|Finland|Konnevesi/Mg_O27B/2005|  : ..C..C.....TT....T.....G..A.....A.....A........A.....A..T.....A.............................G..T..G. : 1000
JQ319167.1|PUUV|N|Finland|Konnevesi/Mg_O15B/2005|  : ..C..C.....TT....T........A.....A.....A........A.....A..T.....A.............................G..T..G. : 1000
JQ319164.1|PUUV|N|Finland|Konnevesi/Mg_O6B/2005|   : ..C..C.....TT....T.....G..A.....A.....A........A.....A..T.....A.............................G..T..G. : 1000
JQ319165.1|PUUV|N|Finland|Konnevesi/Mg_O9B/2005|   : ..C..C.....TT....T.....G..A.....A.....A........A.....A..T.....A.............................G..T..G. : 1000
JQ319170.1|PUUV|N|Finland|Konnevesi/Mg_O74B/2005|  : ..C..C.....TT....T.....G..A.....A.....A........A.....A..T.....A.............................G..T..G. : 1000
JQ319171.1|PUUV|N|Finland|Konnevesi/Mg_M114B/2005| : ..C..C.....TT....T........A.....A.....A........A.....A..T.....A.............................G..T..G. : 1000
AJ238788.1|PUUV|N|Russia|M.g.|Karhumaki|           : .....C.....CT.A..C........A.....C.....A........A.TT..A..T.....A........C.................A.....T..G. : 1000
AJ238789.1|PUUV|N|Russia|M.g.|Kolodozero|          : .....C.....CT.A..C........A.....C.....A........A..T..A..T..G..A........C...........A.....A..G..T..G. : 1000
AJ314597.1|PUUV|N|Finland|M.g.|Pallasjarvi/63Cg/98 : .....C.....CT.T..T........T.....C.....A........A.....A..T.....A........C.................G..G..T..G. : 1000
NC_005224.1|PUUV|N|Finland|M.g.|Sotkamo-revu-NCBI2 : ..C..C.....TT.G..T........A.....C.....A........A.....A........A........C.................A..G..T..G. : 1000
AJ238790.1|PUUV|N|Russia|M.g.|Gomselga|            : ..C..C.....CT.T..T........A.....C.....C........A..T.....T.....A........C.................A..G..C..G. : 1000
AB010731.1|Hokkaido-V|N|Japan|M.r.|Tobetsu-60Cr-93 : ..C........CT....A.....C..A.....A.....A.....C..T..TA..........A........C...........A.....A..GT....A. : 1000
AB010730.1|Hokkaido-V|N|Japan|M.r.|Kamiiso-8Cr-95| : ..C........CT....A.....C..A...........A.....C..T..TA..........A........C...........A.....A..G.....G. : 1000
JX046487.1|Muju-V|N|South-Korea|M.r.|11-5|2011     : ..C..C.......AT..A........A.....A...T.G.....T..T..TA.A........T.....CC.......G...........A.....T.... : 1000
JX046484.1|Muju-V|N|South-Korea|M.r.|11-4|2011|    : ..C..C.......AT..A........A.....A...T.G.....T..T..TA.A........T.....CC.......G..C........A.....T.... : 1000
JX028273.1|Muju-V|N|South-Korea|M.r.|11-1|2011|    : ..C..C.......AT..A........A.....A..CT.G.....T..T..TA.A........T......C.......G...........A..G..T.... : 1000
DQ138128.1|Muju-V|N|South-Korea|E.r.|00-18|        : ..C.....A..G.AT..T........A.....A..CT.C.....T..A..TA....T.....T......C.C........T..A.....C...T...... : 1000
DQ138140.1|Muju-V|N|South-Korea|E.r.|99-27|        : ..C.....A..G.AT..T........A.....A..CT.C.....T..A..TA....T.....T......C.C........T..A.....C...T...... : 1000
DQ138133.1|Muju-V|N|South-Korea|E.r.|96-1|         : ..C.....A..G..T..T........A.....A..CT.C.....T..A..TA....T..G..T......C.C.................C...T...... : 1000
DQ138142.1|Muju-V|N|South-Korea|E.r.|99-28|        : ..C.....A..G.AT..T........A.....A..CT.C.....T..A..TA....T.....T......C.C........T..A.....C...T...... : 1000
```

```
                                                                  *      1020         *      1040         *      1060         *      1080         *      1100
2014.00598|PUUV|N|France|H.s.|59|FOURMIES|        : CATTCTTTTCCATATTACAGGATATGAGAAATACCATTATGGCATCTAAAACTGTTGGAACAGCAGAAGAAAAGTTAAAAAAGAAATCATCATTTTATCA : 1100
2014.00233|PUUV|N|France|H.s.|59|FOURMIES|        : .....................................C...........G.................................................. : 1100
2014.00097|PUUV|N|France|H.s.|02|SAINT-MICHEL|    : .....................................C.............................................................. : 1100
2014.00488|PUUV|N|France|H.s.|08|SIGNY-LE-PETIT|  : .....................................C...................................................T.......... : 1100
2014.00613|PUUV|N|France|H.s.|59|FOURMIES|        : .....................................C................................G............................. : 1100
2015.00402|PUUV|N|France|H.s.|08|CHARLEVILLE-MEZIE : .....................................C...........G.......................A.......................... : 1100
2015.00422|PUUV|N|France|H.s.|59|ANOR|            : .................................................................................................... : 1100
2015.00488|PUUV|N|France|H.s.|08|SEDAN|           : .....................................C...........G.......................A.......................... : 1100
2015.00498|PUUV|N|France|H.s.|08|REMILLY-AILLICOUR : .................................................................................................... : 1100
2016.00295|PUUV|N|France|H.s.|08|CORNY-MACHEROMENI : .....................................C.....G.....G.................................................. : 1100
2016.00333|PUUV|N|France|H.s.|59|FOURMIES|        : .................................................................................................... : 1100
2016.00345|PUUV|N|France|H.s.|59|FOURMIES|        : .................................................................................................... : 1100
2016.00357|PUUV|N|France|H.s.|02|HIRSON|          : .................................................................................................... : 1100
2016.00427|PUUV|N|France|H.s.|59|FOURMIES|        : .................................................................................................... : 1100
2016.00469|PUUV|N|France|H.s.|02|PREMONTRE|       : ..................................T......................................A.......................... : 1100
AJ277075.1|PUUV|N|Belgium|M.g.|Montbliart/CG14444| : ..................................T..C.............................................................. : 1100
2014.00053|PUUV|N|France|H.s.|08|SECHEVAL|        : .....................................C...........G.......................A.......................... : 1100
KT247593.1|PUUV|N|France|M.g.|08|Ardennes/Mg75/201 : .....................................C........A..........................A.......................... : 1100
KT247592.1|PUUV|N|France|M.g.|08|Ardennes/Mg156/20 : .....................................C........A..................................................... : 1100
2015.00019|PUUV|N|France|H.s.|08|ETEIGNIERES|     : ...................A........G........C.............................................................. : 1100
2016.00239|PUUV|N|France|H.s.|08|VIREUX-MOLHAIN|  : .....................................C............................................................C.. : 1100
2015.00329|PUUV|N|France|H.s.|08|BOGNY-SUR-MEUSE| : ...............................C.....C...........G.................................................. : 1100
2014.00209|PUUV|N|France|H.s.|08|TREMBLOIS-LES-ROC : .....................................C.............................................................. : 1100
2015.00456|PUUV|N|France|H.s.|94|ALFORTVILLE|     : ...............................C.....C.............................................................. : 1100
2012.00018|PUUV|N|France|H.s.|08|ROCROI|          : .....................................C.............................................................. : 1100
AJ277032.1|PUUV|N|Belgium|M.g.|Momignies/47Cg/96| : ............................G.....T..C.........................................G.................... : 1100
AJ277034.1|PUUV|N|Belgium|M.g.|Couvin/59Cg/97|    : .....................................C.............................................................. : 1100
AJ277033.1|PUUV|N|Belgium|M.g.|Momignies/55Cg/96| : ............................G.....T..C.........................................G.................... : 1100
2012.00638|PUUV|N|France|H.s.|08|GIVET|           : .....................................C................................G............................. : 1100
2015.00328|PUUV|N|France|H.s.|08|VRIGNE-MEUSE|    : ..........T..........................C.............................................................. : 1100
2015.00419|PUUV|N|France|H.s.|08|DOUZY|           : ............................G........C........C................................G.................... : 1100
2016.00310|PUUV|N|France|H.s.|08|THIN-LE-MOUTIER| : .....................................C.....G.....G.................................................. : 1100
AJ277030.1|PUUV|N|Belgium|M.g.|Thuin/33Cg/96|     : .....................................C....................G......................................... : 1100
AJ238779.1|PUUV|N|Germany|M.g.|NRW/Cg-Erft|       : ..........T.....G....................C................................................G.............. : 1100
2014.00276|PUUV|N|France|H.s.|59|LILLE|           : ....T................................A.............................................................. : 1100
U22423.1|PUUV|N|Belgium|M.g.|CG13891|             : .....................................C....................T.....................................C.. : 1100
2014.00321|PUUV|N|France|H.s.|59|FOURMIES|        : .................................................................................................... : 1100
2015.00457|PUUV|N|France|H.s.|55|REVIGNY-SUR-ORNAI : ..................................T......................................A.......................... : 1100
2013.00250|PUUV|N|France|H.s.|62|VENDIN-LS-BTHUNE| : .....................................C.................C..C....................G..A................. : 1100
2016.00282|PUUV|N|France|H.s.|59|MORBECQUE|       : .............T.......................C.................C..C......................................... : 1100
AJ277076.1|PUUV|N|Belgium|M.g.|Montbliart/CG14445| : ..................................T..C.............................................................. : 1100
2014.00153|PUUV|N|France|H.s.|02|BUIRONFOSSE|     : ..................................T..C.............................................................. : 1100
2014.00171|PUUV|N|France|H.s.|59|SAINT-SAULVE|    : ..........T.....G....................C....................C.................G..G.................... : 1100
2016.00293|PUUV|N|France|H.s.|60|LA-NEUVILLE-SUR-R : ..........T..........................C.................C..C..G...................................... : 1100
2014.00499|PUUV|N|France|H.s.|94|CHAMPIGNY-SUR-MAR : .....................................C.............................................................. : 1100
2012.00057|PUUV|N|France|H.s.|59|COUSOLRE|        : ..........T.......................T..C.............................................................. : 1100
2012.00349|PUUV|N|France|H.s.|02|ENGLANCOURT|     : ..................................T..C.............................................................. : 1100
2014.00135|PUUV|N|France|H.s.|02|ATHIES-SOUS-LAON| : .....................................C.............................................................. : 1100
2014.00184|PUUV|N|France|H.s.|59|FOURMIES|        : ..................................T..C.............................................................. : 1100
2016.00182|PUUV|N|France|H.s.|08|BOULZICOURT|     : ..................................T..C.............................................................. : 1100
2016.00286|PUUV|N|France|H.s.|02|TRUCY|           : .....................................C.............................................................. : 1100
2016.00325|PUUV|N|France|H.s.|59|AULNOYE-AYMERIES| : ..........T.......................T..A.............................................................. : 1100
2016.00452|PUUV|N|France|H.s.|02|LAON|            : .....................................C.............................................................. : 1100
2016.00467|PUUV|N|France|H.s.|02|AISNE|           : .....................................C.............................................................. : 1100
```

```
                                                              *       1020         *       1040         *       1060         *       1080         *       1100
2014.00598|PUUV|N|France|H.s.|59|FOURMIES|         : CATTCTTTTCCATATTACAGGATATGAGAAATACCATTATGGCATCTAAAACTGTTGGAACAGCAGAAGAAAAGTTAAAAAAGAAATCATCATTTTATCA : 1100
2015.00657|PUUV|N|France|H.s.|02|CILLY|            : .....................................C...................................A...........G.............. : 1100
2015.00526|PUUV|N|France|H.s.|08|MONTHERME|        : .....................................C.............................................................. : 1100
2015.00045|PUUV|N|France|H.s.|59|FOURMIES|         : ..................................T..C........A.................G................................... : 1100
2015.00430|PUUV|N|France|H.s.|02|MONTCORNET|       : .....................................C.............................................................. : 1100
2016.00311|PUUV|N|France|H.s.|02|LAON|             : .....................................C.............................................................. : 1100
AJ277031.1|PUUV|N|Belgium|M.g.|Montbliart/23Cg/96| : ..................................T..C.............................................................. : 1100
2016.00326|PUUV|N|France|H.s.|02|LAON|             : .....................................C.............................................................. : 1100
2016.00353|PUUV|N|France|H.s.|02|CESSIERES|        : .....................................C.............................................................. : 1100
2012.00061|PUUV|N|France|H.s.|02|LANISCOURT|       : .....................................C.............................................................. : 1100
2014.00174|PUUV|N|France|H.s.|02|ST-ERME-OUTRE-ET- : .....................................C.............................................................. : 1100
2016.00268|PUUV|N|France|H.s.|02|PRESLES-ET-THIERN : .....................................C.............................................................. : 1100
2015.00660|PUUV|N|France|H.s.|02|VIC-SUR-AISNE|    : ..........T.......................T..C.............................................................. : 1100
2012.00025|PUUV|N|France|H.s.|51|SAINTE-MENEHOULD| : ..........A.................G.............................C..........................G...........C.. : 1100
2015.00665|PUUV|N|France|H.s.|51|REIMS|            : ....T.....A.................G..C.....C........C...........C........G........G........G.............. : 1100
2012.00307|PUUV|N|France|H.s.|54|COLOMBEY-LES-BELL : ....T................................C...........G........C..........................g.....C..C..... : 1100
2012.00123|PUUV|N|France|H.s.|55|BAR-LE-DUC|       : ....T.....A.................G........C.....................C........G........G........G...........C.. : 1100
2012.00278|PUUV|N|France|H.s.|51|REIMS|            : ....T.....A.................G........C.....................C........G........G........G...........C.. : 1100
2015.00185|PUUV|N|France|H.s.|68|FELLERING|        : ....T.....A..C..............G........C.....................C........G.................G...........C.. : 1100
KJ994776.1|PUUV|N|Germany|M.g.|Mu/07/1219|2007|    : ....T...........G.....C...........T..............G.................G.....A.......................... : 1100
2012.00086|PUUV|N|France|H.s|58|CHEVROCHES|        : ....T..C..T...........C.....G.....T..C........g...........C...................................C..... : 1100
KT247595.1|PUUV|N|France|M.g.|45|Orleans/Mg29/2010 : ....T..C..T...........C.....G.....T..C........A...........C........G.....A.......................... : 1100
KT247594.1|PUUV|N|France|M.g.|45|Orleans/Mg23/2010 : ....T.....T...........C.....G.....T..C........A...........C...............A.......................... : 1100
KY365004.1|PUUV|N|France|M.g.|45|Orleans/NCHA373/2 : ....T..C..T...........C.....G.....T..C........A...........C...............A.......................... : 1100
2012.00301|PUUV|N|France|H.s.|25|MOUTHE|           : ....T.....T.................G........C.....G..C...........C.....T........A.....G.....G...........C.. : 1100
AM695638.1|PUUV|N|France|M.g.|Mignovillard/CgY02/2 : ....T.....T.................G........C.....G..C...........C.....T........A.....G.....G...........C.. : 1100
KT247597.1|PUUV|N|France|M.g.|39|Jura/Mg214/2010|  : ....T.....T.................G........C.....G..C...........C.....T........A.....G.....G...........C.. : 1100
KT247596.2|PUUV|N|France|M.g.|39|Jura/Mg2/2010|    : ....T.....T.................G........C.....G..C...........C.....T........A.....G.....G...........C.. : 1100
2012.00102|PUUV|N|France|H.s.|39|COISERETTE|       : ....T.....T..........................C.....G..C...........C.....T........A...........G...........C.. : 1100
2012.00536|PUUV|N|France|H.s.|39|LA-PESSE|         : ....T.....T..........................C.....G..C...........C.....T........A...........G...........C.. : 1100
2014.00622|PUUV|N|France|H.s.|39|ARBOIS|           : ....T.....T.................G........C.....G..C...........C.....T........A.....G.....G.............. : 1100
2015.00567|PUUV|N|France|H.s.|70|RIOZ|             : ....T.....T.................G........C.....................C.....T.....G..A....GG.....G...........C.. : 1100
2014.00637|PUUV|N|France|H.s.|25|SAULES|           : ....T.....T.................G........C.....G.....G.........C.....T.....G.......GG.....G...........C.. : 1100
2012.00396|PUUV|N|France|H.s.|39|SAINT-CLAUDE|     : ....T.....T.................G........C.....G..C...........C.....T........A..G..G.....G...........C.. : 1100
2014.00120|PUUV|N|France|H.s.|38|LE-MOUTARET|      : ....T.....T.................G........C.....G..C...........C.....T........A.....G.....G...........C.. : 1100
2015.00153|PUUV|N|France|H.s.|73|GREZY-SUR-ISERE|  : ....T.....T.................G........C.....G..C...........C.....T........A.....G.....G...........C.. : 1100
2015.00504|PUUV|N|France|H.s.|70|RONCHAMP|         : ....T..C..T.................G........C........C........G..C.....T........A.....G.....G...........C.. : 1100
2016.00275|PUUV|N|France|H.s.|21|JALLANGES|        : ....T..C..T.................G........C.....G..C...........C.....T........AA....G.....G...........C.. : 1100
2016.00320|PUUV|N|France|H.s.|25|SAINT-VIT|        : ....T.....T.................G........C.....G..C...........C.....T.....G..A.....G.....G...........C.. : 1100
2015.00410|PUUV|N|France|H.s.|70|ANGIREY|          : ....T..C..T.................G........C..................G..C.....T........A.....G.....G...........C.. : 1100
DQ016430.2|PUUV|N|Germany|M.g.|Bavaria/CG33/04|    : .T..T.---.......G...........G.....T.......................C.....T..G.....AC.G........G.............. : 1097
DQ016432.2|PUUV|N|Germany|M.g.|Bavaria/CG41/04|    : .C..T..C.........G...........G.....T.......................C.....T..G.....AC.G........G.............. : 1100
AY954723.2|PUUV|N|Germany|M.g.|Bavaria-CG34/04|    : .C..T..C.........G...........G.....T.......................C.....T..G......C.G........G.............. : 1100
AY954722.2|PUUV|N|Germany|M.g.|Bavaria-CG9/04|     : .T..T..C.........G..............C..T..C.....T..C...........C.....T..G.....AC.G........G.............. : 1100
2012.00402|PUUV|N|France|H.s.|60|GOUVIEUX|         : ....T.....T...C.G....................C........C..G........C.....T........A.....G.....G...........C.. : 1100
2014.00540|PUUV|N|France|H.s.|60|CHAMBLY|          : ....T.....T...C.G....................C........A..G........C.....T........A.....G.G...G........C..C.. : 1100
AJ314600.1|PUUV|N|Balkan|M.g.|Balkan-1|            : .C..T.....A..CC.T..A..............T..A........C..G.....G..C.....T........AC.G..G...........C........ : 1100
AJ314601.1|PUUV|N|Balkan|M.g.|Balkan-2|            : .C..T.....A..CC.T..A..............T..A........C..G.....A..C.....T........A..G..G...........C........ : 1100
FN377821.1|PUUV|N|Hungary|M.g.|Mg9/HungaryTR17/00| : .C..T.....G..CC.T..A..............T..A........C..G.....G..C.....T........AC.G..G...........C........ : 1100
FN377822.1|PUUV|N|Hungary|M.g.|Mg23/HungaryTR17/00 : .C..T.....A..CC.T.................T..A........C..G.....G..C.....T........A..G..G...........C........ : 1100
GQ339473.1|PUUV|N|Sweden|M.g.|Kiviniemi/Mg3/05|    : .......C..G..T..G...........G..C..T..A.....C..A..G........C..G........G...C....G..A.....T.....C..... : 1100
GQ339474.1|PUUV|N|Sweden|M.g.|Kiviniemi/Mg5/05|    : ..........G..T..G...........G..C..T..A.....C..A..G........C..G........G...C....G..A.....T.....C..... : 1100
GQ339475.1|PUUV|N|Sweden|M.g.|Kiviniemi/Mg6/05|    : ..........G..T..G...........G..C..T..A.....C..A..G........C..G........G...C....G..A.....T.....C..... : 1100
GQ339476.1|PUUV|N|Sweden|M.g.|Aijajarvi/Mg7/05|    : ..........G..T..G...........G..C..T..A.....C..A..G........C..G........G...C....G..A.....T.....C..... : 1100
```

```
                                                              *        1020           *        1040           *        1060           *        1080           *        1100
2014.00598|PUUV|N|France|H.s.|59|FOURMIES|      : CATTCTTTTCCATATTACAGGATATGAGAAATACCATTATGGCATCTAAAACTGTTGGAACAGCAGAAGAAAAGTTAAAAAAGAAATCATCATTTTATCA : 1100
GQ339477.1|PUUV|N|Sweden|M.g.|Aijajarvi/Mg9/05| : ......C..G..T..G..........G..C..T..A.....C..A..G.......C..G.......G...C....G..A.....T.....C..... : 1100
GQ339478.1|PUUV|N|Sweden|M.g.|Jockfall/Mg12/05| : .........T..T..G..........G..C..T..A.....C..A..G....C..C.....T.....G..AC.T..G..A.....T........... : 1100
GQ339482.1|PUUV|N|Sweden|M.g.|Kalvudden/Mg22/05| : ......C..A..T..G..........G..C..T..A.....C..A..G.......C..T..T.....G...C.T..G..A.....T.....C..... : 1100
GQ339479.1|PUUV|N|Sweden|M.g.|Moskosel/Mg17/05| : ....T.....G..TC.G.............C.....A.....C..A..G.......C.....T.....G...C.T..G..A.....T........... : 1100
GQ339481.1|PUUV|N|Sweden|M.g.|Ljustrask/Mg20/05| : ....T..C..A..T..G.............C.....A.....C..A..G.......C.....T.....G...C.T..G..A.....T........... : 1100
GQ339480.1|PUUV|N|Sweden|M.g.|Gyttjea/Mg19/05| : ....T..C..G..TC.G.............C.....A.....C..A..G.......C.....T.....G...C.T..G..A.....T........... : 1100
AY526219.1|PUUV|N|Sweden|Human|Umea/hu| : .C..T.....A..TC.G..A..C.....G.......A.....C..A..G..C..A..C.....T.......AC.C..G..A.....T.....C..C.. : 1100
AJ223380.1|PUUV|N|Sweden|M.g.|Tavelsjo/Cg81/94| : ....T.....A..CC.G................T..A.....C..A..G..C..G..C.....T.........C.T..G..A.....T.....C..... : 1100
U14137.1|PUUV|N|Bosnia-H.|M.g.|Vranica| : ....T.....A..CC.G................T..A.....C..A..G..C..G..C.....T.........C.T..G..A.....T.....C..... : 1100
AJ223371.1|PUUV|N|Sweden|M.g.|Huggberget/Cg36/94| : ....T.....A..CC.G................T..A.....C..A..G..C..A..C..C..T.....G...C.T..G..A..G..T.....C..... : 1100
Z48586.1|PUUV|N|Sweden|M.g.|Vindeln/L20Cg/83| : ....T.....A..CC.G.............C..T..A........A..G.....G..C.....T.....G...C.T..G..A.....T.....C..C.. : 1100
AJ223374.1|PUUV|N|Sweden|M.g.|Mellansel/Cg47/94| : .T..T..C..A..TC.G..A..C.....G.......A.....C..A..G..C..A..C.....T.......AC.T..G..A.....T.....C..... : 1100
AJ223375.1|PUUV|N|Sweden|M.g.|Mellansel/Cg49/94| : .T..T..C..A..TC.G..A..C.....G.......A.....C..A..G..C..A..C.....T.......AC.T..G..A.....T.....C..... : 1100
AJ238791.1|PUUV|N|Denmark|M.g.|Fyn/19| : .C..T..C.....C.....A.......G.....T..C...............A..C...............C....G..A........T..C..... : 1100
AJ278092.1|PUUV|N|Denmark|M.g.|Fyn47| : .C..T..C.....C.....A.......G...G.T..C...............A..C...............C.G..G..A.....G..C..C..... : 1100
AJ278093.1|PUUV|N|Denmark|M.g.|Fyn131| : .C..T..C.....C.....A.......G.....T..C...............A..C...............C.G..G..A.....G..C..C..... : 1100
AJ223368.1|PUUV|N|Norway|M.g.|Eidsvoll/1124v| : .C........A..T.............G.....G..A........A..G..A..G..C...........G..A.....G..A........T..C..C.. : 1100
AJ223369.1|PUUV|N|Norway|M.g.|Eidsvoll/Cg1138/87| : .C........A..T.............G.....G..A........A..G..A..G..C..............A.....G..A........T..C..C.. : 1100
JN657228.1|PUUV|N|Latvia|M.g.|Jelgava/Mg149/2008| : .C..T.....A..TC.......C.............A...............C..C.....T.....G.......G..A.....C..T..C..... : 1100
KX757839.1|PUUV|N|Lithuania|M.g.|LT15/164|2015| : ....T.....A..CC....A.......G........A..................T.....T.......A..G..G..A.....C........C.. : 1100
KX757840.1|PUUV|N|Lithuania|M.g.|LT15/174|2015| : ....T.....A..C.....A.......G........A..................T.....T.......A..G..G..A.....C........C.. : 1100
KX815394.1|PUUV|N|Poland|M.g.|KS13/855|2009| : .C..T.....A..CC.......C.............A...........G.......C.....T.....G..A..G..G........C..C........ : 1100
KX757841.1|PUUV|N|Lithuania|M.g.|LT15/201|2015| : ....T.....A..CC....A.......G........A..................T.....T.......A..G..G..A.....C........C.. : 1100
AJ314598.1|PUUV|N|Russia|M.g.|Baltic/49Cg/00| : ....T..C..T..T..G.......................C.....G.....A........T.....G..AC.G..G..A.....C.....C..C.. : 1100
AJ314599.1|PUUV|N|Russia|M.g.|Baltic/205Cg/00| : ....T..C..T..C..G..........G.G..........C.....G.....A........C.....G..A..G..G..A.....C........C.. : 1100
JN657229.1|PUUV|N|Latvia|M.g.|Madona/Mg99/2008| : ....T..C..T..T..G.............C.........C..C..G.....A........T.....G..AC.G..G..A.....C.....C..C.. : 1100
JN657232.1|PUUV|N|Latvia|M.g.|Madona/Mg233/2008| : ....T..C..T..T..G.............C.........C..C..G.....A........T.....G..AC.G..G..A.....C.....C..C.. : 1100
JN657230.1|PUUV|N|Latvia|M.g.|Jelgava/Mg136/2008| : ....T..C..T..T..G.......................C.....G.....A........T.....G..AC.G..G..A.....C........C.. : 1100
JN657231.1|PUUV|N|Latvia|M.g.|Jelgava/Mg140/2008| : ....T..C..T..T..G.......................C.....G.....A........T.....G..AC.G..G..A.....C........C.. : 1100
Z30707.1|PUUV|N|Russia|M.g.|Udmurtia/458Cg/88| : .........G..CC.............G..C..T..................A..T..............A...........G..C..C..C..... : 1100
Z30706.1|PUUV|N|Russia|M.g.|Udmurtia/444Cg/88| : .........G..CC.............G..C..T...............C..A..T..............A...........G..C..C..C..... : 1100
Z84204.1|PUUV|N|Russia|M.g.|Kazan| : .........G..CC.............G..C..T..................A..T..............A...........G..C..C..C..... : 1100
Z30708.1|PUUV|N|Russia|M.g.|Udmurtia/338Cg/92| : .........G..CC.............G..C..T..................A..T..............A...........G..C..C..C..... : 1100
Z21497.1|PUUV|N|Russia|M.g.|Udmurtia/894Cg/91| : .........G..CC.............G..C..T..................A..T..............A...........G..C..C..C..... : 1100
AB433843.2|PUUV|N|Russia|M.g.|Samara_49/CG/2005| : ....T.....T..CC....A.......G..C..T..............G.....A..C..............A.....G.......T..C..C..... : 1100
AB433845.2|PUUV|N|Russia|M.g.|Samara_94/CG/2005| : ....T.....T..TC............G..C.....C...............C..A..C..............A.....G..A.....C..C..C..... : 1100
L11347.1|PUUV|N|Russia|Human|P360| : ............C..............G..C..................G..C...........G..AC.G........G..C..C..C..... : 1100
AB297665.2|PUUV|N|Russia|M.g.|DTK/Ufa-97|1997| : ............C..............G..C..................G..C...........G..AC.G........G..C..C..C..... : 1100
M32750.1|PUUV|N|Russia|M.g.|CG1820| : ............C..............G..C..................G..C...........G..AC.G........G..C..C..C..... : 1100
AF442613.1|PUUV|N|Russia|M.g.|CG17/Baskiria-2001| : ............C..............G..C..T...............G..C...........G..AC.G........G..C..C..C..... : 1100
KX815395.1|PUUV|N|Poland|M.g.|KS14/118|2009| : .G..T.....T..TC.G..................C...........G.......G.....G.....G..A.....G.......T..C..C..... : 1100
GQ339483.1|PUUV|N|Sweden|M.g.|Bergsjobo/Mg25/05| : .C........A..C..G..........G.....A..A...........G.....A..........G......C....G..A........C.....C.. : 1100
AJ223377.1|PUUV|N|Sweden|M.g.|Solleftea/Cg6/95| : .C........A..C..G..........G.....A..A...........G.....A........T.......C....G..A........C.....C.. : 1100
GQ339484.1|PUUV|N|Sweden|M.g.|Faboviken/Mg26/05| : .C..T.....A..C..G................A..A...........G.....A.....T...............G..A........T.....C.. : 1100
GQ339485.1|PUUV|N|Sweden|M.g.|Mangelbo/Mg1/05| : .C.....C..A..CC....A.............A..C...............G..T........G..G.......G..A........C.....C.. : 1100
GQ339486.1|PUUV|N|Sweden|M.g.|Munga/Mg2/05| : .C.....C..A..CC....A.............A..C...............G..T........G..G.......G..A........C.....C.. : 1100
GQ339487.1|PUUV|N|Sweden|M.g.|Munga/Mg16/05| : .C.....C..A..CC....A.............A..C...............G..T........G..G.......G..A........C.....C.. : 1100
AJ223376.1|PUUV|N|Sweden|M.g.|Solleftea/Cg3/95| : .C........A..C..G................A..A...........G..C..A.....C..T...............G..A........T.....C.. : 1100
AF367071.1|PUUV|N|Russia|M.r.|CRF366| : .T........A...C.T.............C.....A........A..G...................G...C....G.......C..C..C..... : 1100
AF367064.1|PUUV|N|Russia|M.g.|CG144| : .T........A...C.T.............C.....A........A..G...................G...C....G.......C..C..C..... : 1100
AF367065.1|PUUV|N|Russia|M.g.|CG168| : .T........A...C.T.............C.....A........A..G...................G...C....G.......C..C..C..... : 1100
AF367068.1|PUUV|N|Russia|M.g.|CG315| : .T........A...C.T.............C.....A........A..G...................G...C....G.......C..C..C..... : 1100
AF367069.1|PUUV|N|Russia|M.r.|CRF161| : .T........A...C.T.............C.....A........A..G...................G...C....G.......C..C..C..... : 1100
```

```
                                                                  *        1020         *        1040         *        1060         *        1080         *        1100
2014.00598|PUUV|N|France|H.s.|59|FOURMIES|         : CATTCTTTTCCATATTACAGGATATGAGAAATACCATTATGGCATCTAAAACTGTTGGAACAGCAGAAGAAAAGTTAAAAAAGAAATCATCATTTTATCA : 1100
AF367070.1|PUUV|N|Russia|M.r.|CRF308|              : .T........A...C.T..............C.....A........A..G....................G...C....G........C..C..C..... : 1100
AF367066.1|PUUV|N|Russia|M.g.|CG215|               : .T........A...C.T..............C.....A........A..G....................G...C....G........C..C..C..... : 1100
AF367067.1|PUUV|N|Russia|M.g.|CG222|               : .T........A...C.T..............C.....A........A..G....................G...C....G........C..C..C..... : 1100
Z46942.1|PUUV|N|Finland|M.g.|Puumala/1324Cg/79|    : .C..T.....A...C.G.....C...........A..A...........G........C..............A..G..G..A..G..C..T..C..C.. : 1100
Z30702.1|PUUV|N|Finland|M.g.|Evo/12Cg/93|          : .C..T.....A.......................A..A...........G........C.....T.....G.....G.GG.....G..C..T..C..C.. : 1100
Z30703.1|PUUV|N|Finland|M.g.|Evo/13Cg/93|          : .C..T.....A.......................A..A...........G........C.....T.....G.....G.GG.....G..C..T..C..C.. : 1100
Z30704.1|PUUV|N|Finland|M.g.|Evo/14Cg/93|          : .C..T.....A.......................A..A...........G........C.....T.....G.....G..G.....G..C..T..C..C.. : 1100
Z30705.1|PUUV|N|Finland|M.g.|Evo/15Cg/93|          : .C..T.....A.......................A..A...........G........C.....T.....G.....G..G.....G..C..C..C..C.. : 1100
Z69985.1|PUUV|N|Finland|M.g.|Virrat/25Cg/95|       : .C..T.....A.....G..A..............G..A...........G........C.....T.....G........G..A..G..C..T..C..C.. : 1100
JN831947.1|PUUV|N|Finland|Pieksamaki/human_lung/20 : .T..T.....A...C.G.................A..A.....G.....G........C.....T.....G........G..A..G..C..T..C..C.. : 1100
JN831943.1|PUUV|N|Finland|Pieksamaki/Mg7/2008|     : .T..T.....A...C.G.................A..A.....G.....G........C.....T.....G........G..A..G..C..T..C..C.. : 1100
JN831950.1|PUUV|N|Finland|Pieksamaki/human_kidney/ : .T..T.....A...C.G.................A..A.....G.....G........C.....T.....G........G..A..G..C..T..C..C.. : 1100
JQ319166.1|PUUV|N|Finland|Konnevesi/Mg_O14B/2005|  : .C..T.....A...C.C.....C...........A..A...........G........C..T..T.....G........G..A..G..C..T..C..C.. : 1100
JQ319169.1|PUUV|N|Finland|Konnevesi/Mg_O27B/2005|  : .C..T.....A...C.C.....C...........A..A...........G........C..T..T.....G........G..A..G..C..T..C..C.. : 1100
JQ319167.1|PUUV|N|Finland|Konnevesi/Mg_O15B/2005|  : .C..T.....A...C.C.....C...........A..A...........G........C..T..T.....G........G..A..G..C..T..C..C.. : 1100
JQ319164.1|PUUV|N|Finland|Konnevesi/Mg_O6B/2005|   : .C..T.....A...C.C.....C...........A..A...........G........C..T..T.....G........G..A..G..C..T..C..C.. : 1100
JQ319165.1|PUUV|N|Finland|Konnevesi/Mg_O9B/2005|   : .C..T.....A...C.C.....C...........A..A...........G........C..T..T.....G........G..A..G..C..T..C..C.. : 1100
JQ319170.1|PUUV|N|Finland|Konnevesi/Mg_O74B/2005|  : .T..T.....A...C.C.....C...........A..A...........G........C..T.TT.....G........G..A..G..C..T..C..C.. : 1100
JQ319171.1|PUUV|N|Finland|Konnevesi/Mg_M114B/2005| : .C..T.....A...C.G.....C...........A..A...........G........C..T..T.....G........G..A..G..C..T..C..C.. : 1100
AJ238788.1|PUUV|N|Russia|M.g.|Karhumaki|           : .C..T.....A.....G...........G.....A..A...........G.....G..C..T..T.....G..A.....G..A.....C..T.....C.. : 1100
AJ238789.1|PUUV|N|Russia|M.g.|Kolodozero|          : .C..T.....A.....G.....C...........A..A.................C..C.....T.....G.....G..G..A........C.....C.. : 1100
AJ314597.1|PUUV|N|Finland|M.g.|Pallasjarvi/63Cg/98 : .C........A..T..G.....C.....G.....A..A........C..G........C.....T.....G........G..A.....C..C.....C.. : 1100
NC_005224.1|PUUV|N|Finland|M.g.|Sotkamo-revu-NCBI2 : .C..T.....A.....G.....C........C..A..A...........G........C...........G..A.....G..A..G..T..C.....C.. : 1100
AJ238790.1|PUUV|N|Russia|M.g.|Gomselga|            : .T..T.....A.....G...........G.....T........C..C..G........C..C..T.....G..A.....G........C..T.....C.. : 1100
AB010731.1|Hokkaido-V|N|Japan|M.r.|Tobetsu-60Cr-93 : .G........T..CC.G...........G..C.....A.................A..G.....T........AC.G..G..A........T..C..C.. : 1100
AB010730.1|Hokkaido-V|N|Japan|M.r.|Kamiiso-8Cr-95| : .............CC.G..A...........C.....A.....G..............G.....T........AC....G..A........T..C..C.. : 1100
JX046487.1|Muju-V|N|South-Korea|M.r.|11-5|2011     : ..........A..CC.C..A......C.G.....A..C........A.....A..G..C.....T..G..G..AC.T..G..A.....G........... : 1100
JX046484.1|Muju-V|N|South-Korea|M.r.|11-4|2011|    : ..........A..CC.C..A......C.G.....A..C........A.....A..G..C..G..T..G..G..AC.T..G..A.....G........... : 1100
JX028273.1|Muju-V|N|South-Korea|M.r.|11-1|2011|    : ..........A..CC.C..A......C.G.....A..C........A.....A..G..C.....T..G..G..AC.T..G..A.....G.....C..... : 1100
DQ138128.1|Muju-V|N|South-Korea|E.r.|00-18|        : .......C..A..CC.T.....C.....G.....A..A........C........A..C..T..T.........C.G..G........G........... : 1100
DQ138140.1|Muju-V|N|South-Korea|E.r.|99-27|        : .......C..A..CC.T.....C.....G.....A..A........C........A..C..T..T.........C.G..G........G........C.. : 1100
DQ138133.1|Muju-V|N|South-Korea|E.r.|96-1|         : .......C..A..CC.T.....C.....G.....A..A.................A..C..T..T.........C.G..G........G........... : 1100
DQ138142.1|Muju-V|N|South-Korea|E.r.|99-28|        : .......C..A..CC.T.....C.....G.....A..A........C........A..C..T..T.........C.G..G........G........C.. : 1100
```

```
                                                            *      1120         *      1140         *      1160         *      1180         *      1200
2014.00598|PUUV|N|France|H.s.|59|FOURMIES|         : ATCATACCTGCGGAGGACTCAATCAATGGGAATACAGCTTGACCAAAGAATTATCTTATTGTATATGCTTGAATGGGGCAAGGAAATGGTGGATCATTTT : 1200
2014.00233|PUUV|N|France|H.s.|59|FOURMIES|         : .................................................................................A.................C : 1200
2014.00097|PUUV|N|France|H.s.|02|SAINT-MICHEL|     : ...................................................................................................C : 1200
2014.00488|PUUV|N|France|H.s.|08|SIGNY-LE-PETIT|   : ...................................................................................................C : 1200
2014.00613|PUUV|N|France|H.s.|59|FOURMIES|         : ...................................................................................................C : 1200
2015.00402|PUUV|N|France|H.s.|08|CHARLEVILLE-MEZIE : .................................................................................A.................C : 1200
2015.00422|PUUV|N|France|H.s.|59|ANOR|             : .................................................................................................... : 1200
2015.00488|PUUV|N|France|H.s.|08|SEDAN|            : .................................................................................A.................C : 1200
2015.00498|PUUV|N|France|H.s.|08|REMILLY-AILLICOUR : ...................................................................................................C : 1200
2016.00295|PUUV|N|France|H.s.|08|CORNY-MACHEROMENI : .................................................................................A.................C : 1200
2016.00333|PUUV|N|France|H.s.|59|FOURMIES|         : ...................................................................................................C : 1200
2016.00345|PUUV|N|France|H.s.|59|FOURMIES|         : ...................................................................................................C : 1200
2016.00357|PUUV|N|France|H.s.|02|HIRSON|           : ...................................................................................................C : 1200
2016.00427|PUUV|N|France|H.s.|59|FOURMIES|         : ...................................................................................................C : 1200
2016.00469|PUUV|N|France|H.s.|02|PREMONTRE|        : .......T...........................................C...........C.................A.................. : 1200
AJ277075.1|PUUV|N|Belgium|M.g.|Montbliart/CG14444| : ..........................................................C.......................A.................C : 1200
2014.00053|PUUV|N|France|H.s.|08|SECHEVAL|         : .................................................................................A.................C : 1200
KT247593.1|PUUV|N|France|M.g.|08|Ardennes/Mg75/201 : ............................................................A....................A..G..............C : 1200
KT247592.1|PUUV|N|France|M.g.|08|Ardennes/Mg156/20 : ...C....................C...................................A....................A..G..............C : 1200
2015.00019|PUUV|N|France|H.s.|08|ETEIGNIERES|      : .......T.........................................................................A..G.....A........C : 1200
2016.00239|PUUV|N|France|H.s.|08|VIREUX-MOLHAIN|   : .................................................................................A..G..............C : 1200
2015.00329|PUUV|N|France|H.s.|08|BOGNY-SUR-MEUSE|  : .................................................................................A..G..............C : 1200
2014.00209|PUUV|N|France|H.s.|08|TREMBLOIS-LES-ROC : .......T.........................................................................A..G..............C : 1200
2015.00456|PUUV|N|France|H.s.|94|ALFORTVILLE|      : .................................................................................A..G..............C : 1200
2012.00018|PUUV|N|France|H.s.|08|ROCROI|           : .......T.........................................................................A..G..............C : 1200
AJ277032.1|PUUV|N|Belgium|M.g.|Momignies/47Cg/96|  : .................................................................................A..G..............C : 1200
AJ277034.1|PUUV|N|Belgium|M.g.|Couvin/59Cg/97|     : .................................................................................A..G..............C : 1200
AJ277033.1|PUUV|N|Belgium|M.g.|Momignies/55Cg/96|  : .................................................................................A..G..............C : 1200
2012.00638|PUUV|N|France|H.s.|08|GIVET|            : G................................................................................A..G..............C : 1200
2015.00328|PUUV|N|France|H.s.|08|VRIGNE-MEUSE|     : .........T.........................................A....................G........A.................C : 1200
2015.00419|PUUV|N|France|H.s.|08|DOUZY|            : .....................G.............................A....................G........A.................C : 1200
2016.00310|PUUV|N|France|H.s.|08|THIN-LE-MOUTIER|  : .................................................................................A.................C : 1200
AJ277030.1|PUUV|N|Belgium|M.g.|Thuin/33Cg/96|      : .....................G...........................................................A.................C : 1200
AJ238779.1|PUUV|N|Germany|M.g.|NRW/Cg-Erft|        : ......T..T.....................................................C.................A.................C : 1200
2014.00276|PUUV|N|France|H.s.|59|LILLE|            : G.................C............................................C...................................C : 1200
U22423.1|PUUV|N|Belgium|M.g.|CG13891|              : ...C...T..........A.............................................C.....G........A..A........T......... : 1200
2014.00321|PUUV|N|France|H.s.|59|FOURMIES|         : ...................................................................................................C : 1200
2015.00457|PUUV|N|France|H.s.|55|REVIGNY-SUR-ORNAI : .......T...........................................C...........C.................A.................. : 1200
2013.00250|PUUV|N|France|H.s.|62|VENDIN-LS-BTHUNE| : .......T.......A...................................C...........C.....G..........GA........A......... : 1200
2016.00282|PUUV|N|France|H.s.|59|MORBECQUE|        : ...............A...............................................C.................A........A........C : 1200
AJ277076.1|PUUV|N|Belgium|M.g.|Montbliart/CG14445| : ..........................................................C.......................A.................C : 1200
2014.00153|PUUV|N|France|H.s.|02|BUIRONFOSSE|      : ..........................................................C.......................A........A........C : 1200
2014.00171|PUUV|N|France|H.s.|59|SAINT-SAULVE|     : ......T................................................C.......C..............T..A.................C : 1200
2016.00293|PUUV|N|France|H.s.|60|LA-NEUVILLE-SUR-R : ................................................G..............C.................A.................C : 1200
2014.00499|PUUV|N|France|H.s.|94|CHAMPIGNY-SUR-MAR : .......T.....................................G............C.......................A........A........C : 1200
2012.00057|PUUV|N|France|H.s.|59|COUSOLRE|         : ..........................................................C.......................A.................C : 1200
2012.00349|PUUV|N|France|H.s.|02|ENGLANCOURT|      : ..............................G...........................C.......................A........A........C : 1200
2014.00135|PUUV|N|France|H.s.|02|ATHIES-SOUS-LAON| : .......T.....................................G............C.......................A........A........C : 1200
2014.00184|PUUV|N|France|H.s.|59|FOURMIES|         : .........A................................................C.......................A.................C : 1200
2016.00182|PUUV|N|France|H.s.|08|BOULZICOURT|      : .......T..................................................C.......................A.................C : 1200
2016.00286|PUUV|N|France|H.s.|02|TRUCY|            : .......T.....................................G............C.......................A........A........C : 1200
2016.00325|PUUV|N|France|H.s.|59|AULNOYE-AYMERIES| : ......T.......................G...........................C.......................A.................C : 1200
2016.00452|PUUV|N|France|H.s.|02|LAON|             : .......T.....................................G............C.......................A........A........C : 1200
2016.00467|PUUV|N|France|H.s.|02|AISNE|            : ..........................................................C.......................A........A......... : 1200
```

```
                                                              *        1120         *        1140         *        1160         *        1180         *        1200
2014.00598|PUUV|N|France|H.s.|59|FOURMIES|         : ATCATACCTGCGGAGGACTCAATCAATGGGAATACAGCTTGACCAAAGAATTATCTTATTGTATATGCTTGAATGGGGCAAGGAAATGGTGGATCATTTT : 1200
2015.00657|PUUV|N|France|H.s.|02|CILLY|            : .........T....................G................................C........G........A.................C : 1200
2015.00526|PUUV|N|France|H.s.|08|MONTHERME|        : ......................................................T...C......................A........A........C : 1200
2015.00045|PUUV|N|France|H.s.|59|FOURMIES|         : .....................G....................................C......................A.................C : 1200
2015.00430|PUUV|N|France|H.s.|02|MONTCORNET|       : ..........................................................C......................A........A........C : 1200
2016.00311|PUUV|N|France|H.s.|02|LAON|             : .......T.....................................G............C......................A........A........C : 1200
AJ277031.1|PUUV|N|Belgium|M.g.|Montbliart/23Cg/96| : ..........................................................C......................A........A........C : 1200
2016.00326|PUUV|N|France|H.s.|02|LAON|             : .......T.....................................G............C......................A........A........C : 1200
2016.00353|PUUV|N|France|H.s.|02|CESSIERES|        : .......T.....................................G............C......................A........A........C : 1200
2012.00061|PUUV|N|France|H.s.|02|LANISCOURT|       : .......T.....................................G............C......................A........A........C : 1200
2014.00174|PUUV|N|France|H.s.|02|ST-ERME-OUTRE-ET- : .......T.....................................G............C......................A........A........C : 1200
2016.00268|PUUV|N|France|H.s.|02|PRESLES-ET-THIERN : .......T.....................................G............C......................A........A........C : 1200
2015.00660|PUUV|N|France|H.s.|02|VIC-SUR-AISNE|    : .........A..................................................A..C.................A..G..............C : 1200
2012.00025|PUUV|N|France|H.s.|51|SAINTE-MENEHOULD| : G......T..........C.....C.................T........A..........................T..A..G.....T......... : 1200
2015.00665|PUUV|N|France|H.s.|51|REIMS|            : G......T..........C.....T.................T..............G....................T..A..G..............C : 1200
2012.00307|PUUV|N|France|H.s.|54|COLOMBEY-LES-BELL : g......T.A..A..A...................................C..T...C.A..C....................g..............C : 1200
2012.00123|PUUV|N|France|H.s.|55|BAR-LE-DUC|       : G......T..........C.....C.................T........C..........................T..A..G..............C : 1200
2012.00278|PUUV|N|France|H.s.|51|REIMS|            : G......T..........C.....T.................T........C......C...................T..A..G........C.....C : 1200
2015.00185|PUUV|N|France|H.s.|68|FELLERING|        : G......T..........C.....C.................T........C..........................T..A..G..............C : 1200
KJ994776.1|PUUV|N|Germany|M.g.|Mu/07/1219|2007|    : G.....TT.A..A..A....................A..G........G.............................T..A........A..C..C... : 1200
2012.00086|PUUV|N|France|H.s|58|CHEVROCHES|        : .........A..A..A.....G.................a...........C......C.A..C..............T..A..G.....A.....C... : 1200
KT247595.1|PUUV|N|France|M.g.|45|Orleans/Mg29/2010 : .........AA.A..A.....G........G........G...........C......C.A..................T.....G.....A.....C... : 1200
KT247594.1|PUUV|N|France|M.g.|45|Orleans/Mg23/2010 : .........AA.A..A.....G........G........G...........C......C.A..................T..A..G.....A.....C... : 1200
KY365004.1|PUUV|N|France|M.g.|45|Orleans/NCHA373/2 : .........AA.A..A.....G........G........G...........C......C.A..C...............T..A..G.....A..C..C... : 1200
2012.00301|PUUV|N|France|H.s.|25|MOUTHE|           : G......T....A.....C....................G.....G.....C......C.A..................T..............C..C... : 1200
AM695638.1|PUUV|N|France|M.g.|Mignovillard/CgY02/2 : G......T....A.....C....................G.....G.....C......C.A..................T..............C..C... : 1200
KT247597.1|PUUV|N|France|M.g.|39|Jura/Mg214/2010|  : G...........A.....C....................G.....G..G..C......C....................T..............C..C... : 1200
KT247596.2|PUUV|N|France|M.g.|39|Jura/Mg2/2010|    : G...........A.....C....................G.....G..G..C......C....................T..............C..C... : 1200
2012.00102|PUUV|N|France|H.s.|39|COISERETTE|       : G..........AA.....C....................A..T..G.....C......C.A..................T..............C..C... : 1200
2012.00536|PUUV|N|France|H.s.|39|LA-PESSE|         : G..........AA.....C....................A..T..G.....C......C.A..................T..............C..C... : 1200
2014.00622|PUUV|N|France|H.s.|39|ARBOIS|           : G......T....A.....C....................G.....G.....C......C.A..................T..............C..C... : 1200
2015.00567|PUUV|N|France|H.s.|70|RIOZ|             : G......T....A.....C....................G.....G.....C......C.A..................T..............C..C... : 1200
2014.00637|PUUV|N|France|H.s.|25|SAULES|           : G......T....A.....C....................G.....G.....C......C.A..................T..............C..C... : 1200
2012.00396|PUUV|N|France|H.s.|39|SAINT-CLAUDE|     : G......T....A.....C....................G.....G.....C......C.A..................T..............C..C... : 1200
2014.00120|PUUV|N|France|H.s.|38|LE-MOUTARET|      : G......T....A.....C....................G.....G.....C......C.A..................T..............C..C... : 1200
2015.00153|PUUV|N|France|H.s.|73|GREZY-SUR-ISERE|  : G......T....A.....C....................G.....G.....C......C.A..................T..............C..C... : 1200
2015.00504|PUUV|N|France|H.s.|70|RONCHAMP|         : ......TT....A...........C..............G.....G.....C.....G.....C...............T.................C... : 1200
2016.00275|PUUV|N|France|H.s.|21|JALLANGES|        : G.....T.....A.....C....................G.....G.....C......C.A..................T..............C..C... : 1200
2016.00320|PUUV|N|France|H.s.|25|SAINT-VIT|        : G...........A.....C..G.......................G.....C........A.T................T..............C..C... : 1200
2015.00410|PUUV|N|France|H.s.|70|ANGIREY|          : ......TT....A...........C...........A..G.....G.....C.....G.....................T.................C... : 1200
DQ016430.2|PUUV|N|Germany|M.g.|Bavaria/CG33/04|    : ......T..A..T..A.....G..............A..G..............TC.C....................T.....G.....A......... : 1197
DQ016432.2|PUUV|N|Germany|M.g.|Bavaria/CG41/04|    : ......T..A..T..A.....G..............A..G..............TC.C..A..................T..........T......... : 1200
AY954723.2|PUUV|N|Germany|M.g.|Bavaria-CG34/04|    : ......T..A..T..A.....G..............A..G..............TC.C..A..................T..........T......... : 1200
AY954722.2|PUUV|N|Germany|M.g.|Bavaria-CG9/04|     : ......TT.A..T..A.....G..............A..G..............TC.C..............G.....T..........A......... : 1200
2012.00402|PUUV|N|France|H.s.|60|GOUVIEUX|         : ..................C.................A..G...............C.T..A..C...............T..........A..C..C..C : 1200
2014.00540|PUUV|N|France|H.s.|60|CHAMBLY|          : G.................C.................A..G...............C.T..A..C...............T..........A..C..C..C : 1200
AJ314600.1|PUUV|N|Balkan|M.g.|Balkan-1|            : G..T...T..A...................G....................C...C.CC.A..C...............T..........T......... : 1200
AJ314601.1|PUUV|N|Balkan|M.g.|Balkan-2|            : G..T...T..A....A..............G....................C...C.CC.A..C...............T..........T..C...... : 1200
FN377821.1|PUUV|N|Hungary|M.g.|Mg9/HungaryTR17/00| : G..T...T..A...................G....................C...C.CC.A..C...............T..........T......... : 1200
FN377822.1|PUUV|N|Hungary|M.g.|Mg23/HungaryTR17/00 : G..T...T..A...................G.........................C.CC.A..C..........................T......... : 1200
GQ339473.1|PUUV|N|Sweden|M.g.|Kiviniemi/Mg3/05|    : ...T...T....AC....A..............C..A........G..G.....TC.C....T....T.A........A..A........T.....C... : 1200
GQ339474.1|PUUV|N|Sweden|M.g.|Kiviniemi/Mg5/05|    : ...T...T....AC....A..............C..A...........G.....TC.C....T....T.A........A..A........T.....C... : 1200
GQ339475.1|PUUV|N|Sweden|M.g.|Kiviniemi/Mg6/05|    : ...T...T....AC....A..............C..A...........G.....TC.C....T....T.A........A..A........T.....C... : 1200
GQ339476.1|PUUV|N|Sweden|M.g.|Aijajarvi/Mg7/05|    : ...T...T....AC....A..............C..A........G..G.....TC.C....T....T.A........A..A........T.....C... : 1200
```

```
                                                                      *        1120         *        1140         *        1160         *        1180         *        1200
2014.00598|PUUV|N|France|H.s.|59|FOURMIES|         : ATCATACCTGCGGAGGACTCAATCAATGGGAATACAGCTTGACCAAAGAATTATCTTATTGTATATGCTTGAATGGGGCAAGGAAATGGTGGATCATTTT : 1200
GQ339477.1|PUUV|N|Sweden|M.g.|Aijajarvi/Mg9/05|    : ...T...T....AC....A..............C..A........G..G.....TC.C....T....T.A........A..A........T.....C... : 1200
GQ339478.1|PUUV|N|Sweden|M.g.|Jockfall/Mg12/05|    : ...C...T.A..AC.A..A..G...........T..A.....T.....G.....TC.C....T......A........G..A........C..C..C... : 1200
GQ339482.1|PUUV|N|Sweden|M.g.|Kalvudden/Mg22/05|   : ...C...T....AC....A..G...........C..A.....T.....G.....TC.CC...T......A........A..A........T..C..C... : 1200
GQ339479.1|PUUV|N|Sweden|M.g.|Moskosel/Mg17/05|    : ...C...T.A..AC....A..G...........C.....C........G.....TC.CC.T.T....T.A........A..A........T..C..C... : 1200
GQ339481.1|PUUV|N|Sweden|M.g.|Ljustrask/Mg20/05|   : ...C.....A..AC....A..G...........C.....C........G.....TC.TC...T....T.A........T..A........T..C..C... : 1200
GQ339480.1|PUUV|N|Sweden|M.g.|Gyttjea/Mg19/05|     : ...C.....A..AC....A..G...........C.....C........G.....TC.CC...T....T.A..G.....A..A........T..C..C... : 1200
AY526219.1|PUUV|N|Sweden|Human|Umea/hu|            : G..C..TT.A...C....A..G...........C..A.....T.....G......C.C....................A..A..G.....T..C..C... : 1200
AJ223380.1|PUUV|N|Sweden|M.g.|Tavelsjo/Cg81/94|    : G..T..TT.A...C....A..G...........C........T..G..G.....TC.C..A.................A..A..G.....T..C...... : 1200
U14137.1|PUUV|N|Bosnia-H.|M.g.|Vranica|            : G..T..TT.A...C....A..G...........C........T..G..G.....TC.C..A.................A..A..G.....T..C..C... : 1200
AJ223371.1|PUUV|N|Sweden|M.g.|Huggberget/Cg36/94|  : ...T..TT.A...C....A..G...........C........T..G..G.....TC.C....................A..A..G.....T..C..C... : 1200
Z48586.1|PUUV|N|Sweden|M.g.|Vindeln/L20Cg/83|      : G..T..TT.A...C....A..G...........C.....G..T..G..G.....TC.C....................A..A..G.....T..C...... : 1200
AJ223374.1|PUUV|N|Sweden|M.g.|Mellansel/Cg47/94|   : G..T..TT.A...C....A..G...........C..............G......C.C....................A..A..G.....T..C..C... : 1200
AJ223375.1|PUUV|N|Sweden|M.g.|Mellansel/Cg49/94|   : G..T..TT.A...C....A..G...........C.......G......G......C.C....................A..A..G.....T..C..C... : 1200
AJ238791.1|PUUV|N|Denmark|M.g.|Fyn/19|             : ......T...A..C.A........C.....G..T.....G..............TC.T...........C........G..A..G.....A.....C..C : 1200
AJ278092.1|PUUV|N|Denmark|M.g.|Fyn47|              : ......T...A.AC.A..C.....CG....G..T.....A..............TC.TC...................A.GA..G.....T..C..C..C : 1200
AJ278093.1|PUUV|N|Denmark|M.g.|Fyn131|             : ......T...A.AC.A..C.....C.....G..T.....A..............TC.TC...................A.GA..G.....T..C..C..C : 1200
AJ223368.1|PUUV|N|Norway|M.g.|Eidsvoll/1124v|      : G..C..T......C.A..............G..T....................TC.T..A....................A..G.....A........C : 1200
AJ223369.1|PUUV|N|Norway|M.g.|Eidsvoll/Cg1138/87|  : G..C..T......C.A..............G..T....................TC.T..A....................A..G.....A........C : 1200
JN657228.1|PUUV|N|Latvia|M.g.|Jelgava/Mg149/2008|  : G........C..CC................G..............G.........C.TC..........A........T..............C.....C : 1200
KX757839.1|PUUV|N|Lithuania|M.g.|LT15/164|2015|    : .........A..AC.A.....G....................T..G.........C.C.....C...T.G..G.....T..A........A.....C..C : 1200
KX757840.1|PUUV|N|Lithuania|M.g.|LT15/174|2015|    : .........A...C.A.....G....................T..G.........C.C.....C...T.G..G.....T..A..............C..C : 1200
KX815394.1|PUUV|N|Poland|M.g.|KS13/855|2009|       : ...G.....A...C....C..G........G...........T............C.C...........G........T..A...........C..C..C : 1200
KX757841.1|PUUV|N|Lithuania|M.g.|LT15/201|2015|    : .........A..AC.A.....G....................T..G.........C.C.....C...T.G..G.....T..A........A.....C..C : 1200
AJ314598.1|PUUV|N|Russia|M.g.|Baltic/49Cg/00|      : .........A..CC....A.....T...........A.....T.....G..A...C.TC.A..C...T.G........A..A...........C..C..C : 1200
AJ314599.1|PUUV|N|Russia|M.g.|Baltic/205Cg/00|     : G........A..CC....A..G..T........C..A.....T........A...C.T..A..C...T.G........A..A...........C..C..C : 1200
JN657229.1|PUUV|N|Latvia|M.g.|Madona/Mg99/2008|    : ............CC....A.....G.....G..T..A.....T...C.G..A...C.TC.A..C...T.G........G..A...........C..C..C : 1200
JN657232.1|PUUV|N|Latvia|M.g.|Madona/Mg233/2008|   : ............CC....A.....G.....G..T..A.....T...C.G..A...C.TC.A..C...T.G........G..A...........C..C..C : 1200
JN657230.1|PUUV|N|Latvia|M.g.|Jelgava/Mg136/2008|  : G.....TT....CC....A...........G..T..A.....T.....G..A...C.TC.A..C...T.G..G.....A..A...........C..C..C : 1200
JN657231.1|PUUV|N|Latvia|M.g.|Jelgava/Mg140/2008|  : G.....TT....CC....A...........G..T..A.....T.....G..A...C.TC.A..C...T.G..G.....A..A...........C..C..C : 1200
Z30707.1|PUUV|N|Russia|M.g.|Udmurtia/458Cg/88|     : .........A..CC....A...........G..T..A.....T..G..G..A...C..C....C...T.G..G.....G..A.................C : 1200
Z30706.1|PUUV|N|Russia|M.g.|Udmurtia/444Cg/88|     : .........A..CC....A...........G..T..A.....T..G..G..A...C..C....C...T.G..G.....G..A.................C : 1200
Z84204.1|PUUV|N|Russia|M.g.|Kazan|                 : .........A..CC....A...........G..T..A.....T..G..G..A...C..C....C...T.G..G.....G..A.................C : 1200
Z30708.1|PUUV|N|Russia|M.g.|Udmurtia/338Cg/92|     : .........A..CC....A...........G..T..A.....T..G..G..A...C..C....C...T.G..G.....G..A.................C : 1200
Z21497.1|PUUV|N|Russia|M.g.|Udmurtia/894Cg/91|     : .........A..TC....A...........G..T..A.....T..G..G..A..TC..C.A..C...T.G..G.....G..A.................C : 1200
AB433843.2|PUUV|N|Russia|M.g.|Samara_49/CG/2005|   : G.....TT....CC....A.....G........T..A.....T..G..G..A......C....C...T.G..G.....A..A.................C : 1200
AB433845.2|PUUV|N|Russia|M.g.|Samara_94/CG/2005|   : G......T....CC....A..............T..A........G.....A...C.......C...T.G..G.....G..A.................C : 1200
L11347.1|PUUV|N|Russia|Human|P360|                 : ......TT....CC.A..A...........G..T..A.....T..G..G..A...C..C....C...T.G........A..A.................C : 1200
AB297665.2|PUUV|N|Russia|M.g.|DTK/Ufa-97|1997|     : ......TT....CC.A..A...........G..T..A.....T..G..G..A...C..C....C...T.G........A..A.................C : 1200
M32750.1|PUUV|N|Russia|M.g.|CG1820|                : ......TT....CC.A..A...........G..T..A.....T..G..G..A...C..C....C...T.G........A.GA.................C : 1200
AF442613.1|PUUV|N|Russia|M.g.|CG17/Baskiria-2001|  : ......TT....CC.A..A...........G..T..A.....T..G..G..A...C..C....C...T.G........A..A.................C : 1200
KX815395.1|PUUV|N|Poland|M.g.|KS14/118|2009|       : ......T..A..TC....A...........G..C..A.....T.....G..A...C.CC........T.A........A..A.................. : 1200
GQ339483.1|PUUV|N|Sweden|M.g.|Bergsjobo/Mg25/05|   : ...C..T.....AC..........C........C..A...........G.....TC.T.......................A..G..............C : 1200
AJ223377.1|PUUV|N|Sweden|M.g.|Solleftea/Cg6/95|    : ...C..T..A..AC..........C........C..A...........G.....TC.T.......................A..G..............C : 1200
GQ339484.1|PUUV|N|Sweden|M.g.|Faboviken/Mg26/05|   : ......T.....AC................G..C..A........G..G......C.T...........A...........A..G........C.....C : 1200
GQ339485.1|PUUV|N|Sweden|M.g.|Mangelbo/Mg1/05|     : G..T..T.....AC.......G...........C..A..................C.........................A..G.....A..C.....C : 1200
GQ339486.1|PUUV|N|Sweden|M.g.|Munga/Mg2/05|        : G..T..T.....AC.......G...........C..A..................C.........................A..G.....A..C.....C : 1200
GQ339487.1|PUUV|N|Sweden|M.g.|Munga/Mg16/05|       : G..T..T.....AC.......G...........C..A..................C.........................A..G.....A..C.....C : 1200
AJ223376.1|PUUV|N|Sweden|M.g.|Solleftea/Cg3/95|    : ...C..T.....AC.......G........G..C..A...........G......C.T...........A........T..A........A..C.....C : 1200
AF367071.1|PUUV|N|Russia|M.r.|CRF366|              : ...T..TT....CC.A........G..................T.....G......C.TC.A..C...T.A........T..A.................C : 1200
AF367064.1|PUUV|N|Russia|M.g.|CG144|               : ...T..TT....CC.A........G..................T.....G......C.TC.A..C...T.A........T..A.................C : 1200
AF367065.1|PUUV|N|Russia|M.g.|CG168|               : ...T..TT....CC.A........G..................T.....G......C.TC.A..C...T.A...........A.................C : 1200
AF367068.1|PUUV|N|Russia|M.g.|CG315|               : ...T..TT....CC.A........G..................T.....G......C.TC.A..C...T.A........T..A.................C : 1200
AF367069.1|PUUV|N|Russia|M.r.|CRF161|              : ...T..TT....CC.A........G..................T.....G......C.TC.A..C...T.A...........A.................C : 1200
```

```
                                                      *       1120         *       1140         *       1160         *       1180         *       1200
2014.00598|PUUV|N|France|H.s.|59|FOURMIES|         : ATCATACCTGCGGAGGACTCAATCAATGGGAATACAGCTTGACCAAAGAATTATCTTATTGTATATGCTTGAATGGGGCAAGGAAATGGTGGATCATTTT : 1200
AF367070.1|PUUV|N|Russia|M.r.|CRF308|              : ...T..TT....CC.A........G.................T.....G......C.TC.A..C...T.A........T..A.................C : 1200
AF367066.1|PUUV|N|Russia|M.g.|CG215|               : ...T..TT....CC.A........G.................T.....G......C.TC.A..C...T.A........T..A.................. : 1200
AF367067.1|PUUV|N|Russia|M.g.|CG222|               : ...T..TT....CC.A........G.................T.....G......C.TC.A..C...T.A........T..A.................. : 1200
Z46942.1|PUUV|N|Finland|M.g.|Puumala/1324Cg/79|    : ...T.....A..TC...................T..A........G.....C...C......TC...T.A........T..A..G..............C : 1200
Z30702.1|PUUV|N|Finland|M.g.|Evo/12Cg/93|          : ...T...T.A..CC...................T.................C..TC.T....TC...T.G.............................C : 1200
Z30703.1|PUUV|N|Finland|M.g.|Evo/13Cg/93|          : ...T...T.A..CC...................T.................C..TC.T....TC...T.G.............................C : 1200
Z30704.1|PUUV|N|Finland|M.g.|Evo/14Cg/93|          : ...T...T.A..CC...................T.................C..TC.C....TC...T.G.............................C : 1200
Z30705.1|PUUV|N|Finland|M.g.|Evo/15Cg/93|          : ...T...T.A..CC...................T.................C..TC.T....TC...T.G....................A........C : 1200
Z69985.1|PUUV|N|Finland|M.g.|Virrat/25Cg/95|       : ...T..TT.A..CC.A..............G..T..A.................TC.T....TC...T.G........T..A..G.....A........C : 1200
JN831947.1|PUUV|N|Finland|Pieksamaki/human_lung/20 : ...T...T....CC....C...........G..C..A.....T..G.....C...C......TC...T.A..G.....T..A..G...........C..C : 1200
JN831943.1|PUUV|N|Finland|Pieksamaki/Mg7/2008|     : ...T...T....CC....C...........G..C..A.....T..G.....C...C......TC...T.A..G.....T..A..G...........C..C : 1200
JN831950.1|PUUV|N|Finland|Pieksamaki/human_kidney/ : ...T...T....CC....C...........G..C..A.....T..G.....C...C......TC...T.A..G.....T..A..G...........C..C : 1200
JQ319166.1|PUUV|N|Finland|Konnevesi/Mg_O14B/2005|  : G..T...T.A..CC................T..T..A........G.........C.C....TC...T.G...........A..G..............C : 1200
JQ319169.1|PUUV|N|Finland|Konnevesi/Mg_O27B/2005|  : G..T...T.A..CC................T..T..A........G.........C.C....TC...T.G...........A..G..............C : 1200
JQ319167.1|PUUV|N|Finland|Konnevesi/Mg_O15B/2005|  : G..T..TT.A..CC...................C..A........G.........C.C..A.TC...T.G........T..A..G..............C : 1200
JQ319164.1|PUUV|N|Finland|Konnevesi/Mg_O6B/2005|   : G..T...T.A..CC................T..T..A........G.........C.C....TC...T.G...........A..G..............C : 1200
JQ319165.1|PUUV|N|Finland|Konnevesi/Mg_O9B/2005|   : G..T...T.A..CC................T..T..A........G.........C.C....TC...T.G...........A..G..............C : 1200
JQ319170.1|PUUV|N|Finland|Konnevesi/Mg_O74B/2005|  : G..T...T.A..CC................T..T..A........G.........C.C....TC...T.G..............G..............C : 1200
JQ319171.1|PUUV|N|Finland|Konnevesi/Mg_M114B/2005| : G..T..TT.A..CC...................C..A........G.........C.C..A.TC...T.G........T..A..G..............C : 1200
AJ238788.1|PUUV|N|Russia|M.g.|Karhumaki|           : G..T..TT.A..CC.A........G........T..............G..C..TC.C....TC...T.G...........A..G.....A......... : 1200
AJ238789.1|PUUV|N|Russia|M.g.|Kolodozero|          : ...T.....A..CC..........C........T.CA...........G..C..TC.T..A.TC...T.G.........C.A..G.....A.....C..C : 1200
AJ314597.1|PUUV|N|Finland|M.g.|Pallasjarvi/63Cg/98 : ...T...T.A..TC...................T..A.................TC.C....TC...T.G...........A..G.....A........C : 1200
NC_005224.1|PUUV|N|Finland|M.g.|Sotkamo-revu-NCBI2 : ...T........TC.A.................T........T............C.C....T....T.G...........A..G.....A.....C..C : 1200
AJ238790.1|PUUV|N|Russia|M.g.|Gomselga|            : ...T.....A..TC.A.................T.................C..TC.C....TC...T.G..G.....T..A..G...........C..C : 1200
AB010731.1|Hokkaido-V|N|Japan|M.r.|Tobetsu-60Cr-93 : ......TT.AA..C.T..............G..C..A...........G..A...C.G..A..C........G.....A..A..G...........C... : 1200
AB010730.1|Hokkaido-V|N|Japan|M.r.|Kamiiso-8Cr-95| : ......TT.AA..C.C..............G..T..A..C........G..A.....G..A..C........G.....A..A..G.....A.....C... : 1200
JX046487.1|Muju-V|N|South-Korea|M.r.|11-5|2011     : G.....TT..A..C....A.................A..G......C.G.....TC..C.T..C..............A...........A..C..C..C : 1200
JX046484.1|Muju-V|N|South-Korea|M.r.|11-4|2011|    : G.....TT..A..C....A.................A..G......C.G.....TC..C.T..C..............A...........A..C..C..C : 1200
JX028273.1|Muju-V|N|South-Korea|M.r.|11-1|2011|    : G.....TT..A..C....A.................A..G......C.G.....T...C.T..C..............A...........A..C..C..C : 1200
DQ138128.1|Muju-V|N|South-Korea|E.r.|00-18|        : .........CA..C....A..G...........C..A..G..T..G..G.....TC..C.C...........G.....A..A..G.....A.....C... : 1200
DQ138140.1|Muju-V|N|South-Korea|E.r.|99-27|        : .........CA..C....A..G...........C..A..G..T..G..G.....TC..C.C...........G.....A..A..G.....A.....C... : 1200
DQ138133.1|Muju-V|N|South-Korea|E.r.|96-1|         : .........CA..C....A..G...........C..A..G..T..G..G.....TC..C.C...........G.....A..A..G.....A.....C... : 1200
DQ138142.1|Muju-V|N|South-Korea|E.r.|99-28|        : .........CA..C....A..G...........C..A..G..T..G..G.....TC..C.C...........G.....A..A..G.....A.....C... : 1200
```

```
                                                           *      1220         *      1240         *      1260         *      1280         *      1300
2014.00598|PUUV|N|France|H.s.|59|FOURMIES|         : CACCTCGGCGATGATATGGATCCTGAGCTTAGGGGCCTTGCACAGGCGCTTATTGATCAAAAAGTAAAGGAGATCTCCAACCAAGAGCCCTTAAAAATAT : 1300
2014.00233|PUUV|N|France|H.s.|59|FOURMIES|         : .....T.....................................................G.....G........T......................... : 1300
2014.00097|PUUV|N|France|H.s.|02|SAINT-MICHEL|     : .....T.............................................................................................. : 1300
2014.00488|PUUV|N|France|H.s.|08|SIGNY-LE-PETIT|   : .....T.............................................................................................. : 1300
2014.00613|PUUV|N|France|H.s.|59|FOURMIES|         : .....T.............................................................................................. : 1300
2015.00402|PUUV|N|France|H.s.|08|CHARLEVILLE-MEZIE : .....T..T.................A......................................G.....A...........G...........G.... : 1300
2015.00422|PUUV|N|France|H.s.|59|ANOR|             : .................................................................................................... : 1300
2015.00488|PUUV|N|France|H.s.|08|SEDAN|            : .....T....................A......................................G.................G...........G.... : 1300
2015.00498|PUUV|N|France|H.s.|08|REMILLY-AILLICOUR : .....T.............................................................................................. : 1300
2016.00295|PUUV|N|France|H.s.|08|CORNY-MACHEROMENI : .....T.....................................................G.....G........T..T...................... : 1300
2016.00333|PUUV|N|France|H.s.|59|FOURMIES|         : .....T.............................................................................................. : 1300
2016.00345|PUUV|N|France|H.s.|59|FOURMIES|         : .....T.............................................................................................. : 1300
2016.00357|PUUV|N|France|H.s.|02|HIRSON|           : .....T.............................................................................................. : 1300
2016.00427|PUUV|N|France|H.s.|59|FOURMIES|         : .................................................................................................... : 1300
2016.00469|PUUV|N|France|H.s.|02|PREMONTRE|        : ..T..T..T.......................A..............A.................G.....A..A..G...................... : 1300
AJ277075.1|PUUV|N|Belgium|M.g.|Montbliart/CG14444| : .....T....................A....................A.................G.....A...........G...........G.... : 1300
2014.00053|PUUV|N|France|H.s.|08|SECHEVAL|         : .....T....................A......................................G.................G...........G.... : 1300
KT247593.1|PUUV|N|France|M.g.|08|Ardennes/Mg75/201 : .....T....................A....................A.................G.....A...........G........G..G.... : 1300
KT247592.1|PUUV|N|France|M.g.|08|Ardennes/Mg156/20 : .....T....................A..............T.....T.................G.....A...........G........G..G.... : 1300
2015.00019|PUUV|N|France|H.s.|08|ETEIGNIERES|      : .....T.................C..A....................A.................G.....A...........G...........G.... : 1300
2016.00239|PUUV|N|France|H.s.|08|VIREUX-MOLHAIN|   : .....T....................A....................A.................G.....A...........G...........G.... : 1300
2015.00329|PUUV|N|France|H.s.|08|BOGNY-SUR-MEUSE|  : .....T....................A....................A.................G.....A...........G...........G.... : 1300
2014.00209|PUUV|N|France|H.s.|08|TREMBLOIS-LES-ROC : .....T..T.................A....................A.................G.....A...........G...........G.... : 1300
2015.00456|PUUV|N|France|H.s.|94|ALFORTVILLE|      : .....T....................A....................A.................G.....A...........G...........G.... : 1300
2012.00018|PUUV|N|France|H.s.|08|ROCROI|           : .....T..T.................A....................A.................G.....A...........G...........G.... : 1300
AJ277032.1|PUUV|N|Belgium|M.g.|Momignies/47Cg/96|  : .....T....................A.................A..A.................G.....A...........G...........G.... : 1300
AJ277034.1|PUUV|N|Belgium|M.g.|Couvin/59Cg/97|     : .....T....................A....................A.................G.....A...........G...........G.... : 1300
AJ277033.1|PUUV|N|Belgium|M.g.|Momignies/55Cg/96|  : .....TA...................A....................A.................G.....A...........G...........G.... : 1300
2012.00638|PUUV|N|France|H.s.|08|GIVET|            : .....T....................A......................................G.....A.....T.....G...........G.... : 1300
2015.00328|PUUV|N|France|H.s.|08|VRIGNE-MEUSE|     : ..........................A....................A.................G.....A..A........G...........G.... : 1300
2015.00419|PUUV|N|France|H.s.|08|DOUZY|            : ..........................A......................................G.....A...........G...........G.... : 1300
2016.00310|PUUV|N|France|H.s.|08|THIN-LE-MOUTIER|  : .....T.....................................................G.....G........T......................... : 1300
AJ277030.1|PUUV|N|Belgium|M.g.|Thuin/33Cg/96|      : .....T....................A......................................G.....A...........G.....T.....G.... : 1300
AJ238779.1|PUUV|N|Germany|M.g.|NRW/Cg-Erft|        : ..........................A....................A........C........G.....A..A......................... : 1300
2014.00276|PUUV|N|France|H.s.|59|LILLE|            : .....T........C........C..A.......................C..............G..A..A..A....................G.... : 1300
U22423.1|PUUV|N|Belgium|M.g.|CG13891|              : .....T........C...........A..C.................A..C..............G.....A..A..T...................... : 1300
2014.00321|PUUV|N|France|H.s.|59|FOURMIES|         : .....T.........................................A.................................................... : 1300
2015.00457|PUUV|N|France|H.s.|55|REVIGNY-SUR-ORNAI : ..T..T..T.......................A..............A.................G.....A..A..G...................... : 1300
2013.00250|PUUV|N|France|H.s.|62|VENDIN-LS-BTHUNE| : .....T..G.....C...........A....................A.................G........A......................... : 1300
2016.00282|PUUV|N|France|H.s.|59|MORBECQUE|        : .....T..G.....C...........A....................A.................G.....A..A......................... : 1300
AJ277076.1|PUUV|N|Belgium|M.g.|Montbliart/CG14445| : .....T....................A....................A.................G.....A...........G...........G.... : 1300
2014.00153|PUUV|N|France|H.s.|02|BUIRONFOSSE|      : .....T..T.................A....................A.......................A..T..T.....G...........G.... : 1300
2014.00171|PUUV|N|France|H.s.|59|SAINT-SAULVE|     : .....T..T..C..............A....................A.................G.....A..A.....T.....A..T..G....... : 1300
2016.00293|PUUV|N|France|H.s.|60|LA-NEUVILLE-SUR-R : .....T..T.................A.....AA.......C..A..A..C........G.....G.....A..A...........T............. : 1300
2014.00499|PUUV|N|France|H.s.|94|CHAMPIGNY-SUR-MAR : .....T....................A..C...................................G.....A.....T.....G...........G.... : 1300
2012.00057|PUUV|N|France|H.s.|59|COUSOLRE|         : .....T....................A....................A.................G.....A.......................G.... : 1300
2012.00349|PUUV|N|France|H.s.|02|ENGLANCOURT|      : .....T..T.................A....................A.................G.....A..T..T.....G...........G.... : 1300
2014.00135|PUUV|N|France|H.s.|02|ATHIES-SOUS-LAON| : .....T....................A..C...................................G.....A.....T.....G...........G.... : 1300
2014.00184|PUUV|N|France|H.s.|59|FOURMIES|         : ..T..T..T.................A........T...........A.................G.....A...........G...........G.... : 1300
2016.00182|PUUV|N|France|H.s.|08|BOULZICOURT|      : .....T....................A....................A.................G.....A...........G...........G.... : 1300
2016.00286|PUUV|N|France|H.s.|02|TRUCY|            : .....T....................A..C...................................G.....A.....T.....G...........G.... : 1300
2016.00325|PUUV|N|France|H.s.|59|AULNOYE-AYMERIES| : .....T....................A....................A.................G.....A...........G...........G.... : 1300
2016.00452|PUUV|N|France|H.s.|02|LAON|             : .....T....................A..C...................................G.....A.....T.....G...........G.... : 1300
2016.00467|PUUV|N|France|H.s.|02|AISNE|            : ..T..T....................A....................A.................G.....A.....T.....G...........G.... : 1300
```

```
                                                   *      1220         *      1240         *      1260         *      1280         *      1300
2014.00598|PUUV|N|France|H.s.|59|FOURMIES|       : CACCTCGGCGATGATATGGATCCTGAGCTTAGGGGCCTTGCACAGGCGCTTATTGATCAAAAAGTAAAGGAGATCTCCAACCAAGAGCCCTTAAAAATAT : 1300
2015.00657|PUUV|N|France|H.s.|02|CILLY|          : .....T........C...........A.....A..............A.......................A..A..............A.......... : 1300
2015.00526|PUUV|N|France|H.s.|08|MONTHERME|      : .....T....................A......................................G.....A.....T.....G...........G.... : 1300
2015.00045|PUUV|N|France|H.s.|59|FOURMIES|       : .....T..T.................A....................A.................G.....A...........G...........G.... : 1300
2015.00430|PUUV|N|France|H.s.|02|MONTCORNET|     : .....T..T.................A......................................G..A........T.....G...........G.... : 1300
2016.00311|PUUV|N|France|H.s.|02|LAON|           : .....T....................A..C...................................G.....A.....T.....G...........G.... : 1300
AJ277031.1|PUUV|N|Belgium|M.g.|Montbliart/23Cg/96| : .....T....................A....................A.................G.....A...........G...........G.... : 1300
2016.00326|PUUV|N|France|H.s.|02|LAON|           : .....T....................A..C...................................G.....A.....T.....G...........G.... : 1300
2016.00353|PUUV|N|France|H.s.|02|CESSIERES|      : .....T....................A..C...................................G.....A.....T.....G...........G.... : 1300
2012.00061|PUUV|N|France|H.s.|02|LANISCOURT|     : .....T....................A..C...................................G.....A.....T.....G...........G.... : 1300
2014.00174|PUUV|N|France|H.s.|02|ST-ERME-OUTRE-ET- : .....T....................A..C...................................G.....A.....T.....G...........G.... : 1300
2016.00268|PUUV|N|France|H.s.|02|PRESLES-ET-THIERN : .....T....................A..C...................................G.....A.....T.....G...........G.... : 1300
2015.00660|PUUV|N|France|H.s.|02|VIC-SUR-AISNE|  : .....T..T.................A....................A.................G..A..A..A.................G..G.... : 1300
2012.00025|PUUV|N|France|H.s.|51|SAINTE-MENEHOULD| : .....G..T.................A..C.....T........A..A...........G..G..G.....A..A..............T.......... : 1300
2015.00665|PUUV|N|France|H.s.|51|REIMS|          : .....G..T.....C...........A..C..A...........A..A...........G..G..G..A..A..A..A..T........T.......... : 1300
2012.00307|PUUV|N|France|H.s.|54|COLOMBEY-LES-BELL : .....G..T.....C............T.G..............A..A..A..C.....G.....G..A..a..A......................... : 1300
2012.00123|PUUV|N|France|H.s.|55|BAR-LE-DUC|     : ..T..G..T.................A..C..A...........A..A...........G..G..G.....A..A..............TC......... : 1300
2012.00278|PUUV|N|France|H.s.|51|REIMS|          : .....G..T.....C...........A..C..A...........A..A...........G..G..G..A..A..A..A.....G.....T.......... : 1300
2015.00185|PUUV|N|France|H.s.|68|FELLERING|      : .....G..T.....C...........A..C..A...........A..A.....C.....G..G..G.....A..A..............T.......... : 1300
KJ994776.1|PUUV|N|Germany|M.g.|Mu/07/1219|2007|  : ..T..G..T.................A..C..A..T........A..A..C..............G..A..A..A..T..T........T.......... : 1300
2012.00086|PUUV|N|France|H.s|58|CHEVROCHES|      : ..TT.G..T.................A.....A........C..A..A..g........G.....T..A..A..A..A.....G.......C........ : 1300
KT247595.1|PUUV|N|France|M.g.|45|Orleans/Mg29/2010 : ..TT.G..T.................A.....A........C..A..A..G........G.....T..A..A..A..A.....G.......C........ : 1300
KT247594.1|PUUV|N|France|M.g.|45|Orleans/Mg23/2010 : ..TT.G..T.................A.....A........C..A..A..G........G.....T..A..A..A..A.....G.......C........ : 1300
KY365004.1|PUUV|N|France|M.g.|45|Orleans/NCHA373/2 : ..TT.G..T.................A.....A........C..A..A..G........G.....T..A..A..A..A.....G.......C........ : 1300
2012.00301|PUUV|N|France|H.s.|25|MOUTHE|         : ..T..G..T..C........C...........A........C..A..A...........G.....G..A.....T.....T..G..A............. : 1300
AM695638.1|PUUV|N|France|M.g.|Mignovillard/CgY02/2 : ..T..G..T..C........C...........A........C..A..A...........G.....G..A.....T.....T..G..A............. : 1300
KT247597.1|PUUV|N|France|M.g.|39|Jura/Mg214/2010| : ..T..G..T..C....................A........C..A..A...........G.....G..A.....T..T..T..G..A............. : 1300
KT247596.2|PUUV|N|France|M.g.|39|Jura/Mg2/2010|  : ..T..G..T..C.............................C..A..A...........G.....G..A.....T..T..T..G..A............. : 1300
2012.00102|PUUV|N|France|H.s.|39|COISERETTE|     : ..T..G..T..C....................A........C..A..A.................G..A.....T..T..T..G..A............. : 1300
2012.00536|PUUV|N|France|H.s.|39|LA-PESSE|       : ..T..G..T..C....................A........C..A..A.................G..A.....T..T..T..G..A............. : 1300
2014.00622|PUUV|N|France|H.s.|39|ARBOIS|         : ..T..G..T..C....................A........C..A..A...........G........A.....T.....T..G..A........G.... : 1300
2015.00567|PUUV|N|France|H.s.|70|RIOZ|           : ..T..G..T..C....................A........C..A..A...........G.....G..A.....T..T..T..G..A............. : 1300
2014.00637|PUUV|N|France|H.s.|25|SAULES|         : ..T..G..T..C....................A........C..A..A...........G.....G..A.....T..T..T..G..A............. : 1300
2012.00396|PUUV|N|France|H.s.|39|SAINT-CLAUDE|   : ..T..G..T..C....................A........C..AA.A...........G.....G..A...........T..G..A............. : 1300
2014.00120|PUUV|N|France|H.s.|38|LE-MOUTARET|    : ..T..G..T..C....................A........C..AA.A...........G.....G..A.....T.....T..G..A............. : 1300
2015.00153|PUUV|N|France|H.s.|73|GREZY-SUR-ISERE| : ..T..G..T.......................A........C..AA.A...........G.....G..A.....T.....T..G..A............. : 1300
2015.00504|PUUV|N|France|H.s.|70|RONCHAMP|       : ..T..G..T....................C..A........C..A..A..C........G.....G..A........T..T.....A..A.....G.... : 1300
2016.00275|PUUV|N|France|H.s.|21|JALLANGES|      : ..T..G..T.......................A........C..A..A...........G.....G..A.....T.....T..G..A............. : 1300
2016.00320|PUUV|N|France|H.s.|25|SAINT-VIT|      : ..T..G..T..C....................A........C..A..A...........G.....G..A.....T..T..T..G..A............. : 1300
2015.00410|PUUV|N|France|H.s.|70|ANGIREY|        : ..T..G..T....................C..A........C..A..A..C........G.....G..A.....T..T..T.....A........G.... : 1300
DQ016430.2|PUUV|N|Germany|M.g.|Bavaria/CG33/04|  : ..T..T..T.....C........A..A.....A...........A..A....................A..A..A.....T..G........G....... : 1297
DQ016432.2|PUUV|N|Germany|M.g.|Bavaria/CG41/04|  : ..T..G..T.....C........A..A.....A...........A..A........C..............A..A.....T..G................ : 1300
AY954723.2|PUUV|N|Germany|M.g.|Bavaria-CG34/04|  : ..T..G..T.....C........A..A.....A...........A..A........C..............A..A.....T..G................ : 1300
AY954722.2|PUUV|N|Germany|M.g.|Bavaria-CG9/04|   : ..T..G..T.....C.....C..A..A.....A...........A..A........C........G.....A..A..T..T..G........G....... : 1300
2012.00402|PUUV|N|France|H.s.|60|GOUVIEUX|       : ..T..G..T.......................A........C..A..T...........G.....G..A..A..T..T..T................... : 1300
2014.00540|PUUV|N|France|H.s.|60|CHAMBLY|        : ..T..G..T.......................A........C..A..T...........G.....G..A..A..T..T..T.....A............. : 1300
AJ314600.1|PUUV|N|Balkan|M.g.|Balkan-1|          : ..T..T..T.................A..C..A..A...........T........C..G.....G.....A..A..T.....G................ : 1300
AJ314601.1|PUUV|N|Balkan|M.g.|Balkan-2|          : ..T..T..T..C..............A..C..A..A...........T..C.....C..G.....G.....A..A..T.....G...........G.... : 1300
FN377821.1|PUUV|N|Hungary|M.g.|Mg9/HungaryTR17/00| : ..T..T..T.................A..C..A..G...........C..C.....C..G.....G........A..T.....G........G....... : 1300
FN377822.1|PUUV|N|Hungary|M.g.|Mg23/HungaryTR17/00 : ..T..T..T.................A..C..A..A...........T..C.....C..G.....G.....A..A..T.....G................ : 1300
GQ339473.1|PUUV|N|Sweden|M.g.|Kiviniemi/Mg3/05|  : ...T.G..T.....C.................A..T.....C.....A..G...........G..G...........T..T..G..A..A..G....... : 1300
GQ339474.1|PUUV|N|Sweden|M.g.|Kiviniemi/Mg5/05|  : ...T.G..T.....C.................A..T.....C.....A..G...........G..G..............T..G..A..A..G....... : 1300
GQ339475.1|PUUV|N|Sweden|M.g.|Kiviniemi/Mg6/05|  : ...T.G..T.....C.................A..T.....C.....A..G...........G..G..............T..G..A..A..G....... : 1300
GQ339476.1|PUUV|N|Sweden|M.g.|Aijajarvi/Mg7/05|  : ...T.G..T.....C.................A..T.....C.....A..G...........G..G...........T..T..G..A..A..G....... : 1300
```

```
                                                  *      1220         *      1240         *      1260         *      1280         *      1300
2014.00598|PUUV|N|France|H.s.|59|FOURMIES|        : CACCTCGGCGATGATATGGATCCTGAGCTTAGGGGCCTTGCACAGGCGCTTATTGATCAAAAAGTAAAGGAGATCTCCAACCAAGAGCCCTTAAAAATAT : 1300
GQ339477.1|PUUV|N|Sweden|M.g.|Aijajarvi/Mg9/05|   : ...T.G..T.....C.................A..T.....C.....A..G...........G..G...........T..T..G..A..A..G....... : 1300
GQ339478.1|PUUV|N|Sweden|M.g.|Jockfall/Mg12/05|   : ...T.G..T...........C..............T.....C.....A..G...........G..G........T..T..T..G..A..AC.T....... : 1300
GQ339482.1|PUUV|N|Sweden|M.g.|Kalvudden/Mg22/05|  : ..TT.G..T.................A.....A..T.....C.....A..G.....C.....G..G..A.....T..T..T..G..A..AC.T....... : 1300
GQ339479.1|PUUV|N|Sweden|M.g.|Moskosel/Mg17/05|   : .....G..T.......................A..T.....C.....A..G........G..G..G...........T..T..G..A..AC.T....... : 1300
GQ339481.1|PUUV|N|Sweden|M.g.|Ljustrask/Mg20/05|  : ...T.G..T.......................A..T.....C.....AT.G........G..G..G...........T..T..G..A..AC.T....... : 1300
GQ339480.1|PUUV|N|Sweden|M.g.|Gyttjea/Mg19/05|    : ...T.G..T..C....................A..T.....C.....A..G........G..G..G...........T..T..G..A..AC.T....... : 1300
AY526219.1|PUUV|N|Sweden|Human|Umea/hu|           : .....G..T.....C........G..A..C..A..T.....C.....AT.G........G..............A..T..T..G..T..AC.T..G.... : 1300
AJ223380.1|PUUV|N|Sweden|M.g.|Tavelsjo/Cg81/94|   : ..T..G..T.....C..............C..A..T.....C.....A..G........G..G.....A..A..A..T..T..G..T..AC.T..G.... : 1300
U14137.1|PUUV|N|Bosnia-H.|M.g.|Vranica|           : ..T..G..T.....C..............C..A..T.....C.....A..G........G..G.....A..A..A..T..T..G..T..AC.T..G.... : 1300
AJ223371.1|PUUV|N|Sweden|M.g.|Huggberget/Cg36/94| : ..T..G..T....................A..A..T.....C.....A..G........G..G........A..A..T..T..G..T..AC.T..G.... : 1300
Z48586.1|PUUV|N|Sweden|M.g.|Vindeln/L20Cg/83|     : ..T..G..T.....C..............C..A..T.....C.....A..G........G..G.....A..A..A..T..T..G..T..GC.T..G.... : 1300
AJ223374.1|PUUV|N|Sweden|M.g.|Mellansel/Cg47/94|  : ..T..G..T.....C..............C..A..T.....C.....AT.G........G..............A..T..T..G..T..AC.T..G.... : 1300
AJ223375.1|PUUV|N|Sweden|M.g.|Mellansel/Cg49/94|  : ..T..G..T.....C..............C..A..T.....C.....AT.G........GG.............A..T..T..G..T..AC.T..G.... : 1300
AJ238791.1|PUUV|N|Denmark|M.g.|Fyn/19|            : .....A..T.....C.....C.........C.A..T...........A..A........G.....C.....A..A..T..T.....A..T..G....... : 1300
AJ278092.1|PUUV|N|Denmark|M.g.|Fyn47|             : ..T..A..T...........C.....A..CC.A..............AT.A........G.....C..A..A..A..T..T..G..A..T..G..G.... : 1300
AJ278093.1|PUUV|N|Denmark|M.g.|Fyn131|            : ..T..A..T...........C.....A..CC.A..............AT.A...A....G.....C..A..A..A..T..T..G..A..T..G..G.... : 1300
AJ223368.1|PUUV|N|Norway|M.g.|Eidsvoll/1124v|     : ........T.....C........A.....C..A..T..G........TT.A..............G..A.....A..T..T..G.....TC.......T. : 1300
AJ223369.1|PUUV|N|Norway|M.g.|Eidsvoll/Cg1138/87| : ........T.....C........A.....C..A..T..G........TT.A..............G..A.....A..T..T..G.....TC.......T. : 1300
JN657228.1|PUUV|N|Latvia|M.g.|Jelgava/Mg149/2008| : ..T..T..T.....C............T.G..A..T........A..A..G..A...........G........A..A..T..G..A........G.... : 1300
KX757839.1|PUUV|N|Lithuania|M.g.|LT15/164|2015|   : ..T..T..T.....C........A.....A..A..T...........A..G..A..C..G.....G..A..A..A..A..T..............G.... : 1300
KX757840.1|PUUV|N|Lithuania|M.g.|LT15/174|2015|   : ..T..T..T.....C........A.....A..A..T...........AT.G..A..C..G.....G..A..A..A..A..T..............G.... : 1300
KX815394.1|PUUV|N|Poland|M.g.|KS13/855|2009|      : ..T..T..T.....C........A.....G..A..T........A..A..A..A..C..G.....G.....A..T..A.................G.... : 1300
KX757841.1|PUUV|N|Lithuania|M.g.|LT15/201|2015|   : ..T..T..T.....C........A.....A..A..T...........A..G..A..C..G.....G..A..A..A..A..T..............G.... : 1300
AJ314598.1|PUUV|N|Russia|M.g.|Baltic/49Cg/00|     : ..T..T..T.....C...........A..C.....T.....C.....AT.G...........G...........A..A.................G.... : 1300
AJ314599.1|PUUV|N|Russia|M.g.|Baltic/205Cg/00|    : ..T..T..T.....C...........A........T..A..T.....AT.G........G..G.....A..A..A..A..T..............G.... : 1300
JN657229.1|PUUV|N|Latvia|M.g.|Madona/Mg99/2008|   : ..T..T..T.................A........T.....T.....AT.G........G........A.....A..A..T.....A............. : 1300
JN657232.1|PUUV|N|Latvia|M.g.|Madona/Mg233/2008|  : ..T..T..T.................A........T.....T.....AT.G........G........A.....A..A..T................... : 1300
JN657230.1|PUUV|N|Latvia|M.g.|Jelgava/Mg136/2008| : .....T..T.................A..C.....T.....C.....AT.G........G.....G..A.....A..A..T..............G.... : 1300
JN657231.1|PUUV|N|Latvia|M.g.|Jelgava/Mg140/2008| : .....T..T.................A..C.....T.....C.....AT.G........G.....G..A.....A..A..T..............G.... : 1300
Z30707.1|PUUV|N|Russia|M.g.|Udmurtia/458Cg/88|    : ..T..T..T....................A..A........T...T.A..C..A..C..G..G.....A.....A..A..T..G...........G.... : 1300
Z30706.1|PUUV|N|Russia|M.g.|Udmurtia/444Cg/88|    : ..T..T..T....................A..A........T...T.A..C..A..C..G..G.....A.....A..A..T..G...........G.... : 1300
Z84204.1|PUUV|N|Russia|M.g.|Kazan|                : ..T..T..T....................A..A........T...T.A..C..A..C..G..G.....A.....A..A..T..G...........G.... : 1300
Z30708.1|PUUV|N|Russia|M.g.|Udmurtia/338Cg/92|    : ..T..T..T....................A..A........T...T.A..C..A..C..G..G.....A.....A..A..T..G...........G.... : 1300
Z21497.1|PUUV|N|Russia|M.g.|Udmurtia/894Cg/91|    : ..T..T..T....................A..A........T...T.A..C..A..C..G..G.....A.....A..A.................G.... : 1300
AB433843.2|PUUV|N|Russia|M.g.|Samara_49/CG/2005|  : ..T..T..T....................A...........C...T.A..C..C.....G..G.....A.....A..A.................G.... : 1300
AB433845.2|PUUV|N|Russia|M.g.|Samara_94/CG/2005|  : ..T..T..T..................T.A...........C...T.A..C..C.....G..G.....A.....A..A.....G...........G.... : 1300
L11347.1|PUUV|N|Russia|Human|P360|                : ..T..T..T.....C..............A...........T...T.A..C..A..C..G..G.....A.....A..A........A........G.... : 1300
AB297665.2|PUUV|N|Russia|M.g.|DTK/Ufa-97|1997|    : ..T..T..T.....C..............A...........T...T.A..C..A..C..G..G.....A.....A..A........A........G.... : 1300
M32750.1|PUUV|N|Russia|M.g.|CG1820|               : ..T..T..T.....C..............A...........T...T.A..C..A..C..G..G.....A.....A..A........A........G.... : 1300
AF442613.1|PUUV|N|Russia|M.g.|CG17/Baskiria-2001| : ..T..T..T.....C..............A...........T...T.A..C..A..C..G..G.....A.....A..A........A........G.... : 1300
KX815395.1|PUUV|N|Poland|M.g.|KS14/118|2009|      : ..T..T..T.....C..............C.....T.....T.....T...........G..G..T..A.....A..A..T........T.......... : 1300
GQ339483.1|PUUV|N|Sweden|M.g.|Bergsjobo/Mg25/05|  : ..T..A..A...........C..A.....G..A...T.A..C..A..A..G..A..C..G..G.....A..A..A..T.....G.....T..G..G..T. : 1300
AJ223377.1|PUUV|N|Sweden|M.g.|Solleftea/Cg6/95|   : ..T..A..A...........C..A.....G..A...T.A.....A..A..G..A..C..G..G.....A..A..A..T.....G.....T..G..G..T. : 1300
GQ339484.1|PUUV|N|Sweden|M.g.|Faboviken/Mg26/05|  : ..T..A..A...........C..A..A..G..A...T.G..C..A..A..G.....C..G..G.....A..A..A........G.....T.....G..T. : 1300
GQ339485.1|PUUV|N|Sweden|M.g.|Mangelbo/Mg1/05|    : ..T..A..A.....C........A..A..G..A..TT.GA.C..A..A..A..C..C.....G........A..A..T....C......T.....G..T. : 1300
GQ339486.1|PUUV|N|Sweden|M.g.|Munga/Mg2/05|       : ..T..A..A.....C........A..A..G..A..TT.G..C..A..A..A..C..C.....G...........A..T.................G..T. : 1300
GQ339487.1|PUUV|N|Sweden|M.g.|Munga/Mg16/05|      : ..T..A..A.....C........A..A..G..A..TT.G..C..A..A..A..C..C.....G...........A..T.................G..T. : 1300
AJ223376.1|PUUV|N|Sweden|M.g.|Solleftea/Cg3/95|   : .....A..A...........C..A..A..G..A...T.A.....A..A..G.....C..G..G.....A..A..A.....T..G..A..T.....G..T. : 1300
AF367071.1|PUUV|N|Russia|M.r.|CRF366|             : ..T..T..T.................AT.G.....G..A........A..G..A...........G..A.....A..A...........A.....G.... : 1300
AF367064.1|PUUV|N|Russia|M.g.|CG144|              : ..T..T..T.................AT.G.....G..A........A..G..A...........G..A.....A..A...........A.....G.... : 1300
AF367065.1|PUUV|N|Russia|M.g.|CG168|              : ..T..T..T.................A..G.....G..A........A..G..A...........G..A.....A..A...........A.....G.... : 1300
AF367068.1|PUUV|N|Russia|M.g.|CG315|              : ..T..T..T.................A..A.....G..A........A..G..A...........G..A.....A..A...........A.....G.... : 1300
AF367069.1|PUUV|N|Russia|M.r.|CRF161|             : ..T..T..T.................A..G.....G..A........A..G..A...........G..A.....A..A...........A.....G.... : 1300
```

```
                                                            *      1220         *      1240         *      1260         *      1280         *      1300
2014.00598|PUUV|N|France|H.s.|59|FOURMIES|         : CACCTCGGCGATGATATGGATCCTGAGCTTAGGGGCCTTGCACAGGCGCTTATTGATCAAAAAGTAAAGGAGATCTCCAACCAAGAGCCCTTAAAAATAT : 1300
AF367070.1|PUUV|N|Russia|M.r.|CRF308|              : ..T..T..T.................A..A.....G..A........A..G..A...........G..A.....A..A...........A.....G.... : 1300
AF367066.1|PUUV|N|Russia|M.g.|CG215|               : ..T..T..T.................A..G.....G..A......A.A..G..A...........G..A.....A..A...........A.....G.... : 1300
AF367067.1|PUUV|N|Russia|M.g.|CG222|               : ..T..T..T.................A..G.....G..A......A.A..G..A...........G..A.....A..A...........A.....G.... : 1300
Z46942.1|PUUV|N|Finland|M.g.|Puumala/1324Cg/79|    : ..T.....T..............A........A..T...........A..G..A........G..G..A..A..A..A.....G.....A.......... : 1300
Z30702.1|PUUV|N|Finland|M.g.|Evo/12Cg/93|          : ..T..A..T..............A..A..C..A..T...........A..G...........G..G.....A..A..A...........A.......... : 1300
Z30703.1|PUUV|N|Finland|M.g.|Evo/13Cg/93|          : ..T..A..T..............A..A..C..A..T...........A..G...........G..G.....A..A..A...........A.......... : 1300
Z30704.1|PUUV|N|Finland|M.g.|Evo/14Cg/93|          : ..T..A..T..............A..A..C..A..T...........A..G...........G..G.....A..A..A...........A.....G.... : 1300
Z30705.1|PUUV|N|Finland|M.g.|Evo/15Cg/93|          : ..T..A..T..............A..A..C..A..T...........T..G...........G..G.....A..A..A...........A.......... : 1300
Z69985.1|PUUV|N|Finland|M.g.|Virrat/25Cg/95|       : .....T..T..............A........A..............A..G..A........G..G.....A..A..A..T..G.....A.....G.... : 1300
JN831947.1|PUUV|N|Finland|Pieksamaki/human_lung/20 : ..T..T..T.....C........A........A..T...........A..G..A..C.....G..G..A..A..A..A...........A.......... : 1300
JN831943.1|PUUV|N|Finland|Pieksamaki/Mg7/2008|     : ..T..T..T.....C........A........A..T...........A..G..A..C.....G..G..A..A..A..A...........A.......... : 1300
JN831950.1|PUUV|N|Finland|Pieksamaki/human_kidney/ : ..T..T..T.....C........A........A..T...........A..G..A..C.....G..G..A..A..A..A...........A.......... : 1300
JQ319166.1|PUUV|N|Finland|Konnevesi/Mg_014B/2005|  : ..T.....T.....C........A.....C..A..T........A..A..G..A........G..G.....A..A..A.....G.....A..G....... : 1300
JQ319169.1|PUUV|N|Finland|Konnevesi/Mg_027B/2005|  : ..T.....T.....C........A.....C..A..T........A..A..G..A........G..G.....A..A..A.....G.....A..G....... : 1300
JQ319167.1|PUUV|N|Finland|Konnevesi/Mg_015B/2005|  : ..T..T..T..............A.....A..A..T..............G..A........G..G.....A..A..A...........G..G....... : 1300
JQ319164.1|PUUV|N|Finland|Konnevesi/Mg_06B/2005|   : ..T.....T.....C........A.....C..A..T........A..A..G..A........G..G.....A..A..A.....G.....A..G....... : 1300
JQ319165.1|PUUV|N|Finland|Konnevesi/Mg_09B/2005|   : ..T.....T.....C........A.....C..A..T........A..A..G..A........G..G.....A..A..A.....G.....A..G....... : 1300
JQ319170.1|PUUV|N|Finland|Konnevesi/Mg_074B/2005|  : ..T.....T.....C........A.....C..A..T........A..A..G..A........G..G.....A..A..A.....G.....A..G....... : 1300
JQ319171.1|PUUV|N|Finland|Konnevesi/Mg_M114B/2005| : ..TT.T..T..............A.....A..A..T..............G..A........G..G.....A..A..A...........G..G....... : 1300
AJ238788.1|PUUV|N|Russia|M.g.|Karhumaki|           : ..T..T..T..............A.....C..A..T........A..A..G..A..C.....G..G..A..A..A..A.....G.....A.....G.... : 1300
AJ238789.1|PUUV|N|Russia|M.g.|Kolodozero|          : ..T..T..T...........C..A..A..C.....T...........A..G..A.....G..G........A..A..A.....G.....A..G....... : 1300
AJ314597.1|PUUV|N|Finland|M.g.|Pallasjarvi/63Cg/98 : ..T..T..T.....C.....C..A.....C..A..T..G........AT.G..A........G..G........A..A........A..T.......... : 1300
NC_005224.1|PUUV|N|Finland|M.g.|Sotkamo-revu-NCBI2 : ..T..T..T..............A.....C..A..T...........A..G..A........G..C.....A..A..G.....G..A..AC....G.... : 1300
AJ238790.1|PUUV|N|Russia|M.g.|Gomselga|            : ..T..T..T..............A.....C..A..T...........A..G..A........G..G.....A..A..A...........A.......... : 1300
AB010731.1|Hokkaido-V|N|Japan|M.r.|Tobetsu-60Cr-93 : ..T.....G.......................A..G..G..G.....AT.G........G...........A..T..G.....G.....AC....G.... : 1300
AB010730.1|Hokkaido-V|N|Japan|M.r.|Kamiiso-8Cr-95| : ..T..T..G.......................A..G..G........AT.G........G...........A..T..G.....G..A..AC....G.... : 1300
JX046487.1|Muju-V|N|South-Korea|M.r.|11-5|2011     : .....G..T.....C..............GC..AC.T.G........TT.G..C..C..G..G..G.....A..A..T........A...C.T.....T. : 1300
JX046484.1|Muju-V|N|South-Korea|M.r.|11-4|2011|    : .....G..T.....C..............GC..AC.T.G........TT.G..C..C..G..G........A..A..T........A...C.T.....T. : 1300
JX028273.1|Muju-V|N|South-Korea|M.r.|11-1|2011|    : ...T.G..T.....C..............AC..AC.T.G........TT.G..C..C..G.....G.....A..A..T........A...C.T.....T. : 1300
DQ138128.1|Muju-V|N|South-Korea|E.r.|00-18|        : .....G..G....................G..AAC.T.A........AT.G.....C.....G..G..A.....A.....T..G..A...C.T.....T. : 1300
DQ138140.1|Muju-V|N|South-Korea|E.r.|99-27|        : .....G..G....................G..AAC.T.A........AT.G.....C.....G..G..A.....A.....T..G..A...C.T.....T. : 1300
DQ138133.1|Muju-V|N|South-Korea|E.r.|96-1|         : .....G..G....................G..AAC.T.A........AT.G.....C.....G..G..A.....A.....T..G..A...C.T.....T. : 1300
DQ138142.1|Muju-V|N|South-Korea|E.r.|99-28|        : .....G..G....................G..AAC.T.A........AT.G.....C.....G..G..A.....A.....T..G..A...C.T.....T. : 1300
```

```
2014.00598|PUUV|N|France|H.s.|59|FOURMIES|         : GA : 1302
2014.00233|PUUV|N|France|H.s.|59|FOURMIES|         : .. : 1302
2014.00097|PUUV|N|France|H.s.|02|SAINT-MICHEL|     : .. : 1302
2014.00488|PUUV|N|France|H.s.|08|SIGNY-LE-PETIT|   : .. : 1302
2014.00613|PUUV|N|France|H.s.|59|FOURMIES|         : .. : 1302
2015.00402|PUUV|N|France|H.s.|08|CHARLEVILLE-MEZIE : .. : 1302
2015.00422|PUUV|N|France|H.s.|59|ANOR|             : .. : 1302
2015.00488|PUUV|N|France|H.s.|08|SEDAN|            : .. : 1302
2015.00498|PUUV|N|France|H.s.|08|REMILLY-AILLICOUR : .. : 1302
2016.00295|PUUV|N|France|H.s.|08|CORNY-MACHEROMENI : .. : 1302
2016.00333|PUUV|N|France|H.s.|59|FOURMIES|         : .. : 1302
2016.00345|PUUV|N|France|H.s.|59|FOURMIES|         : .. : 1302
2016.00357|PUUV|N|France|H.s.|02|HIRSON|           : .. : 1302
2016.00427|PUUV|N|France|H.s.|59|FOURMIES|         : .. : 1302
2016.00469|PUUV|N|France|H.s.|02|PREMONTRE|        : .. : 1302
AJ277075.1|PUUV|N|Belgium|M.g.|Montbliart/CG14444| : .. : 1302
2014.00053|PUUV|N|France|H.s.|08|SECHEVAL|         : .. : 1302
KT247593.1|PUUV|N|France|M.g.|08|Ardennes/Mg75/201 : .. : 1302
KT247592.1|PUUV|N|France|M.g.|08|Ardennes/Mg156/20 : .. : 1302
2015.00019|PUUV|N|France|H.s.|08|ETEIGNIERES|      : .. : 1302
2016.00239|PUUV|N|France|H.s.|08|VIREUX-MOLHAIN|   : .. : 1302
2015.00329|PUUV|N|France|H.s.|08|BOGNY-SUR-MEUSE|  : .. : 1302
2014.00209|PUUV|N|France|H.s.|08|TREMBLOIS-LES-ROC : A. : 1302
2015.00456|PUUV|N|France|H.s.|94|ALFORTVILLE|      : .. : 1302
2012.00018|PUUV|N|France|H.s.|08|ROCROI|           : A. : 1302
AJ277032.1|PUUV|N|Belgium|M.g.|Momignies/47Cg/96|  : .. : 1302
AJ277034.1|PUUV|N|Belgium|M.g.|Couvin/59Cg/97|     : .. : 1302
AJ277033.1|PUUV|N|Belgium|M.g.|Momignies/55Cg/96|  : .. : 1302
2012.00638|PUUV|N|France|H.s.|08|GIVET|            : .. : 1302
2015.00328|PUUV|N|France|H.s.|08|VRIGNE-MEUSE|     : .. : 1302
2015.00419|PUUV|N|France|H.s.|08|DOUZY|            : .. : 1302
2016.00310|PUUV|N|France|H.s.|08|THIN-LE-MOUTIER|  : .. : 1302
AJ277030.1|PUUV|N|Belgium|M.g.|Thuin/33Cg/96|      : .. : 1302
AJ238779.1|PUUV|N|Germany|M.g.|NRW/Cg-Erft|        : .. : 1302
2014.00276|PUUV|N|France|H.s.|59|LILLE|            : .. : 1302
U22423.1|PUUV|N|Belgium|M.g.|CG13891|              : A. : 1302
2014.00321|PUUV|N|France|H.s.|59|FOURMIES|         : .. : 1302
2015.00457|PUUV|N|France|H.s.|55|REVIGNY-SUR-ORNAI : .. : 1302
2013.00250|PUUV|N|France|H.s.|62|VENDIN-LS-BTHUNE| : A. : 1302
2016.00282|PUUV|N|France|H.s.|59|MORBECQUE|        : .. : 1302
AJ277076.1|PUUV|N|Belgium|M.g.|Montbliart/CG14445| : .. : 1302
2014.00153|PUUV|N|France|H.s.|02|BUIRONFOSSE|      : .. : 1302
2014.00171|PUUV|N|France|H.s.|59|SAINT-SAULVE|     : .. : 1302
2016.00293|PUUV|N|France|H.s.|60|LA-NEUVILLE-SUR-R : .. : 1302
2014.00499|PUUV|N|France|H.s.|94|CHAMPIGNY-SUR-MAR : .. : 1302
2012.00057|PUUV|N|France|H.s.|59|COUSOLRE|         : .. : 1302
2012.00349|PUUV|N|France|H.s.|02|ENGLANCOURT|      : .. : 1302
2014.00135|PUUV|N|France|H.s.|02|ATHIES-SOUS-LAON| : .. : 1302
2014.00184|PUUV|N|France|H.s.|59|FOURMIES|         : .. : 1302
2016.00182|PUUV|N|France|H.s.|08|BOULZICOURT|      : .. : 1302
2016.00286|PUUV|N|France|H.s.|02|TRUCY|            : .. : 1302
2016.00325|PUUV|N|France|H.s.|59|AULNOYE-AYMERIES| : .. : 1302
2016.00452|PUUV|N|France|H.s.|02|LAON|             : .. : 1302
2016.00467|PUUV|N|France|H.s.|02|AISNE|            : .. : 1302
```

```
2014.00598|PUUV|N|France|H.s.|59|FOURMIES|         : GA : 1302
2015.00657|PUUV|N|France|H.s.|02|CILLY|            : A. : 1302
2015.00526|PUUV|N|France|H.s.|08|MONTHERME|        : A. : 1302
2015.00045|PUUV|N|France|H.s.|59|FOURMIES|         : .. : 1302
2015.00430|PUUV|N|France|H.s.|02|MONTCORNET|       : .. : 1302
2016.00311|PUUV|N|France|H.s.|02|LAON|             : .. : 1302
AJ277031.1|PUUV|N|Belgium|M.g.|Montbliart/23Cg/96| : .. : 1302
2016.00326|PUUV|N|France|H.s.|02|LAON|             : .. : 1302
2016.00353|PUUV|N|France|H.s.|02|CESSIERES|        : .. : 1302
2012.00061|PUUV|N|France|H.s.|02|LANISCOURT|       : .. : 1302
2014.00174|PUUV|N|France|H.s.|02|ST-ERME-OUTRE-ET- : .. : 1302
2016.00268|PUUV|N|France|H.s.|02|PRESLES-ET-THIERN : .. : 1302
2015.00660|PUUV|N|France|H.s.|02|VIC-SUR-AISNE|    : .. : 1302
2012.00025|PUUV|N|France|H.s.|51|SAINTE-MENEHOULD| : .. : 1302
2015.00665|PUUV|N|France|H.s.|51|REIMS|            : .. : 1302
2012.00307|PUUV|N|France|H.s.|54|COLOMBEY-LES-BELL : .. : 1302
2012.00123|PUUV|N|France|H.s.|55|BAR-LE-DUC|       : .. : 1302
2012.00278|PUUV|N|France|H.s.|51|REIMS|            : .. : 1302
2015.00185|PUUV|N|France|H.s.|68|FELLERING|        : .. : 1302
KJ994776.1|PUUV|N|Germany|M.g.|Mu/07/1219|2007|    : .. : 1302
2012.00086|PUUV|N|France|H.s|58|CHEVROCHES|        : .. : 1302
KT247595.1|PUUV|N|France|M.g.|45|Orleans/Mg29/2010 : .. : 1302
KT247594.1|PUUV|N|France|M.g.|45|Orleans/Mg23/2010 : .. : 1302
KY365004.1|PUUV|N|France|M.g.|45|Orleans/NCHA373/2 : .. : 1302
2012.00301|PUUV|N|France|H.s.|25|MOUTHE|           : .. : 1302
AM695638.1|PUUV|N|France|M.g.|Mignovillard/CgY02/2 : .. : 1302
KT247597.1|PUUV|N|France|M.g.|39|Jura/Mg214/2010|  : .. : 1302
KT247596.2|PUUV|N|France|M.g.|39|Jura/Mg2/2010|    : .. : 1302
2012.00102|PUUV|N|France|H.s.|39|COISERETTE|       : A. : 1302
2012.00536|PUUV|N|France|H.s.|39|LA-PESSE|         : A. : 1302
2014.00622|PUUV|N|France|H.s.|39|ARBOIS|           : .. : 1302
2015.00567|PUUV|N|France|H.s.|70|RIOZ|             : .. : 1302
2014.00637|PUUV|N|France|H.s.|25|SAULES|           : .. : 1302
2012.00396|PUUV|N|France|H.s.|39|SAINT-CLAUDE|     : .. : 1302
2014.00120|PUUV|N|France|H.s.|38|LE-MOUTARET|      : .. : 1302
2015.00153|PUUV|N|France|H.s.|73|GREZY-SUR-ISERE|  : .. : 1302
2015.00504|PUUV|N|France|H.s.|70|RONCHAMP|         : .. : 1302
2016.00275|PUUV|N|France|H.s.|21|JALLANGES|        : .. : 1302
2016.00320|PUUV|N|France|H.s.|25|SAINT-VIT|        : .. : 1302
2015.00410|PUUV|N|France|H.s.|70|ANGIREY|          : .. : 1302
DQ016430.2|PUUV|N|Germany|M.g.|Bavaria/CG33/04|    : .. : 1299
DQ016432.2|PUUV|N|Germany|M.g.|Bavaria/CG41/04|    : .. : 1302
AY954723.2|PUUV|N|Germany|M.g.|Bavaria-CG34/04|    : .. : 1302
AY954722.2|PUUV|N|Germany|M.g.|Bavaria-CG9/04|     : .. : 1302
2012.00402|PUUV|N|France|H.s.|60|GOUVIEUX|         : .. : 1302
2014.00540|PUUV|N|France|H.s.|60|CHAMBLY|          : A. : 1302
AJ314600.1|PUUV|N|Balkan|M.g.|Balkan-1|            : .. : 1302
AJ314601.1|PUUV|N|Balkan|M.g.|Balkan-2|            : .. : 1302
FN377821.1|PUUV|N|Hungary|M.g.|Mg9/HungaryTR17/00| : .. : 1302
FN377822.1|PUUV|N|Hungary|M.g.|Mg23/HungaryTR17/00 : .. : 1302
GQ339473.1|PUUV|N|Sweden|M.g.|Kiviniemi/Mg3/05|    : AG : 1302
GQ339474.1|PUUV|N|Sweden|M.g.|Kiviniemi/Mg5/05|    : A. : 1302
GQ339475.1|PUUV|N|Sweden|M.g.|Kiviniemi/Mg6/05|    : A. : 1302
GQ339476.1|PUUV|N|Sweden|M.g.|Aijajarvi/Mg7/05|    : AG : 1302
```

```
2014.00598|PUUV|N|France|H.s.|59|FOURMIES|         : GA : 1302
GQ339477.1|PUUV|N|Sweden|M.g.|Aijajarvi/Mg9/05|     : AG : 1302
GQ339478.1|PUUV|N|Sweden|M.g.|Jockfall/Mg12/05|     : AG : 1302
GQ339482.1|PUUV|N|Sweden|M.g.|Kalvudden/Mg22/05|    : AG : 1302
GQ339479.1|PUUV|N|Sweden|M.g.|Moskosel/Mg17/05|     : AG : 1302
GQ339481.1|PUUV|N|Sweden|M.g.|Ljustrask/Mg20/05|    : AG : 1302
GQ339480.1|PUUV|N|Sweden|M.g.|Gyttjea/Mg19/05|      : AG : 1302
AY526219.1|PUUV|N|Sweden|Human|Umea/hu|             : AG : 1302
AJ223380.1|PUUV|N|Sweden|M.g.|Tavelsjo/Cg81/94|     : AG : 1302
U14137.1|PUUV|N|Bosnia-H.|M.g.|Vranica|             : AG : 1302
AJ223371.1|PUUV|N|Sweden|M.g.|Huggberget/Cg36/94|   : AG : 1302
Z48586.1|PUUV|N|Sweden|M.g.|Vindeln/L20Cg/83|       : AG : 1302
AJ223374.1|PUUV|N|Sweden|M.g.|Mellansel/Cg47/94|    : AG : 1302
AJ223375.1|PUUV|N|Sweden|M.g.|Mellansel/Cg49/94|    : AG : 1302
AJ238791.1|PUUV|N|Denmark|M.g.|Fyn/19|              : .. : 1302
AJ278092.1|PUUV|N|Denmark|M.g.|Fyn47|               : .. : 1302
AJ278093.1|PUUV|N|Denmark|M.g.|Fyn131|              : .. : 1302
AJ223368.1|PUUV|N|Norway|M.g.|Eidsvoll/1124v|       : .. : 1302
AJ223369.1|PUUV|N|Norway|M.g.|Eidsvoll/Cg1138/87|   : A. : 1302
JN657228.1|PUUV|N|Latvia|M.g.|Jelgava/Mg149/2008|   : A. : 1302
KX757839.1|PUUV|N|Lithuania|M.g.|LT15/164|2015|     : .. : 1302
KX757840.1|PUUV|N|Lithuania|M.g.|LT15/174|2015|     : .. : 1302
KX815394.1|PUUV|N|Poland|M.g.|KS13/855|2009|        : .. : 1302
KX757841.1|PUUV|N|Lithuania|M.g.|LT15/201|2015|     : .. : 1302
AJ314598.1|PUUV|N|Russia|M.g.|Baltic/49Cg/00|       : .. : 1302
AJ314599.1|PUUV|N|Russia|M.g.|Baltic/205Cg/00|      : .. : 1302
JN657229.1|PUUV|N|Latvia|M.g.|Madona/Mg99/2008|     : A. : 1302
JN657232.1|PUUV|N|Latvia|M.g.|Madona/Mg233/2008|    : A. : 1302
JN657230.1|PUUV|N|Latvia|M.g.|Jelgava/Mg136/2008|   : .. : 1302
JN657231.1|PUUV|N|Latvia|M.g.|Jelgava/Mg140/2008|   : .. : 1302
Z30707.1|PUUV|N|Russia|M.g.|Udmurtia/458Cg/88|      : .. : 1302
Z30706.1|PUUV|N|Russia|M.g.|Udmurtia/444Cg/88|      : .. : 1302
Z84204.1|PUUV|N|Russia|M.g.|Kazan|                  : .. : 1302
Z30708.1|PUUV|N|Russia|M.g.|Udmurtia/338Cg/92|      : .. : 1302
Z21497.1|PUUV|N|Russia|M.g.|Udmurtia/894Cg/91|      : .. : 1302
AB433843.2|PUUV|N|Russia|M.g.|Samara_49/CG/2005|    : .. : 1302
AB433845.2|PUUV|N|Russia|M.g.|Samara_94/CG/2005|    : .. : 1302
L11347.1|PUUV|N|Russia|Human|P360|                  : .. : 1302
AB297665.2|PUUV|N|Russia|M.g.|DTK/Ufa-97|1997|      : .. : 1302
M32750.1|PUUV|N|Russia|M.g.|CG1820|                 : .. : 1302
AF442613.1|PUUV|N|Russia|M.g.|CG17/Baskiria-2001|   : .. : 1302
KX815395.1|PUUV|N|Poland|M.g.|KS14/118|2009|        : A. : 1302
GQ339483.1|PUUV|N|Sweden|M.g.|Bergsjobo/Mg25/05|    : AG : 1302
AJ223377.1|PUUV|N|Sweden|M.g.|Solleftea/Cg6/95|     : AG : 1302
GQ339484.1|PUUV|N|Sweden|M.g.|Faboviken/Mg26/05|    : A. : 1302
GQ339485.1|PUUV|N|Sweden|M.g.|Mangelbo/Mg1/05|      : .. : 1302
GQ339486.1|PUUV|N|Sweden|M.g.|Munga/Mg2/05|         : .. : 1302
GQ339487.1|PUUV|N|Sweden|M.g.|Munga/Mg16/05|        : .. : 1302
AJ223376.1|PUUV|N|Sweden|M.g.|Solleftea/Cg3/95|     : A. : 1302
AF367071.1|PUUV|N|Russia|M.r.|CRF366|               : A. : 1302
AF367064.1|PUUV|N|Russia|M.g.|CG144|                : A. : 1302
AF367065.1|PUUV|N|Russia|M.g.|CG168|                : A. : 1302
AF367068.1|PUUV|N|Russia|M.g.|CG315|                : A. : 1302
AF367069.1|PUUV|N|Russia|M.r.|CRF161|               : A. : 1302
```

```
2014.00598|PUUV|N|France|H.s.|59|FOURMIES|         : GA : 1302
AF367070.1|PUUV|N|Russia|M.r.|CRF308|              : A. : 1302
AF367066.1|PUUV|N|Russia|M.g.|CG215|               : A. : 1302
AF367067.1|PUUV|N|Russia|M.g.|CG222|               : A. : 1302
Z46942.1|PUUV|N|Finland|M.g.|Puumala/1324Cg/79|    : A. : 1302
Z30702.1|PUUV|N|Finland|M.g.|Evo/12Cg/93|          : A. : 1302
Z30703.1|PUUV|N|Finland|M.g.|Evo/13Cg/93|          : A. : 1302
Z30704.1|PUUV|N|Finland|M.g.|Evo/14Cg/93|          : A. : 1302
Z30705.1|PUUV|N|Finland|M.g.|Evo/15Cg/93|          : A. : 1302
Z69985.1|PUUV|N|Finland|M.g.|Virrat/25Cg/95|       : A. : 1302
JN831947.1|PUUV|N|Finland|Pieksamaki/human_lung/20 : A. : 1302
JN831943.1|PUUV|N|Finland|Pieksamaki/Mg7/2008|     : A. : 1302
JN831950.1|PUUV|N|Finland|Pieksamaki/human_kidney/ : A. : 1302
JQ319166.1|PUUV|N|Finland|Konnevesi/Mg_O14B/2005|  : A. : 1302
JQ319169.1|PUUV|N|Finland|Konnevesi/Mg_O27B/2005|  : A. : 1302
JQ319167.1|PUUV|N|Finland|Konnevesi/Mg_O15B/2005|  : A. : 1302
JQ319164.1|PUUV|N|Finland|Konnevesi/Mg_O6B/2005|   : A. : 1302
JQ319165.1|PUUV|N|Finland|Konnevesi/Mg_O9B/2005|   : A. : 1302
JQ319170.1|PUUV|N|Finland|Konnevesi/Mg_O74B/2005|  : A. : 1302
JQ319171.1|PUUV|N|Finland|Konnevesi/Mg_M114B/2005| : A. : 1302
AJ238788.1|PUUV|N|Russia|M.g.|Karhumaki|           : A. : 1302
AJ238789.1|PUUV|N|Russia|M.g.|Kolodozero|          : A. : 1302
AJ314597.1|PUUV|N|Finland|M.g.|Pallasjarvi/63Cg/98 : A. : 1302
NC_005224.1|PUUV|N|Finland|M.g.|Sotkamo-revu-NCBI2 : A. : 1302
AJ238790.1|PUUV|N|Russia|M.g.|Gomselga|            : A. : 1302
AB010731.1|Hokkaido-V|N|Japan|M.r.|Tobetsu-60Cr-93 : .. : 1302
AB010730.1|Hokkaido-V|N|Japan|M.r.|Kamiiso-8Cr-95| : .. : 1302
JX046487.1|Muju-V|N|South-Korea|M.r.|11-5|2011     : A. : 1302
JX046484.1|Muju-V|N|South-Korea|M.r.|11-4|2011|    : A. : 1302
JX028273.1|Muju-V|N|South-Korea|M.r.|11-1|2011|    : A. : 1302
DQ138128.1|Muju-V|N|South-Korea|E.r.|00-18|        : AG : 1302
DQ138140.1|Muju-V|N|South-Korea|E.r.|99-27|        : AG : 1302
DQ138133.1|Muju-V|N|South-Korea|E.r.|96-1|         : AG : 1302
DQ138142.1|Muju-V|N|South-Korea|E.r.|99-28|        : AG : 1302
```

References

1. Reynes JM, Carli D, Renaudin B, Fizet A, Bour JB, Brodard V, et al. Surveillance of human hantavirus infections in metropolitan France 2012–2016. [in French]. Bull Epidemiol Hebd (Paris). 2017;23:492–9.

2. Aitichou M, Saleh SS, McElroy AK, Schmaljohn C, Ibrahim MS. Identification of Dobrava, Hantaan, Seoul, and Puumala viruses by one-step real-time RT-PCR. J Virol Methods. 2005;124:21–6. PubMed http://dx.doi.org/10.1016/j.jviromet.2004.10.004

3. Evander M, Eriksson I, Pettersson L, Juto P, Ahlm C, Olsson GE, et al. Puumala hantavirus viremia diagnosed by real-time reverse transcriptase PCR using samples from patients with hemorrhagic fever and renal syndrome. J Clin Microbiol. 2007;45:2491–7. PubMed http://dx.doi.org/10.1128/JCM.01902-06

4. Garin D, Peyrefitte C, Crance JM, Le Faou A, Jouan A, Bouloy M. Highly sensitive Taqman PCR detection of Puumala hantavirus. Microbes Infect. 2001;3:739–45. PubMed http://dx.doi.org/10.1016/S1286-4579(01)01424-1

5. Korva M, Saksida A, Kejžar N, Schmaljohn C, Avšič-Županc T. Viral load and immune response dynamics in patients with haemorrhagic fever with renal syndrome. Clin Microbiol Infect. 2013;19:e358–66. PubMed http://dx.doi.org/10.1111/1469-0691.12218

6. Kramski M, Meisel H, Klempa B, Krüger DH, Pauli G, Nitsche A. Detection and typing of human pathogenic hantaviruses by real-time reverse transcription-PCR and pyrosequencing. Clin Chem. 2007;53:1899–905. PubMed http://dx.doi.org/10.1373/clinchem.2007.093245

7. Lagerqvist N, Hagström Å, Lundahl M, Nilsson E, Juremalm M, Larsson I, et al. Molecular diagnosis of hemorrhagic fever with renal syndrome caused by Puumala virus. J Clin Microbiol. 2016;54:1335–9. PubMed http://dx.doi.org/10.1128/JCM.00113-16

8. Bowen MD, Gelbmann W, Ksiazek TG, Nichol ST, Nowotny N. Puumala virus and two genetic variants of Tula virus are present in Austrian rodents. J Med Virol. 1997;53:174–81. PubMed http://dx.doi.org/10.1002/(SICI)1096-9071(199710)53:2<174::AID-JMV11>3.0.CO;2-J
